# Genetic fingerprint construction and genetic diversity analysis of sweet potato (*Ipomoea batatas*) germplasm resources

**DOI:** 10.1186/s12870-023-04329-1

**Published:** 2023-07-11

**Authors:** Zhongxia Luo, Zhufang Yao, Yiling Yang, Zhangying Wang, Hongda Zou, Xiongjian Zhang, Jingyi Chen, Boping Fang, Lifei Huang

**Affiliations:** Guangdong Provincial Key Laboratory of Crops Genetics and Improvement/Crops Research Institute, Guangdong Academy of Agricultural Sciences, Guangzhou, Guangdong 510640 China

**Keywords:** Sweet potato, Germplasm resources, Genetic markers, Genetic fingerprint, Genetic diversity

## Abstract

**Background:**

China is the largest producer of sweet potato in the world, accounting for 57.0% of the global output. Germplasm resources are the basis for promoting innovations in the seed industry and ensuring food security. Individual and accurate identification of sweet potato germplasm is an important part of conservation and efficient utilization.

**Results:**

In this study, nine pairs of simple sequence repeat molecular markers and 16 morphological markers were used to construct genetic fingerprints for sweet potato individual identification. Combined with basic information, typical phenotypic photographs, genotype peak graphs, and a two-dimensional code for detection and identification were generated. Finally, a genetic fingerprint database containing 1021 sweet potato germplasm resources in the “National Germplasm Guangzhou Sweet Potato Nursery Genebank in China” was constructed. Genetic diversity analysis of the 1021 sweet potato genotypes using the nine pairs of simple sequence repeat markers revealed a narrow genetic variation range of Chinese native sweet potato germplasm resources, and Chinese germplasm was close to that from Japan and the United States, far from that from the Philippines and Thailand, and the furthest from that from Peru. Sweet potato germplasm resources from Peru had the richest genetic diversity, supporting the view that Peru is the center of origin and domestication of sweet potato varieties.

**Conclusions:**

Overall, this study provides scientific guidance for the conservation, identification, and utilization of sweet potato germplasm resources and offers a reference to facilitate the discovery of important genes to boost sweet potato breeding.

**Supplementary Information:**

The online version contains supplementary material available at 10.1186/s12870-023-04329-1.

## Background

Sweet potato (*Ipomoea batatas* (L.) Lam.) is a hexaploid annual or perennial dicotyledonous root plant of the *Ipomoea* genus in the Convolvulaceae family. Sweet potato has the valuable characteristics of high yield, strong adaptability, and rich nutritional value [1–3]. It is a widely distributed crop grown in more than 120 countries and regions from the temperate zone south of 40° N to the tropics. Sweet potato has a wide range of uses [4–6], as fresh produce [7–9], in processed form [10, 11] as fodder, and as a leafy vegetable [12]. Sweet potato has become the world’s seventh largest food crop after rice, wheat, corn, potato, cassava, and barley [13, 14]. It can play an important role in solving the global food crisis and ensuring energy supply [15]. China has consistently been the world’s largest sweet potato producer. According to the Food and Agriculture Organization (FAO), in 2020, China’s total area under sweet potato was 225 × 10^4^ ha, accounting for 30.4% of the global planted area and 55% of the worldwide output [16].

Archaeological, historical, and biological studies have shown that Latin America is the key diversity center of sweet potato germplasm resources, also considered the most likely origin center of sweet potato [17, 18]. Clarke [19], Green [20], Roullier et al. [21], and Yen [22] have hypothesized three migration routes (the Kumara, Kamote, and Batata routes) from South America to the Pacific Islands, which led to the introduction of sweet potato to China at the end of the 16^th^ century through India and Myanmar (by land) [23] or the Philippines and Vietnam (by sea). China holds more than 2000 sweet potato germplasm accessions, mainly at the National Germplasm Guangzhou Sweet Potato Nursery Genebank (NGGSNG) and the National Germplasm Xuzhou Sweet Potato Test-tube Seedling Bank. The NGGSNG is the only national-level resource nursery for the outdoor vegetative preservation of sweet potato in China, including 1981 sweet potato resources from China and other countries and 1380 national catalogs.

Sweet potato propagates vegetatively and is thus, mainly preserved in the form of vegetative bodies, such as root tubers, seedlings, and test-tube seedlings. Resource identification has long been based on traditional phenotypic characteristics, which are susceptible to variation owing to environmental and subjective factors and thus, have low reliability. Furthermore, China’s collection methods and sources of sweet potato germplasm are diverse, with little standardization. Germplasm exchange and independent naming schemes among sweet potato planting operators or conservation organizations also result in the repeated introduction of germplasm, different names assigned to the same variety, and different varieties assigned under the same name, leading to unclear genetic relationships among germplasm accessions. This situation poses a challenge to cataloging and preserving sweet potato germplasm resources, selecting breeding parents, and promoting high-quality cultivars.

Many methods are available for biological species identification and genetic diversity analysis. Phenotypic markers remain important research tools owing to their advantages of intuitiveness and convenience [24]. The combination of phenotypic and molecular markers has become the preferred method for fingerprint construction and genetic variation analyses [25]. Considerable progress has been made in resource variety identification [26–28]. Genetic diversity research has been performed on sweet potatoes using morphological markers [29] and molecular markers [30–35] to analyze resources from multiple perspectives. Further, fingerprint or molecular ID databases of sweet potatoes have been constructed using molecular markers [36, 37]. Among the many types of molecular markers, simple sequence repeats (SSRs) and single nucleotide polymorphisms are recommended as the preferred markers for crop species identification and fingerprint database construction by the International Union for the Protection of New Varieties of Plants, International Seed Federation, and International Safe Transit Association, owing to their high polymorphism, wide distribution in the genome, good reproducibility, high throughput, and easy automation [38, 39].

To determine the genetic structure and diversity of sweet potato resources in China and provide a standardized guide to the available resources, we analyzed the population structure and genetic diversity of 1021 sweet potato genotypes from natural populations and a full-sib population of 55 clones using SSR molecular marker technology. Subsequently, we constructed a fingerprint database with a combination of phenotypic and molecular markers to provide evidence and references for variety identification, research, and further utilization of sweet potato germplasm resources in China. This work should also provide technical support for the in-depth exploration and utilization of sweet potato germplasm resources, including their collection, identification, cataloging, conservation, and selection of appropriate breeding parents.

## Results

### Phenotypic traits

**Fig. 1:**
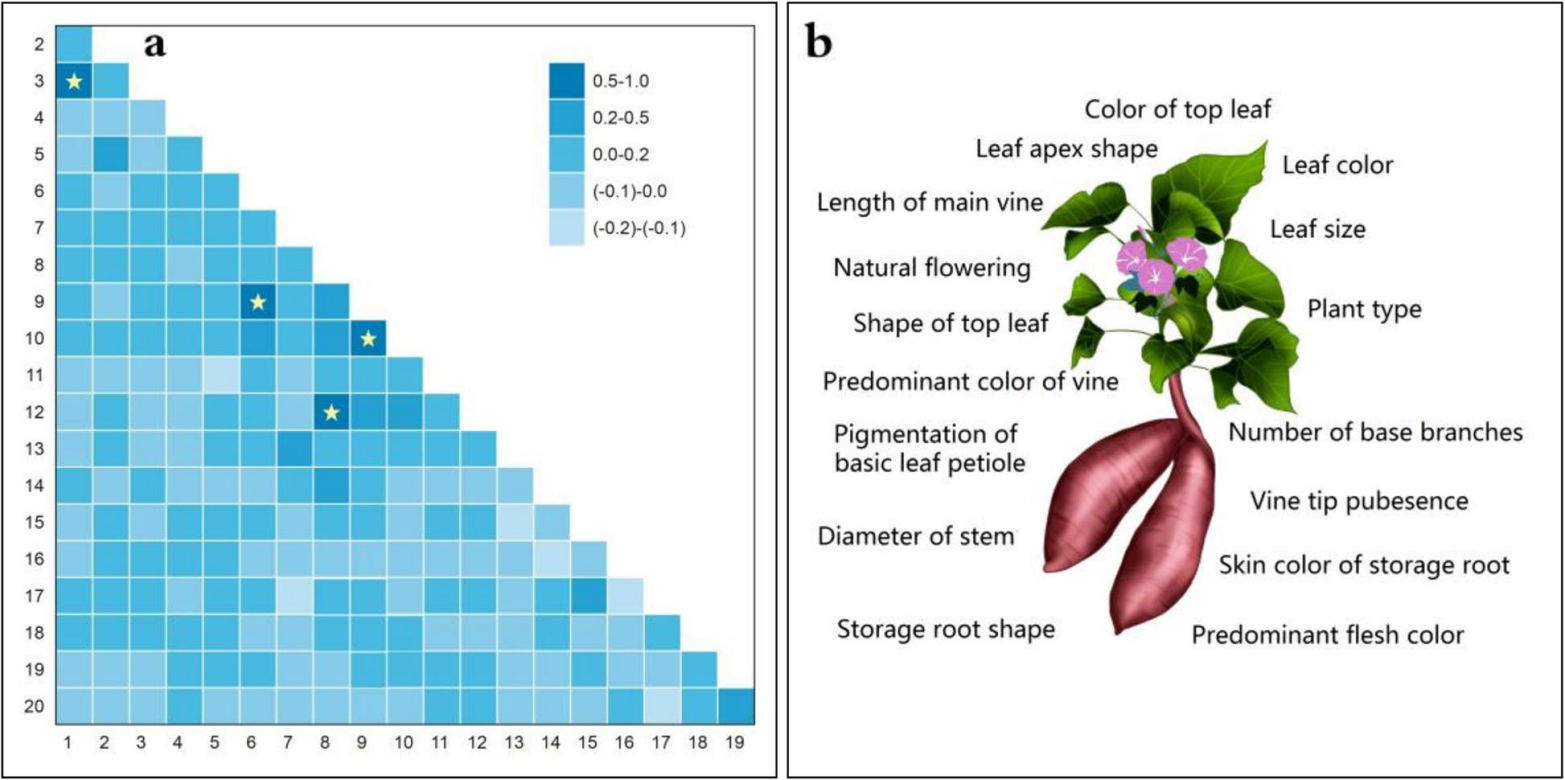
Kaiser-Meyer-Olkin measures and selection of morphological markers

Using 1021 sweet potato germplasm resources as a data set, 20 phenotypic traits were analyzed by factor analysis. In our Kaiser-Meyer-Olkin (KMO analysis) (Fig. 1a), only four features showed a KMO value > 0.5, namely, top leaf shape and leaf shape (0.8557), basic leaf vein pigmentation and basic leaf petiole pigmentation (0.6839), basic leaf vein pigmentation and main vein pigmentation color (0.5266), and petiole predominant color and vine predominant color (0.5405), accounting for 2.1% of the total data, indicating that the selected 20 phenotypic traits have good independence. Top leaf shape, basic leaf petiole pigmentation, main vein pigmentation color, and petiole predominant color were discarded, and 16 phenotypes were retained to construct the genetic fingerprint of sweet potato germplasm (Fig. 1b).

### Screening of SSR core primers

**Fig. 2:**
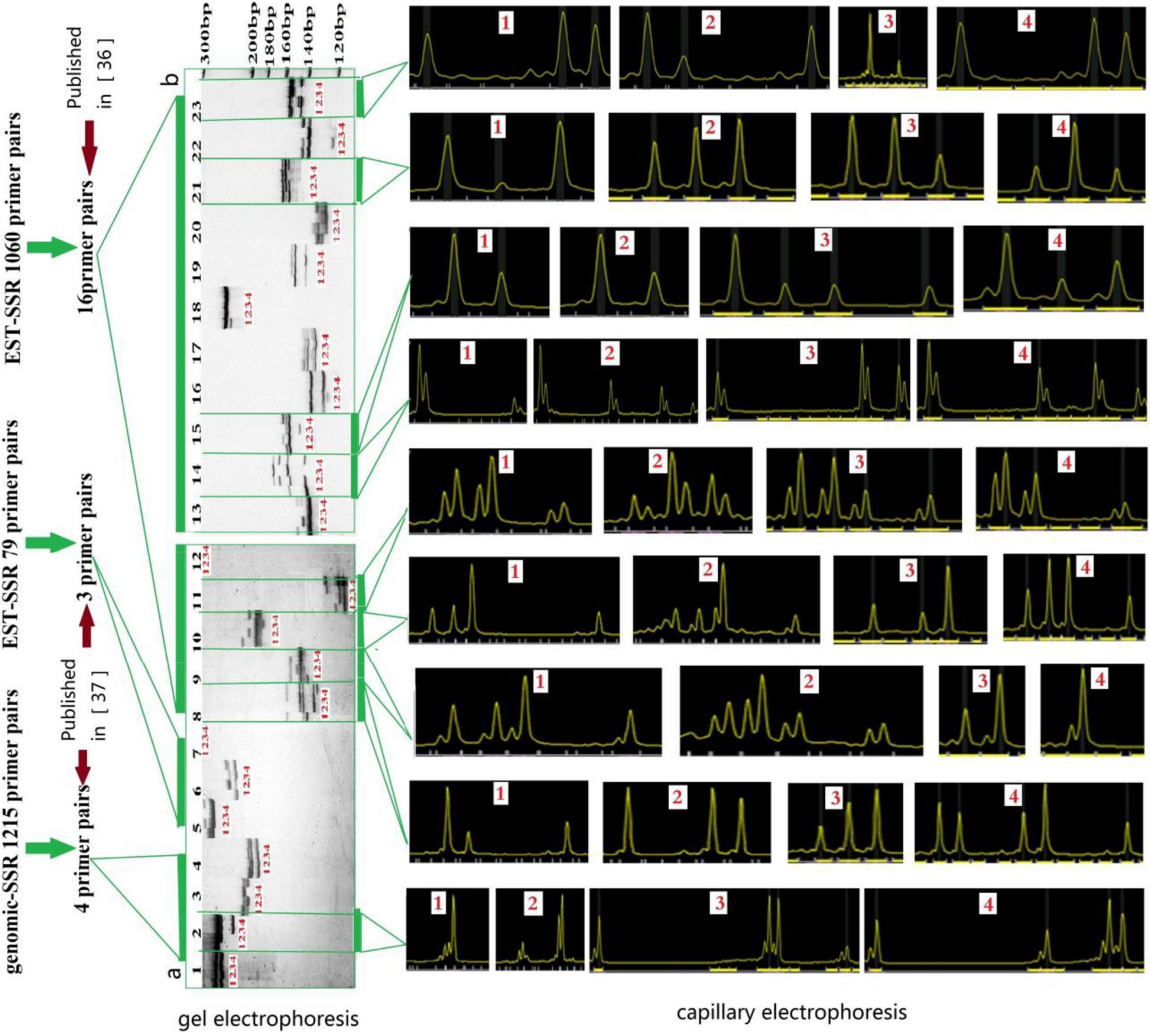
Electrophoresis and selection of core simple sequence repeat (SSR) primers. **a** Electrophoretic gel of primers 1–12, (**b**) Electrophoretic gel of primers 13–23. 1–23 blace numbers: SSR primers, 1–4 red numbers: sweet potato resources

In this study, we further screened 16 pairs of polymorphic primers [36, 40], and 7 pairs of primers published by Meng et al. [37]. Among the 23 pairs of SSR primers, nine primer pairs (Fig. 2 and S1, Table S1) showed high polymorphism and clear bands in four test materials, and these were selected to construct the genetic fingerprints and analyze the population genetic diversity of sweet potato germplasm.

### Genetic diversity and discrimination ability of primers

A total of 120 alleles (Na) were detected by the nine pairs of SSR primers in the 1021 germplasm samples (Table 1). The number of alleles detected by each pair of primers ranged from 8 to 21, with an average of 13.33. The number of genotypes ranged from 25 to 279, with an average of 104.44. The average Shannon’s genetic diversity index (I) of the population was 1.7439. The polymorphism information content (PIC) value ranged from 0.6079 to 0.8598, with an average value of 0.7516, demonstrating rich genetic diversity. The probability of identity (PI) and PIsibs values were used to evaluate the fingerprint discrimination ability of the selected SSR markers. The PI value ranged from 0.0291 to 0.167, and the cumulative value of the nine markers was 3.91 × 10^−11^. The PIsibs value ranged from 0.3939 to 0.5938, with a cumulative value of 9.22 × 10^−4^. A PI or PIsibs between 0.0001 and 0.001 indicated sufficient individual discrimination ability. These results confirmed that the selected molecular marker combinations have extremely high genetic discrimination ability for natural and full-sib populations of sweet potato germplasm.

**Table 1:** Statistics of the nine selected SSR markers

Primer name	Genotype No.	Na	Ne	I	PIC	PI	PIsibs
GDAAS0338	101	16	5.6488	1.9216	0.8000	0.0581	0.4218
GDAAS0694	25	10	3.0698	1.2241	0.6079	0.1670	0.5938
GDAAS0782	96	10	5.4010	1.8090	0.7893	0.0575	0.4087
GDAAS0819	64	11	4.6924	1.6728	0.7569	0.0752	0.3954
GDAAS0871	33	8	3.1475	1.2198	0.6156	0.1497	0.4384
GDAAS0911	278	18	7.8710	2.2103	0.8598	0.0291	0.3939
GDAAS0922	166	17	6.5412	2.0424	0.8294	0.0431	0.4257
GDAAS0940	62	9	4.2810	1.6701	0.7334	0.0850	0.5510
SPGS2	118	21	4.9348	1.9241	0.7749	0.0584	0.5625
Mean^a^	104.8	13	5.0653	1.7439	0.7519	3.91 × 10^−11^	9.22 × 10^−4^

*Na* Observed number of alleles, *Ne* Effective number of alleles, *I* Shannon’s information index, *PIC* Polymorphism information comtent, *PI* Average probability of two random individuals with the same genotype, *PIsibs* PI in the sibling population

^a^For PI and PIsibs, the values are the combined probabilities, which are the products of the PI or PIsibs of all individual loci

### Genetic diversity and principal component analysis of sweet potato germplasm

The test data from the nine SSR primer pairs in the 1021 sweet potato genotypes showed that the genetic distance between individuals ranged from 0.0145 to 1.376. The average for the population was 0.4619, indicating that the tested materials have a wide range of variation with significant genetic differences.

The genetic distances of six populations from the United States, Japan, Thailand, the Philippines, Peru, and China, comprising more than ten resources each, are summarized in Table 2. The Chinese population showed the greatest within-population genetic variation, followed by Japan, Peru, and the United States, whereas the Philippines and Thailand populations showed the smallest intra-population variation. Peru and China showed the largest variation between populations, whereas Thailand and the Philippines had the lowest variation. This indicated that China and Peru represent extreme points on the resource spectrum, whereas the resources of Thailand and the Philippines are the most similar. Diverse resources contain a wealth of specific genes that provide opportunities for creating new genetic material and developing novel cultivars.

**Table 2:** Genetic distances in different sweet potato populations

	USA	Japan	Thailand	Philippines	Peru	China
USA	0.017–1.055					
Japan	0.118–1.032	0.136–1.147				
Thailand	0.019–0.821	0.034–0.991	0.035–0.734			
Philippines	0.067–0.936	0.056–0.886	0.152–0.703	0.032–0.785		
Peru	0.178–1.165	0.160–1.119	0.214–1.165	0.214–1.096	0.190–1.068	
China	0.017–1.075	0.015–1.253	0.019–0.885	0.015–1.127	0.015–1.376	0.014–1.214

**Fig. 3:**
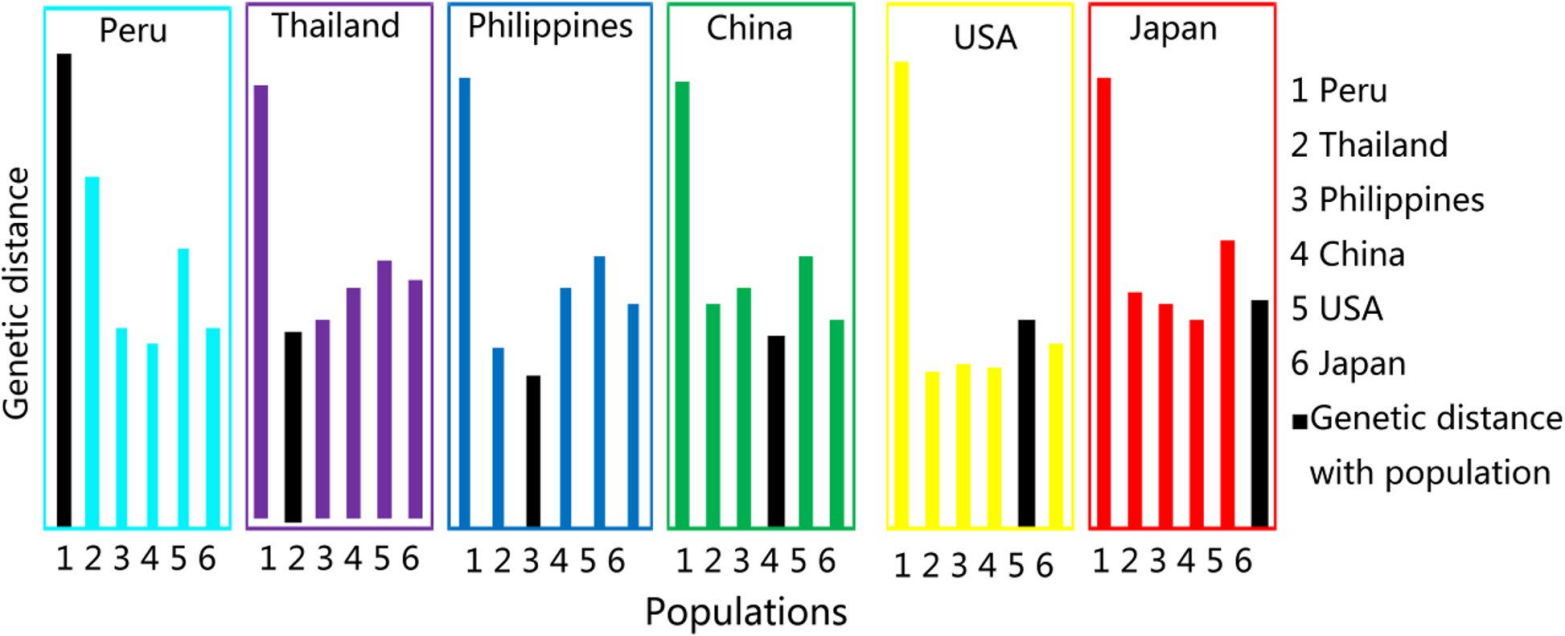
Average genetic distances in sweet potato populations by country

The comparative analysis of the average genetic distance for populations is shown in Fig. 3. The average genetic distance in the Peruvian population was significantly higher than that of the other populations, at both the within- and between-population levels. However, the mean within-population genetic diversity (0.5275) was higher than that of between populations, indicating that Peru has the largest genetic variation in sweet potato resources. By contrast, the mean within-population genetic diversity from the Philippines, Thailand, and China were 0.4515, 0.4490, and 0.4563, respectively, which showed similar characteristics, smaller than that of between populations, indicating small genetic variation within these populations.

**Fig. 4:**
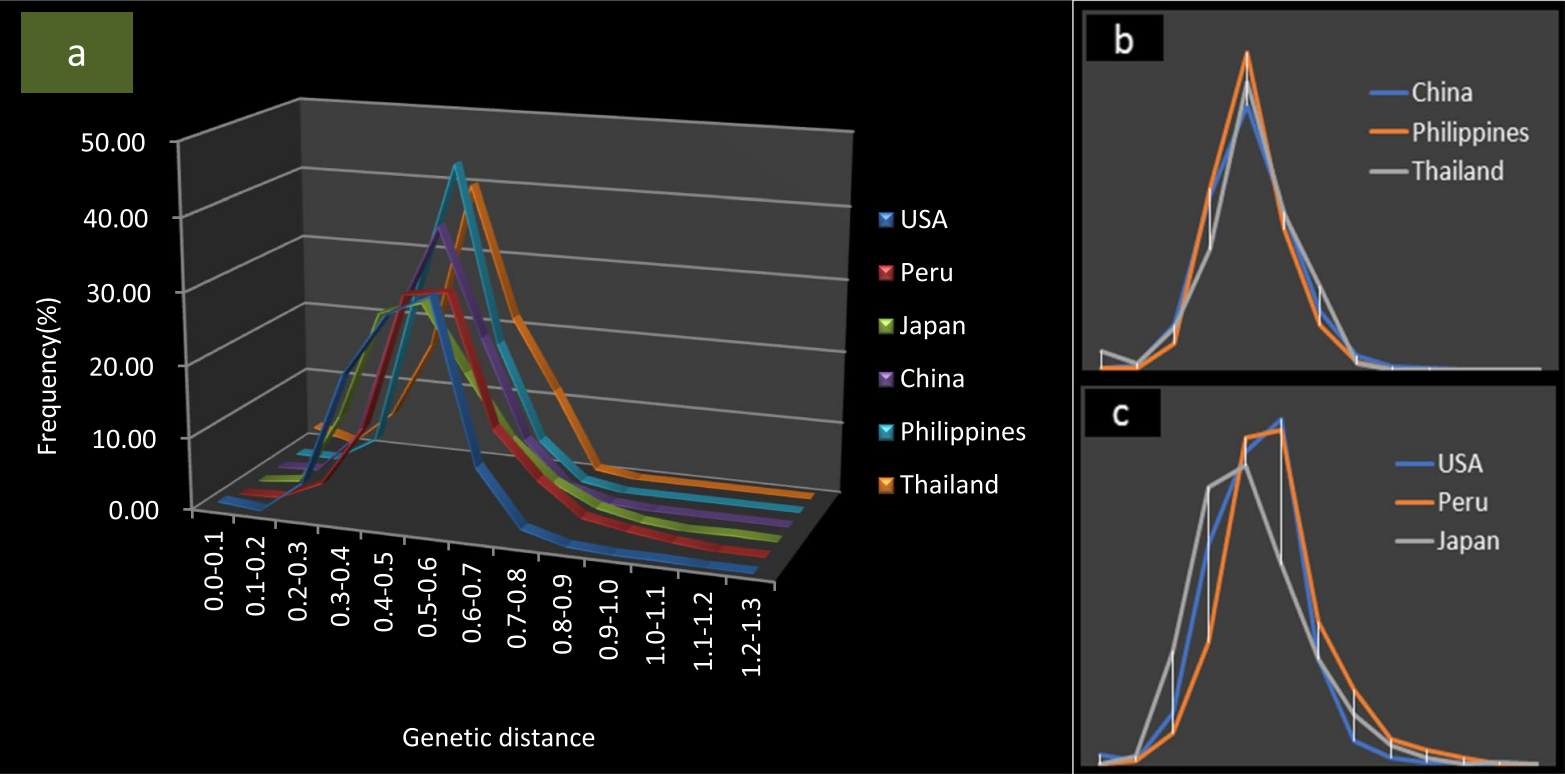
Variation trends in different genetic distance ranges for sweet potato germplasm

Figure 4a shows the genetic distances for individual resources of each population by country. The peak shapes of resources from China, Thailand, and the Philippines were high and sharp (Fig. 4b), indicating that most of the resources in these populations are distributed within an extremely narrow range of genetic distance. The peak shapes for the Japan, United States, and Peru resources were similar, with a relatively wider peak distance and right tails declining slowly across a larger genetic distance, indicating that most of the resources in these populations have a wide genetic distribution range and have more unique alleles (Fig. 4c).

**Fig. 5:**
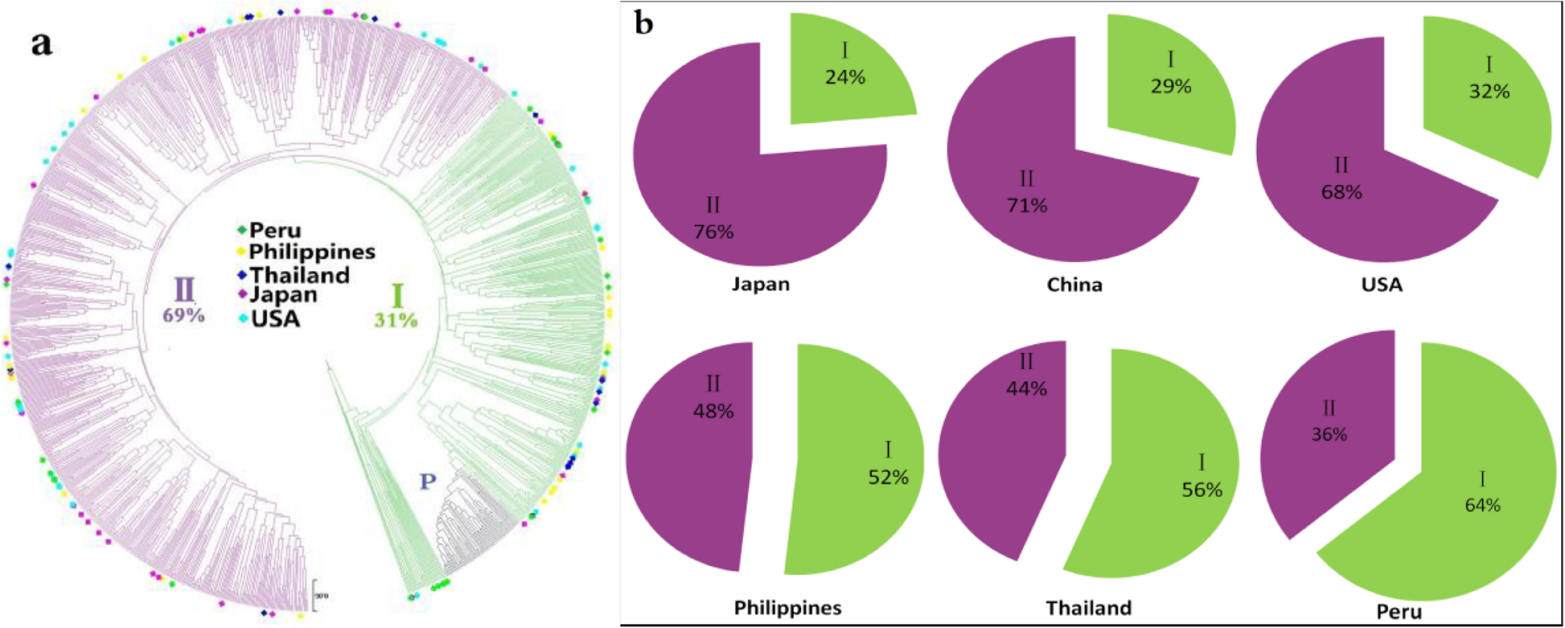
Unweighted pair group method with arithmetic mean dendrogram of sweet potatoes. For the analysis, 1076 sweet potatoes (1021 samples from natural populations and 55 from sib populations) were considered based on the SSR markers

Clustering divided all materials into two groups (Group I and Group II) at the nearest location (0.4619) to the mean value (0.4648) (Fig. 5a). Group I contained 317 of the 1021 materials (31.05%), and Group II contained 704 (68.95%). Group P represents a full-sib family and is not included in Group I. In terms of population genetic structure, Group I represents materials with more distant relatives, and Group II represents closely related materials. Analysis of the distribution of resources from different sources across the two groups (Fig. 5b) showed the degree of genetic diversity and dispersion in decreasing order as follows: Peru, Thailand, the Philippines, the United States, China, and Japan.

The full-sib families with 55 clones clustered together completely in Group P, with an average genetic distance of 0.2277. These results further demonstrate the high discrimination efficiency of the primer combinations selected in this study for sweet potato resources.

**Fig. 6:**
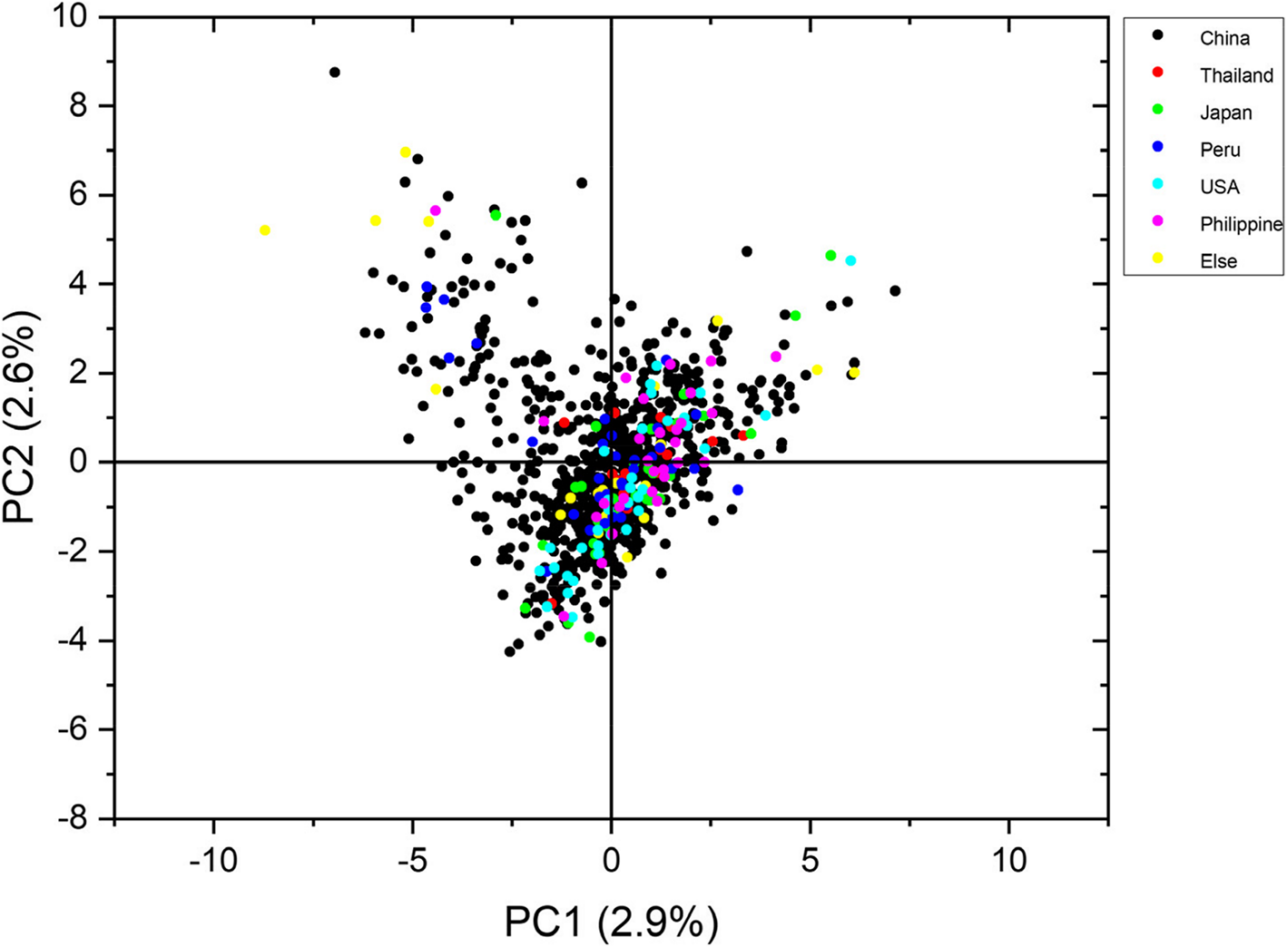
Principal component analysis of 1021 accessions based on 9 pairs SSR markers

PCA results also showed that the 1021 sweet potato germplasms were not clustered together according to their regions (Fig. 6), but there are certain differences in the distribution range between different countries,which was consisdent with the results of the cluster diagram and genetic analysis.

### Genetic fingerprint construction for sweet potato germplasm

**Fig. 7:**
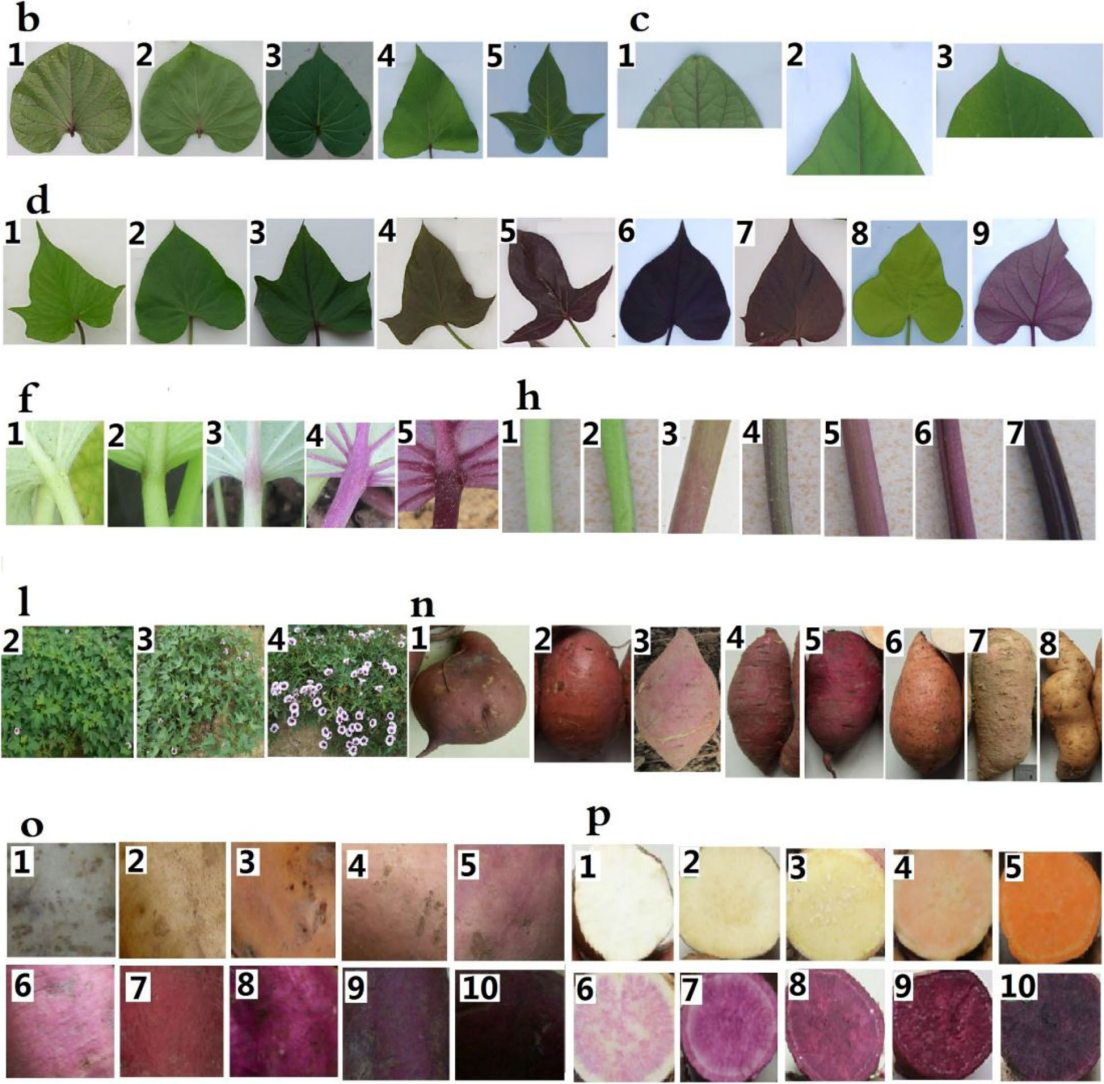
Phenotypic codes and pictures for sweet potato germplasm

**Fig. 8:**
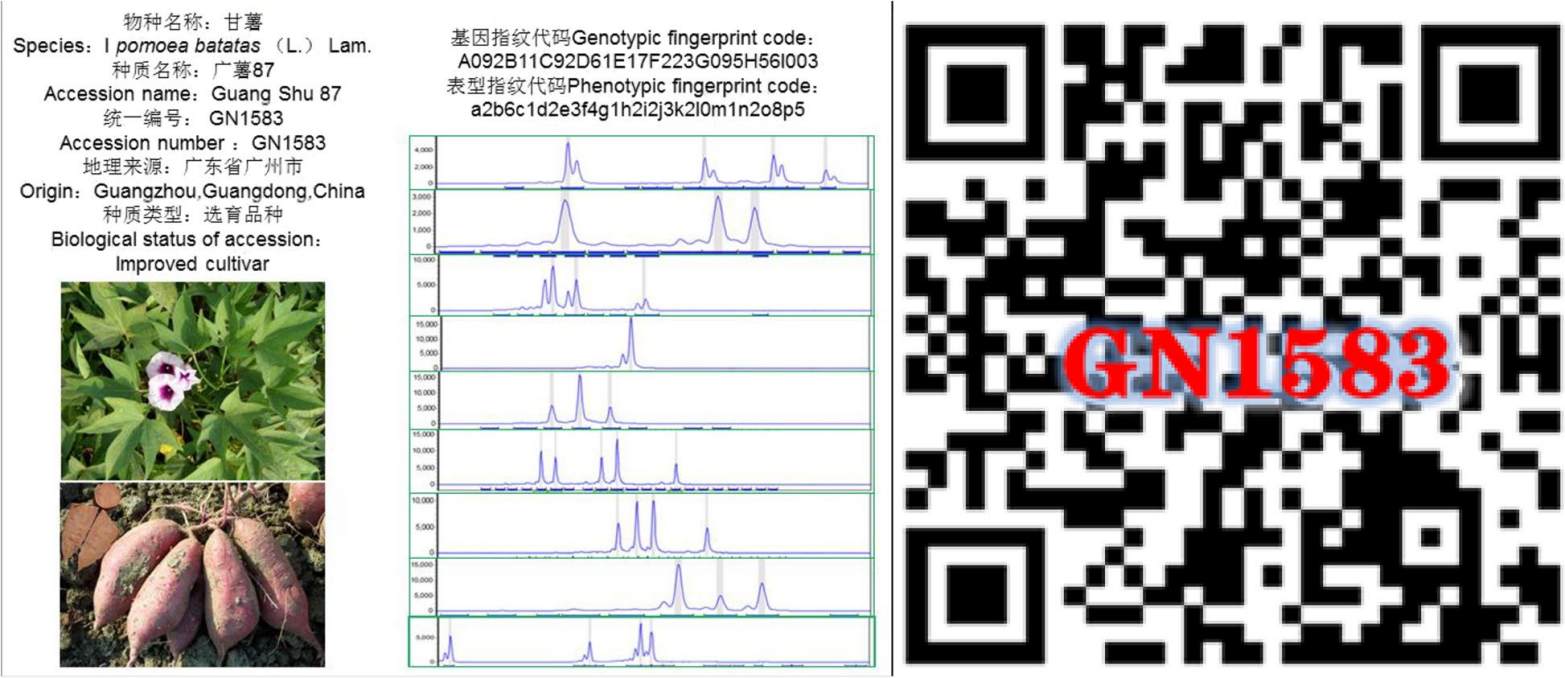
Two-dimensional code of the sweet potato genotype Guangshu 87

Finally, we constructed a unique genetic fingerprint database for sweet potato germplasm, including genotypic (uppercase letters for molecular markers and numerals for genotypes, Table S3) and phenotypic (lowercase letters for traits and numerals as a phenotypic code, Table S4) fingerprints based on nine pairs of molecular markers and 16 phenotypic traits (Table 3, Fig. 7). For example, for germplasm resource Guangshu 87, the molecular marker is GDAAS0338, coded as A, and its genotypic (gene locus fragment length of 142159166172 bp) code is 092, resulting in the genotypic fingerprint A092B11C92D61E17F223G095H56I003. The color code of the top leaf is a, and the phenotype is green, which is coded as 2, resulting in the phenotypic fingerprint a2b6c1d2e3f4g1h2i2j3k2l0m1n2o8p5. In this manner, combining the genetic fingerprints with basic information (Table S2), representative phenotype photographs, and the molecular marker scanning peaks (Fig. S2), two-dimensional codes (Fig. 8) of each germplasm resource were generated, and the fingerprint map and its two-dimensional code database, including 1021 resources, were constructed.

**Table 3:** Genetic markers and codes of genetic fingerprints for sweet potato germplasm

Primer name	Code	Trait name	Code	Trait name	Code
GDAAS0338	A	Top leaf color	a	Stem diameter	i
GDAAS0694	B	Leaf shape	b	Number of base branches	j
GDAAS0782	C	Leaf apex shape	c	Plant type	k
GDAAS0819	D	Leaf color	d	Natural flowering	l
GDAAS0871	E	Leaf size	e	Main vine length	m
GDAAS0911	F	Basic leaf vein pigmentation	f	Stored root shape	n
GDAAS0922	G	Vine tip pubescence	g	Stored root skin color	o
GDAAS0940	H	Predominant vine color	h	Predominant flesh color	p
SPGS2	I

## Discussion

At present, the identification of sweet potato germplasm resources is mainly based on phenotypic characteristics, which can be significantly affected by environmental conditions and cultivation methods, as well as subjective assessments, leading to errors and inaccurate results. Despite these limitations, morphological identification remains an indispensable method for studying the genetic diversity of germplasm, given its intuitiveness and convenience. It is also an important technique for species identification and the determination of parent combinations in breeding programs. Alternatively, molecular identification represents the most reliable method for identifying a crop variety. Molecular markers can be used to distinguish and identify genetic differences between sweet potato genotypes at the DNA level, without being affected by external environmental conditions and cultivation methods. DNA analysis is the most effective genetic analysis method [41, 42], and SSR markers offer advantageous features of simple operation, good polymorphism, and low cost, with strong specificity and good reproducibility, making them a suitable tool for the construction of fingerprints for a large number of resources. DNA fingerprints based on molecular markers offer rich information on polymorphisms with a high degree of individual specificity and environmental stability. They can help identify biological differences between phenotypically similar individuals, similar to human fingerprints. Moreover, DNA fingerprinting is fast and accurate, making it a powerful tool for identifying species and strains, and it is particularly suitable for identifying germplasm resources.

In this study, we constructed a two-dimensional code database containing 1021 sweet potato germplasm resources using nine pairs of SSR molecular marker primers that can completely distinguish among all materials, along with 16 phenotypic traits and basic morphological information of the experimental materials, providing a unique genetic fingerprint for each resource.

Genetic diversity is the sum of the genetic variation of populations and individuals [43]. A change in genetic diversity is essentially a change in gene number or frequency [44]. A population with higher genetic diversity will have a stronger ability to adapt to environmental changes. During the long-term targeted artificial breeding and natural selection processes of sweet potato, homogeneity among varieties increased, while genetic diversity decreased. Long-term directional selection imposed in the breeding process to select for traits of interest also caused narrowing of the genetic base and gene loss, resulting in an overall decrease in population diversity [45]. Thus, research on the genetic diversity of sweet potato germplasm resources is critical for accurate identification, the discovery of new genes, and the selection and breeding of new varieties [46].

In this study, no significant geographical patterns were found between the resources from different countries or from the different provinces and cities of China, which is consistent with the findings of Meng et al. [37]. One explanation for this lack of geographical diversity is that with the development of advanced biological technology and the convenience of transportation, the gene migration speed of sweet potato might have accelerated, resulting in the fading or disappearance of regional boundaries. Nevertheless, some local varieties have gradually evolved into new local varieties due to long-term and multiple rounds of asexual (vegetative) reproduction and environmental influences.

The cluster dendrogram showed that a small number of varieties clustered into one group alone or few varieties grouped together, with wide genetic distances from other materials. This phenomenon may be related to the hybrid incompatibility and asexual reproduction mode of sweet potato. Hybrid incompatibility can readily isolate certain germplasm resources and genes from genetic exchange, while asexual reproduction greatly reduces the gene exchange frequency, leading to a targeted mutation in sweet potato.

Many studies have shown that sweet potato cultivars originated in tropical regions of America, with Mexico, Venezuela, or Peru considered as the origin and domestication centers [47–51]. There are an estimated 8000 accessions of sweet potato germplasm worldwide [52, 53]. In this study, we focused on 1021 of these resources from 16 countries and institutions covering six continents, representing virtually the entire global distribution area. Our analysis shows that the individual- and population-level genetic diversity of sweet potato germplasm resources from Peru are significantly higher than those of other populations, supporting the theory that Central/ South America is the center of origin of sweet potato.

We also found that Chinese sweet potato has the closest genetic relationship with sweet potato resources from Japan and the United States. The furthest relationship was with the germplasm from the Philippines and Thailand, with the latter two being the most closely inter-related. It is speculated that after sweet potatoes were first domesticated in Peru, gene flow occurred toward other places. Some germplasm may have first traveled through the United States, spreading to China and Japan, whose resources show an extremely close genetic relationship. Other sweet potato genes migrated to Thailand and the Philippines separately.

By combining genotypic and phenotypic information, the constructed genetic fingerprints can help solve current identification issues of the same variety being assigned different names or different varieties under the same name (i.e., synonymy and homonymy) and aid in dispelling cultivar confusion. If the genetic fingerprint data of two materials are consistent at both the phenotypic and genotypic levels, they will be considered the same variety. Thus, establishing genetic fingerprints of sweet potato germplasm resources will improve the efficiency and accuracy of variety identification, providing a theoretical foundation for future identification, protection, and breeding of sweet potato germplasm resources. The fingerprint database of sweet potato germplasm resources constructed in this study represents the largest such resource in the world to date.

A genetic fingerprint database containing 1021 sweet potato germplasm resources was constructed with nine molecular markers and 16 morphological markers as core indicators. The genetic variation of native Chinese sweet potato germplasm resources was found to be narrow, demonstrating a closer relationship to those from Japan and the United States and the furthest relationship with resources from Peru. This study provides further scientific and technical support for the conservation, identification, research, and utilization of sweet potato germplasm resources.

## Materials and methods

### Plant materials

A total of 1021 sweet potato germplasm resource materials (Table S4) and a full-sib family containing 55 clones were provided by the Crop Research Institute of Guangdong Academy of Agricultural Sciences. These germplasm resources are now conserved in the NGGSNG in China.

### Extraction of genomic DNA

The experimental materials were planted in the Guangzhou Baiyun base of the Guangdong Academy of Agricultural Sciences, and DNA was extracted from the young leaves 45–50 d after planting. Genomic DNA was extracted using the ionic detergent cetyltrimethylammonium bromide, and DNA quality was examined by 2.0% agarose gel electrophoresis. The concentration of DNA was determined on a NanoDrop 2000 ultraviolet spectrophotometer. The DNA was then diluted to 50 ng μL^−1^ and stored at −20 ℃ for future use. The DNA stock solution was stored at −80 ℃. Sampling and DNA extraction were completed in 2018 and 2019.

### SSR primer screening and evaluation

Twenty-three pairs of sweet potato SSR primers were used in the experiment (Table 4 and Fig. 2), of which seven pairs (Nos. 1–7) were published in 2018 [37], and 16 pairs (Nos. 8–23) were from 2014 [36]. Common primers were synthesized by Sangon Biotech (Shanghai, China). A monochromatic fluorescent primer was used for capillary electrophoresis, and the upstream 5′-end was labeled with FAM (blue), which was synthesized by General Biosystems Co., Ltd. (Anhui, China).

**Table 4:** Twenty-three SSR primer pairs that were screened in this study

No.	Primer name	F sequences (5′-3′)	R sequences (5′-3′)
1	SPGS1	CTCGCTCACGATTGATGATG	CGGAGTGGTAGGGCTAAACC
2	SPGS2	AGACTGGACTCCCAGAAGCA	CAAGCAGTCAGAAGTCGACAA
3	SPGS3	CCGATCATTCCCAAACTCAT	AGCAGGGGAGACGTAAGGAT
4	SPGS4	ATCAGAGCCTGGCAAAGAAA	GGGGAACTTCAGCTAAGCAA
5	SPES1	AATGCCAACCAAAGCCATAG	CGATGACAAAGCAGCTGAAG
6	SPES2	TCGGAACGGAGATAGATTGG	AAGCAAGAAAAAGAAGTGAAGGAA
7	SPES3	ATGACATCCCAAGGAGCATC	GAGGTTGAGGGCGTATCTGA
8	GDAAS0911	CTTCGCACTCGCATCTCT	GGTATGGTGTAAGTTGTTGTTC
9	GDAAS0819	GAGGATCAACTGCGTCTTCTG	CGTGAACCCAGCCTAACAAG
10	GDAAS0922	CGCCTTCGTTAATAACCACTC	CCTGCTTAATCCGATCCTCTC
11	GDAAS0782	GCACCACATTAATAATGATGCC	TGTTGAAGGTTCTGATGGAGTA
12	GDAAS0926	GCTCATCTTGGATCTCTTGAAG	CGAAGGAGGGTTTAGGGTTTA
13	GDAAS0385	AACTATTCTTGTCCAATCTGCG	GCCATGTGTATTCCTGATTCC
14	GDAAS0338	GCAGCGGATGGAATACTCA	TCTACACGACTACCAACTACAA
15	GDAAS0940	CCGATGATTATAGCACTTACG	GGTTCACCTTCCACACTC
16	GDAAS0848	CGCTTCCTTCTTCTGATTAGA	GCAGTGCAGTGAGTTGAG
17	GDAAS0858	GCACTGCCAGCAAACCAA	TTCCTCGTCCATGAAGAACAC
18	GDAAS0354	GTATCTTCCAGTTCAGTTCCACAT	ATCCATCCACCACGCAATCA
19	GDAAS0914	TTGATGGCAACGCAATCT	CCTCTCGTCCACTTGATG
20	GDAAS0843	AACAGGAGCAGCACCATT	TGACCCAACCCAGAAAGATT
21	GDAAS0871	GCAGAGTGAGAATTAGAGTT	GTCCCTTCTTTGCCAGTA
22	GDAAS0360	TGTGTAGACTCACTCAATCATCTG	GGTGTATGCGTAATCTGGAAGG
23	GDAAS0694	GTCTAAGATGGAGTGAGGAA	GATCAAGGCTGAAGTTACG

Four sweet potato resources (1: Guangzishu 2, 2: Guangzishu 8, 3: Guangshu 72, 4: Guangshu 87) were selected from the experimental materials to screen the 23 pairs of SSR core primers by polyacrylamide gel electrophoresis (PAGE)-silver staining, with three replicates for each primer. The primers exhibiting high polymorphism and good repeatability were selected as core primers for genetic diversity analysis and genetic fingerprint construction of sweet potato germplasm resources.

The total volume used for polymerase chain reaction (PCR) was 20 μL, including 2 μL of DNA template (50 ng μL^−1^), 0.2 μL TaqDNA polymerase, 0.3 μL each of the upstream and downstream primer (20 μM), 0.4 μL dNTPs, 2 μL buffer, and 14.8 μL ddH_2_O. The following PCR cycling program was used: pre-denaturation at 94 ℃ for 5 min, denaturation at 94 ℃ for 30 s, annealing at 54 ℃ (varied according to primer) for 35 s, and extension at 72 ℃ for 40 s for a total of 35 cycles. The final product was extended at 72 ℃ for 3 min. The products were separated and visualized by PAGE-silver staining on a 6% gel, and fluorescence capillary electrophoresis was carried out on a 3730XL DNA analyzer (Applied Biosystems, Thermo Fisher Sci Corp., Waltham, MA, USA).

### Selection of phenotypic traits

According to the Description and Data Standard for Sweet Potato Germplasm Resources [54], 20 phenotypic traits (Table 5) were selected for fingerprint construction. The phenotypic data were derived from the sweet potato germplasm resources management database of the NGGSNG.

**Table 5:** Twenty phenotypic traits and grading criteria

No.	Phenotypic traits	Assignment of phenotypic traits
1	Top leaf shape	1: Round; 2: Reniform; 3: Cordate; 4: Acuminate-cordate; 5: Triangular; 6: Incised
2	Top leaf color	1: Light green; 2: Green; 3: Purple-green; 4: Brown-green; 5: Light purple; 6: Purple; 7: Brown; 8: Golden-yellow; 9: Red
3	Leaf shape	1: Round; 2: Reniform; 3: Cordate; 4: Acuminate-cordate; 5: Triangular; 6: Incised
4	Leaf apex shape	0: Absent; 1: Acute; 2: Blunt
5	Leaf color	1: Light green; 2: Green; 3: Purple-green; 4: Brown-green; 5: Light purple; 6: Purple; 7: Brown; 8: Golden-yellow; 9: Red
6	Main vein pigmentation color	1: Light green; 2: Green; 3: Yellow; 4: Light purple; 5: Purple; 6: Purple speckle
7	Leaf size (Length × Width (cm^2^))	1: Small (< 80); 2: Medium (80–160); 3: Large (> 160)
8	Petiole predominant color	1: Light green; 2: Green; 3: Light purple; 4: Purple; 5: Dark purple
9	Basic leaf vein pigmentation	1: Light green; 2: Green; 3: Light purple; 4: Purple; 5: Dark purple
10	Basic leaf petiole pigmentation	1: Light green; 2: Green; 3: Light purple; 4: Purple; 5: Dark purple
11	Vine tip pubescence	0: None; 1: Little; 2: Moderate; 3: More
12	Vine predominant color	1: Light green; 2: Green; 3: Mauve; 4: Light purple; 5: Purple; 6: Dark purple; 7: Brown
13	Stem diameter (mm)	1: Thin (< 4); 2; Moderate (4–6); 3: Thick (6–8); 4: Extra-thick (> 8)
14	Number of base branches	1: Little (< 6); 2: Moderate (6–10); 3: Many (10–20); 4: Very many (> 20)
15	Plant type	1: Erect; 2: Semi-erect; 3: Prostrate; 4: Scramble
16	Natural flowering	0: None; 1: Contingent; 2: Sparse; 3: Moderate; 4: Profuse
17	Main vine length (cm)	1: Short (< 100); 2: Moderate (100–200); 3: Long (200–300); 4: Very long (> 300)
18	Stored root shape	0: None; 1: Rotundity; 2: Short elliptic; 3: Elliptic; 4: Long elliptic; 5: Obovate; 6: Ovate; 7: Rectangle; 8: Curve; 9: Anomalous
19	Stored root skin color	1: White; 2: Light yellow; 3: Brown-yellow; 4: Yellow; 5: Brown; 6: Pink; 7: Red; 8: Mauve; 9: Purple; 10: Dark purple
20	Predominant flesh color	1: White; 2: Light yellow; 3: Yellow; 4: Orange-yellow; 5: Orange-red; 6: Pink; 7: Red; 8: Mauve; 9: Purple; 10: Dark purple

### Data analyses

The genetic parameters of SSR typing from the raw capillary electrophoresis data were analyzed using Fragment (plant) analysis software in Genemaker. The labeled molecular weight in each lane was compared with the position of the peak value of each sample; the amplified band at the same migration position was marked as 1, and the non-amplified band was marked as 0 to construct a binary data matrix of 0 s and 1 s. The NTSYSpc 2.11 software package was used to calculate the genetic distance between materials. The number of alleles (Na), allele frequency, and genotype frequency were statistically analyzed. Shannon’s information index (I), number of effective alleles (Ne), and PIC were calculated according to the 0/1 data matrix using the following formulae:

$$\begin{array}{l}\mathrm{Ne}=1/\sum_{\mathrm i=1}^{\mathrm n}\mathrm P_{\mathrm i}^2\\\mathrm I=1/\sum_{\mathrm i=1}^{\mathrm n}{\mathrm P}_{\mathrm i}{\mathrm{InP}}_{\mathrm i}\\\mathrm{PIC}=1-\sum_{\mathrm i=1}^{\mathrm n}\mathrm P_{\mathrm i}^2-\sum_{\mathrm i=1}^{\mathrm n-1}\sum_{\mathrm j=\mathrm i+1}^{\mathrm n}2\mathrm P_{\mathrm i}^2\mathrm P_{\mathrm j}^2,\end{array}$$

where n is the total number of alleles and Pi is the allele frequency of the i^th^ allele. MEGA7.0.26 was used to perform cluster analysis, and the cluster dendrogram was constructed using the unweighted pair group method with the arithmetic mean approach. SPSS 21.0 analysis software was used for the KMO test of tabular data, and principal component analysis(PCA) was performed using Origin 2021. The KMO test was used to assess correlations and partial correlations between variables; a test outcome of < 0.5 indicates that each variable is independent and unsuitable for factor analysis.

Two parameters were used to evaluate the individual recognition ability of nine pairs of SSR markers, PI, and PIsibs, representing the average probability of two random individuals with the same genotype, in the natural (1021 germplasm samples) and sibling (55 full-sib clones) population, respectively, calculated using the following equations:

$$\begin{array}{l}PI=2\left(\Sigma p_i^2\right)^2-\Sigma p_i^4\\PIsibs=0.25+\left(0.5\Sigma p_i^2\right)+\left[0.5\left(\Sigma p_i^2\right)^2\right]-\left(0.25\Sigma p_i^4\right),\end{array}$$

where *p*_*i*_ represents the gene frequency of the i^th^ allele at a given locus.

### Genetic fingerprint construction

Molecular markers combined with phenotypic markers were used to construct the genetic fingerprint of sweet potato germplasm. The selected genotypic marker combinations were labeled with uppercase letters (A, B, C, etc.), and the genotypes were sorted and labeled with Arabic numerals; similarly, different phenotypic markers were labeled with lowercase letters (a, b, c, etc.), and the phenotypes were assigned Arabic numerals. Using this method, a series of numbers composed of uppercase or lowercase letters plus Arabic numerals were formed for each resource, providing genetic and phenotypic fingerprints of the germplasm. We combined the genotypic and phenotypic fingerprints with basic information, phenotype photographs, and molecular marker scanning peaks graph to develop a two-dimensional code of each germplasm resource using Golang on the local server (http://192.168.3.177), which was ultimately used to build genetic fingerprints and the two-dimensional code database of sweet potato germplasm resources.

## Supplementary Information

**Additional file 1: Figure S1.** Electrophoretic gels of simple sequence repeat (SSR) primers. **Figure S2.** Scanning peak graphs of all 943 genotypics.

**Additional file 2: Table S1.** Genomic positons of core simple sequence repeat (SSR) primers. **Table S2.** Basic information of 1021 sweet potato germplasm resources. **Table S3.** Genotypics of 1021 sweet potato germplasm resources. **Table S4.** Phenotypics of 1021 sweet potato germplasm resources.

## Data Availability

The datasets generated during and/or analyzed during the current study are available from the corresponding author on reasonable request.

## Abbreviations

NGGSNG: National Germplasm Guangzhou Sweet Potato Nursery Genebank
SSR: Simple sequence repeats
KMO: Kaiser-Meyer-Olkin
PIC: Polymorphism information content
PI: Probability of identity
PAGE: Polyacrylamide gel electrophoresis
PCR: Polymerase chain reaction
