# Supplementary material for: Genetic fingerprint construction and genetic diversity analysis of sweet potato (Ipomoea batatas) germplasm resources
Source: BMC Plant Biol. 2023 Jul 11;23:355. doi: 10.1186/s12870-023-04329-1 (PMC10334575; doi:10.1186/s12870-023-04329-1)
Supplement: Supplementary file 1 — Additional file 1: Figure S1. Electrophoretic gels of simple sequence repeat (SSR) primers. Figure S2. Scanning peak graphs of all 943 genotypics. [file 12870_2023_4329_MOESM1_ESM.pdf]

Figure S1. Electrophoretic gels of simple sequence repeat (SSR) primers

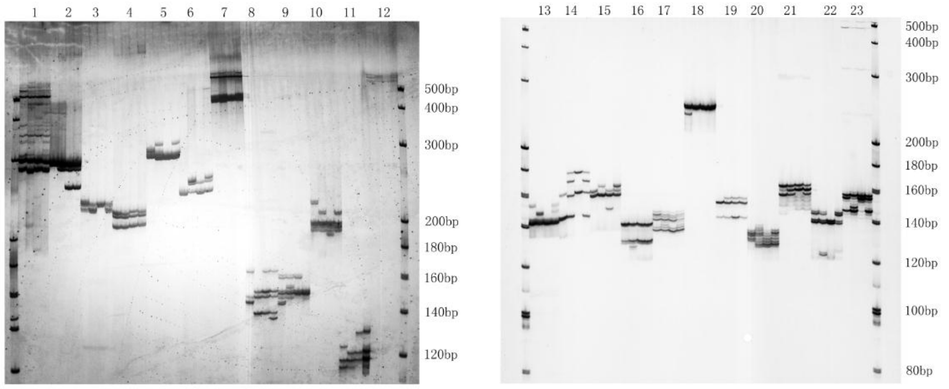

Figure S2. Scanning peak graphs of all 943 genotypics.

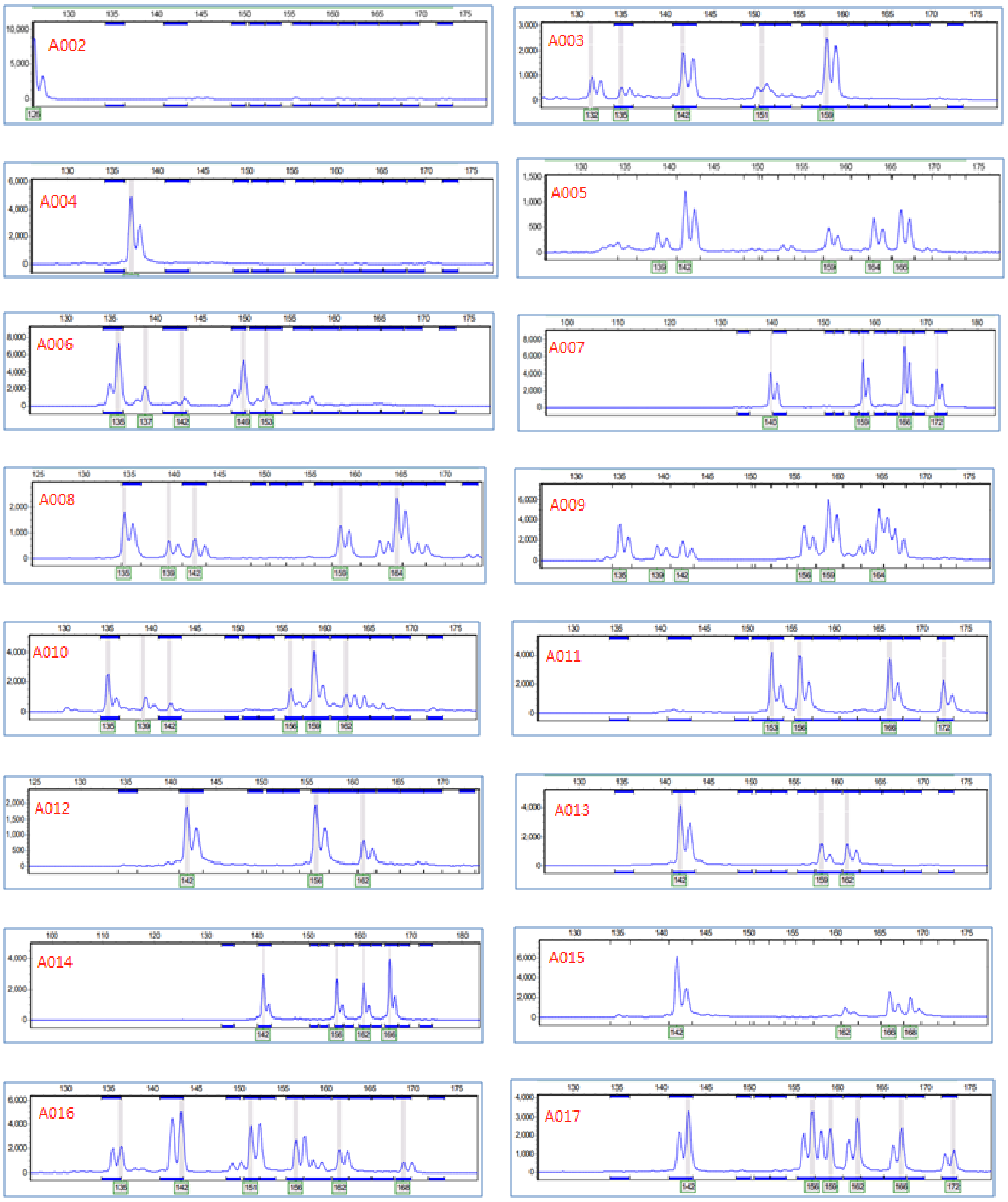

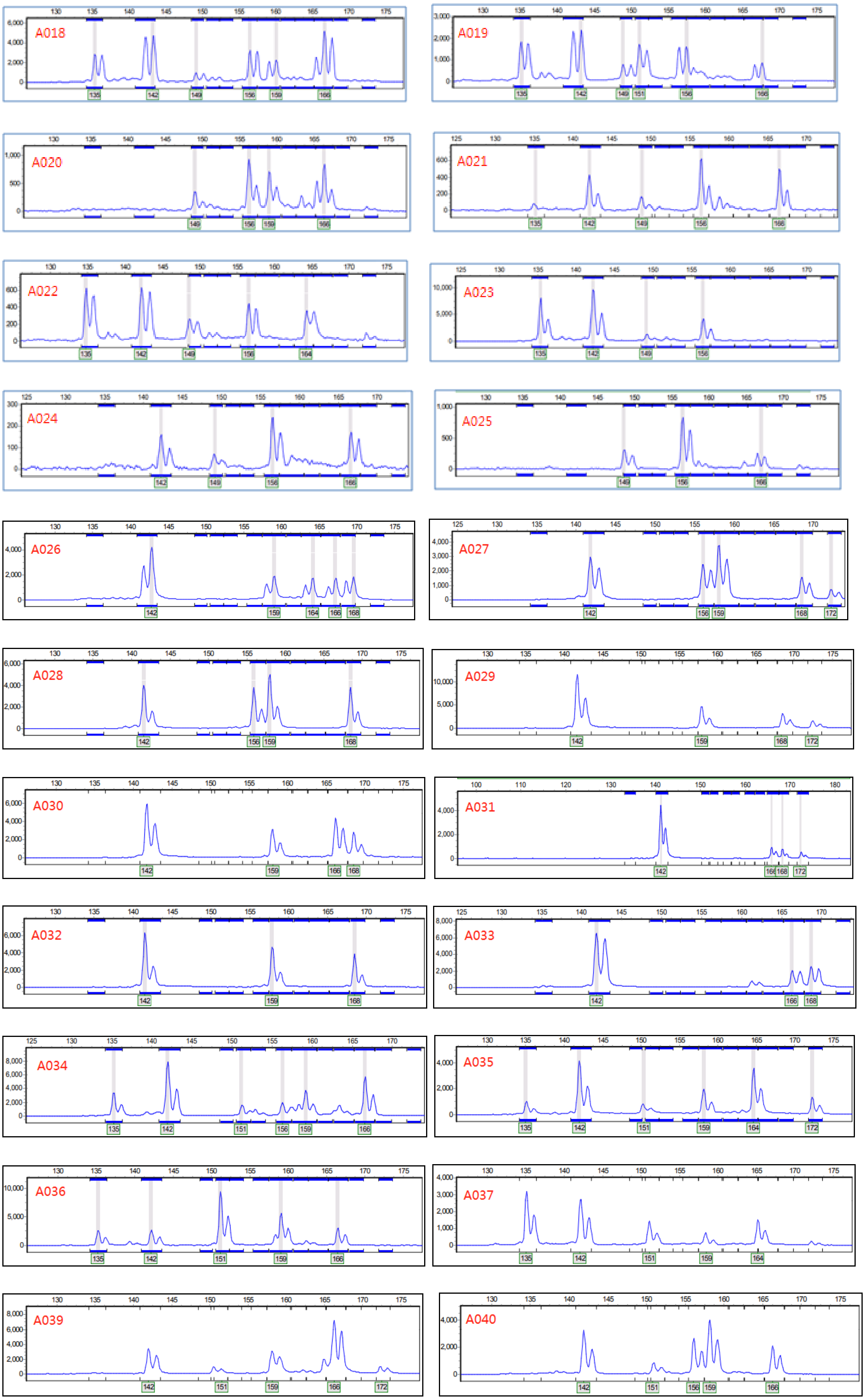
A018
135 142 149 156 159 166
A019
135 142 149 151 156 166
A020
149 156 159 166
A021
135 142 149 156 166
A022
135 142 149 156 164
A023
135 142 149 156
A024
142 149 156 166
A025
149 156 166
A026
142 159 164 166 168
A027
142 156 159 168 172
A028
142 156 159 168
A029
142 159 168 172
A030
142 159 166 168
A031
142 166 168 172
A032
142 159 168
A033
142 166 168
A034
135 142 151 156 159 166
A035
135 142 151 159 164 172
A036
135 142 151 159 166
A037
135 142 151 159 164
A039
142 151 159 166 172
A040
142 151 156 159 166

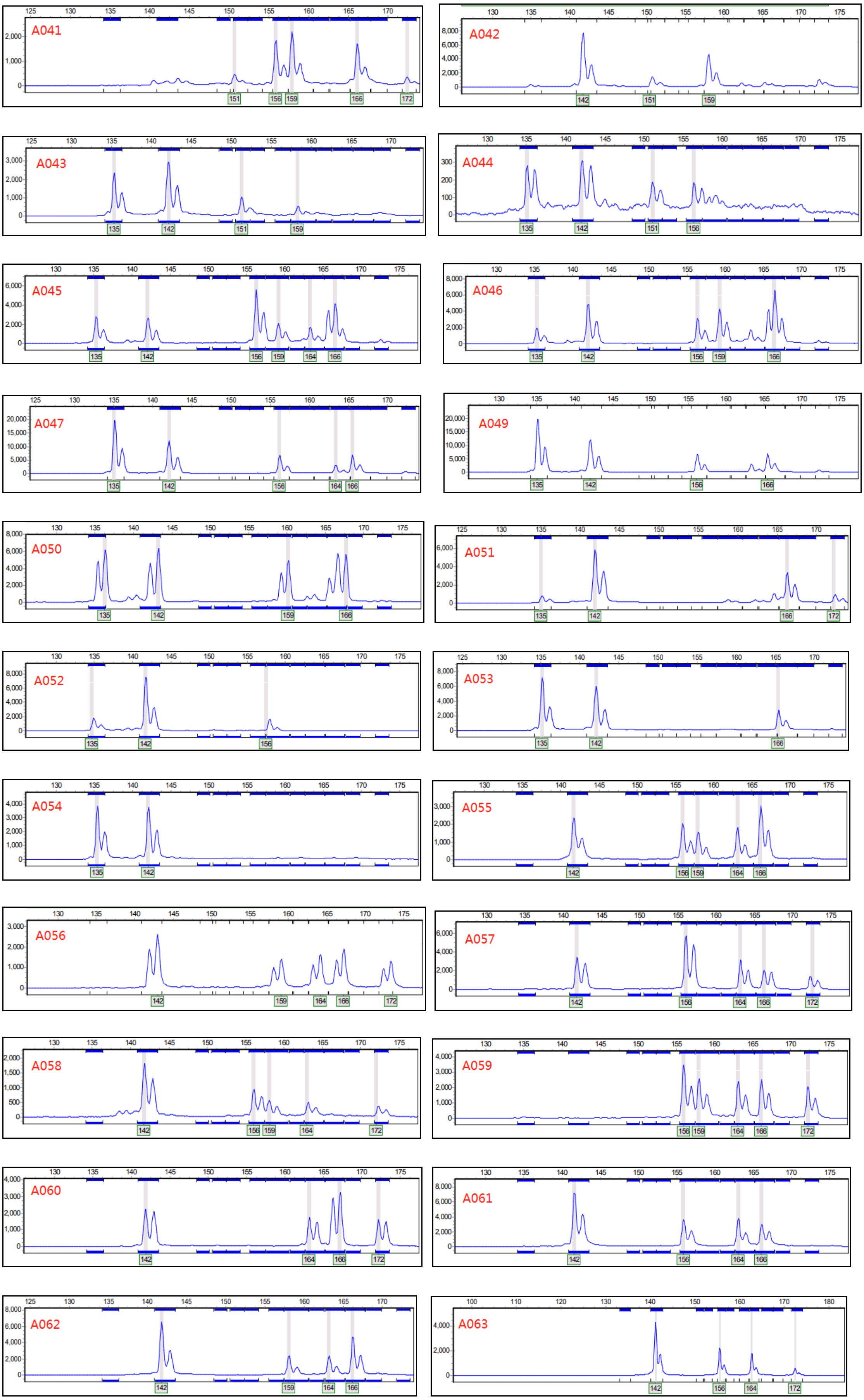
A041
151 156 159 166 172
A042
142 151 159
A043
135 142 151 159
A044
135 142 151 156
A045
135 142 156 159 164 166
A046
135 142 156 159 166
A047
135 142 156 164 166
A049
135 142 156 166
A050
135 142 159 166
A051
135 142 166 172
A052
135 142 156
A053
135 142 166
A054
135 142
A055
142 156 159 164 166
A056
142 159 164 166 172
A057
142 156 164 166 172
A058
142 156 159 164 172
A059
156 159 164 166 172
A060
142 164 166 172
A061
142 156 164 166
A062
142 159 164 166
A063
142 156 164 172

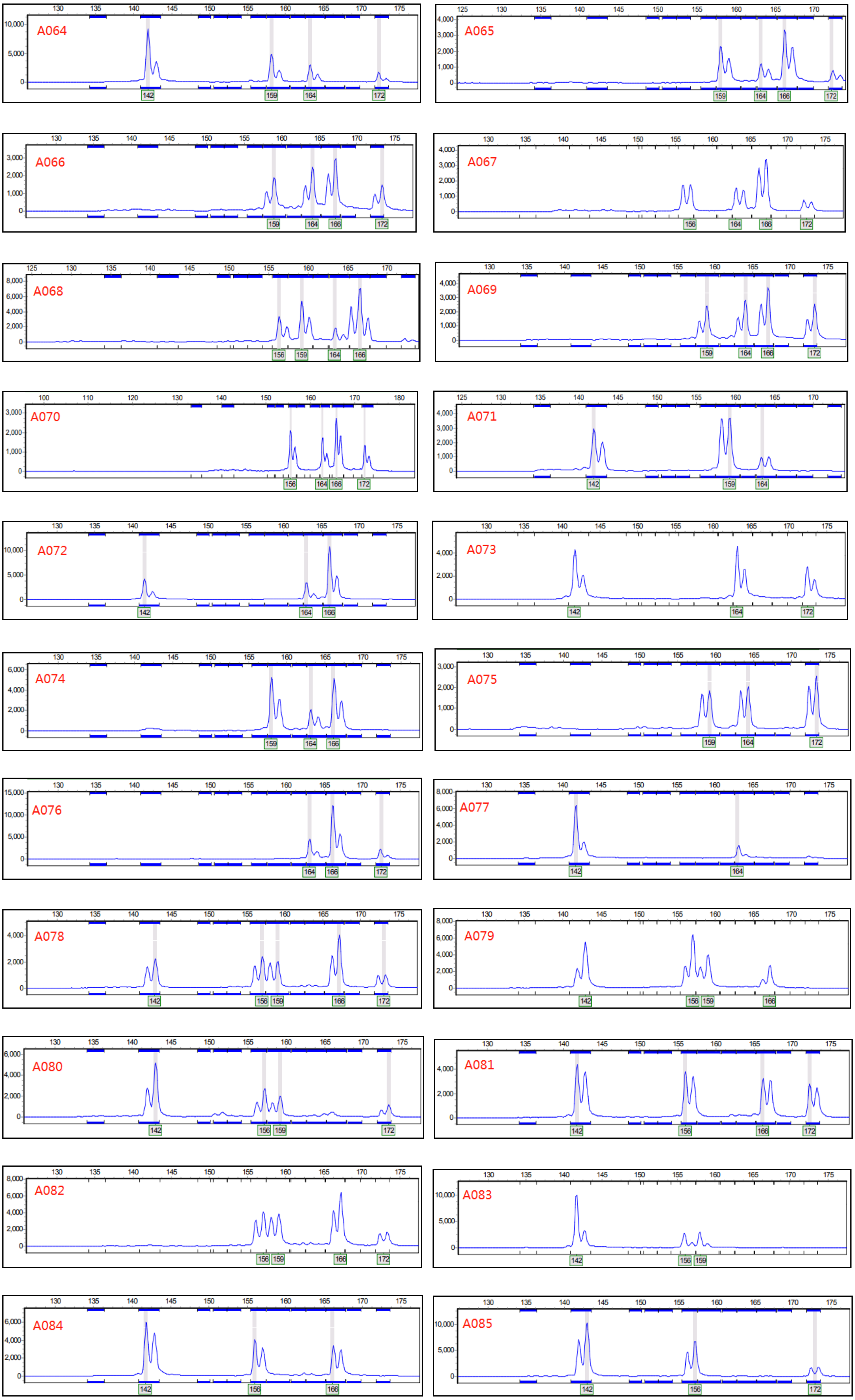
A064
142 159 164 172
A065
159 164 166 172
A066
159 164 166 172
A067
156 164 166 172
A068
156 159 164 166
A069
159 164 166 172
A070
156 164 166 172
A071
142 159 164
A072
142 164 166
A073
142 164 172
A074
159 164 166
A075
159 164 172
A076
164 166 172
A077
142 164
A078
142 156 159 166 172
A079
142 156 159 166
A080
142 156 159 172
A081
142 156 166 172
A082
156 159 166 172
A083
142 156 159
A084
142 156 166
A085
142 156 172

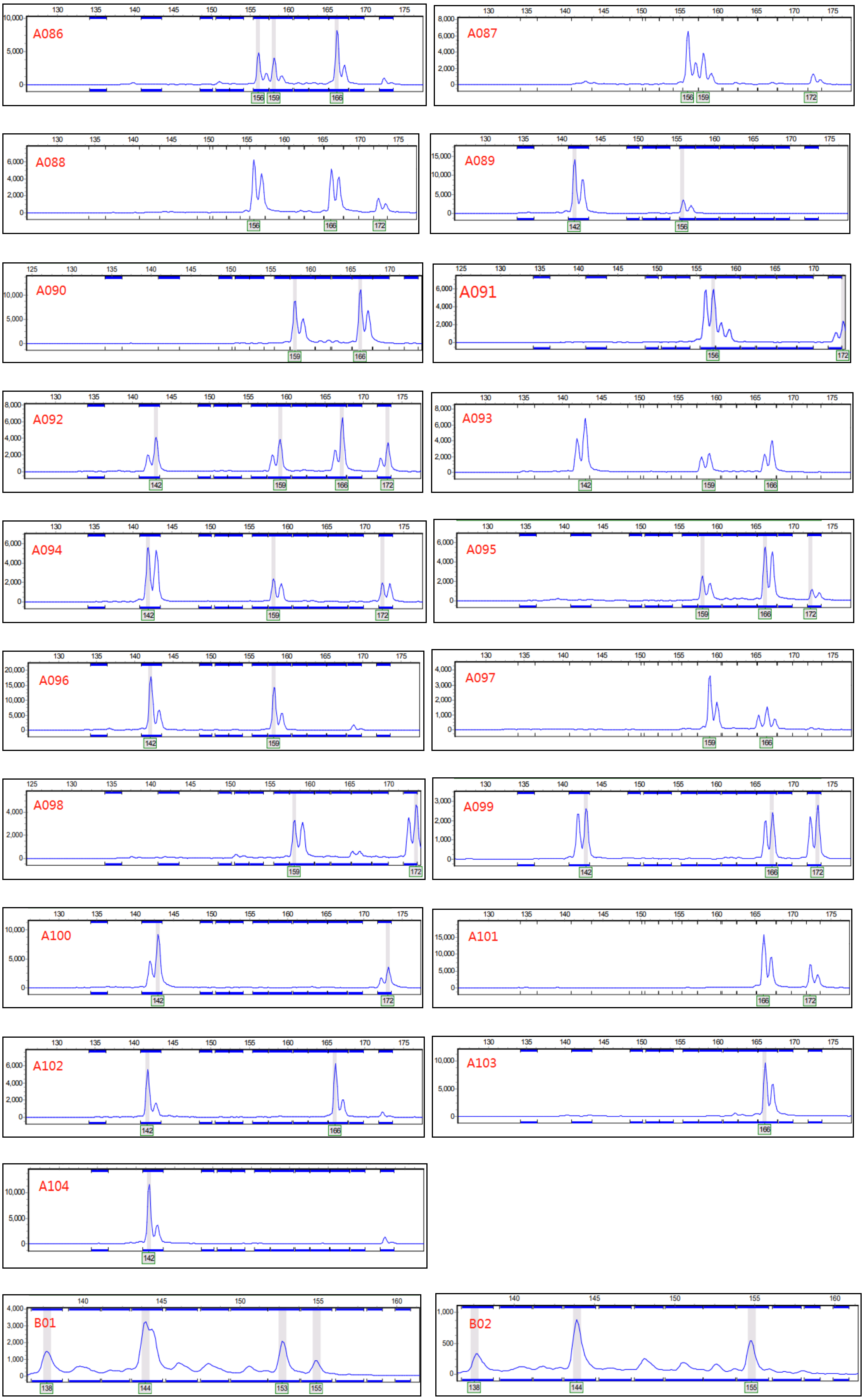
A086
156
159
166
A087
156
159
172
A088
156
166
172
A089
142
156
A090
159
166
A091
156
172
A092
142
159
166
172
A093
142
159
166
A094
142
159
172
A095
159
166
172
A096
142
159
A097
159
166
A098
159
172
A099
142
166
172
A100
142
172
A101
166
172
A102
142
166
A103
166
A104
142
B01
138
144
153
155
B02
138
144
155

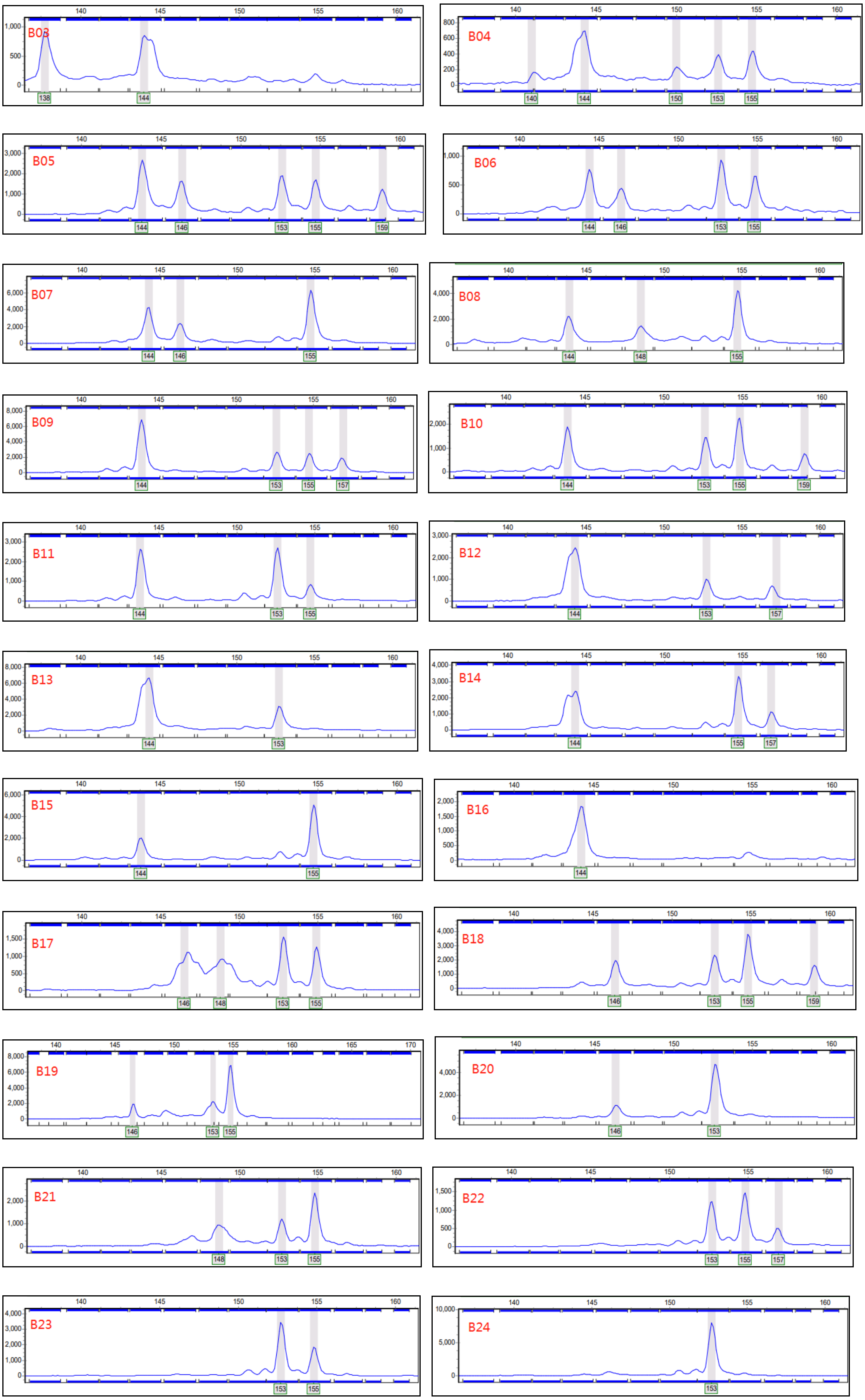
B03 138 144
B04 140 144 150 153 155
B05 144 146 153 155 159
B06 144 146 153 155
B07 144 146 155
B08 144 148 155
B09 144 153 155 157
B10 144 153 155 159
B11 144 153 155
B12 144 153 157
B13 144 153
B14 144 155 157
B15 144 155
B16 144
B17 146 148 153 155
B18 146 153 155 159
B19 146 153 155
B20 146 153
B21 148 153 155
B22 153 155 157
B23 153 155
B24 153

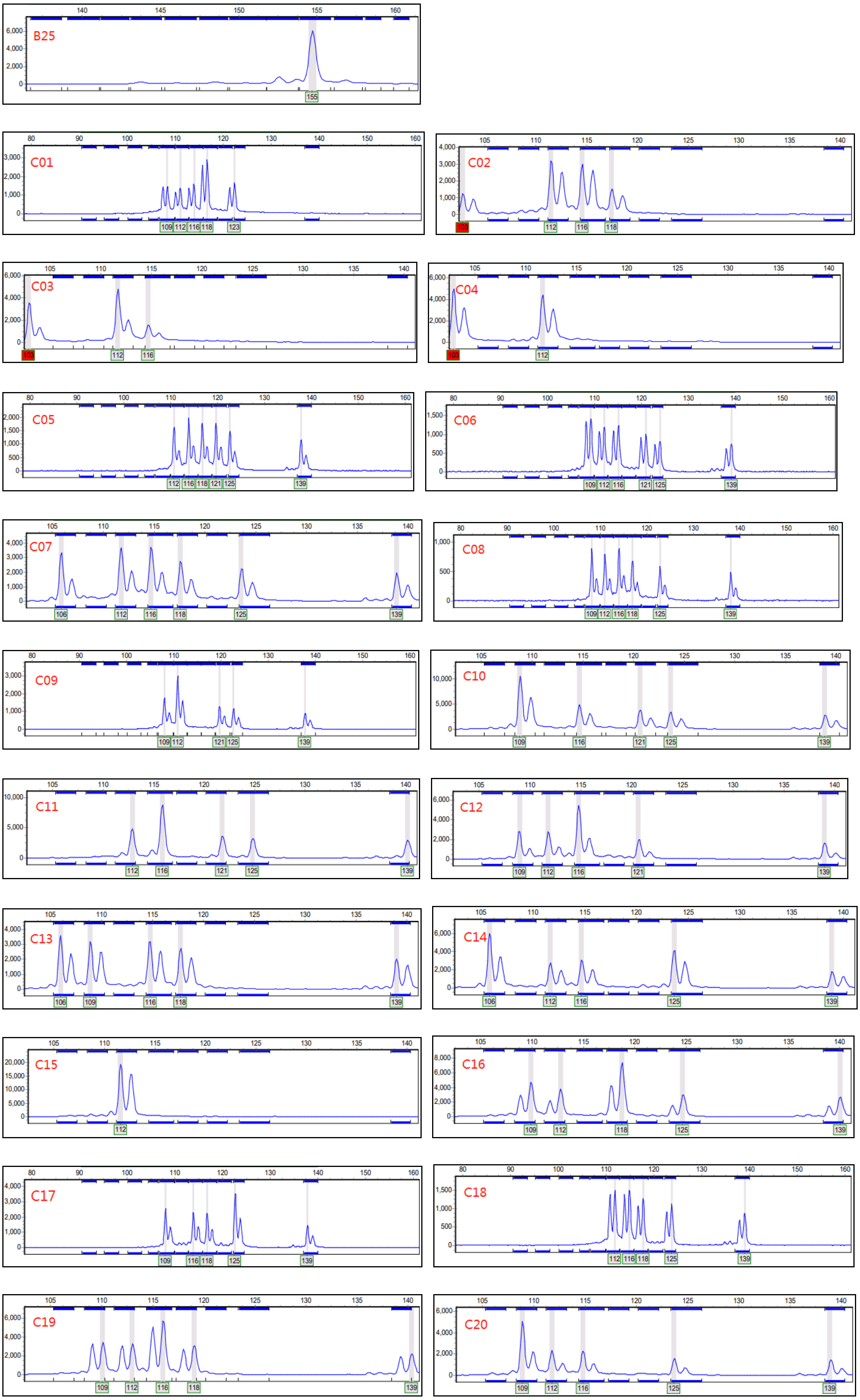
B25
155
C01
109 112 116 118 123
C02
103 112 116 118
C03
103 112 116
C04
103 112
C05
112 116 118 121 125 139
C06
109 112 116 121 125 139
C07
106 112 116 118 125 139
C08
109 112 116 118 125 139
C09
109 112 121 125 139
C10
109 116 121 125 139
C11
112 116 121 125 139
C12
109 112 116 121 139
C13
106 109 116 118 139
C14
106 112 116 125 139
C15
112
C16
109 112 118 125 139
C17
109 116 118 125 139
C18
112 116 118 125 139
C19
109 112 116 118 139
C20
109 112 116 125 139

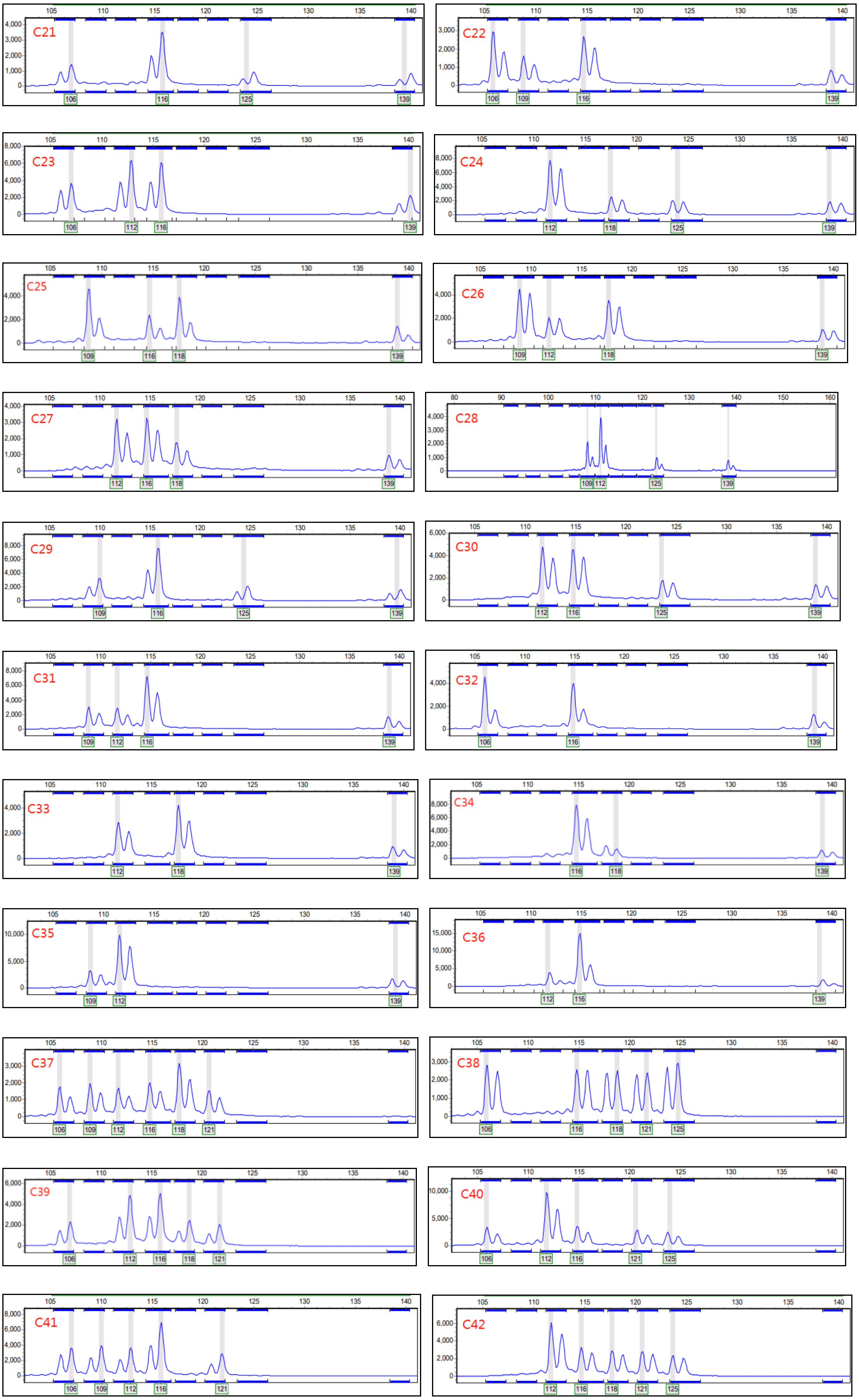
C21 106 116 125 139
C22 106 109 116 139
C23 106 112 116 139
C24 112 118 125 139
C25 109 116 118 139
C26 109 112 118 139
C27 112 116 118 139
C28 109 112 125 139
C29 109 116 125 139
C30 112 116 125 139
C31 109 112 116 139
C32 106 116 139
C33 112 118 139
C34 116 118 139
C35 109 112 139
C36 112 116 139
C37 106 109 112 116 118 121
C38 106 116 118 121 125
C39 106 112 116 118 121
C40 106 112 116 121 125
C41 106 109 112 116 121
C42 112 116 118 121 125

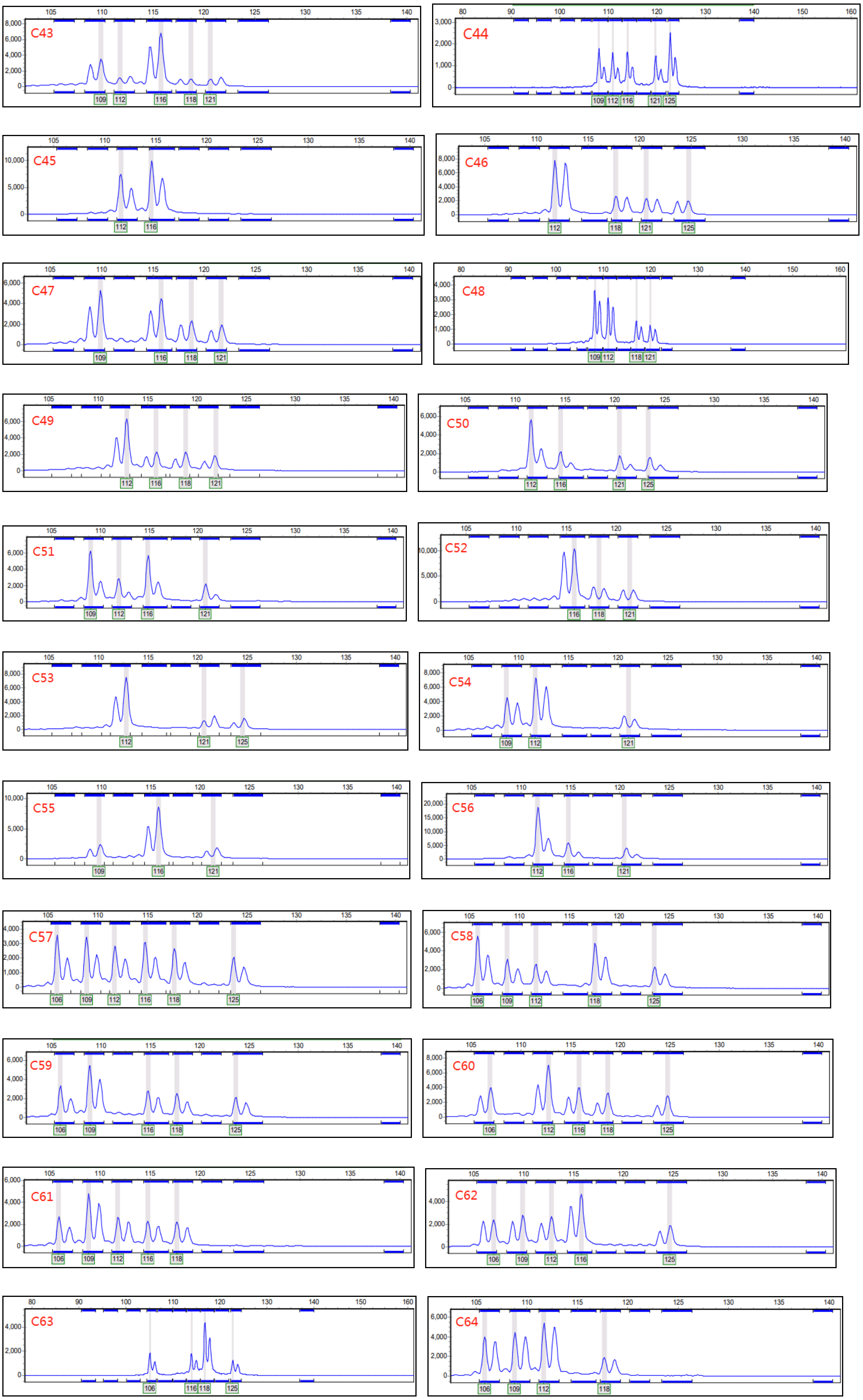

C43
109 112 116 118 121
C44
109 112 116 121 125
C45
112 116
C46
112 118 121 125
C47
109 116 118 121
C48
109 112 118 121
C49
112 116 118 121
C50
112 116 121 125
C51
109 112 116 121
C52
116 118 121
C53
112 121 125
C54
109 112 121
C55
109 116 121
C56
112 116 121
C57
106 109 112 116 118 125
C58
106 109 112 118 125
C59
106 109 116 118 125
C60
106 112 116 118 125
C61
106 109 112 116 118
C62
106 109 112 116 125
C63
106 116 118 125
C64
106 109 112 118

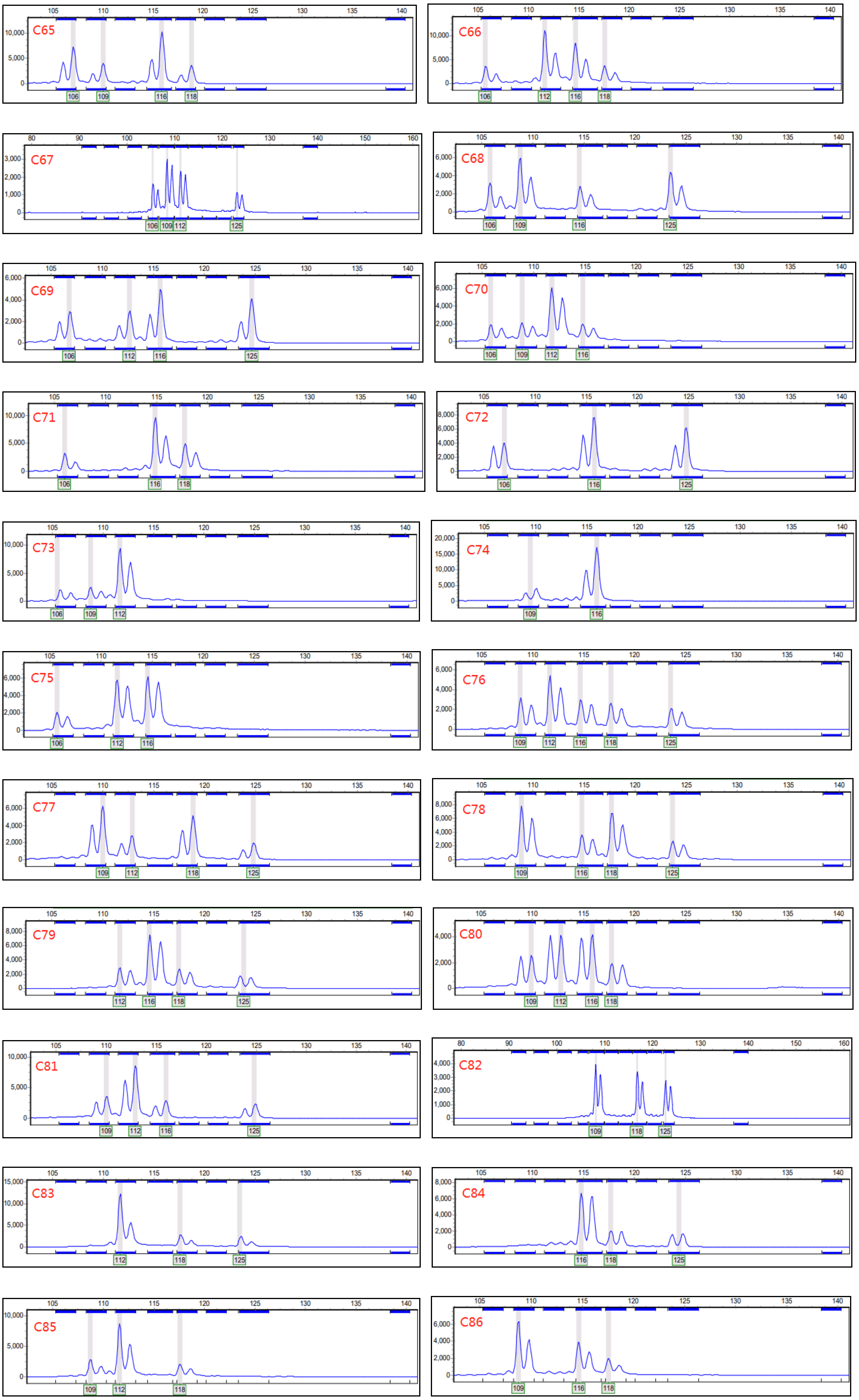
C65
106 109 116 118
C66
106 112 116 118
C67
106 109 112 125
C68
106 109 116 125
C69
106 112 116 125
C70
106 109 112 116
C71
106 116 118
C72
106 116 125
C73
106 109 112
C74
109 116
C75
106 112 116
C76
109 112 116 118 125
C77
109 112 118 125
C78
109 116 118 125
C79
112 116 118 125
C80
109 112 116 118
C81
109 112 116 125
C82
109 118 125
C83
112 118 125
C84
116 118 125
C85
109 112 118
C86
109 116 118

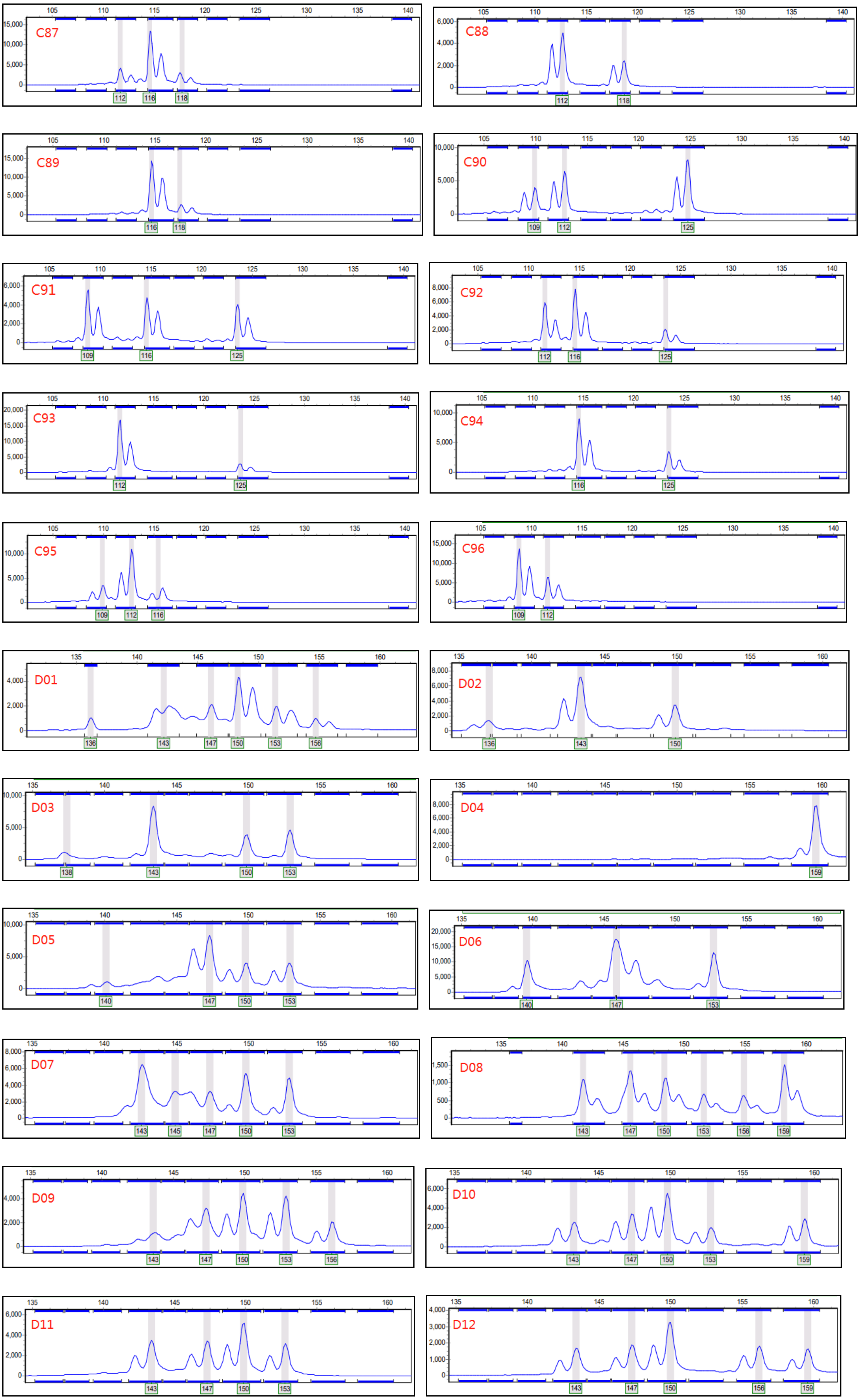
C87
C88
C89
C90
C91
C92
C93
C94
C95
C96
D01
D02
D03
D04
D05
D06
D07
D08
D09
D10
D11
D12

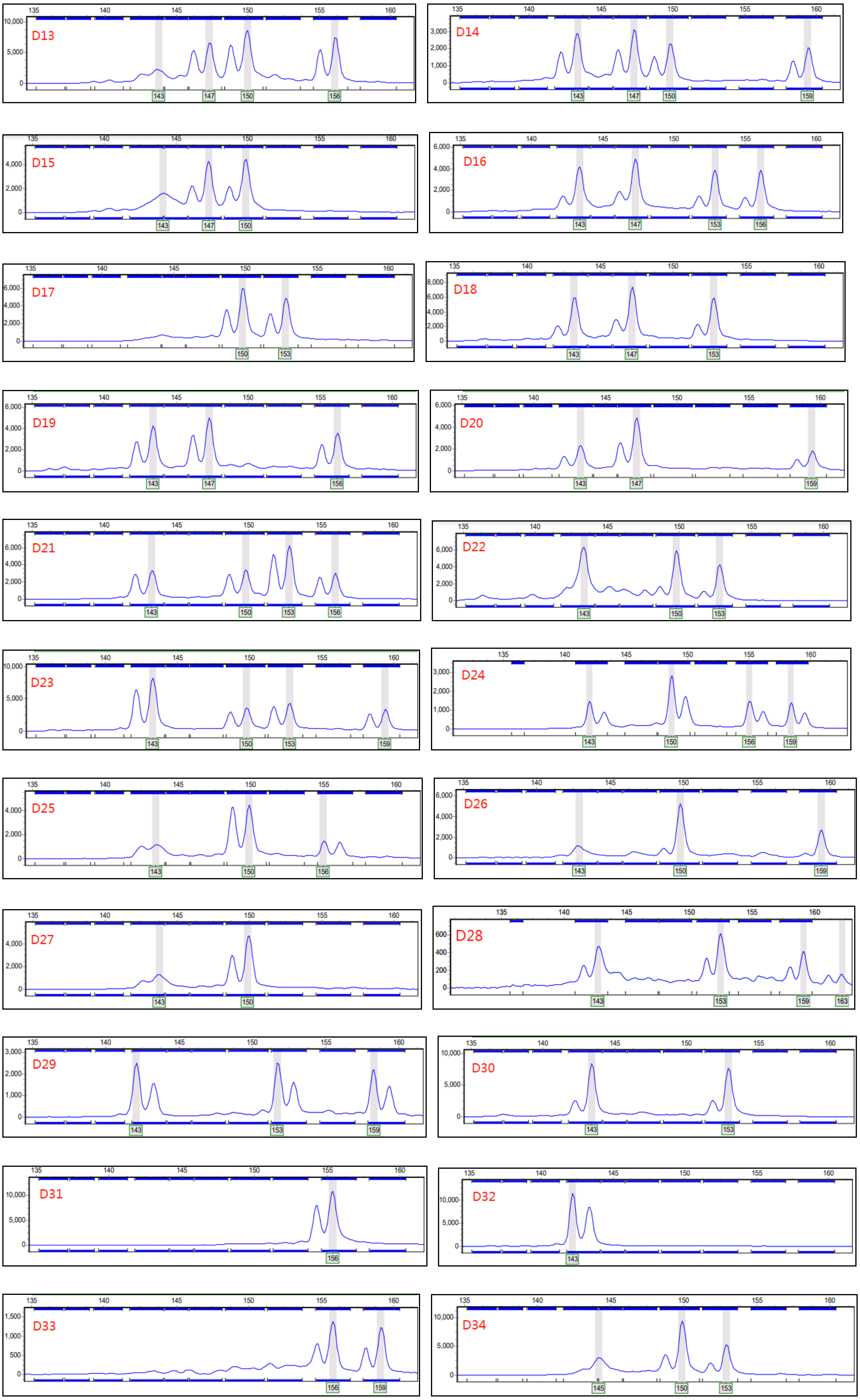
D13
143 147 150 156
D14
143 147 150 159
D15
143 147 150
D16
143 147 153 156
D17
150 153
D18
143 147 153
D19
143 147 156
D20
143 147 159
D21
143 150 153 156
D22
143 150 153
D23
143 150 153 159
D24
143 150 156 159
D25
143 150 156
D26
143 150 159
D27
143 150
D28
143 153 159 163
D29
143 153 159
D30
143 153
D31
156
D32
143
D33
156 159
D34
145 150 153

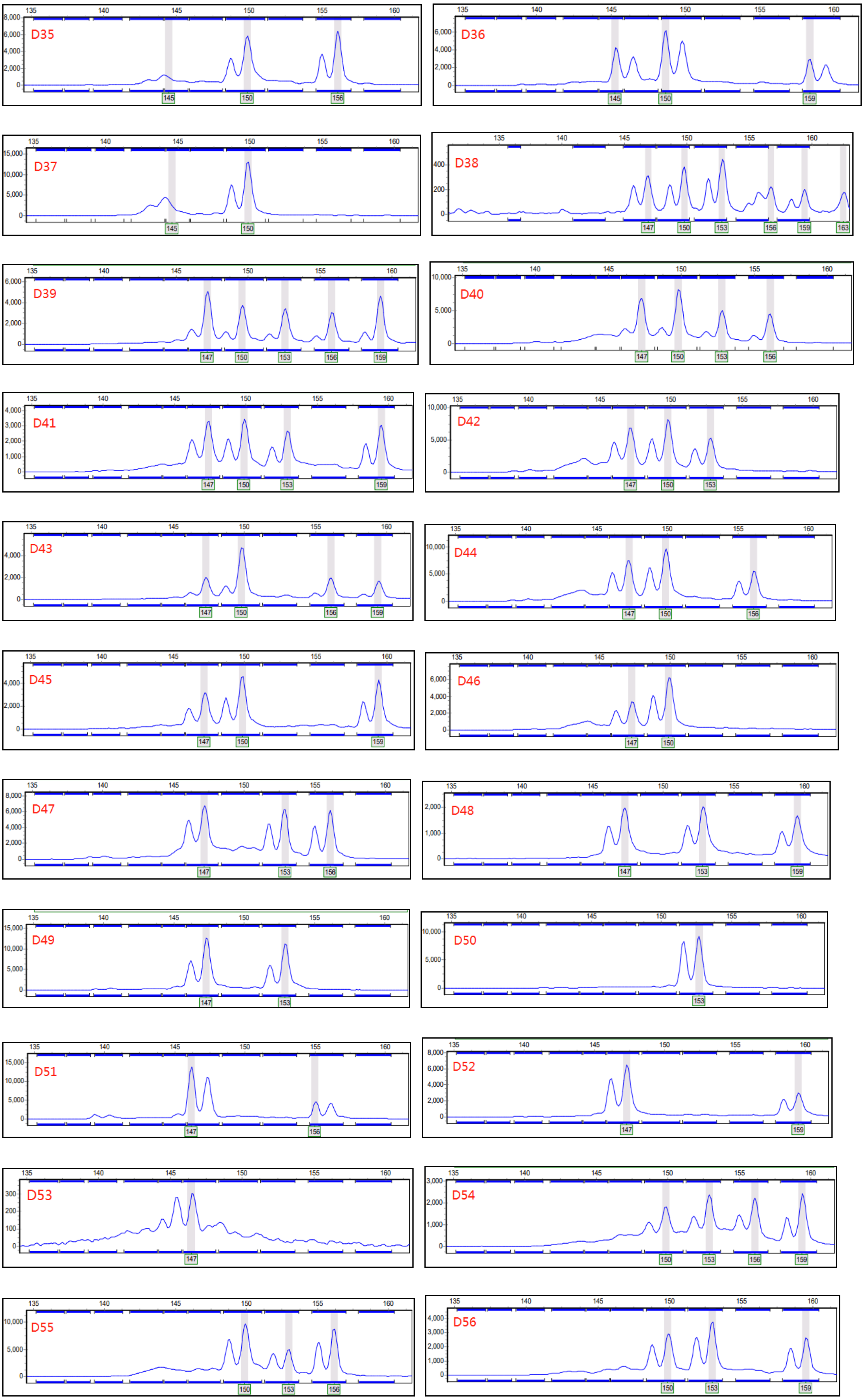
D35 145 150 156
D36 145 150 159
D37 145 150
D38 147 150 153 156 159 163
D39 147 150 153 156 159
D40 147 150 153 156
D41 147 150 153 159
D42 147 150 153
D43 147 150 156 159
D44 147 150 156
D45 147 150 159
D46 147 150
D47 147 153 156
D48 147 153 159
D49 147 153
D50 153
D51 147 156
D52 147 159
D53 147
D54 150 153 156 159
D55 150 153 156
D56 150 153 159

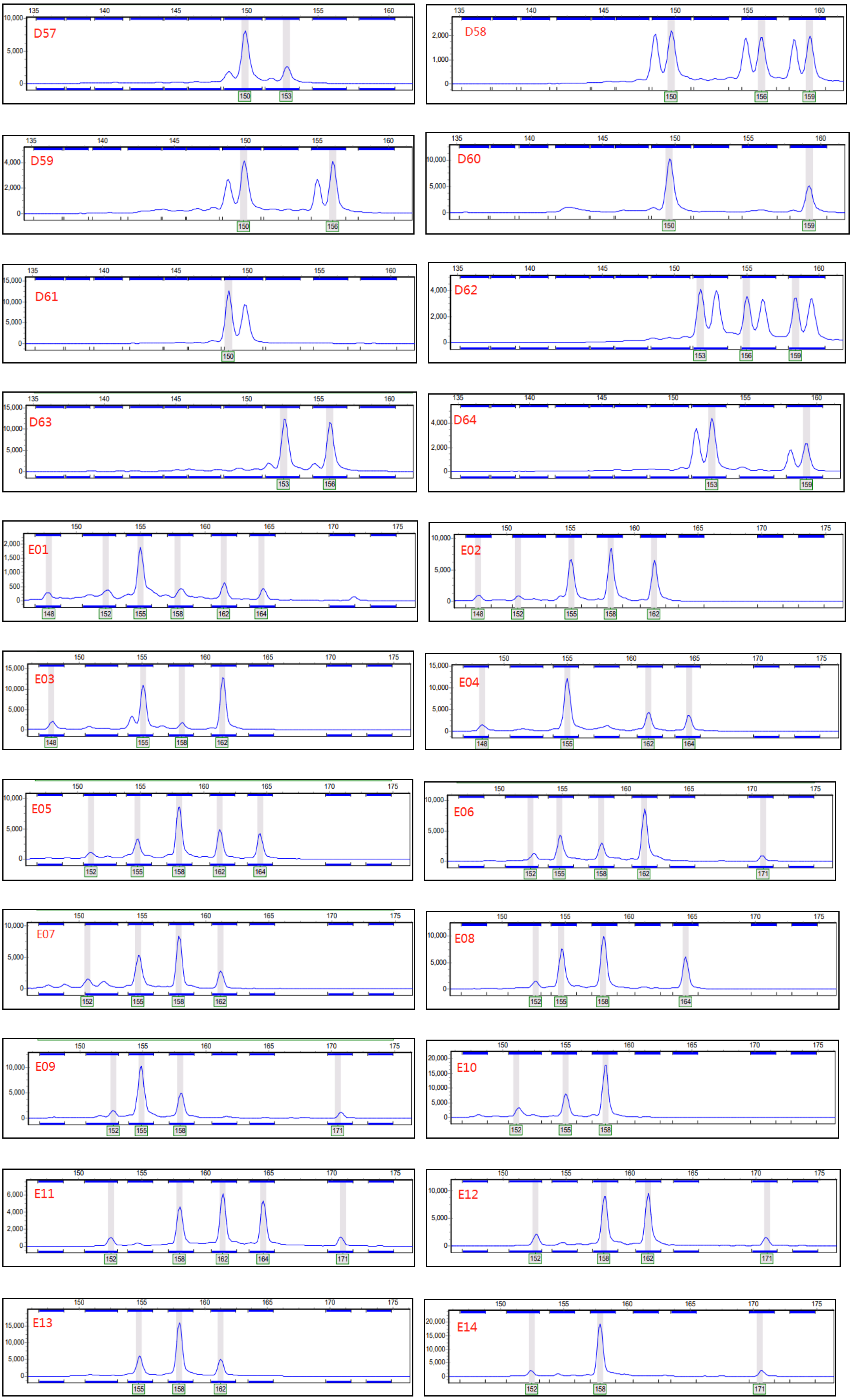
D57
150
153
D58
150
156
159
D59
150
156
D60
150
159
D61
150
D62
153
156
159
D63
153
156
D64
153
159
E01
148
152
155
158
162
164
E02
148
152
155
158
162
E03
148
155
158
162
E04
148
155
162
164
E05
152
155
158
162
164
E06
152
155
158
162
171
E07
152
155
158
162
E08
152
155
158
164
E09
152
155
158
171
E10
152
155
158
E11
152
158
162
164
171
E12
152
158
162
171
E13
155
158
162
E14
152
158
171

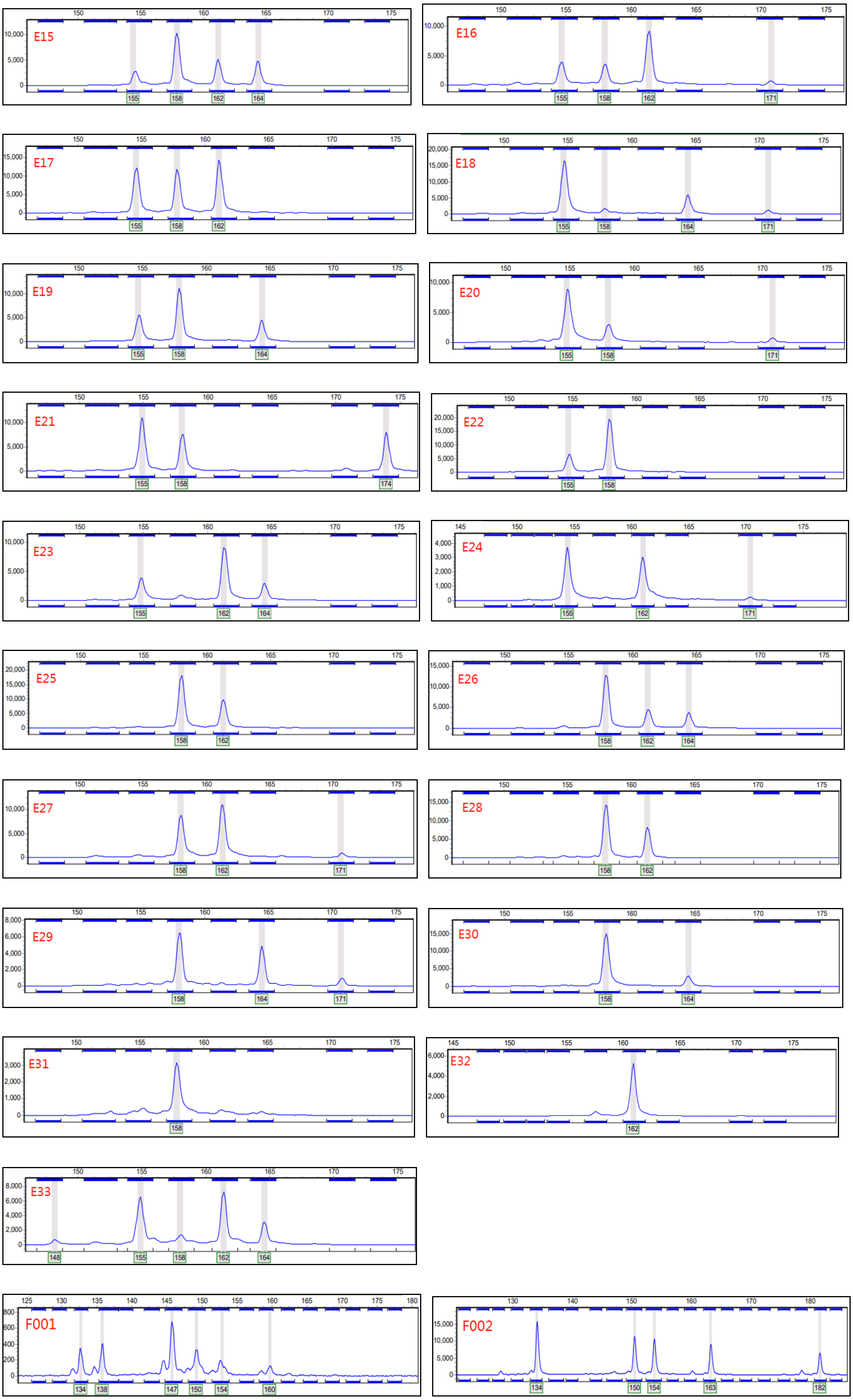

E15
155 158 162 164
E16
155 158 162 171
E17
155 158 162
E18
155 158 164 171
E19
155 158 164
E20
155 158 171
E21
155 158 174
E22
155 158
E23
155 162 164
E24
155 162 171
E25
158 162
E26
158 162 164
E27
158 162 171
E28
158 162
E29
158 164 171
E30
158 164
E31
158
E32
162
E33
148 155 158 162 164
F001
134 138 147 150 154 160
F002
134 150 154 163 182

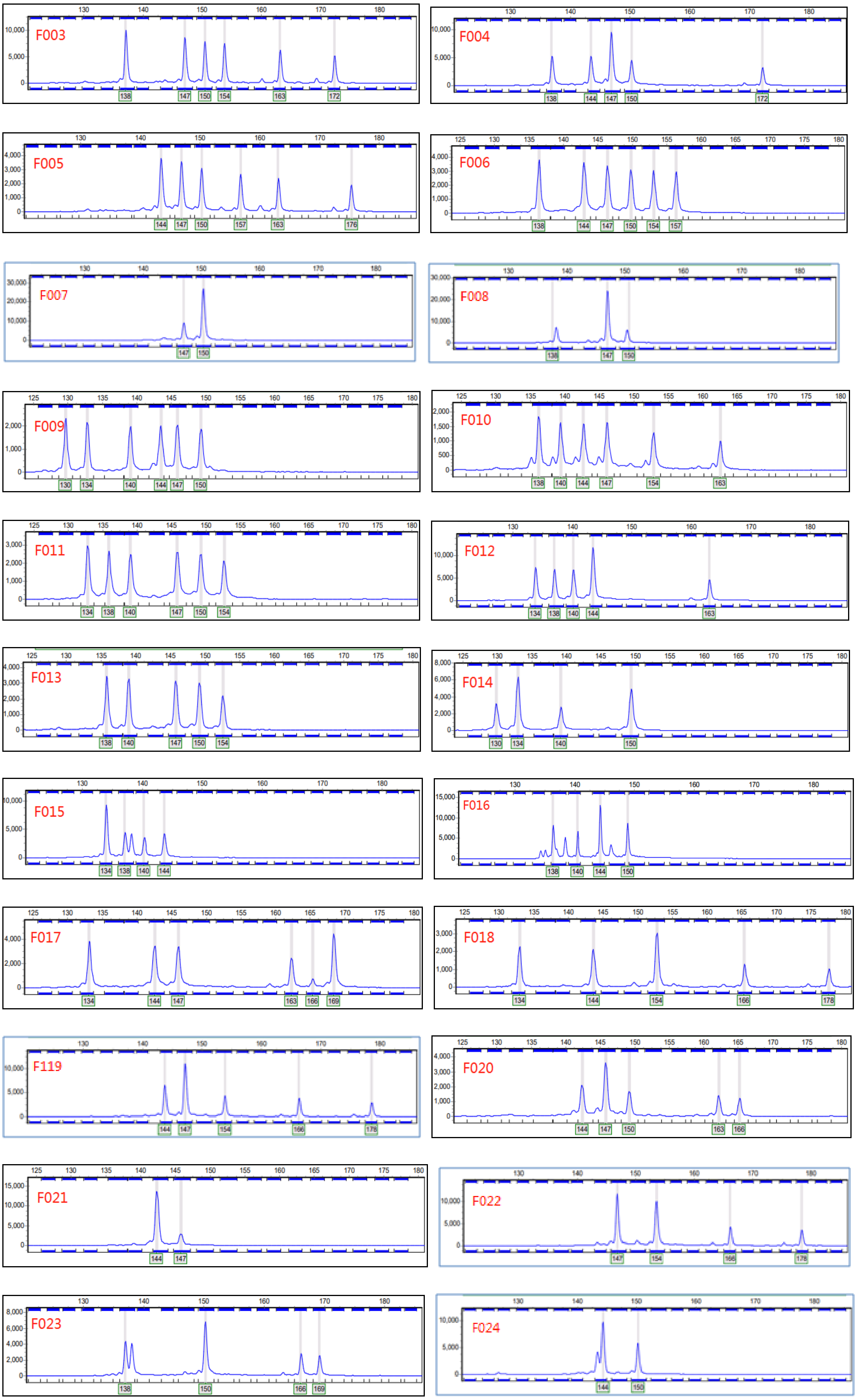
F003 138 147 150 154 163 172
F004 138 144 147 150 172
F005 144 147 150 157 163 176
F006 138 144 147 150 154 157
F007 147 150
F008 138 147 150
F009 130 134 140 144 147 150
F010 138 140 144 147 154 163
F011 134 138 140 147 150 154
F012 134 138 140 144 163
F013 138 140 147 150 154
F014 130 134 140 150
F015 134 138 140 144
F016 138 140 144 150
F017 134 144 147 163 166 169
F018 134 144 154 166 178
F119 144 147 154 166 178
F020 144 147 150 163 166
F021 144 147
F022 147 154 166 178
F023 138 150 166 169
F024 144 150

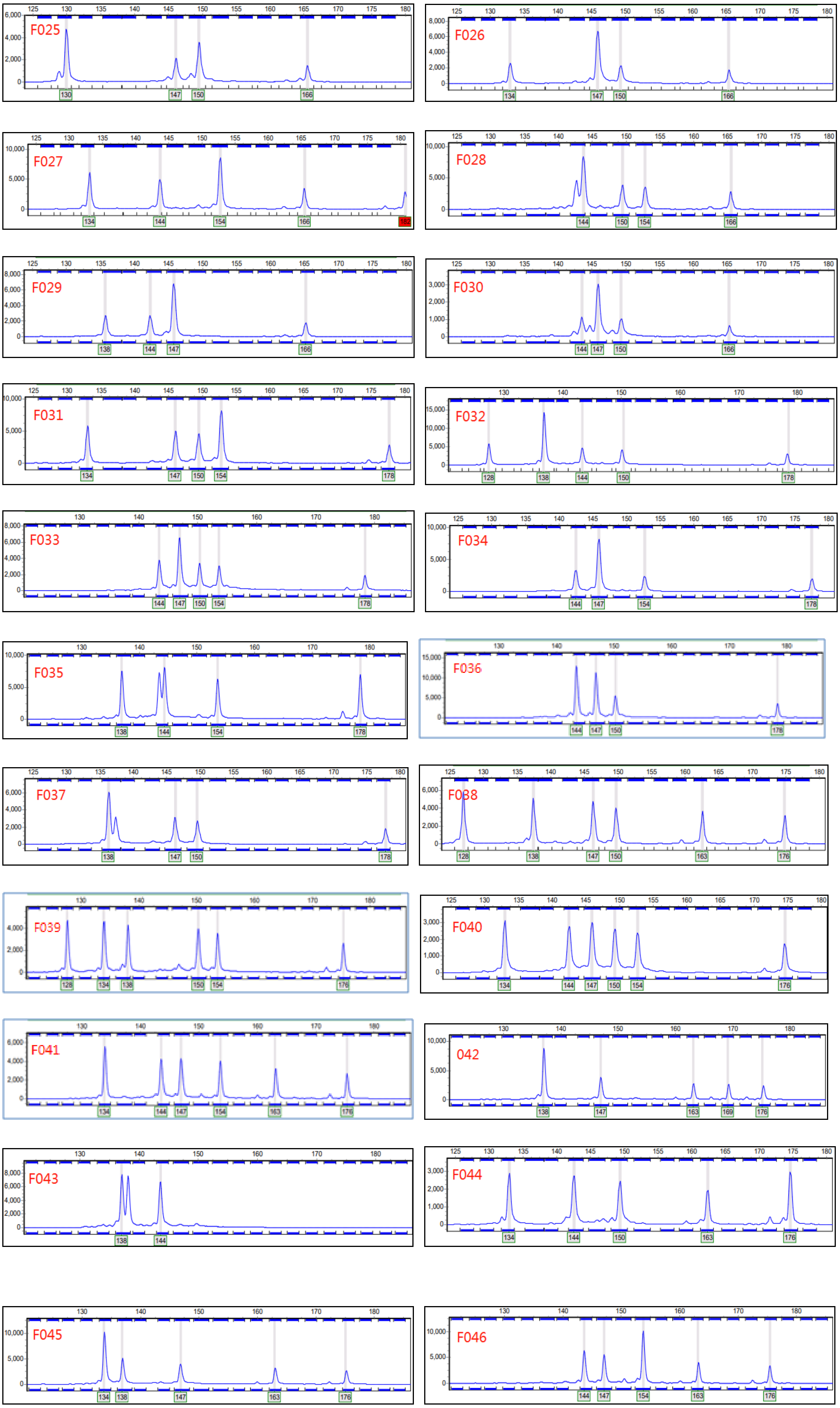

F025
130
147
150
166
F026
134
147
150
166
F027
134
144
154
166
F028
144
150
154
166
F029
138
144
147
166
F030
144
147
150
166
F031
134
147
150
154
178
F032
128
138
144
150
178
F033
144
147
150
154
178
F034
144
147
154
178
F035
138
144
154
178
F036
144
147
150
178
F037
138
147
150
178
F038
128
138
147
150
163
176
F039
128
134
138
150
154
176
F040
134
144
147
150
154
176
F041
134
144
147
154
163
176
042
138
147
163
169
176
F043
138
144
F044
134
144
150
163
176
F045
134
138
147
163
176
F046
144
147
154
163
176

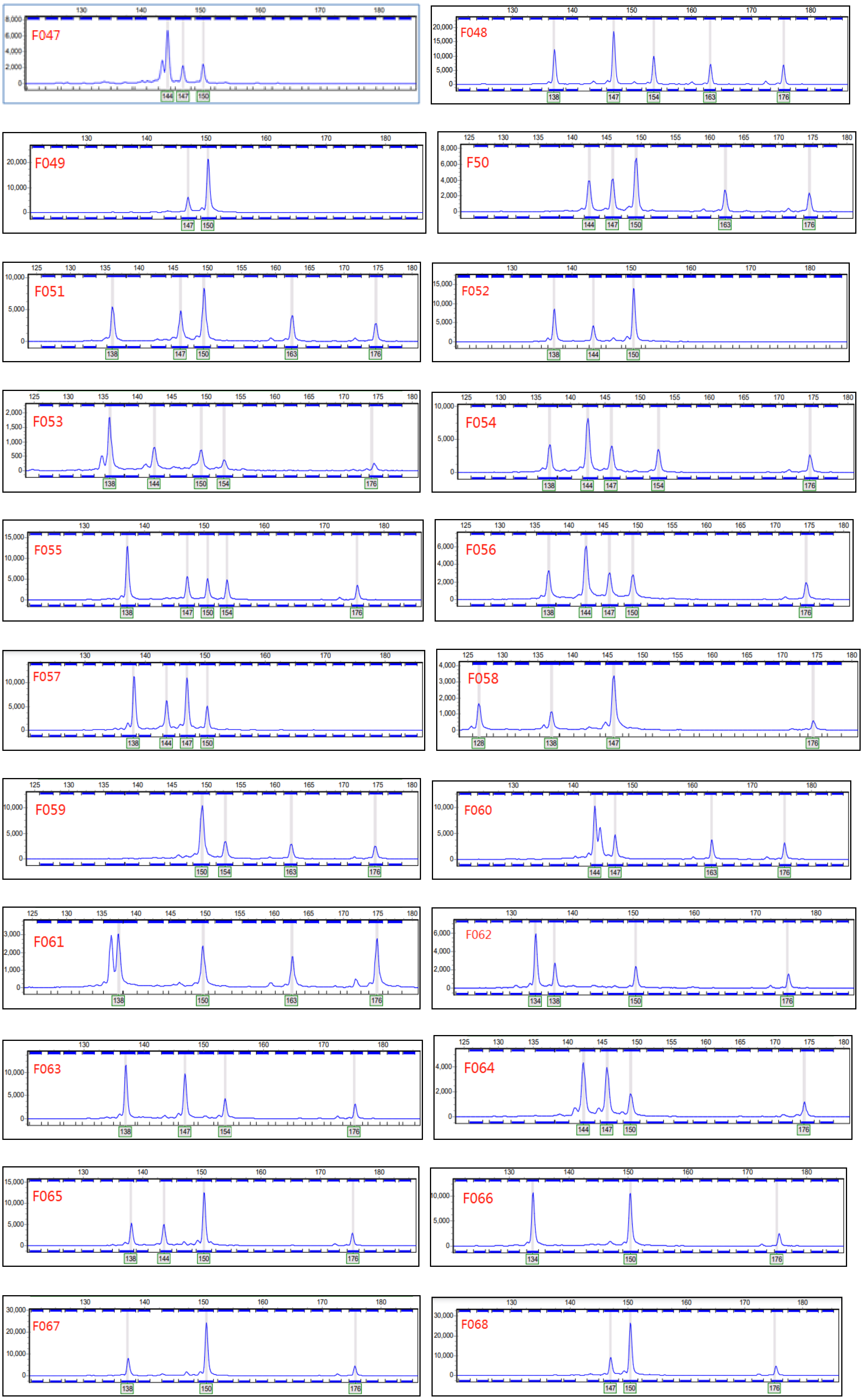

F047
144 147 150
F048
138 147 154 163 176
F049
147 150
F50
144 147 150 163 176
F051
138 147 150 163 176
F052
138 144 150
F053
138 144 150 154 176
F054
138 144 147 154 176
F055
138 147 150 154 176
F056
138 144 147 150 176
F057
138 144 147 150
F058
128 138 147 176
F059
150 154 163 176
F060
144 147 163 176
F061
138 150 163 176
F062
134 138 150 176
F063
138 147 154 176
F064
144 147 150 176
F065
138 144 150 176
F066
134 150 176
F067
138 150 176
F068
147 150 176

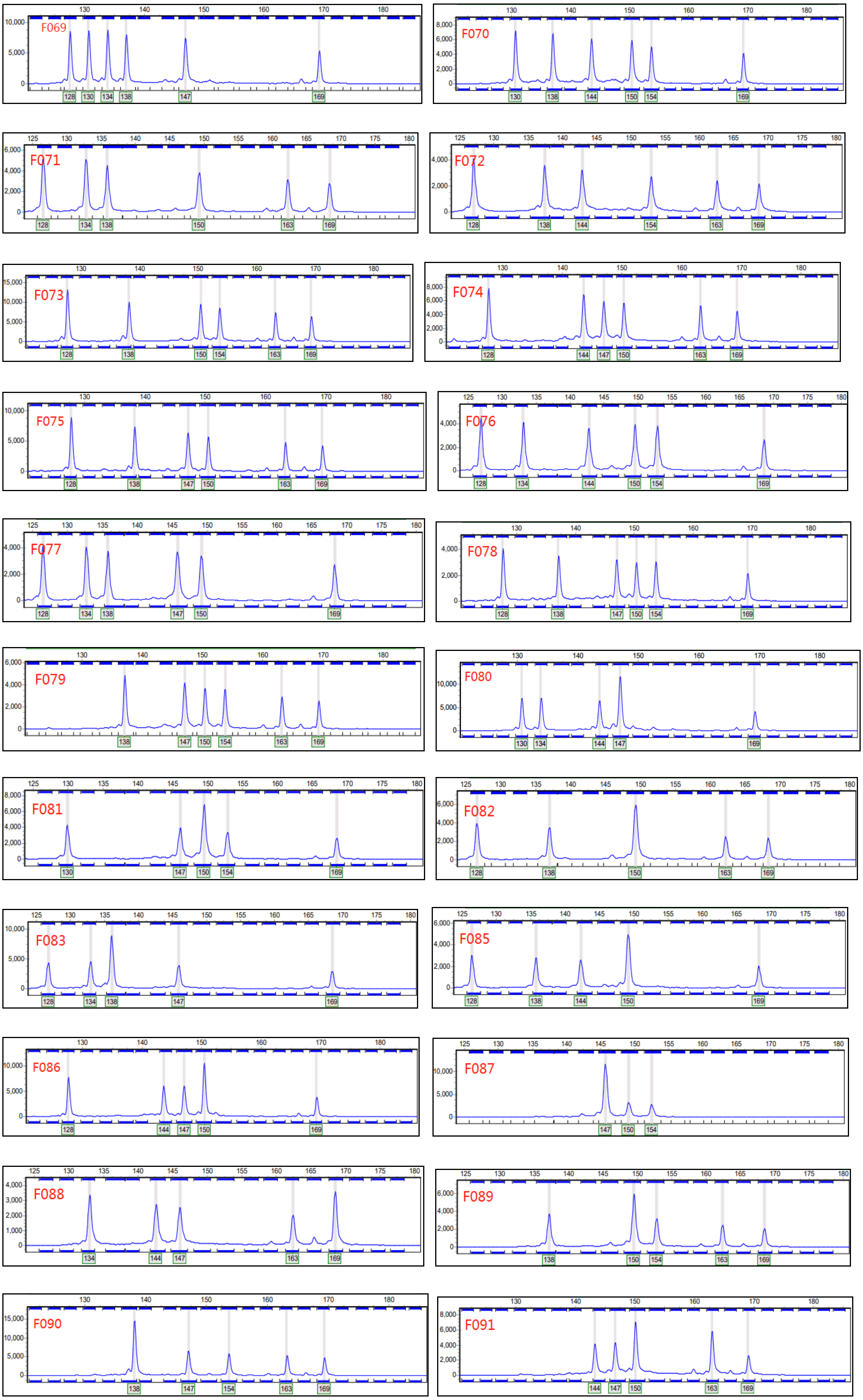

F069
128 130 134 138 147 169
F070
130 138 144 150 154 169
F071
128 134 138 150 163 169
F072
128 138 144 154 163 169
F073
128 138 150 154 163 169
F074
128 144 147 150 163 169
F075
128 138 147 150 163 169
F076
128 134 144 150 154 169
F077
128 134 138 147 150 169
F078
128 138 147 150 154 169
F079
138 147 150 154 163 169
F080
130 134 144 147 169
F081
130 147 150 154 169
F082
128 138 150 163 169
F083
128 134 138 147 169
F085
128 138 144 150 169
F086
128 144 147 150 169
F087
147 150 154
F088
134 144 147 163 169
F089
138 150 154 163 169
F090
138 147 154 163 169
F091
144 147 150 163 169

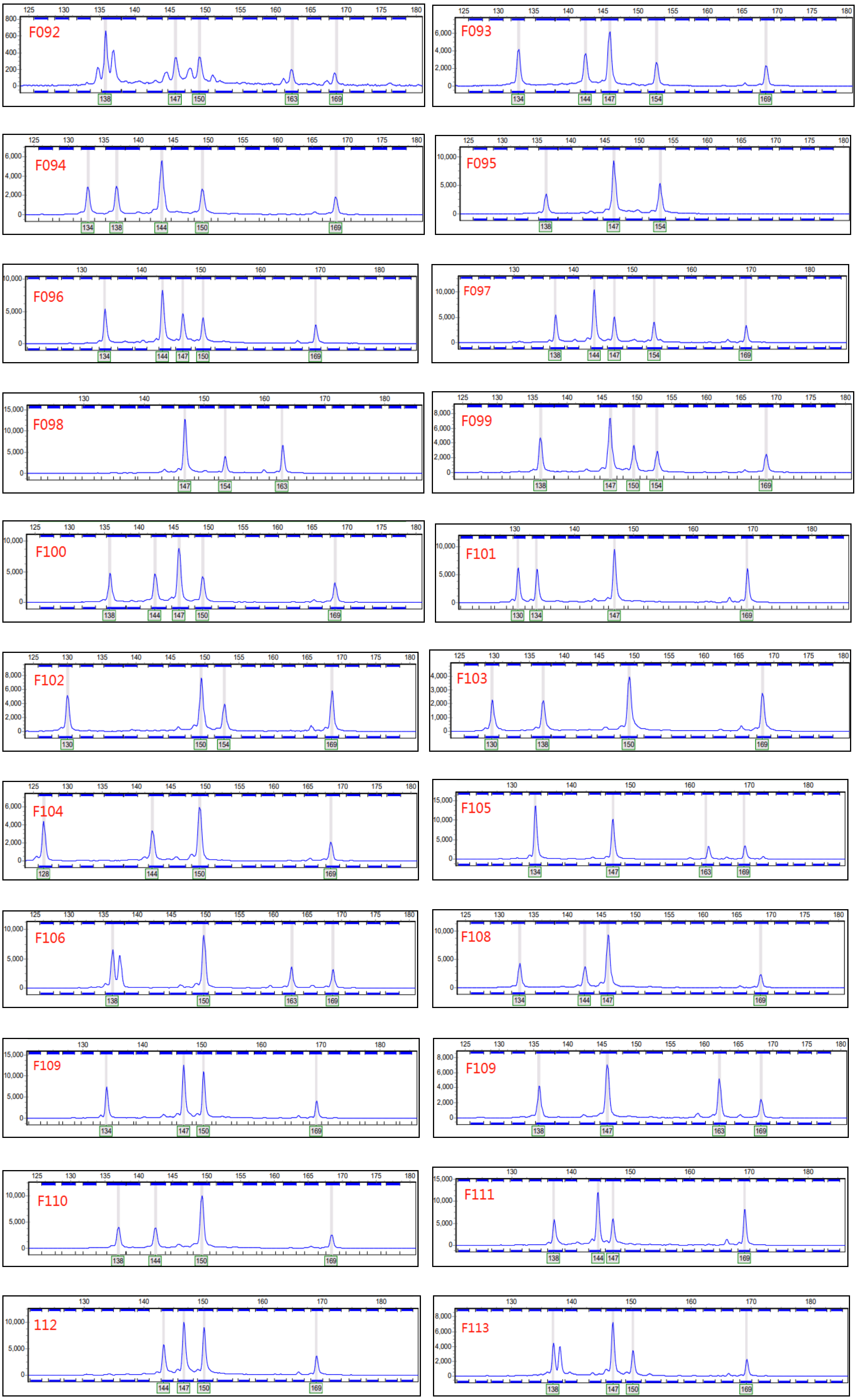
F092
138 147 150 163 169
F093
134 144 147 154 169
F094
134 138 144 150 169
F095
138 147 154
F096
134 144 147 150 169
F097
138 144 147 154 169
F098
147 154 163
F099
138 147 150 154 169
F100
138 144 147 150 169
F101
130 134 147 169
F102
130 150 154 169
F103
130 138 150 169
F104
128 144 150 169
F105
134 147 163 169
F106
138 150 163 169
F108
134 144 147 169
F109
134 147 150 169
F109
138 147 163 169
F110
138 144 150 169
F111
138 144 147 169
112
144 147 150 169
F113
138 147 150 169

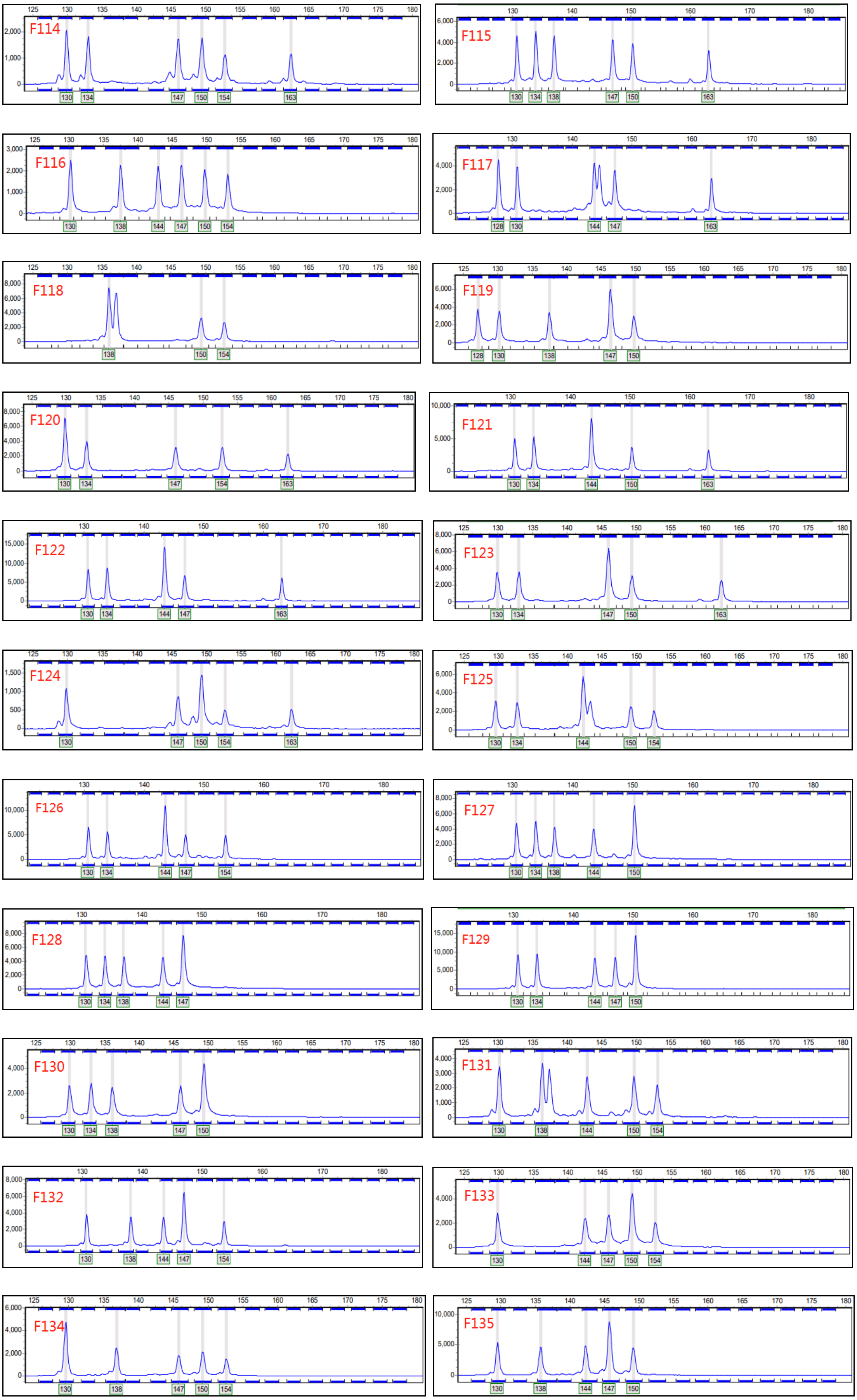

F114
130 134 147 150 154 163
F115
130 134 138 147 150 163
F116
130 138 144 147 150 154
F117
128 130 144 147 163
F118
138 150 154
F119
128 130 138 147 150
F120
130 134 147 154 163
F121
130 134 144 150 163
F122
130 134 144 147 163
F123
130 134 147 150 163
F124
130 147 150 154 163
F125
130 134 144 150 154
F126
130 134 144 147 154
F127
130 134 138 144 150
F128
130 134 138 144 147
F129
130 134 144 147 150
F130
130 134 138 147 150
F131
130 138 144 150 154
F132
130 138 144 147 154
F133
130 144 147 150 154
F134
130 138 147 150 154
F135
130 138 144 147 150

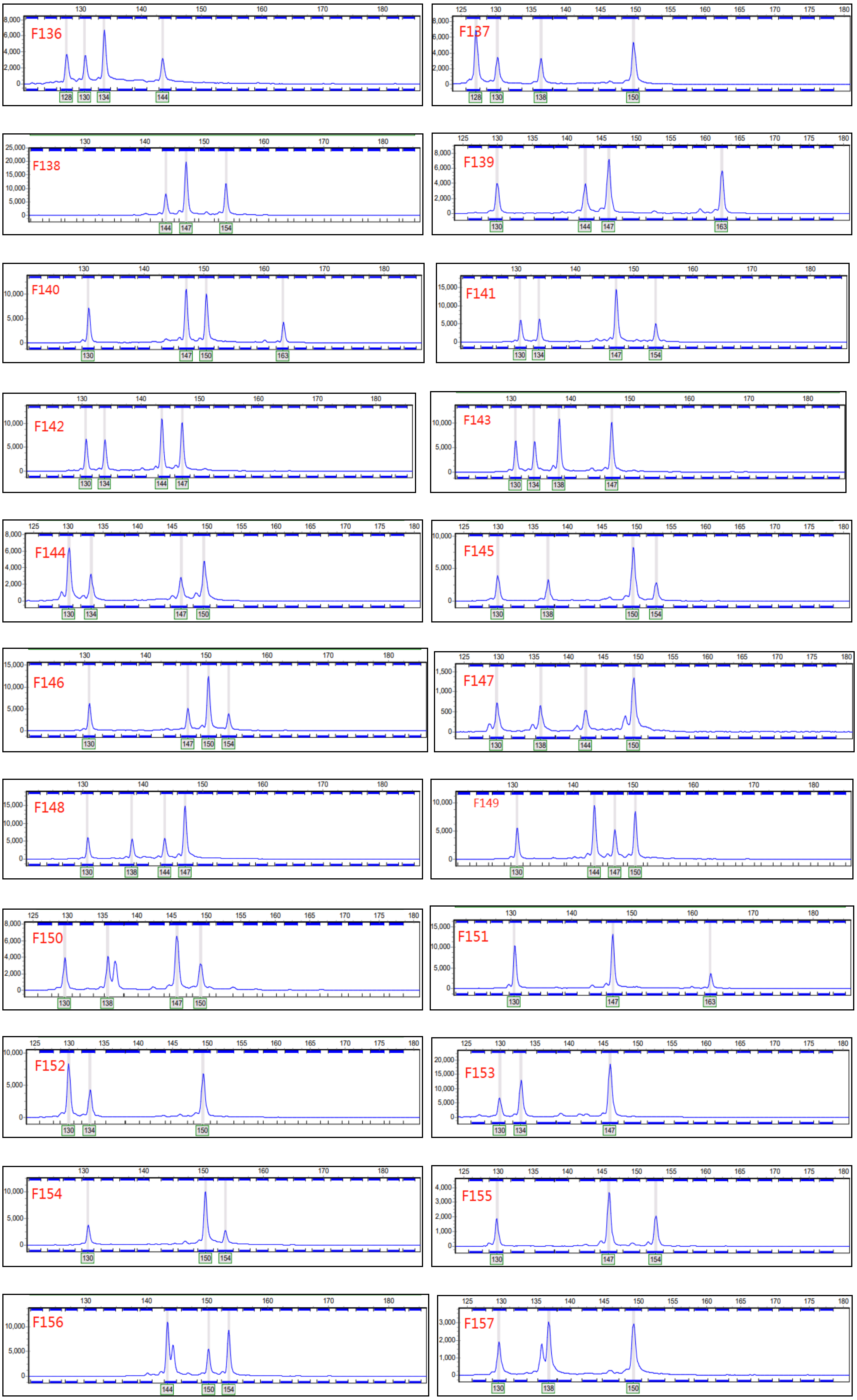
F136
128 130 134 144
F137
128 130 138 150
F138
144 147 154
F139
130 144 147 163
F140
130 147 150 163
F141
130 134 147 154
F142
130 134 144 147
F143
130 134 138 147
F144
130 134 147 150
F145
130 138 150 154
F146
130 147 150 154
F147
130 138 144 150
F148
130 138 144 147
F149
130 144 147 150
F150
130 138 147 150
F151
130 147 163
F152
130 134 150
F153
130 134 147
F154
130 150 154
F155
130 147 154
F156
144 150 154
F157
130 138 150

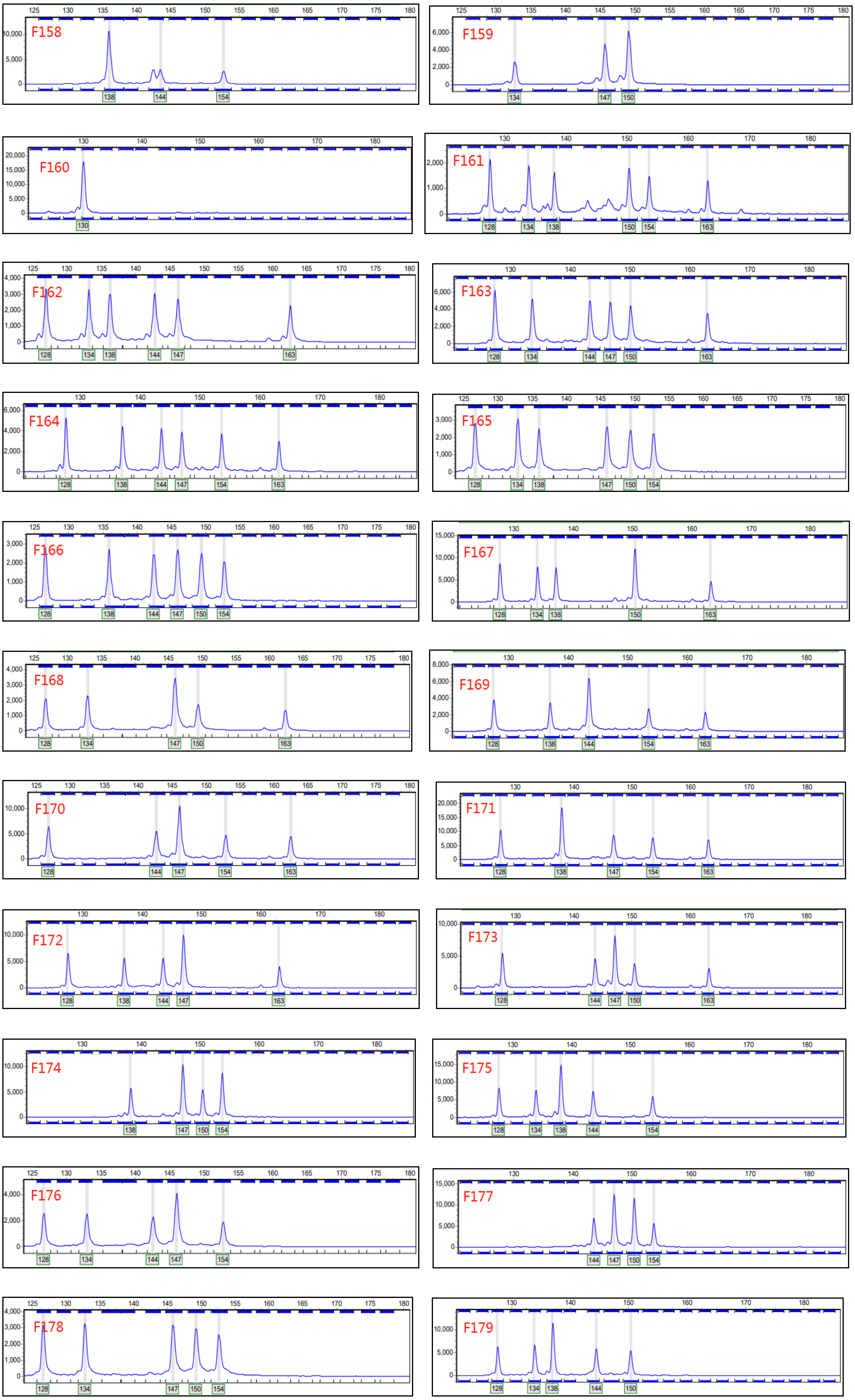
F158 138 144 154
F159 134 147 150
F160 130
F161 128 134 138 150 154 163
F162 128 134 138 144 147 163
F163 128 134 144 147 150 163
F164 128 138 144 147 154 163
F165 128 134 138 147 150 154
F166 128 138 144 147 150 154
F167 128 134 138 150 163
F168 128 134 147 150 163
F169 128 138 144 154 163
F170 128 144 147 154 163
F171 128 138 147 154 163
F172 128 138 144 147 163
F173 128 144 147 150 163
F174 138 147 150 154
F175 128 134 138 144 154
F176 128 134 144 147 154
F177 144 147 150 154
F178 128 134 147 150 154
F179 128 134 138 144 150

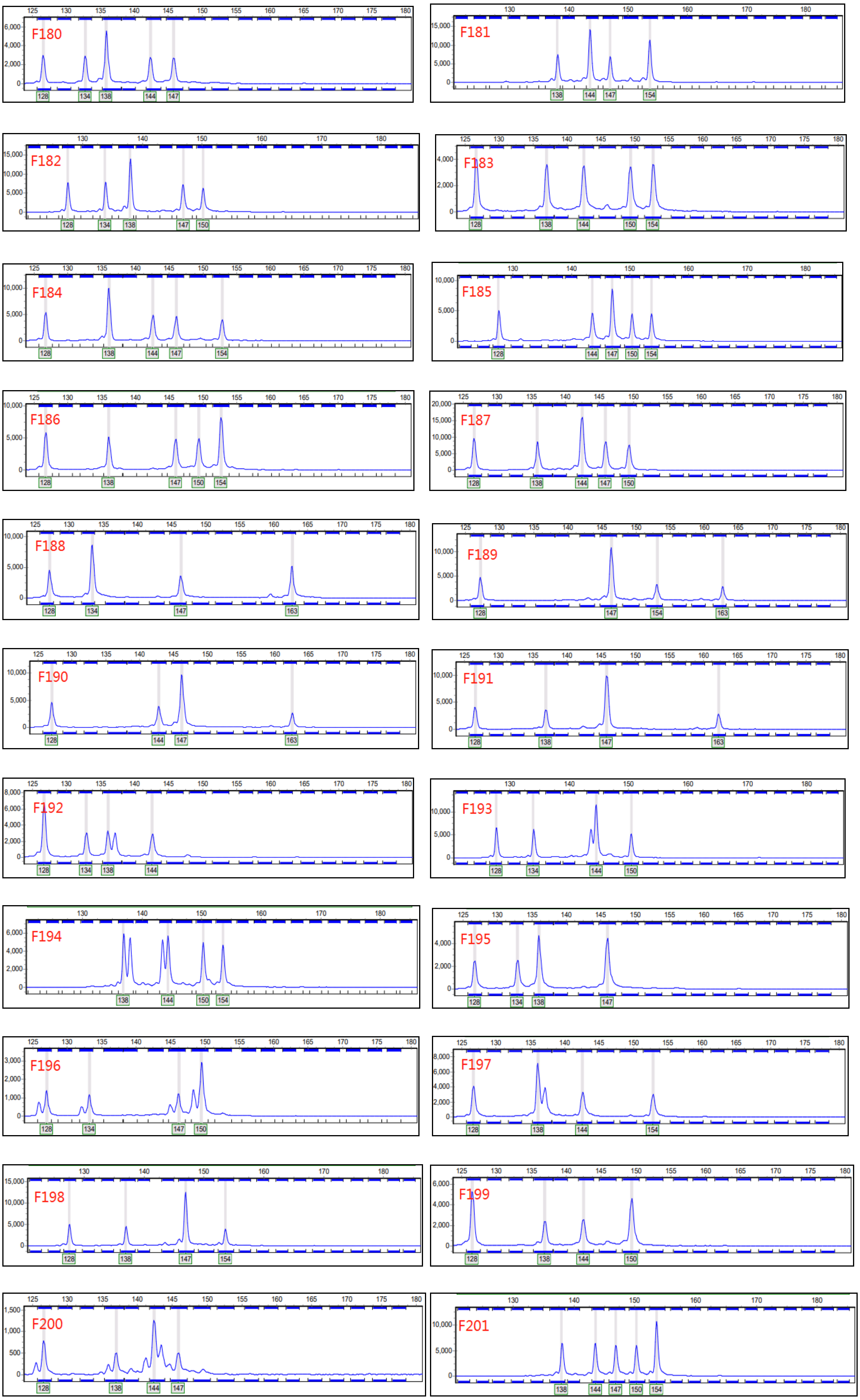
F180
F181
F182
F183
F184
F185
F186
F187
F188
F189
F190
F191
F192
F193
F194
F195
F196
F197
F198
F199
F200
F201

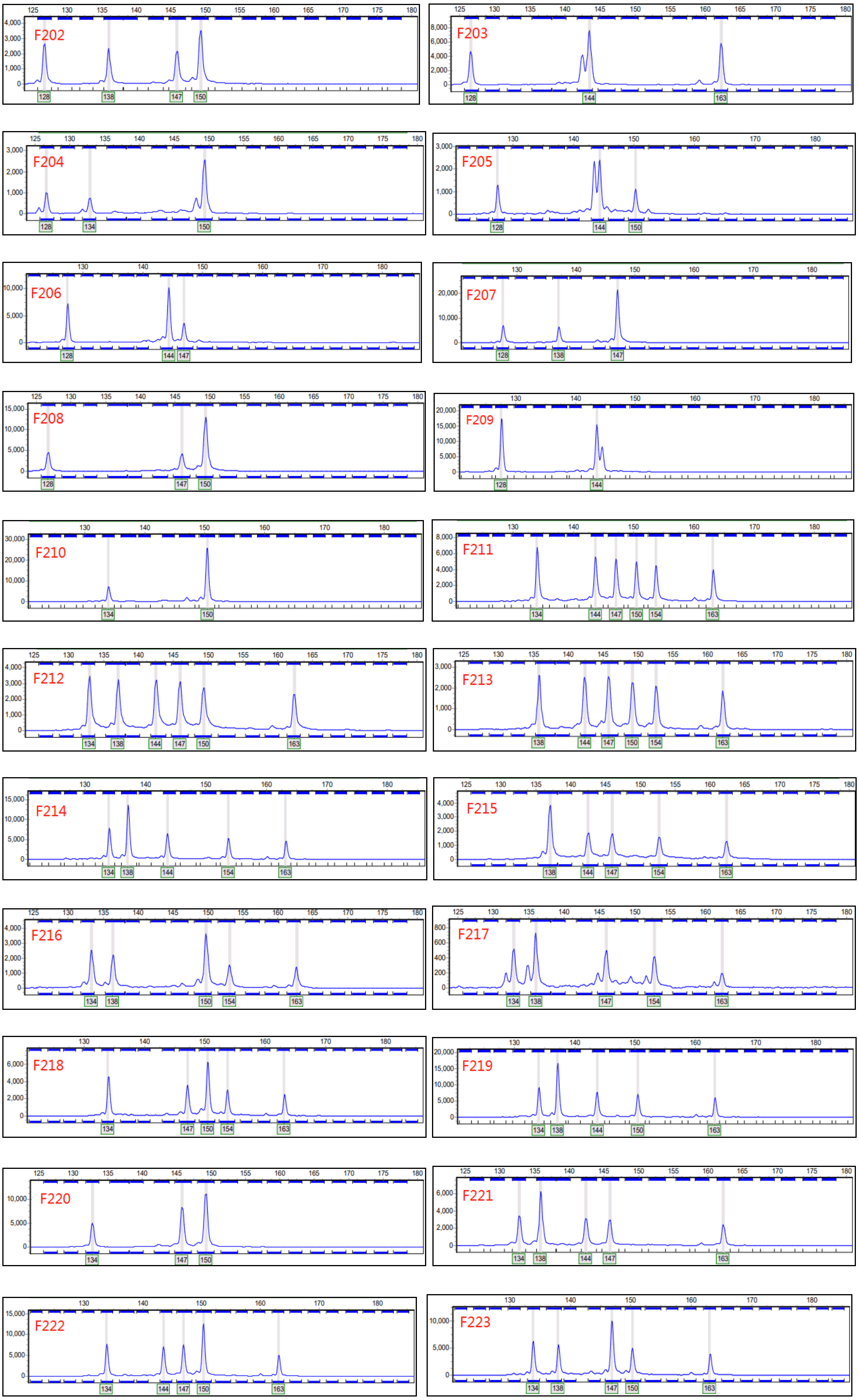
F202
128 138 147 150
F203
128 144 163
F204
128 134 150
F205
128 144 150
F206
128 144 147
F207
128 138 147
F208
128 147 150
F209
128 144
F210
134 150
F211
134 144 147 150 154 163
F212
134 138 144 147 150 163
F213
138 144 147 150 154 163
F214
134 138 144 154 163
F215
138 144 147 154 163
F216
134 138 150 154 163
F217
134 138 147 154 163
F218
134 147 150 154 163
F219
134 138 144 150 163
F220
134 147 150
F221
134 138 144 147 163
F222
134 144 147 150 163
F223
134 138 147 150 163

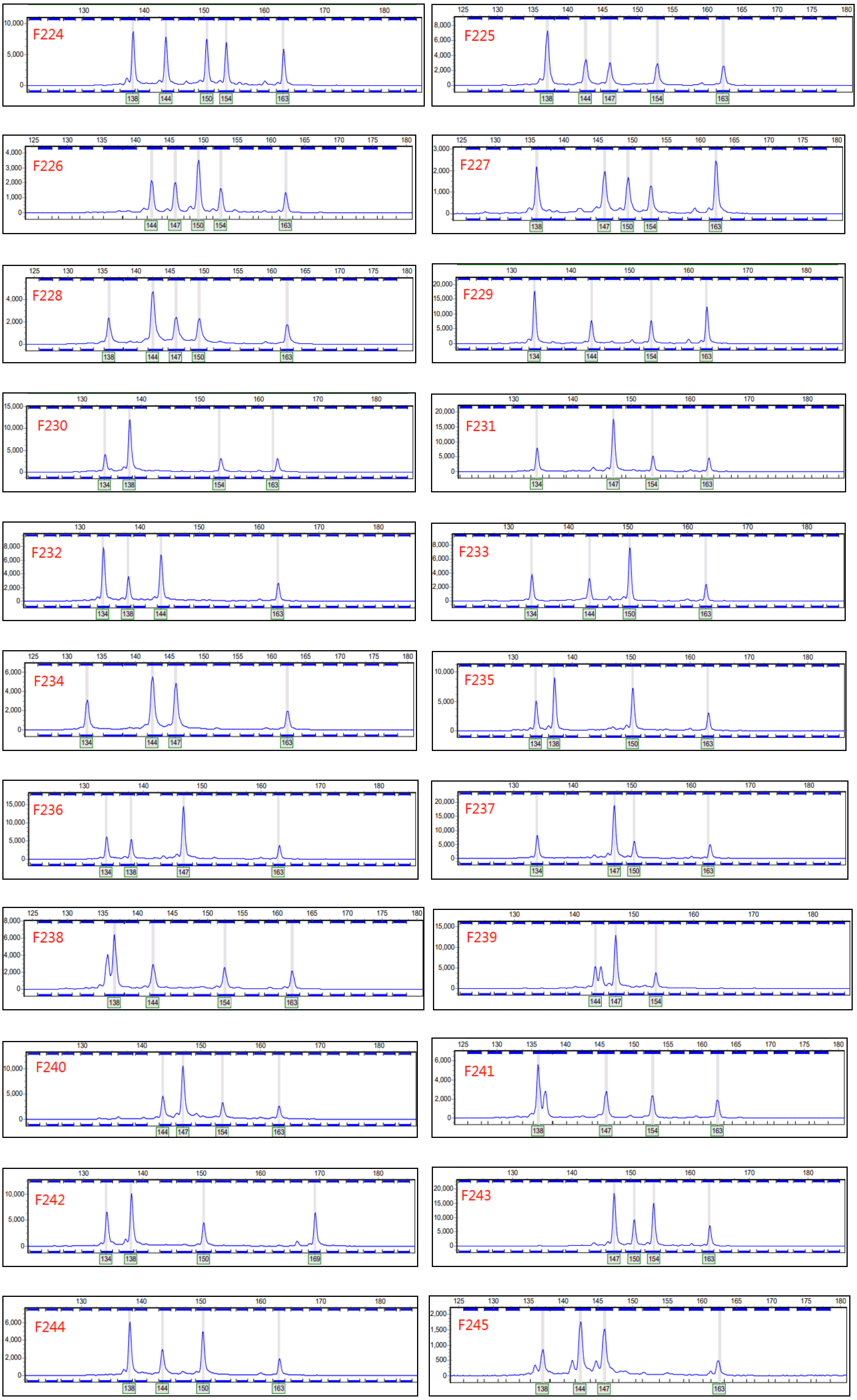
F224
138 144 150 154 163
F225
138 144 147 154 163
F226
144 147 150 154 163
F227
138 147 150 154 163
F228
138 144 147 150 163
F229
134 144 154 163
F230
134 138 154 163
F231
134 147 154 163
F232
134 138 144 163
F233
134 144 150 163
F234
134 144 147 163
F235
134 138 150 163
F236
134 138 147 163
F237
134 147 150 163
F238
138 144 154 163
F239
144 147 154
F240
144 147 154 163
F241
138 147 154 163
F242
134 138 150 169
F243
147 150 154 163
F244
138 144 150 163
F245
138 144 147 163

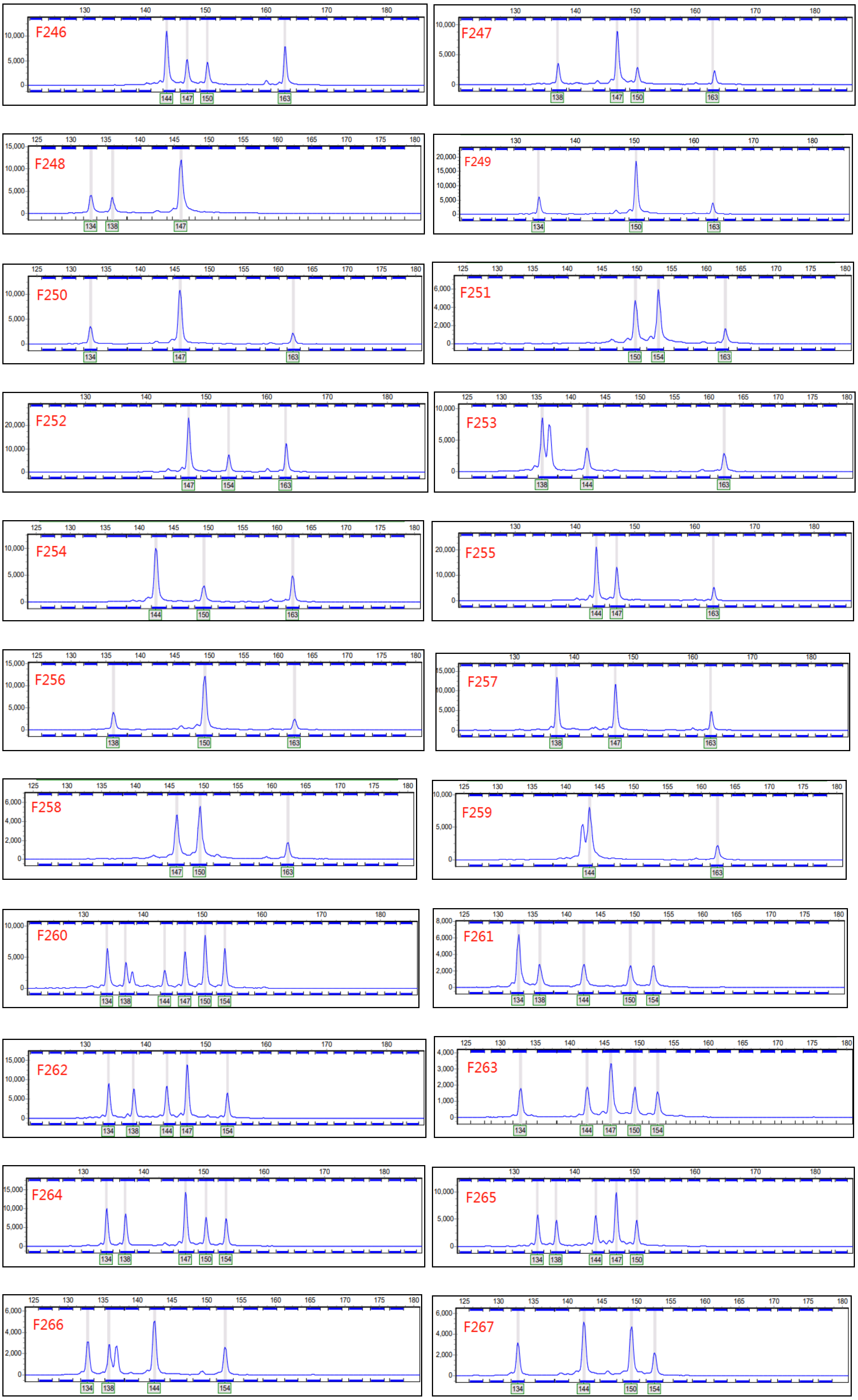
F246
F247
F248
F249
F250
F251
F252
F253
F254
F255
F256
F257
F258
F259
F260
F261
F262
F263
F264
F265
F266
F267

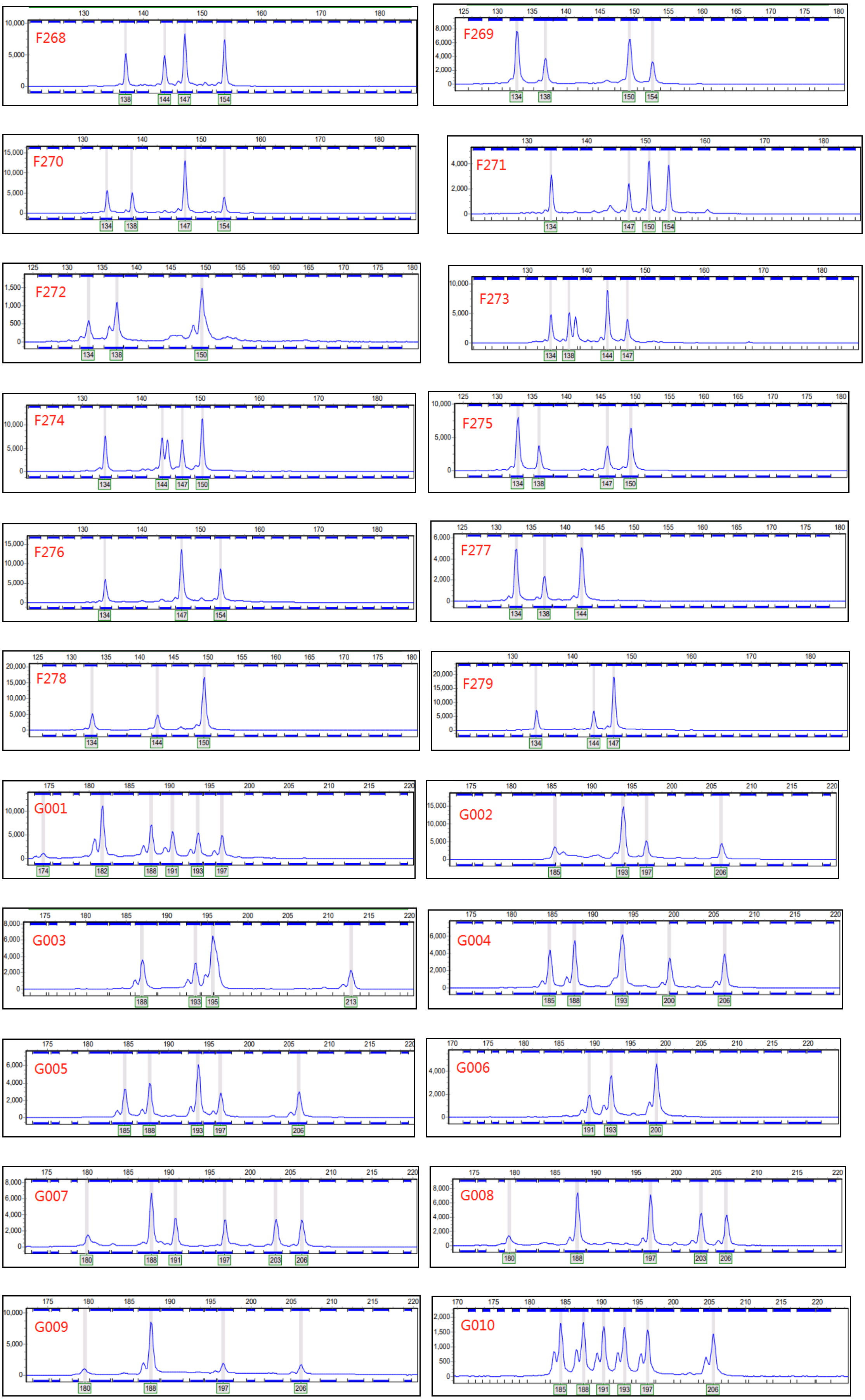
F268
138 144 147 154
F269
134 138 150 154
F270
134 138 147 154
F271
134 147 150 154
F272
134 138 150
F273
134 138 144 147
F274
134 144 147 150
F275
134 138 147 150
F276
134 147 154
F277
134 138 144
F278
134 144 150
F279
134 144 147
G001
174 182 188 191 193 197
G002
185 193 197 206
G003
188 193 195 213
G004
185 188 193 200 206
G005
185 188 193 197 206
G006
191 193 200
G007
180 188 191 197 203 206
G008
180 188 197 203 206
G009
180 188 197 206
G010
185 188 191 193 197 206

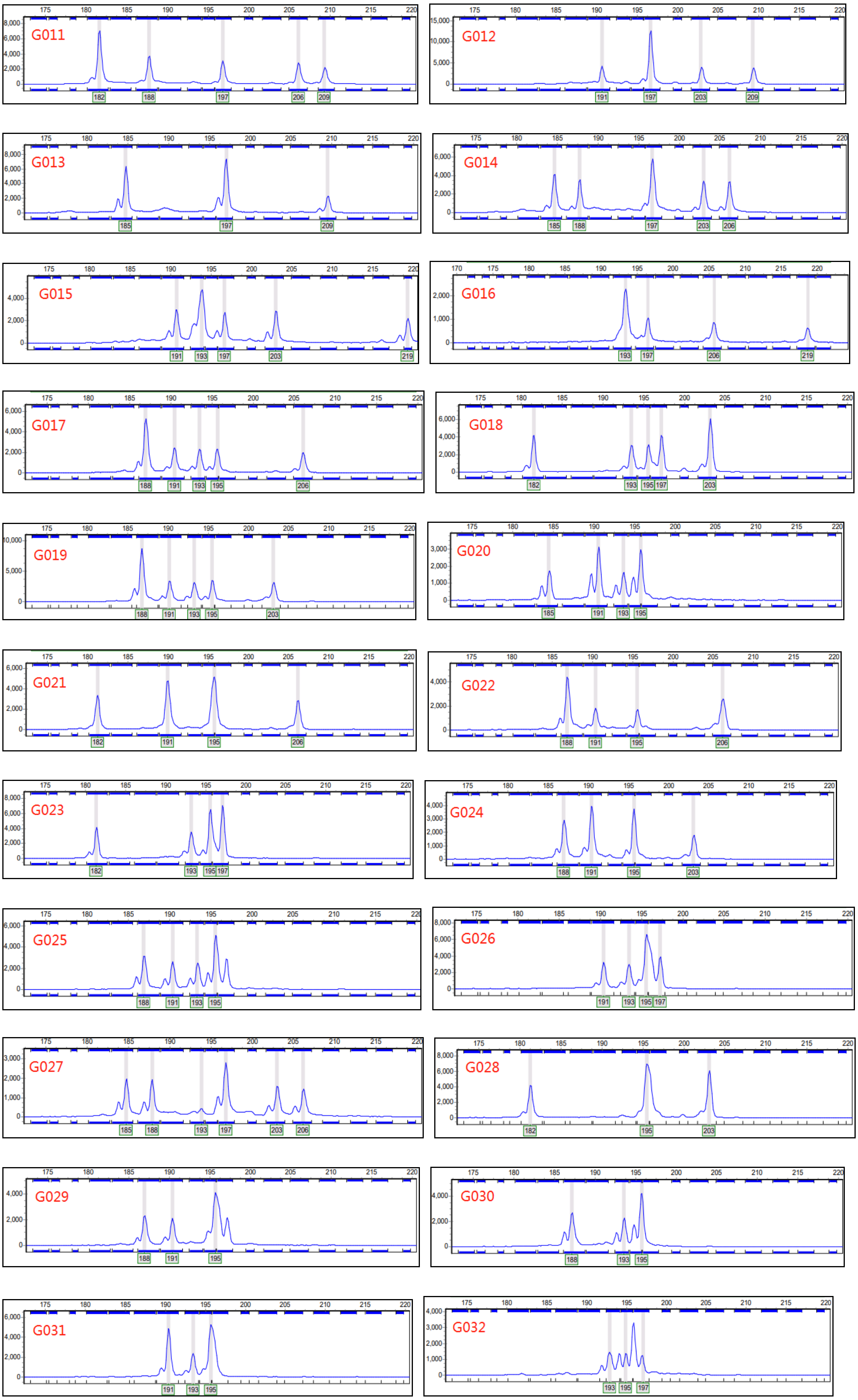
G011 182 188 197 206 209
G012 191 197 203 209
G013 185 197 209
G014 185 188 197 203 206
G015 191 193 197 203 219
G016 193 197 206 219
G017 188 191 193 195 206
G018 182 193 195 197 203
G019 188 191 193 195 203
G020 185 191 193 195
G021 182 191 195 206
G022 188 191 195 206
G023 182 193 195 197
G024 188 191 195 203
G025 188 191 193 195
G026 191 193 195 197
G027 185 188 193 197 203 206
G028 182 195 203
G029 188 191 195
G030 188 193 195
G031 191 193 195
G032 193 195 197

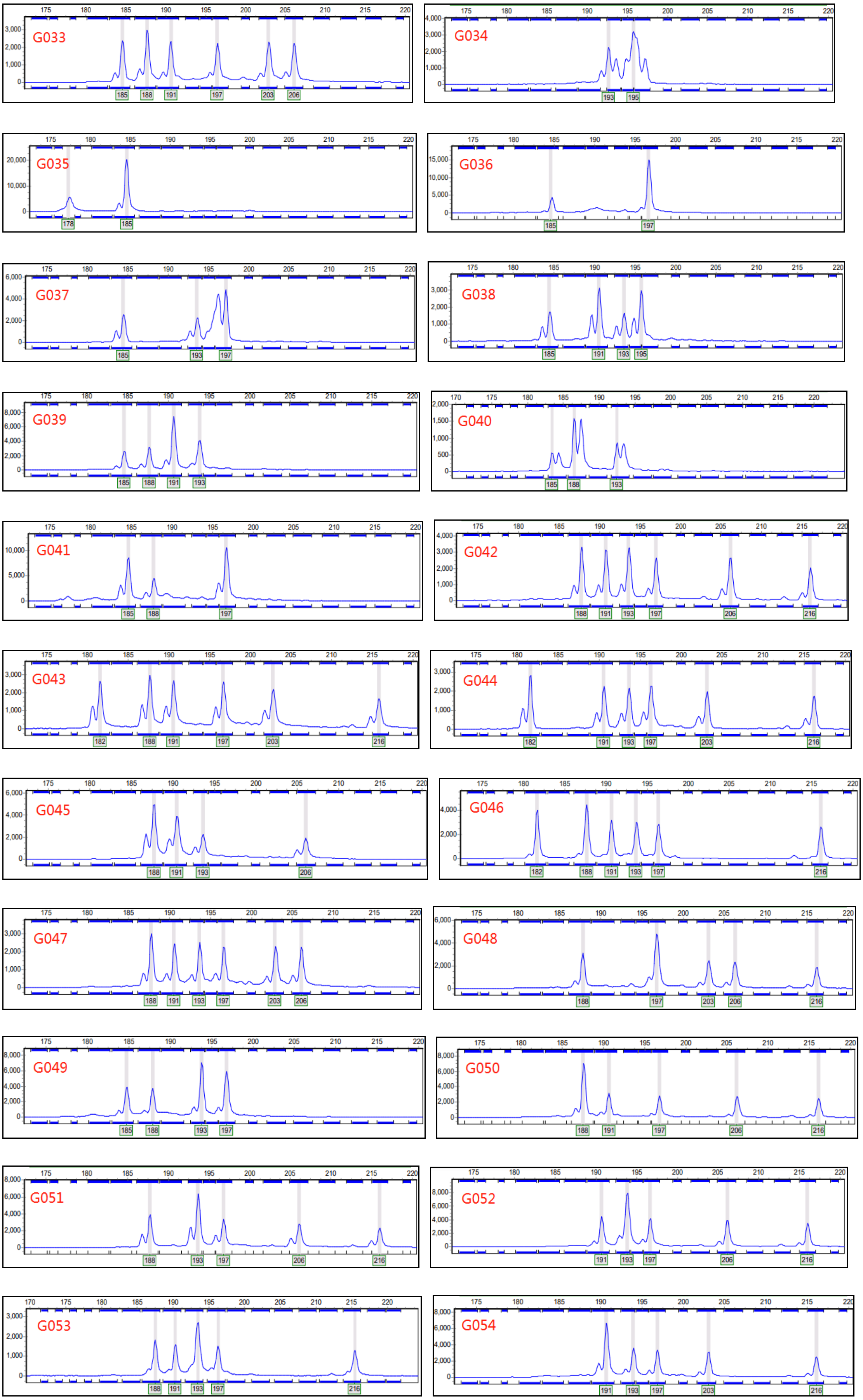
G033
185 188 191 197 203 206
G034
193 195
G035
178 185
G036
185 197
G037
185 193 197
G038
185 191 193 195
G039
185 188 191 193
G040
185 188 193
G041
185 188 197
G042
188 191 193 197 206 216
G043
182 188 191 197 203 216
G044
182 191 193 197 203 216
G045
188 191 193 206
G046
182 188 191 193 197 216
G047
188 191 193 197 203 206
G048
188 197 203 206 216
G049
185 188 193 197
G050
188 191 197 206 216
G051
188 193 197 206 216
G052
191 193 197 206 216
G053
188 191 193 197 216
G054
191 193 197 203 216

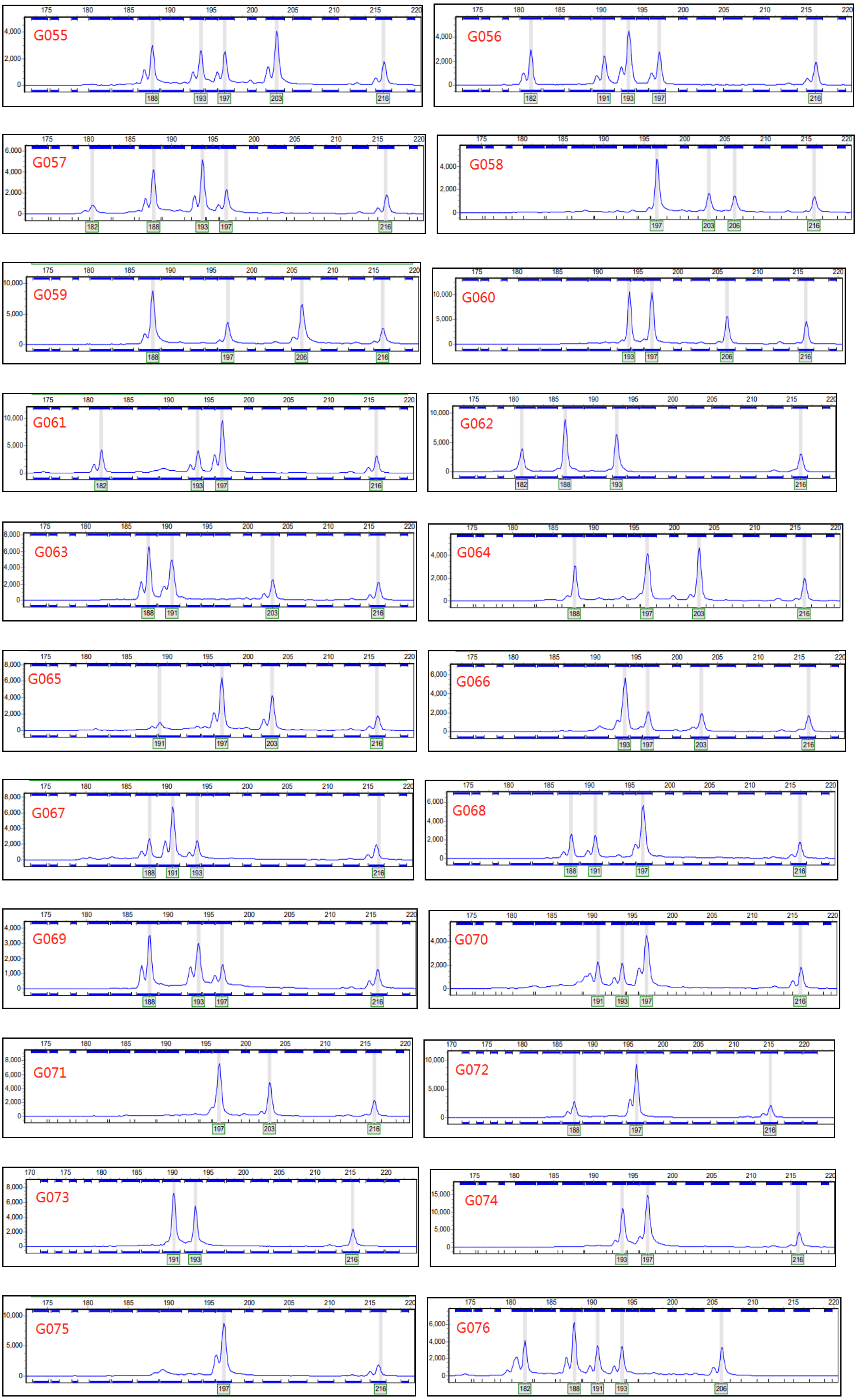

G055 188 193 197 203 216
G056 182 191 193 197 216
G057 182 188 193 197 216
G058 197 203 206 216
G059 188 197 206 216
G060 193 197 206 216
G061 182 193 197 216
G062 182 188 193 216
G063 188 191 203 216
G064 188 197 203 216
G065 191 197 203 216
G066 193 197 203 216
G067 188 191 193 216
G068 188 191 197 216
G069 188 193 197 216
G070 191 193 197 216
G071 197 203 216
G072 188 197 216
G073 191 193 216
G074 193 197 216
G075 197 216
G076 182 188 191 193 206

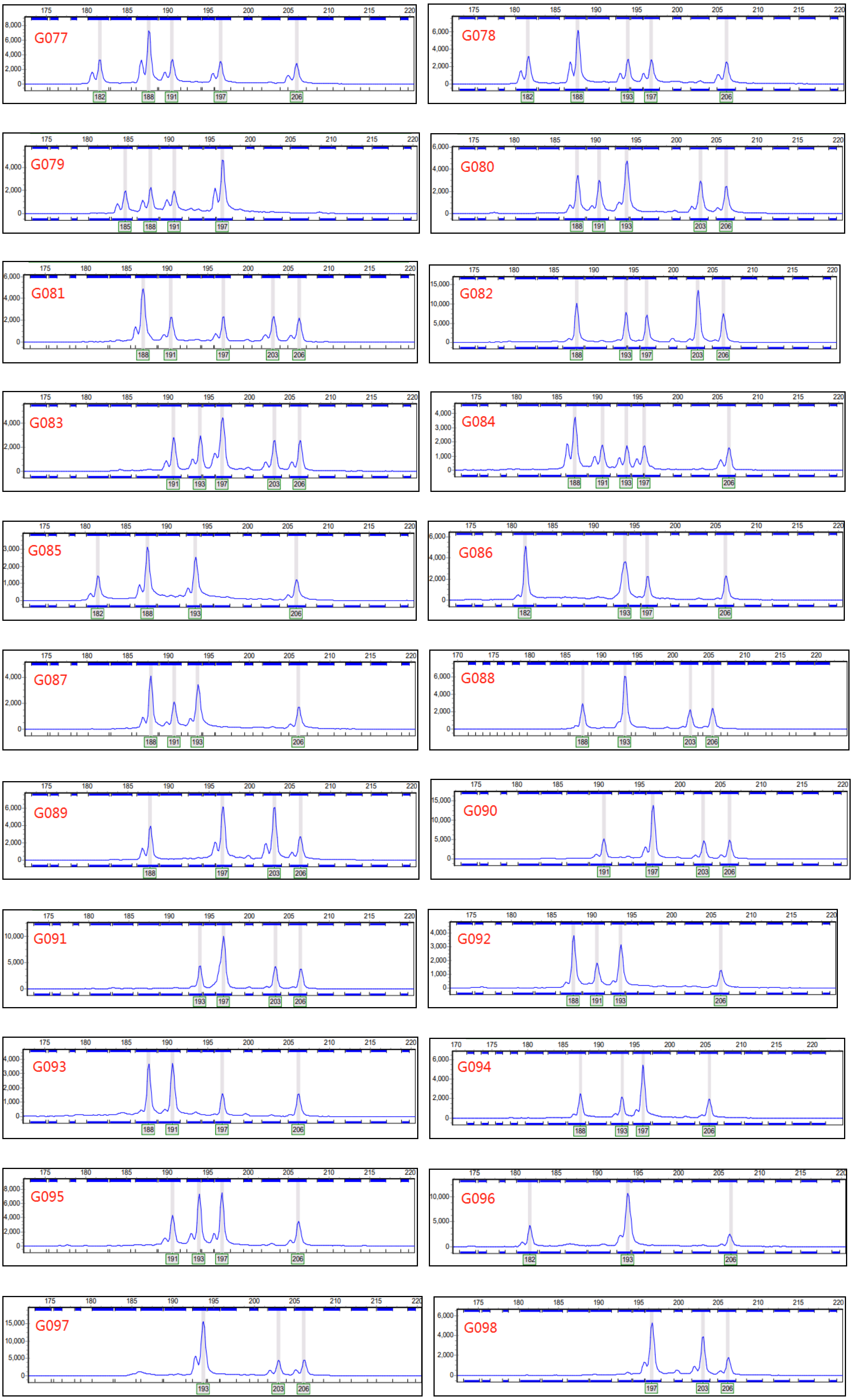
G077
182 188 191 197 206
G078
182 188 193 197 206
G079
185 188 191 197
G080
188 191 193 203 206
G081
188 191 197 203 206
G082
188 193 197 203 206
G083
191 193 197 203 206
G084
188 191 193 197 206
G085
182 188 193 206
G086
182 193 197 206
G087
188 191 193 206
G088
188 193 203 206
G089
188 197 203 206
G090
191 197 203 206
G091
193 197 203 206
G092
188 191 193 206
G093
188 191 197 206
G094
188 193 197 206
G095
191 193 197 206
G096
182 193 206
G097
193 203 206
G098
197 203 206

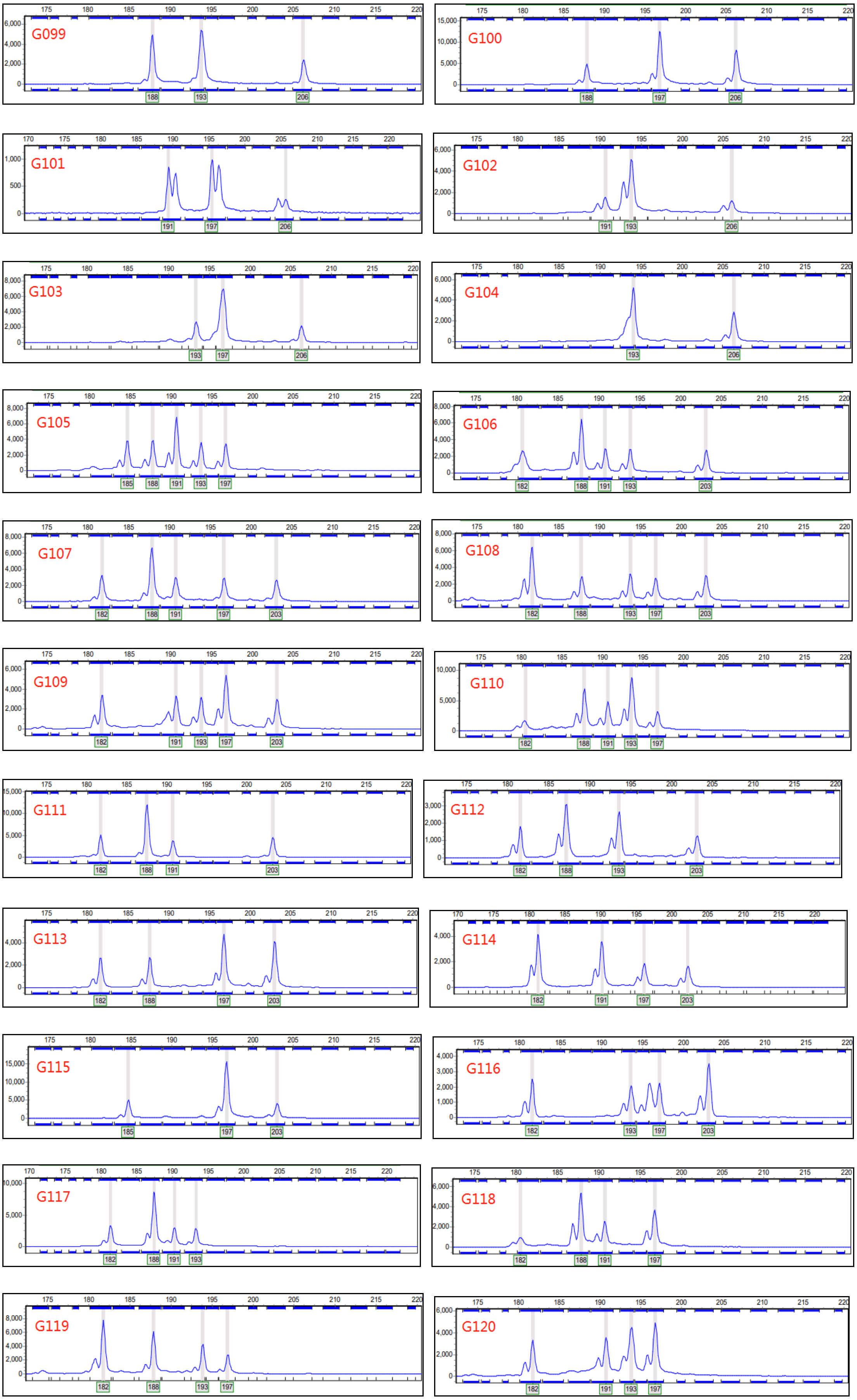
G099 188 193 206
G100 188 197 206
G101 191 197 206
G102 191 193 206
G103 193 197 206
G104 193 206
G105 185 188 191 193 197
G106 182 188 191 193 203
G107 182 188 191 197 203
G108 182 188 193 197 203
G109 182 191 193 197 203
G110 182 188 191 193 197
G111 182 188 191 203
G112 182 188 193 203
G113 182 188 197 203
G114 182 191 197 203
G115 185 197 203
G116 182 193 197 203
G117 182 188 191 193
G118 182 188 191 197
G119 182 188 193 197
G120 182 191 193 197

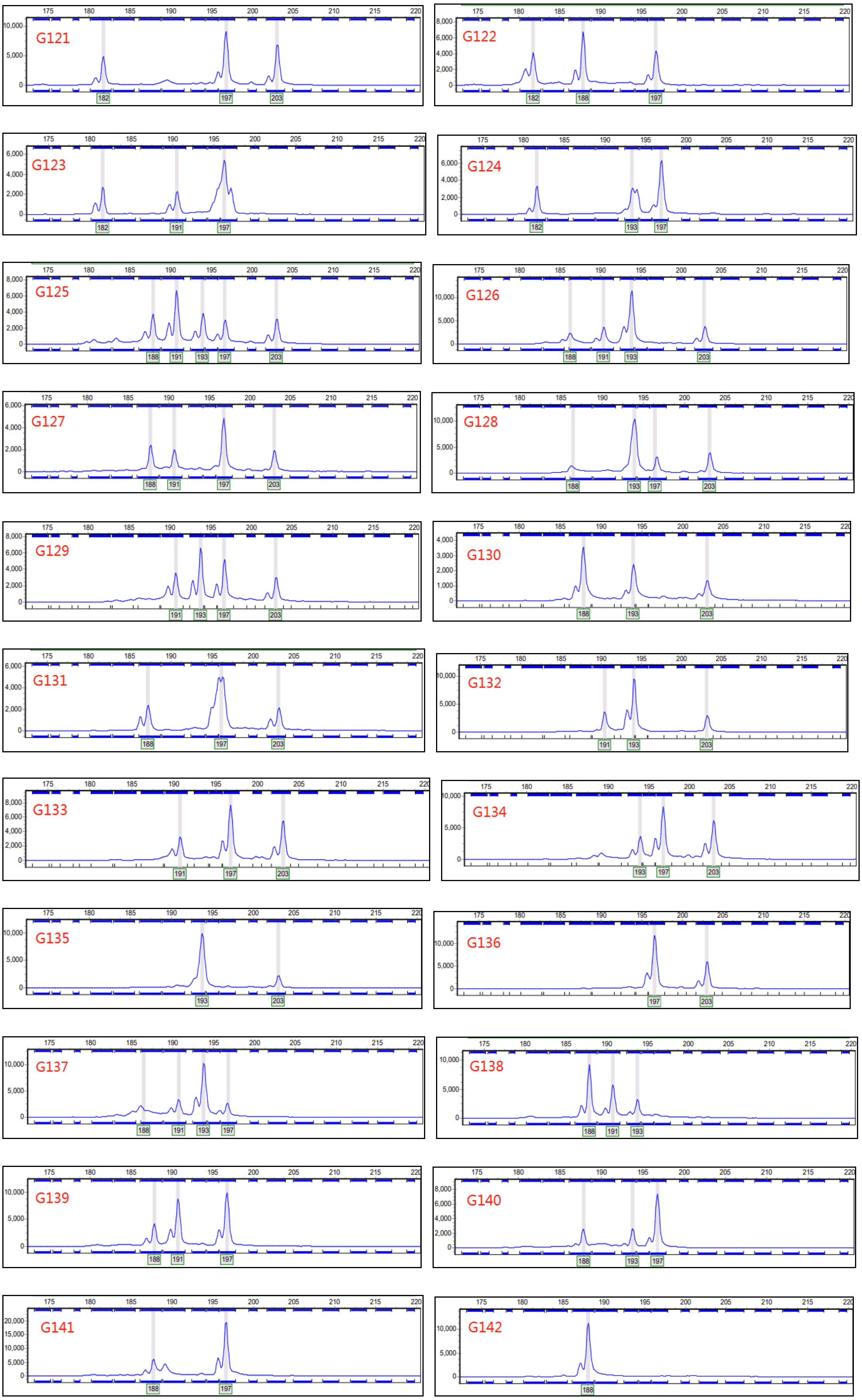
G121 182 197 203
G122 182 188 197
G123 182 191 197
G124 182 193 197
G125 188 191 193 197 203
G126 188 191 193 203
G127 188 191 197 203
G128 188 193 197 203
G129 191 193 197 203
G130 188 193 203
G131 188 197 203
G132 191 193 203
G133 191 197 203
G134 193 197 203
G135 193 203
G136 197 203
G137 188 191 193 197
G138 188 191 193
G139 188 191 197
G140 188 193 197
G141 188 197
G142 188

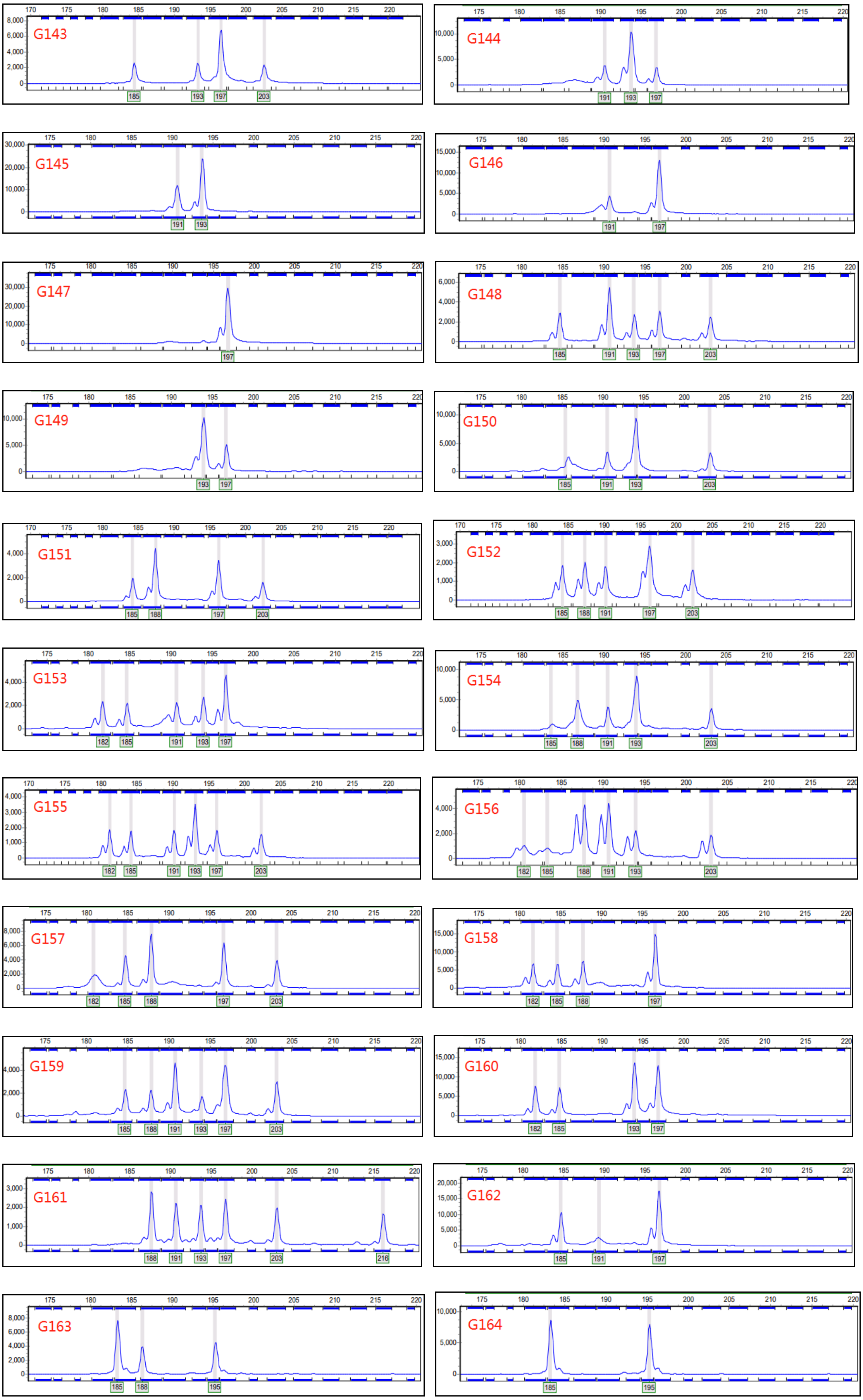
G143 185 193 197 203
G144 191 193 197
G145 191 193
G146 191 197
G147 197
G148 185 191 193 197 203
G149 193 197
G150 185 191 193 203
G151 185 188 197 203
G152 185 188 191 197 203
G153 182 185 191 193 197
G154 185 188 191 193 203
G155 182 185 191 193 197 203
G156 182 185 188 191 193 203
G157 182 185 188 197 203
G158 182 185 188 197
G159 185 188 191 193 197 203
G160 182 185 193 197
G161 188 191 193 197 203 216
G162 185 191 197
G163 185 188 195
G164 185 195

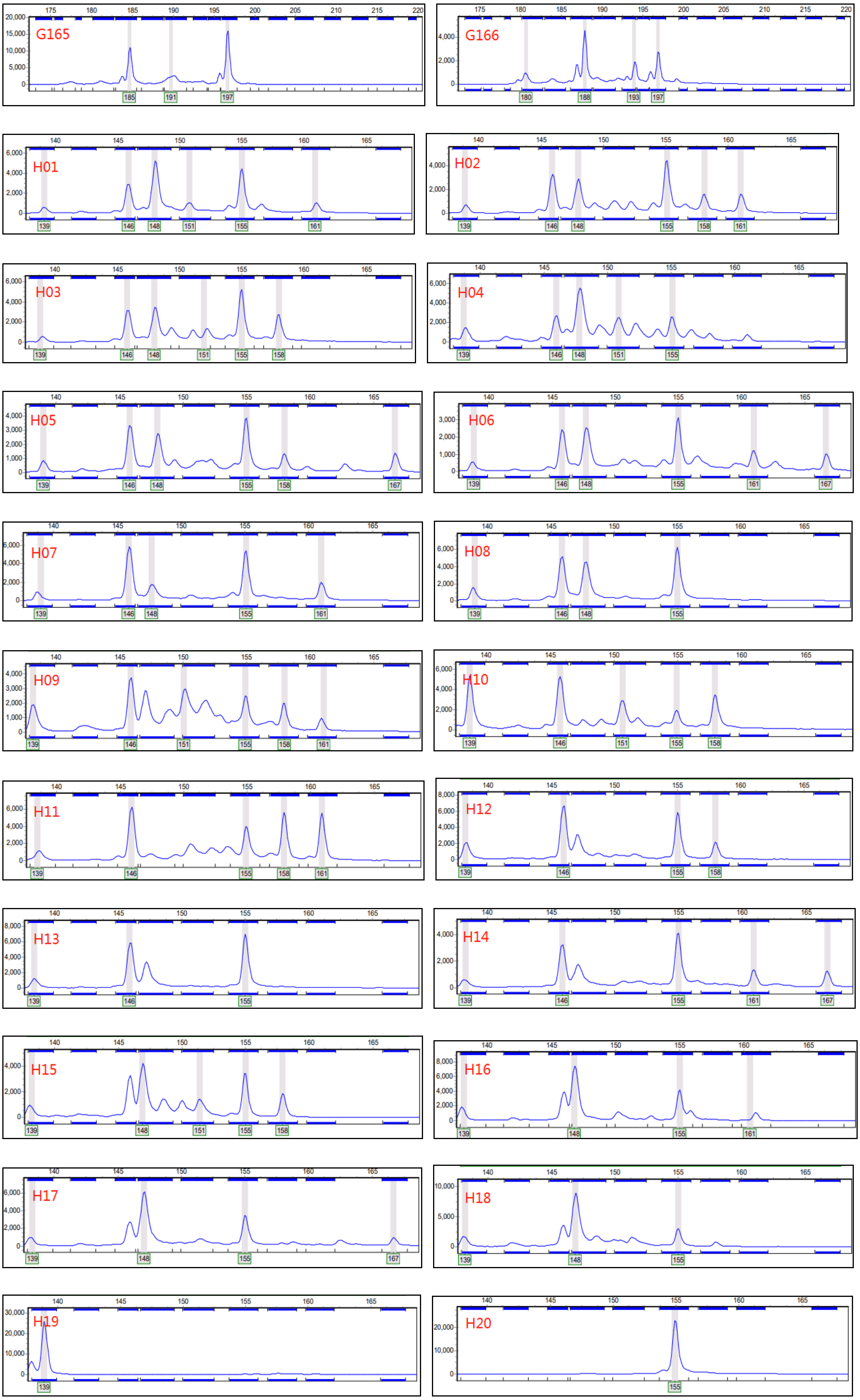
G165
185 191 197
G166
180 188 193 197
H01
139 146 148 151 155 161
H02
139 146 148 155 158 161
H03
139 146 148 151 155 158
H04
139 146 148 151 155
H05
139 146 148 155 158 167
H06
139 146 148 155 161 167
H07
139 146 148 155 161
H08
139 146 148 155
H09
139 146 151 155 158 161
H10
139 146 151 155 158
H11
139 146 155 158 161
H12
139 146 155 158
H13
139 146 155
H14
139 146 155 161 167
H15
139 148 151 155 158
H16
139 148 155 161
H17
139 148 155 167
H18
139 148 155
H19
139
H20
155

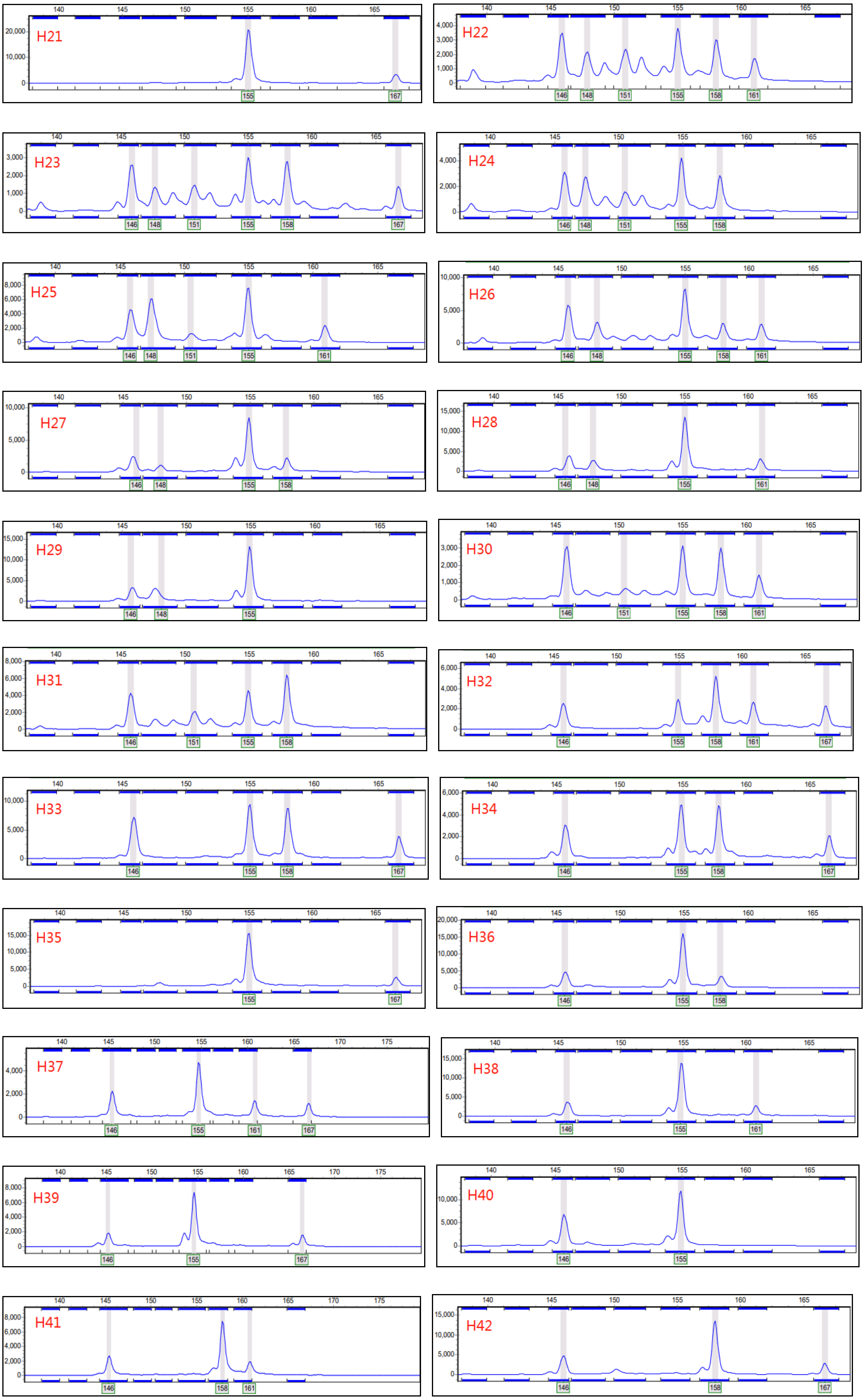
H21
155 167
H22
146 148 151 155 158 161
H23
146 148 151 155 158 167
H24
146 148 151 155 158
H25
146 148 151 155 161
H26
146 148 155 158 161
H27
146 148 155 158
H28
146 148 155 161
H29
146 148 155
H30
146 151 155 158 161
H31
146 151 155 158
H32
146 155 158 161 167
H33
146 155 158 167
H34
146 155 158 167
H35
155 167
H36
146 155 158
H37
146 155 161 167
H38
146 155 161
H39
146 155 167
H40
146 155
H41
146 158 161
H42
146 158 167

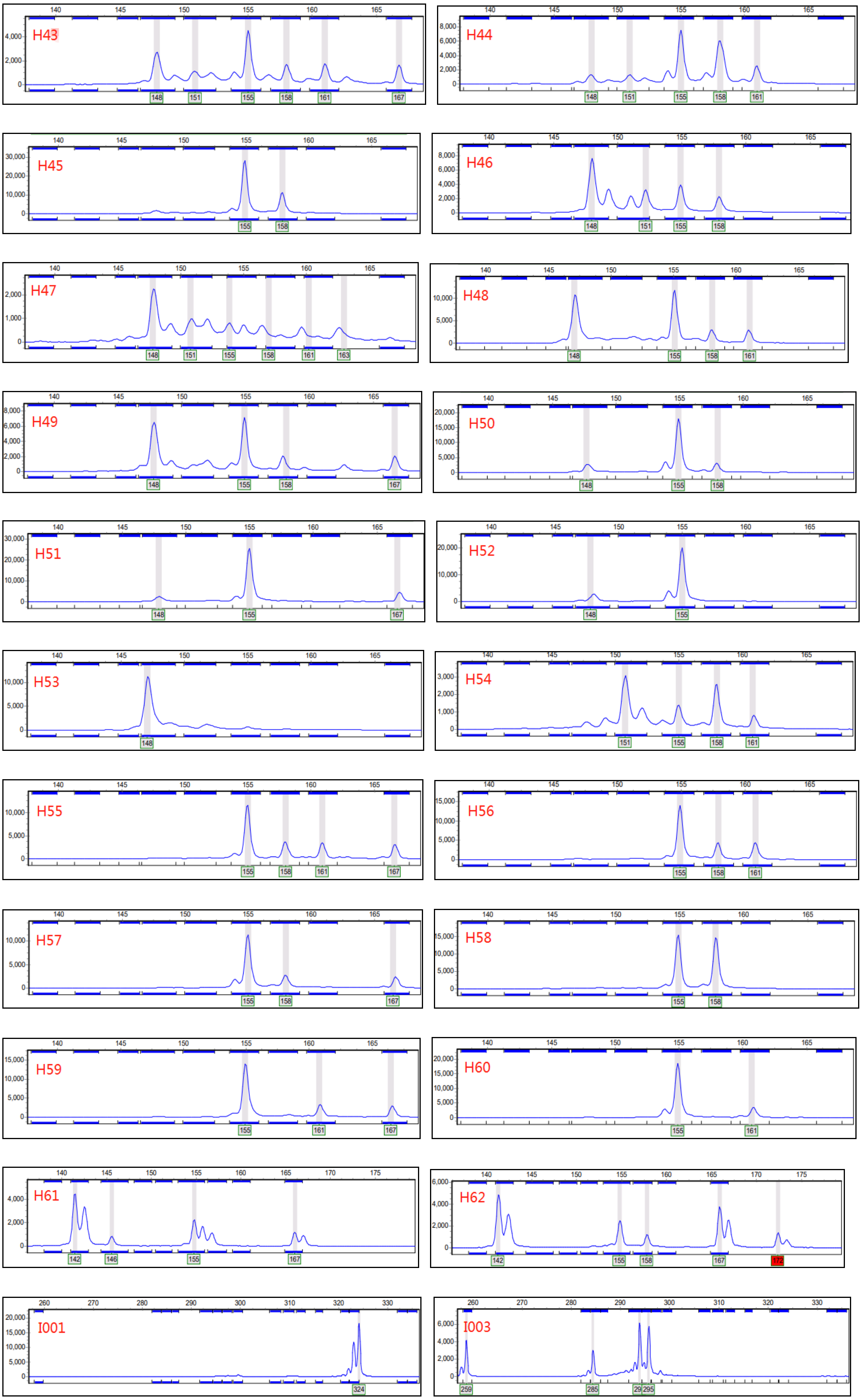
H43
H44
H45
H46
H47
H48
H49
H50
H51
H52
H53
H54
H55
H56
H57
H58
H59
H60
H61
H62
I001
I003

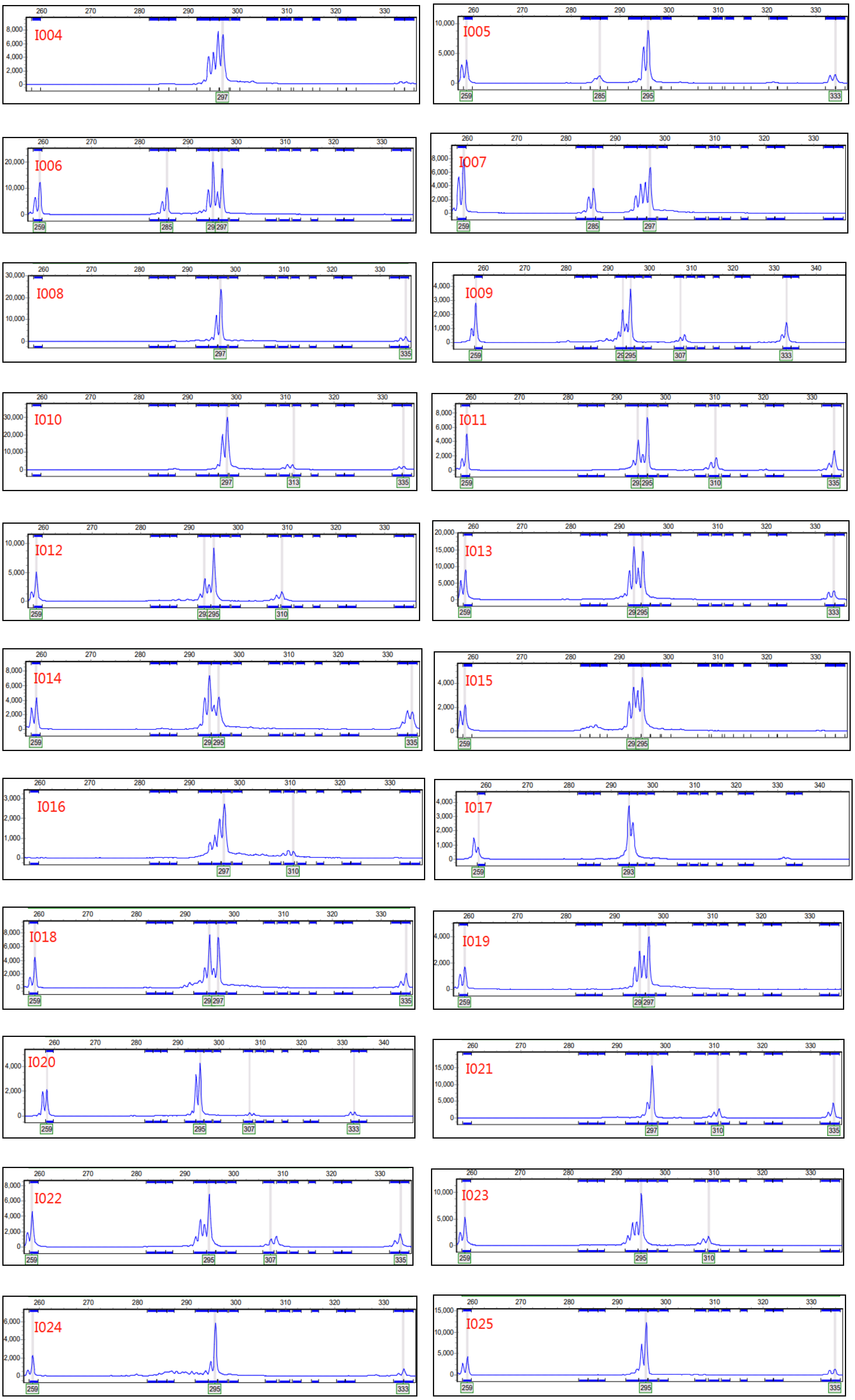

I004
I005
I006
I007
I008
I009
I010
I011
I012
I013
I014
I015
I016
I017
I018
I019
I020
I021
I022
I023
I024
I025

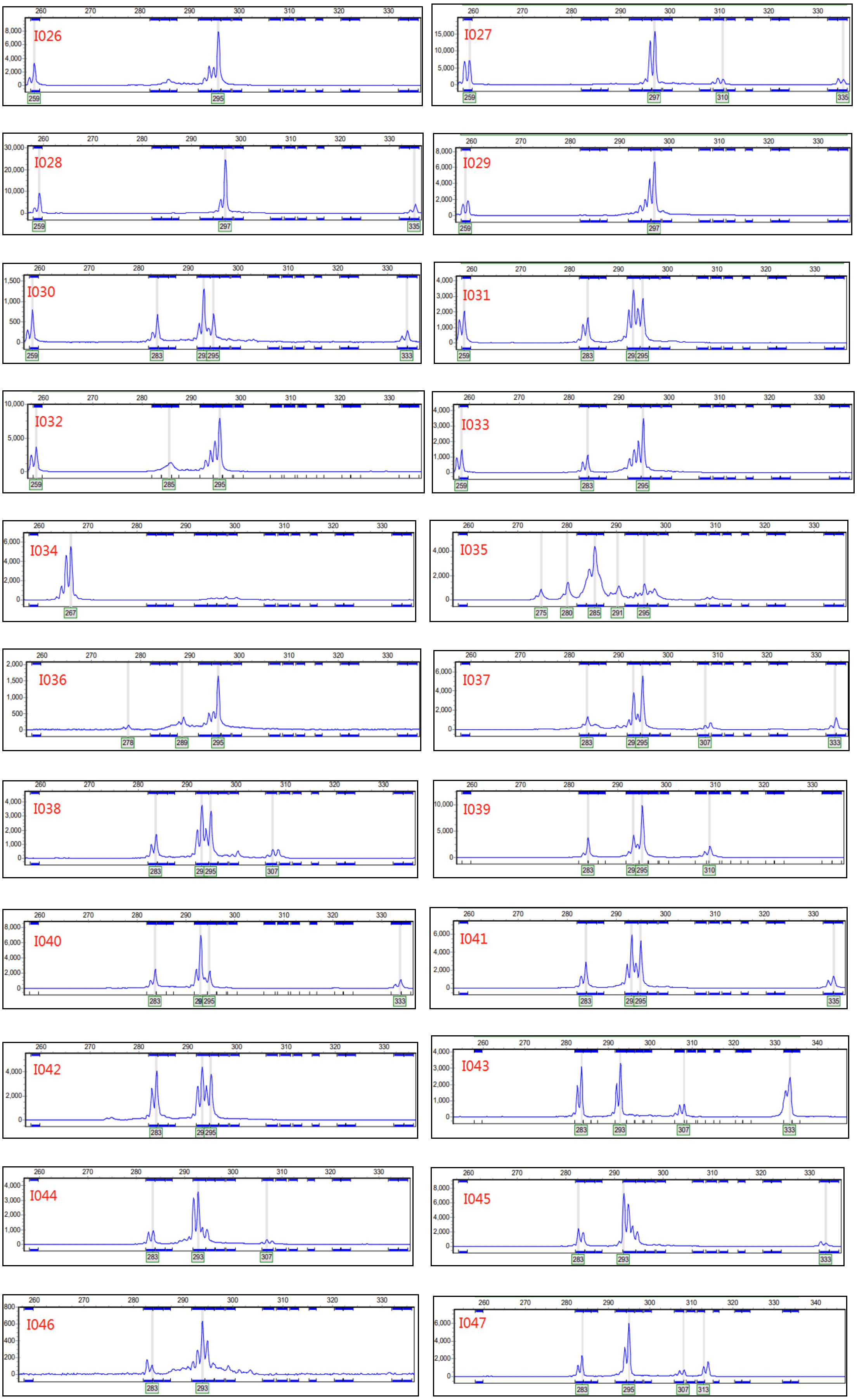
I026
I027
I028
I029
I030
I031
I032
I033
I034
I035
I036
I037
I038
I039
I040
I041
I042
I043
I044
I045
I046
I047

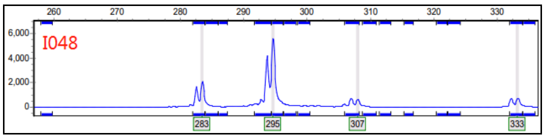
I048
283
295
307
333

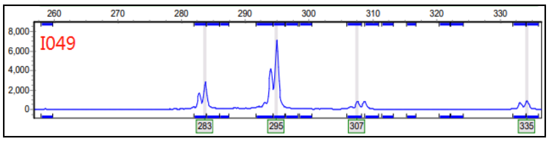
I049
283
295
307
335

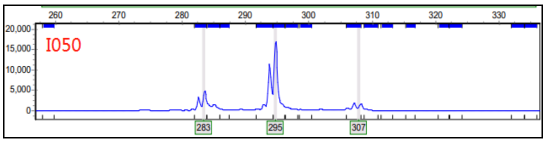
I050
283
295
307

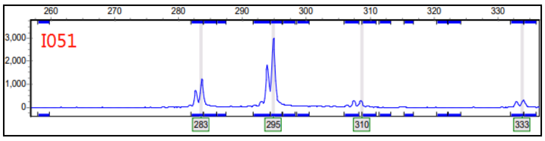
I051
283
295
310
333

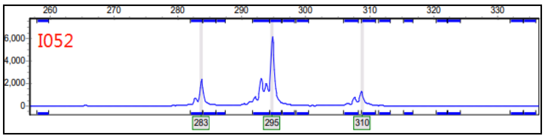
I052
283
295
310

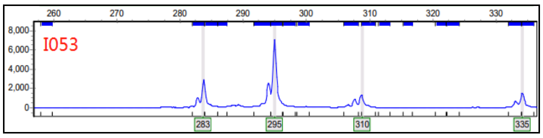
I053
283
295
310
335

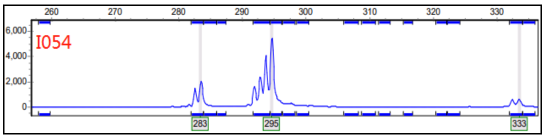
I054
283
295
333

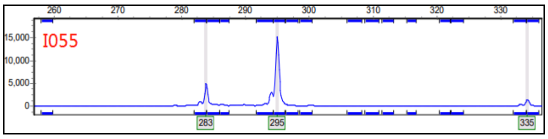
I055
283
295
335

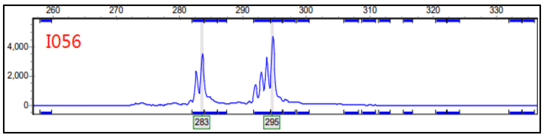
I056
283
295

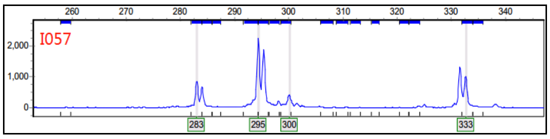
I057
283
295
300
333

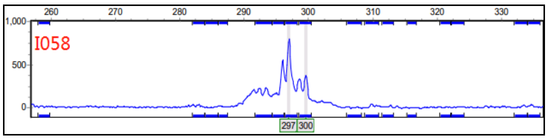
I058
297
300

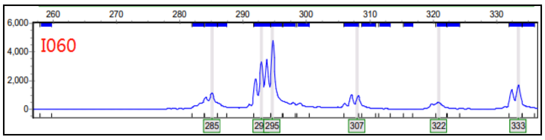
I060
285
29
295
307
322
333

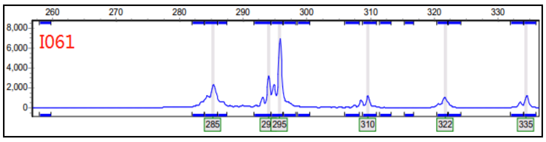
I061
285
29
295
310
322
335

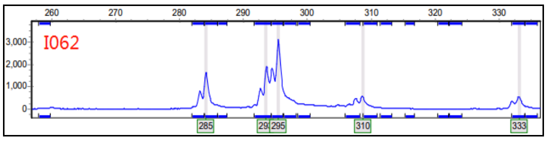
I062
285
29
295
310
333

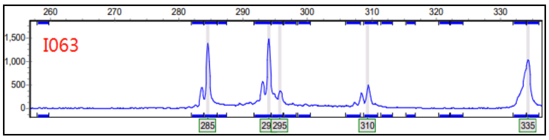
I063
285
29
295
310
335

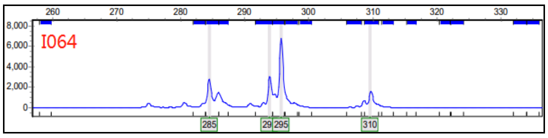
I064
285
29
295
310

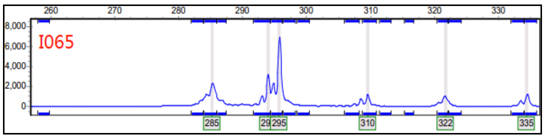
I065
285
29
295
310
322
335

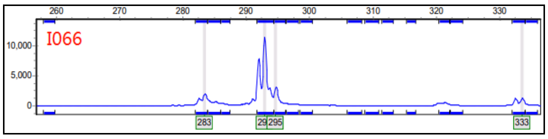
I066
283
29
295
333

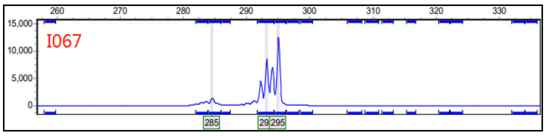
I067
285
29
295

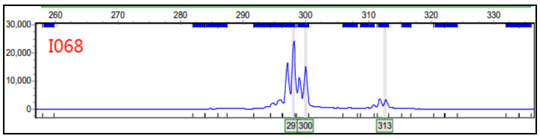
I068
29
300
313

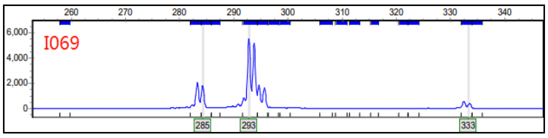
I069
285
293
333

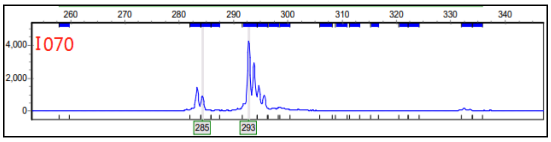
I070
285
293

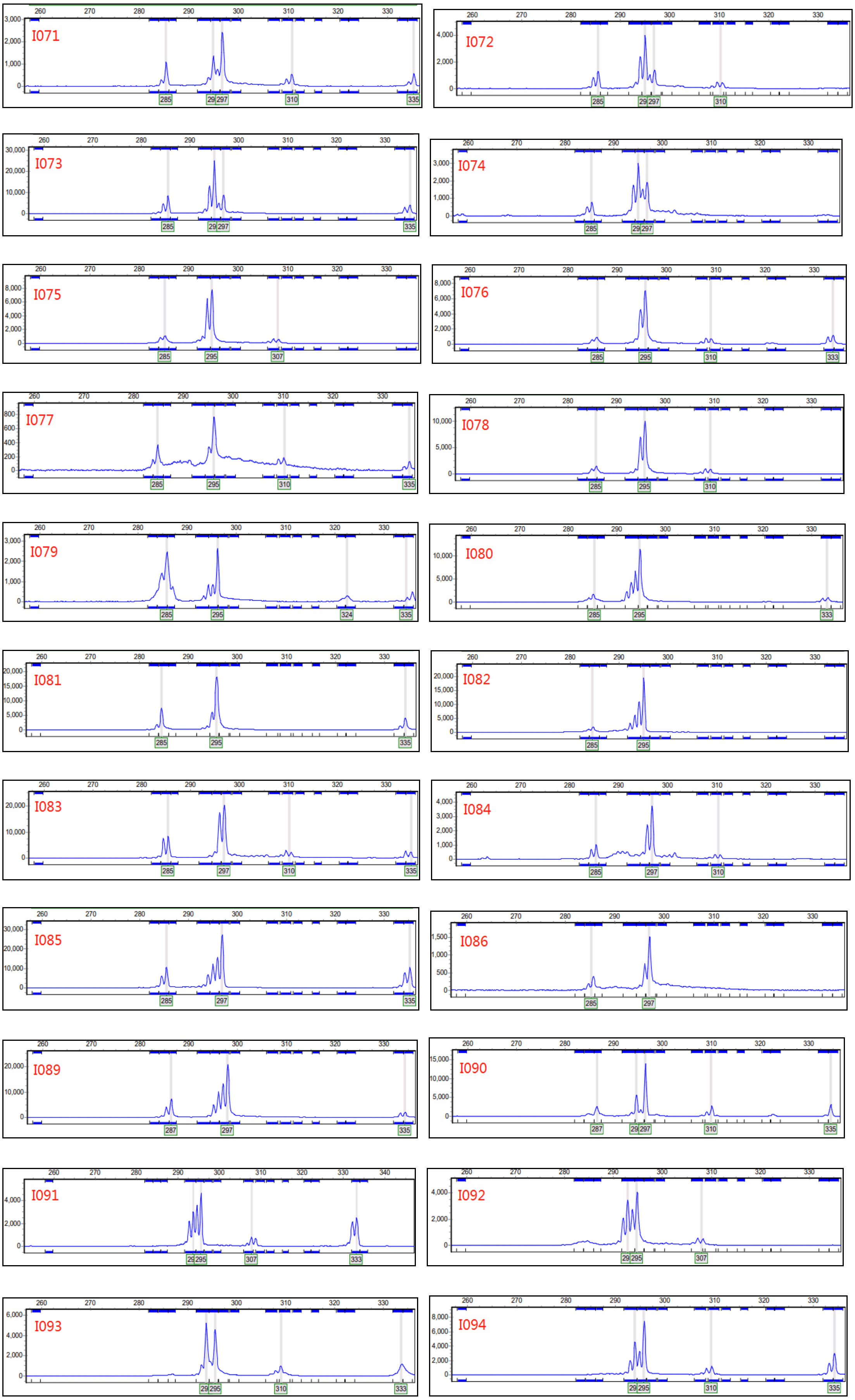
I071
I072
I073
I074
I075
I076
I077
I078
I079
I080
I081
I082
I083
I084
I085
I086
I089
I090
I091
I092
I093
I094

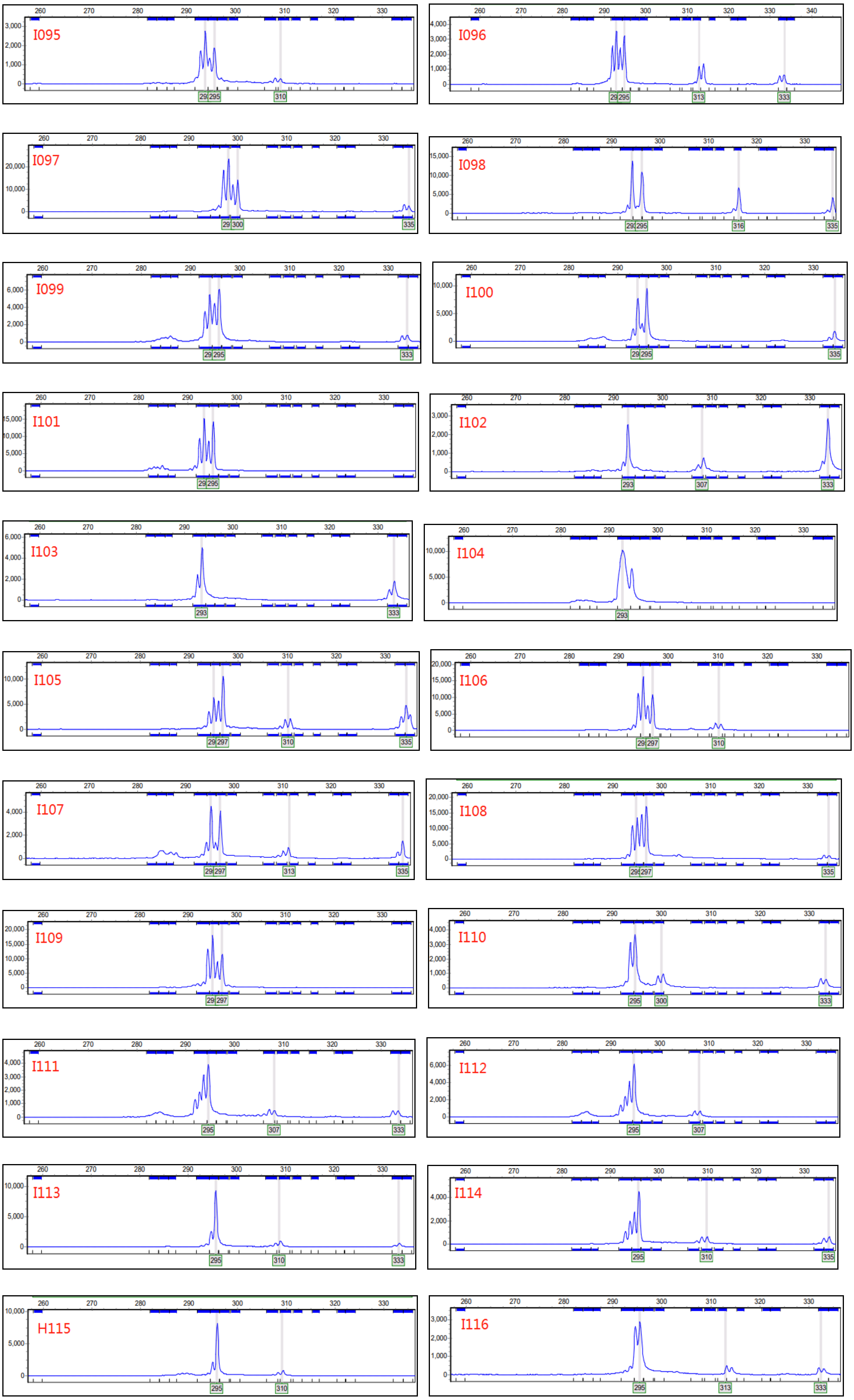
I095 29 295 310
I096 29 295 313 333
I097 29 300 335
I098 29 295 316 335
I099 29 295 333
I100 29 295 335
I101 29 295
I102 293 307 333
I103 293 333
I104 293
I105 29 297 310 335
I106 29 297 310
I107 29 297 313 335
I108 29 297 335
I109 29 297
I110 295 300 333
I111 295 307 333
I112 295 307
I113 295 310 333
I114 295 310 335
H115 295 310
I116 295 313 333

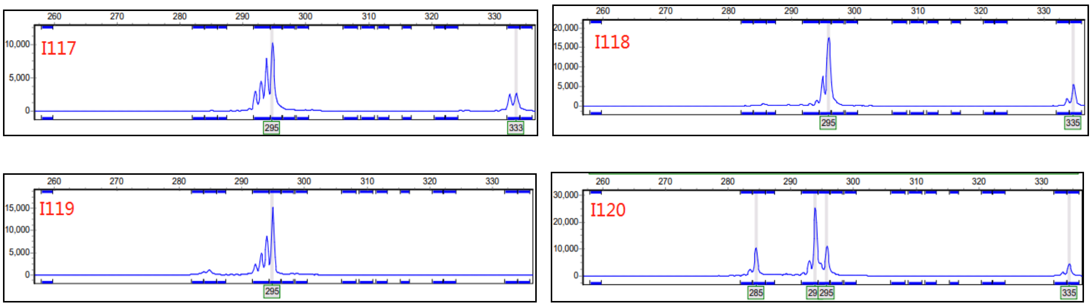
I117
295
333
I118
295
335
I119
295
I120
285
29
295
335
