# Supplementary material for: Genetic fingerprint construction and genetic diversity analysis of sweet potato (Ipomoea batatas) germplasm resources
Source: BMC Plant Biol. 2023 Jul 11;23:355. doi: 10.1186/s12870-023-04329-1 (PMC10334575; doi:10.1186/s12870-023-04329-1)
Supplement: Supplementary file 2 — Additional file 2: Table S1. Genomic positons of core simple sequence repeat (SSR) primers. Table S2. Basic information of 1021 sweet potato germplasm resources. Table S3. Genotypics of 1021 sweet potato germplasm resources. Table S4. Phenotypics of 1021 sweet potato germplasm resources. [file 12870_2023_4329_MOESM2_ESM.pdf]

Table S1. Genomic positons of core simple sequence repeat (SSR) primers

Primer name	chromosome	Start(bp)	end(bp)
GDAAS0338_F	LG9	5683601	5683583
GDAAS0338_R	LG9	5683436	5683457
GDAAS0694_F	LG7	2760913	2760932
GDAAS0694_R	LG7	2761048	2761066
GDAAS0782_F	LG11	7433110	7433131
GDAAS0782_R	LG11	7433207	7433228
GDAAS0819_F	na	na	na
GDAAS0819_R	na	na	na
GDAAS0871_F	LG15	5528101	5528120
GDAAS0871_R	LG15	5528244	5528261
GDAAS0911_F	LG11	1866110	1866127
GDAAS0911_R	LG11	1865976	1865997
GDAAS0922_F	LG13	23636854	23636874
GDAAS0922_R	LG13	23636681	23636701
GDAAS0940_F	LG5	664930	664950
GDAAS0940_R	LG5	665068	665085
SPGS2_F	LG12	2660628	2660647
SPGS2_R	LG12	2660355	2660375

Table S2. basic information of 1021 sweet potato germplasm resources.

Accession number	Accession name	Origin	Biological status
GN0001	Yan Zi Shu	Deqing,Guangdong,China	Landrace
GN0002	Gao Nong Xuan 113	Gaozhou,Guangdong,China	Improved cultivar
GN0005	Biao Xin Hong	Zhanjiang,Guangdong,China	Landrace
GN0007	Cheng Shu 68-9	Shantou,Guangdong,China	Improved cultivar
GN0008	Peng Wei	Zhanjiang,Guangdong,China	Landrace
GN0010	Bao Ting Zhong	Baoting,Hainan,China	Landrace
GN0012	Si Ji Zhong	Shantou,Guangdong,China	Landrace
GN0013	Bai Pi Xin Lai Mei	Zhanjiang,Guangdong,China	Landrace
GN0015	Sheng Mao Long	Haikang,Guangdong,China	Landrace
GN0018	Hong Xin Zai	Zhanjiang,Guangdong,China	Landrace
GN0019	Ya Zai Shu	Lianjiang,Guangdong,China	Landrace
GN0023	Zhou Nong 13	Haikou,Hainan,China	Improved cultivar
GN0025	Bu Lun Chun	Haikang,Guangdong,China	Landrace
GN0027	Dang Shu	Zhanjiang,Guangdong,China	Landrace
GN0029	Da Ye Po	Zhanjiang,Guangdong,China	Landrace
GN0030	Wu Shi Xian	Haikang,Guangdong,China	Landrace
GN0031	Liu Shi Ri	Xinxing,Guangdong,China	Landrace
GN0032	Gao Nong Xuan 3	Gaozhou,Guangdong,China	Improved cultivar
GN0033	NaN Ao Miao	Dongguan,Guangdong,China	Landrace
GN0037	Bai Pi Jie Yu Zhi	Jieyang,Guangdong,China	Landrace

GN0038	San Ya Miao	Gaozhou,Guangdong,China	Landrace
GN0039	Nan Ao Miao	Dongguan,Guangdong,China	Landrace
GN0040	Jin Lian Shu	Wenchang,Hainan,China	Landrace
GN0041	Mi Cao Lu Zai	Zhanjiang,Guangdong,China	Landrace
GN0043	Hong Po Ai Shu Li	Wuchuan,Guangdong,China	Landrace
GN0047	Xue Shu	Xinxing,Guangdong,China	Landrace
GN0049	Pan Shu Xiang	Foshan,Guangdong,China	Landrace
GN0050	Bai Pi Da Ye Hong	Zhanjiang,Guangdong,China	Landrace
GN0051	Da Ye Tian Jian Luo Li	Maoming,Guangdong,China	Landrace
GN0052	Bai Jin Long	Zhanjiang,Guangdong,China	Landrace
GN0056	Jia Shan You	Lingao,Hainan,China	Landrace
GN0059	Chi Pi Bu Lun Chun	Zhanjiang,Guangdong,China	Landrace
GN0062	Da Du	Qingyuan,Guangdong,China	Landrace
GN0064	Niao Zhong	Lufeng,Guangdong,China	Landrace
GN0069	Yu Bei Bai	Guangzhou,Guangdong,China	Landrace
GN0070	Ji Zhua Er	Zhanjiang,Guangdong,China	Landrace
GN0071	Zhu Tou	Shantou,Guangdong,China	Landrace
GN0075	Bai Ye Chang Le Miao	Xingning,Guangdong,China	Landrace
GN0080	Ai Lao Shu	Maoming,Guangdong,China	Landrace
GN0094	Ji Er Zai	Zhanjiang,Guangdong,China	Landrace
GN0099	Tie Li Zai	Zhanjiang,Guangdong,China	Landrace
GN0103	Hong Xin Gong	Wuchuan,Guangdong,China	Landrace
GN0106	Liu Shi Ri	Fengkai,Guangdong,China	Landrace
GN0107	Hong Teng	Deqing,Guangdong,China	Landrace
GN0111	Chuan Shu Hong	Danzhou,Hainan,China	Landrace
GN0113	Xiang Shu	Zhongshan,Guangdong,China	Landrace
GN0115	Hong Ya Wu	Zhanjiang,Guangdong,China	Landrace
GN0120	Lei Zhou Lan	Zhongshan,Guangdong,China	Landrace
GN0121	Pu Shu 6 Hao	Puning,Guangdong,China	Improved cultivar
GN0123	Niang Zai Zhong	Hainan,China	Landrace
GN0124	Hao Ming	Foshan,Guangdong,China	Landrace
GN0125	Xiao Zhong Peng Shu	Dongfang,Hainan,China	Landrace
GN0126	Bai You Ye Shu	Guangdong,China	Landrace
GN0128	Bang Shu	Hainan,China	Landrace
GN0130	Shan Tou Hong	Shantou,Guangdong,China	Landrace
GN0132	Sha Za 2 Hao	Shantou,Guangdong,China	Improved cultivar
GN0133	San Jiao Yu	Wenchang,Hainan,China	Landrace
GN0135	Ma Liu Jia	Malaysia	Introduced Resources
GN0137	Sha La Yue	Shantou,Guangdong,China	Landrace
GN0138	Luo Bai Shu	Luoding,Guangdong,China	Landrace
GN0139	Diao Si Hong	Maoming,Guangdong,China	Landrace
GN0144	Niao Yao Li	Lufeng,Guangdong,China	Landrace
GN0150	Jiu Cai Zhong	Shantou,Guangdong,China	Landrace
GN0151	Xie Xi Zhong	Dongguan,Guangdong,China	Landrace

GN0153	He Tian Shu	Longchuan,Guangdong,China	Landrace
GN0155	Chang Nian Qing	Wenchang,Hainan,China	Landrace
GN0156	Wu Gu Qi Long	Guangdong,China	Landrace
GN0157	Gao Nong Xuan Liu	Gaozhou,Guangdong,China	Improved cultivar
GN0158	Xi Nü Xiao	Zhanjiang,Guangdong,China	Landrace
GN0162	Ri Ben Shu	Hainan,China	Landrace
GN0168	Jie Yang Zhu Tou Hong	Jieyang,Guangdong,China	Landrace
GN0169	Weng Cai Zhong	Zhanjiang,Guangdong,China	Landrace
GN0170	Liu Shi Ri	Qiongzhong,Hainan,China	Landrace
GN0172	Xiang Gang Shu	Shenzhen,Guangdong,China	Landrace
GN0173	Xin Zhu Tou	Jieyang,Guangdong,China	Landrace
GN0179	Jin Shan Zhong	U.S.A	Introduced Resources
GN0180	Bai Mian Fen Shu	Zhanjiang,Guangdong,China	Landrace
GN0181	Ju Hua Zhong	Shantou,Guangdong,China	Landrace
GN0183	Xiang Fen Shu	Guangzhou,Guangdong,China	Landrace
GN0184	Hui Za 6 Hao	Huilai,Guangdong,China	Improved cultivar
GN0186	Yu Gou Zhong	Hainan,China	Landrace
GN0187	Meng Shu	Zhanjiang,Guangdong,China	Landrace
GN0191	Gu Niang Shu	Zhanjiang,Guangdong,China	Landrace
GN0192	Bing Lang Shu	Zhongshan,Guangdong,China	Landrace
GN0196	Guang Shu 76-4	Guangzhou,Guangdong,China	Improved cultivar
GN0197	Guang Shu 76-12	Guangzhou,Guangdong,China	Improved cultivar
GN0198	Guang Shu 7 Hao	Guangzhou,Guangdong,China	Improved cultivar
GN0199	Guang Shu 3 Hao	Guangzhou,Guangdong,China	Improved cultivar
GN0200	Za Zhong	Guangzhou,Guangdong,China	Improved cultivar
GN0201	Guang Shu 14 Hao	Guangzhou,Guangdong,China	Improved cultivar
GN0203	Guang Shu 15 Hao	Guangzhou,Guangdong,China	Improved cultivar
GN0205	Guang Shu 74-64	Guangzhou,Guangdong,China	Improved cultivar
GN0206	Guang Shu 73-21	Guangzhou,Guangdong,China	Improved cultivar
GN0207	Guang Shu 72-50	Guangzhou,Guangdong,China	Improved cultivar
GN0208	Guang Shu 75-48	Guangzhou,Guangdong,China	Improved cultivar
GN0209	Kang Han Zhong	Guangzhou,Guangdong,China	Improved cultivar
GN0210	Guang Shu 76-15	Guangzhou,Guangdong,China	Improved cultivar
GN0211	Guang Shu 73-38	Guangzhou,Guangdong,China	Improved cultivar
GN0212	Sui 75-5	Shuixi,Guangdong,China	Improved cultivar
GN0213	Guang Shu 75-83	Guangzhou,Guangdong,China	Improved cultivar
GN0214	Guang Shu 75-17	Guangzhou,Guangdong,China	Improved cultivar
GN0215	Guang Shu 75-90	Guangzhou,Guangdong,China	Improved cultivar
GN0216	Guang Shu 77-63	Guangzhou,Guangdong,China	Improved cultivar
GN0217	Yu Xuan Yi	Guangzhou,Guangdong,China	Improved cultivar
GN0218	Bai Pi Peng Wei	Zhanjiang,Guangdong,China	Landrace
GN0219	Hong Pi 60 Ri	Zhanjiang,Guangdong,China	Landrace
GN0224	Nong Zhong Xuan	Guangzhou,Guangdong,China	Improved cultivar
GN0257	Er Tong Tui	Zhanjiang,Guangdong,China	Landrace

GN0259	Hei Gu Zai	Zhanjiang,Guangdong,China	Landrace
GN0262	Shi Hui Gong	Zhanjiang,Guangdong,China	Landrace
GN0271	Zhi Chuang Mi	Gaozhou,Guangdong,China	Landrace
GN0272	Da Wei Hong	Gaozhou,Guangdong,China	Landrace
GN0284	Qing Pi Zhong	Maoming,Guangdong,China	Landrace
GN0292	Mian Bao Shu	Gaozhou,Guangdong,China	Landrace
GN0297	Zi Le Shu	Maoming,Guangdong,China	Landrace
GN0298	San Ya Zai	Gaozhou,Guangdong,China	Landrace
GN0299	Ying Ge Shu	Gaozhou,Guangdong,China	Landrace
GN0300	Ji Gua Huang	Zhanjiang,Guangdong,China	Landrace
GN0304	Guang Xi Shu	Huazhou,Guangdong,China	Landrace
GN0305	Zhu Tou Bai	Huazhou,Guangdong,China	Landrace
GN0307	Diao Si Huang	Huazhou,Guangdong,China	Landrace
GN0312	Cheng Tuo Shu	Huazhou,Guangdong,China	Landrace
GN0320	Fang Gui Gong	Maoming,Guangdong,China	Landrace
GN0324	Fen Shu	Maoming,Guangdong,China	Landrace
GN0326	Zhu Tou Luo	Maoming,Guangdong,China	Landrace
GN0351	Ji Dan Huang	Yangjiang,Guangdong,China	Landrace
GN0355	Ji Gua Miao	Yangchun,Guangdong,China	Landrace
GN0356	Na Yang Miao	Yangjiang,Guangdong,China	Landrace
GN0362	Huang Pi Zai	Yangjiang,Guangdong,China	Landrace
GN0363	Guang Xi Cai Ye	Guangxi,China	Landrace
GN0365	Xiang Shu	Taishan,Guangdong,China	Landrace
GN0374	Xun Nong 18	Xuwen,Guangdong,China	Breeding line
GN0375	Jiu Ri Shu	Guangdong,China	Landrace
GN0376	Liu Shi Ri Hong Xin	Xiamen,Fujian,China	Landrace
GN0380	Tai Nong 36 Hao	Taiwan,China	Improved cultivar
GN0381	Nong Lin 1 Hao	Japan	Introduced Resources
GN0382	Xiao Hong 70 Ri	Xiamen,Fujian,China	Landrace
GN0383	Yi Zi 138	Beijing,China	Improved cultivar
GN0388	Liu Shi Zai	Xiamen,Fujian,China	Landrace
GN0389	Ning 46	Nanjing,Jiangshu,China	Improved cultivar
GN0391	Long Yan 7-3	Longyan,Fujian,China	Improved cultivar
GN0392	Hu Nan 182	Changsha,Hunani,China	Improved cultivar
GN0396	Liao 249	Shenyang,Liaoning,China	Breeding line
GN0397	Hua Bei 169	Xuzhou,Jiangshu,China	Improved cultivar
GN0398	Huo Dong 51-93	Nanjing,Jiangshu,China	Improved cultivar
GN0399	Hu Nan 138	Changsha,Hunani,China	Improved cultivar
GN0400	Hua Bei 51-7-14	Beijing,China	Improved cultivar
GN0401	Cai Tou Bai	Xiamen,Fujian,China	Landrace
GN0402	Chao Zhou Ben	Guangdong,China	Landrace
GN0404	Huang Xin Zao	Guangdong,China	Landrace
GN0410	Wu Kui Hao	U.S.A	Introduced Resources
GN0411	Tai Nong 46	Taiwan,China	Improved cultivar

GN0414	Tai Nong 3 Hao	Taiwan,China	Improved cultivar
GN0415	He Bei 351	Hebei,China	Improved cultivar
GN0417	Hu Nan 183	Changsha,Hunani,China	Improved cultivar
GN0418	Tai Nong 47	Taiwan,China	Improved cultivar
GN0419	Da Hong Hua	Liuan,Anhui,China	Landrace
GN0420	Nei Yuan	Japan	Introduced Resources
GN0421	Tai Nong 9 Hao	Taiwan,China	Improved cultivar
GN0422	Yan Gao Tang	Longyan,Fujian,China	Improved cultivar
GN0423	Li Zi Xiang	Xuzhou,Jiangshu,China	Improved cultivar
GN0425	Bai Xing	U.S.A	Introduced Resources
GN0426	SOUTHEN QUEEN	U.S.A	Introduced Resources
GN0427	Nong Lin 3 Hao	Japan	Introduced Resources
GN0429	Nong Lin 4 Hao	Japan	Introduced Resources
GN0430	92878	Bulgaria	Introduced Resources
GN0433	25__3	Peru	Introduced Resources
GN0434	Hong Hong 1 Hao	Hangzhou,Zhejiang,China	Improved cultivar
GN0437	Bai Zhen Yang	Anhui,China	Landrace
GN0438	Xin Da Zi	Xuzhou,Jiangshu,China	Improved cultivar
GN0440	Nong Lin 2 Hao	Japan	Introduced Resources
GN0442	NANCY HALL	U.S.A	Introduced Resources
GN0446	Yuan Ji	North Korea	Introduced Resources
GN0449	Ao Mo Hong	U.S.A	Introduced Resources
GN0451	Mei 24	U.S.A	Introduced Resources
GN0453	Mei 116	U.S.A	Introduced Resources
GN0454	Mei 10	U.S.A	Introduced Resources
GN0460	Gao Zi 1 Hao	Yangling,Shanxi,China	Improved cultivar
GN0462	Jiang Xi Feng Sheng	Jiangshu,China	Landrace
GN0463	Da Nan You	Longyan,Fujian,China	Improved cultivar
GN0467	Niang Zai Zhong	Shantou,Guangdong,China	Landrace
GN0468	Ji Yin Hong	Hainan,China	Landrace
GN0469	Chi Niao	Wenchang,Hainan,China	Landrace
GN0470	Da Zhong Chang Nian Qing	Hainan,China	Landrace
GN0471	San Yue Bai	Qionghai,Hainan,China	Landrace
GN0473	Nan Feng Fen	Haikou,Hainan,China	Landrace
GN0479	Hong Teng Bai Xin	Guangdong,China	Landrace
GN0480	Jie Fang Shu	Jiangmen,Guangdong,China	Landrace
GN0489	Hong Gu Zai	Guangdong,China	Landrace
GN0503	Huang Jin Qian Guan	Japan	Introduced Resources
GN0505	Gao Xi 14	Japan	Introduced Resources
GN0512	Da Hong Pao	Guangdong,China	Landrace
GN0520	Sheng Li Bai Hao	Japan	Introduced Resources
GN0521	Tai Nong 10 Hao	Taiwan,China	Improved cultivar
GN0523	Mei Guo Hong	U.S.A	Introduced Resources
GN0527	Guang Shu 78-60	Guangzhou,Guangdong,China	Improved cultivar

GN0528	Guang Shu 78-45	Guangzhou,Guangdong,China	Improved cultivar
GN0529	Guang Shu 79-39	Guangzhou,Guangdong,China	Improved cultivar
GN0530	Guang Shu 79-15	Guangzhou,Guangdong,China	Improved cultivar
GN0538	Hu Guo	Japan	Introduced Resources
GN0542	Si Liang Miao	Guangdong,China	Landrace
GN0544	Xu Shu18	Xuzhou,Jiangshu,China	Improved cultivar
GN0545	Heng Jin	Taiwan,China	Landrace
GN0546	Xu Zhou 12-21	Xuzhou,Jiangshu,China	Improved cultivar
GN0547	Hong Tou 8 Hao	Hangzhou,Zhejiang,China	Improved cultivar
GN0551	Lu Hong 22 Hao	Lufeng,Guangdong,China	Improved cultivar
GN0553	Ji Long Xiang	Malaysia	Introduced Resources
GN0558	Tian Ban Fang	Guangdong,China	Landrace
GN0560	PR-S87	Philippines	Introduced Resources
GN0562	PR-S252	Philippines	Introduced Resources
GN0563	PR-S6	Philippines	Introduced Resources
GN0564	PR-S16-6	Philippines	Introduced Resources
GN0565	PR-S19-12	Philippines	Introduced Resources
GN0569	Xian Luo Hong	Thailand	Introduced Resources
GN0572	Heng Jin / Bai Pi Zhu Tou	Guangdong,China	Breeding line
GN0573	Si Ji Hong	Guangdong,China	Landrace
GN0578	Mei 1-110	U.S.A	Introduced Resources
GN0579	Mei 1-9	U.S.A	Introduced Resources
GN0580	Shan Shu 1 Hao	Shantou,Guangdong,China	Improved cultivar
GN0581	Shan Shu 3 Hao	Shantou,Guangdong,China	Improved cultivar
GN0582	Dong Fang 2 Hao	Chenghai,Guangdong,China	Improved cultivar
GN0585	Guang Shu 80-122	Guangzhou,Guangdong,China	Improved cultivar
GN0586	Guang Shu 80-159	Guangzhou,Guangdong,China	Improved cultivar
GN0587	Guang Shu 74-10	Guangzhou,Guangdong,China	Improved cultivar
GN0589	Zhan 59	Zhanjiang,Guangdong,China	Improved cultivar
GN0590	Zhan 73-165	Zhanjiang,Guangdong,China	Improved cultivar
GN0591	Zhan 75-57	Zhanjiang,Guangdong,China	Improved cultivar
GN0592	Zhan 64-285	Zhanjiang,Guangdong,China	Improved cultivar
GN0593	Zhan Xiao 74-21	Zhanjiang,Guangdong,China	Improved cultivar
GN0595	Tai Shu 81-5	Taishan,Guangdong,China	Improved cultivar
GN0597	Cheng Shu 8 Hao	Shantou,Guangdong,China	Improved cultivar
GN0602	Pu Shu 13 Hao	Puning,Guangdong,China	Improved cultivar
GN0605	Jie Shu 8 Hao	Jieyang,Guangdong,China	Improved cultivar
GN0606	He Ding Feng Huang Fu	Raoping,Guangdong,China	Landrace
GN0607	Ji Zhao Shu	Wuhua,Guangdong,China	Landrace
GN0608	Xue Lao Shu	Shantou,Guangdong,China	Landrace
GN0611	Nan Yang Shu	Wuhua,Guangdong,China	Landrace
GN0612	Duo Zai Niang	Wuhua,Guangdong,China	Landrace
GN0613	Weng Cai Zhong	Dapu,Guangdong,China	Landrace
GN0617	Dong Fang Hong	Fengshun,Guangdong,China	Landrace

GN0618	Wu Fan	Fengshun,Guangdong,China	Landrace
GN0619	Ni Fen Shu	Fengshun,Guangdong,China	Landrace
GN0620	Wu Jiao Xing	Baoting,Hainan,China	Landrace
GN0622	Ji Zu Zhao	Hainan,China	Landrace
GN0624	Feng Shou Hong	Dongfang,Hainan,China	Landrace
GN0630	Li Shu	Qiongzhong,Hainan,China	Landrace
GN0633	Man Lun	Dongfang,Hainan,China	Landrace
GN0636	Li Hong	Lingshui,Hainan,China	Landrace
GN0637	San Yue Bai	Qiongzhong,Hainan,China	Landrace
GN0638	Man Cheng Shu	Dongfang,Hainan,China	Landrace
GN0639	Hei Yan Shu	Qiongzhong,Hainan,China	Landrace
GN0641	Da Ye Shu	Qiongzhong,Hainan,China	Landrace
GN0643	Dan Zhou Shu	Shanya,Hainan,China	Landrace
GN0644	Hong Jing Zhong	Shanya,Hainan,China	Landrace
GN0646	Qi Cha Shu	Dongfang,Hainan,China	Landrace
GN0648	Tai Hong	Taishan,Guangdong,China	Landrace
GN0649	Dan Lan Shu	Taishan,Guangdong,China	Landrace
GN0650	Sheng Ma Shu	Taishan,Guangdong,China	Landrace
GN0651	Bai Pi Bu Lun Chun	Taishan,Guangdong,China	Landrace
GN0652	Tai Cheng Shu	Taishan,Guangdong,China	Landrace
GN0653	Da Ye Mu	Taishan,Guangdong,China	Landrace
GN0656	Chang Nian Peng	Haikou,Hainan,China	Landrace
GN0658	Wu Zhao Hong	Anding,Hainan,China	Landrace
GN0659	Chao Shan Bai	Wenchang,Hainan,China	Landrace
GN0661	Zhu Tou	Wenchang,Hainan,China	Landrace
GN0662	Bai Du Mian	Hainan,China	Landrace
GN0663	Huang Shu	Wenchang,Hainan,China	Landrace
GN0664	Zhan Jiang Bai	Haikou,Hainan,China	Landrace
GN0665	Xiao Ye Shu	Wenchang,Hainan,China	Landrace
GN0667	Wu Zhao Huang	Wenchang,Hainan,China	Landrace
GN0672	Wu Zhao Bai	Wenchang,Hainan,China	Landrace
GN0673	Ye Yuan Bo	Hainan,China	Landrace
GN0677	Xi Ye Shu	Wengyuan,Guangdong,China	Landrace
GN0678	Qing Teng Zai	Shaoguan,Guangdong,China	Landrace
GN0679	Cheng Tuo Shu	Ruyuan,Guangdong,China	Landrace
GN0680	Hong Xing Shu	Ruyuan,Guangdong,China	Landrace
GN0681	Mian Hua Zhong	Shantou,Guangdong,China	Landrace
GN0682	Ji Zhuo Zhong	Lufeng,Guangdong,China	Landrace
GN0683	Ban Ye Fu	Shantou,Guangdong,China	Landrace
GN0684	Sheng Song Shu	Chaoan,Guangdong,China	Landrace
GN0686	Chai Tou Zhong	Raoping,Guangdong,China	Landrace
GN0687	Lao Zhu Tou	Shantou,Guangdong,China	Landrace
GN0688	Ya Ma Ti	Jiexi,Guangdong,China	Landrace
GN0689	Ju Hua Zhong	Shantou,Guangdong,China	Landrace

GN0690	Pu Shu 0 Hao	Puning,Guangdong,China	Landrace
GN0691	Ba Xiang Huang	Chaoan,Guangdong,China	Landrace
GN0693	Liu Ye Que	Shantou,Guangdong,China	Landrace
GN0695	Zhu Zai Zhong	Shantou,Guangdong,China	Landrace
GN0696	Fan Zai Zhong	Jieyang,Guangdong,China	Landrace
GN0701	Bai Fang	Shantou,Guangdong,China	Landrace
GN0706	Ying Cai Zhong	Jiexi,Guangdong,China	Landrace
GN0707	Che Lu Zhong	Lufeng,Guangdong,China	Landrace
GN0709	Zhu Tou Zhong	Lufeng,Guangdong,China	Landrace
GN0711	Da Ji Zhao	Jiexi,Guangdong,China	Landrace
GN0713	Cun Teng	Shantou,Guangdong,China	Landrace
GN0714	Bian Dou Zhong	Shantou,Guangdong,China	Landrace
GN0717	Chu Pi Lian	Lufeng,Guangdong,China	Landrace
GN0718	Zi Rou	Shantou,Guangdong,China	Landrace
GN0724	Hei Gu Zai	Haikang,Guangdong,China	Landrace
GN0731	Hong Shu	Lianjiang,Guangdong,China	Landrace
GN0736	Gou Zhao Shu	Xinyi,Guangdong,China	Landrace
GN0740	Huang Xin Gao	Yangjiang,Guangdong,China	Landrace
GN0741	Da Lao Shu	Yangchun,Guangdong,China	Landrace
GN0742	Xiao Ye Lan Jiao Shu	Gaozhou,Guangdong,China	Landrace
GN0743	Shu Zai Miao	Yangjiang,Guangdong,China	Landrace
GN0744	Bai Pi Zhu Tou Hong	Yangjiang,Guangdong,China	Landrace
GN0745	Guang Xi Bai	Yangjiang,Guangdong,China	Landrace
GN0747	Qing Miao Zai	Yangjiang,Guangdong,China	Landrace
GN0749	Tie Si Shu	Zhanjiang,Guangdong,China	Landrace
GN0750	Si Lian Shu	Zhanjiang,Guangdong,China	Landrace
GN0754	Xin Fu Yin	Zhanjiang,Guangdong,China	Landrace
GN0756	Gao Zhou Shu	Maoming,Guangdong,China	Landrace
GN0757	Tong You Shu	Maoming,Guangdong,China	Landrace
GN0758	Liu Shi Ri Shu	Maoming,Guangdong,China	Landrace
GN0760	Hong Ji Mu shu	Maoming,Guangdong,China	Landrace
GN0761	Pu Shu 16 Hao	Puning,Guangdong,China	Improved cultivar
GN0762	Bai Pi Hong Xin Shu	Guangdong,China	Landrace
GN0763	Huang Pi 9 Hao	Zhejiang,China	Improved cultivar
GN0764	OPK	U.S.A	Introduced Resources
GN0766	Nong Da Hong	Beijing,China	Improved cultivar
GN0767	Xiu Yu 1 Hao	Suqian,Jiangshu,China	Improved cultivar
GN0770	Carver	U.S.A	Introduced Resources
GN0773	Golden belle	U.S.A	Introduced Resources
GN0774	Gcorgia Red	U.S.A	Introduced Resources
GN0775	Red cliff	U.S.A	Introduced Resources
GN0777	Nema gold	U.S.A	Introduced Resources
GN0778	Acadian	U.S.A	Introduced Resources
GN0783	Daja	Philippines	Introduced Resources

GN0786	UPLB-7	Philippines	Introduced Resources
GN0789	Visca 8	Philippines	Introduced Resources
GN0793	Bakabakahan	Philippines	Introduced Resources
GN0794	Cugunen	Philippines	Introduced Resources
GN0796	Norinnigo	Philippines	Introduced Resources
GN0797	HOK-HOK-TOT	Philippines	Introduced Resources
GN0798	Taiwan 63	Taiwan,China	Improved cultivar
GN0800	Kacahne	Philippines	Introduced Resources
GN0802	Taiwan 471	Taiwan,China	Improved cultivar
GN0804	Colo	Philippines	Introduced Resources
GN0806	Benguet	Philippines	Introduced Resources
GN0807	Pangil	Philippines	Introduced Resources
GN0811	Copper Skin Gold Rush	Philippines	Introduced Resources
GN0812	Kabiti	Philippines	Introduced Resources
GN0813	LC-Taiwan	Philippines	Introduced Resources
GN0814	UPR-Variaga	Philippines	Introduced Resources
GN0815	Nortnnigo	Philippines	Introduced Resources
GN0816	Bauo-osagod	Philippines	Introduced Resources
GN0817	Zhong Guo 28	Japan	Introduced Resources
GN0819	L-4-5	U.S.A	Introduced Resources
GN0820	W178	U.S.A	Introduced Resources
GN0822	Ao Zhou Huang	U.S.A	Introduced Resources
GN0823	W51	U.S.A	Introduced Resources
GN0824	Tai Shan 2 Hao	U.S.A	Introduced Resources
GN0825	W4	U.S.A	Introduced Resources
GN0827	Tai Nong 3 Hao	Taiwan,China	Improved cultivar
GN0828	Ji Nan Hong	Jinan,Shandong,China	Improved cultivar
GN0830	Huo Bei 52-45	Xuzhou,Jiangshu,China	Improved cultivar
GN0834	Guang Shu 79-92	Guangzhou,Guangdong,China	Improved cultivar
GN0835	Guang Shu 79-93	Guangzhou,Guangdong,China	Improved cultivar
GN0837	Guang Shu 80-24	Guangzhou,Guangdong,China	Improved cultivar
GN0839	Wu Zhao Shu	Guangdong,China	Landrace
GN0840	60 Ri Zao	Guangdong,China	Landrace
GN0844	Bai Pi Da Ye Hong	Guangdong,China	Landrace
GN0845	Hai Bai	Guangdong,China	Landrace
GN0847	Ri Ben Hong	Guangdong,China	Landrace
GN0849	Hong Wei Ma	Guangdong,China	Landrace
GN0852	Nen Nu Shu	Guangdong,China	Landrace
GN0853	Shuang Zai	Guangdong,China	Landrace
GN0854	Feng Shu 2 Hao	Fengshun,Guangdong,China	Improved cultivar
GN0856	Pu Shu 221	Puning,Guangdong,China	Improved cultivar
GN0858	Tai Yin 31	Thailand	Introduced Resources
GN0860	Guang Shu 80-61	Guangzhou,Guangdong,China	Improved cultivar
GN0864	Nan Shu 48	Haikou,Hainan,China	Improved cultivar

GN0865	Guang Shu 128	Guangzhou,Guangdong,China	Improved cultivar
GN0865	Guang Shu 182	Guangzhou,Guangdong,China	Improved cultivar
GN0866	Guang Shu 82-68	Guangzhou,Guangdong,China	Improved cultivar
GN0867	Shao Shu 78-1	Shaoguan,Guangdong,China	Improved cultivar
GN0868	Guang Shu 79-130	Guangzhou,Guangdong,China	Improved cultivar
GN0870	Guang Shu 82-149	Guangzhou,Guangdong,China	Improved cultivar
GN0872	Zhan 83-26	Zhanjiang,Guangdong,China	Improved cultivar
GN0874	Guang Shu 84-143	Guangzhou,Guangdong,China	Improved cultivar
GN0875	Ungbuay	Thailand	Introduced Resources
GN0877	Xi Meng 1 Hao	Brazil	Introduced Resources
GN0882	Huang Pi Niao Li	Lufeng,Guangdong,China	Landrace
GN0884	Long Yan 8-6	Longyan,Fujian,China	Improved cultivar
GN0885	Pu Shu 53	Putian,Fujian,China	Improved cultivar
GN0886	Fu Shu 87	Fuzhou,Fujian,China	Improved cultivar
GN0888	Xing Zhong Hua	Aanxi,Fujian,China	Improved cultivar
GN0889	Jin Shu 70-13	Fujian,China	Breeding line
GN0891	Tai Yin 2 Hao	Thailand	Introduced Resources
GN0895	Tai Yin 6 Hao	Thailand	Introduced Resources
GN0900	Tai Yin 11 Hao	Thailand	Introduced Resources
GN0902	Tai Yin 13 Hao	Thailand	Introduced Resources
GN0905	Tai Yin 16 Hao	Thailand	Introduced Resources
GN0908	Tai Yin 19 Hao	Thailand	Introduced Resources
GN0909	Tai Yin 20 Hao	Thailand	Introduced Resources
GN0910	Pe79	Thailand	Introduced Resources
GN0913	CN942-47	Thailand	Introduced Resources
GN0914	CI412	Thailand	Introduced Resources
GN0916	I166	Thailand	Introduced Resources
GN0917	Yong Chun Wu Chi	Yongchun,Fujian,China	Landrace
GN0919	Huang Zai Men	Jinjiang,Fujian,China	Landrace
GN0923	Liu Shi Ri Zao	Yongtai,Fujian,China	Landrace
GN0925	Man Cun Xiang	Nanning,Guangxi,China	Landrace
GN0926	Wu You Ji	Guangxi,China	Landrace
GN0928	Wu Li Xiang	Guangxi,China	Landrace
GN0931	Guang Shu 84-79	Guangzhou,Guangdong,China	Improved cultivar
GN0932	Guang Shu 85-61	Guangzhou,Guangdong,China	Improved cultivar
GN0933	Pu Shu 19	Puning,Guangdong,China	Improved cultivar
GN0934	Pu Shu 84-6	Puning,Guangdong,China	Improved cultivar
GN0935	Pu Shu 81-111	Puning,Guangdong,China	Improved cultivar
GN0936	Pu Shu 10 Hao	Puning,Guangdong,China	Improved cultivar
GN0944	DJ6-31	Japan	Introduced Resources
GN0945	Jiu Xi 17-3028	Japan	Introduced Resources
GN0946	Nong Lin 5 Hao	Japan	Introduced Resources
GN0947	Nong Lin 40 Hao	Japan	Introduced Resources
GN0948	Jiu Zhou 62	Japan	Introduced Resources

GN0949	Si Bei Ti-1	Japan	Introduced Resources
GN0950	Nong Lin 38 Hao	Japan	Introduced Resources
GN0952	Si Chuan 36	Japan	Introduced Resources
GN0954	Yen 1392	Japan	Introduced Resources
GN0956	FV 62-64	Japan	Introduced Resources
GN0957	Miao Bu Zhi	Japan	Introduced Resources
GN0959	Nong Lin 36	Japan	Introduced Resources
GN0963	Nong Lin 37	Japan	Introduced Resources
GN0965	Jiu Zhou 107	Japan	Introduced Resources
GN0972	Di Gua Nuo	Zhangpu,Fujian,China	Landrace
GN0973	Bing Lang Shu	Putian,Fujian,China	Landrace
GN0974	Teng Zai	Jinjiang,Fujian,China	Landrace
GN0975	Hu Tou Ben	Aanxi,Fujian,China	Landrace
GN0976	Te Bie Shu	Xiapu,Fujian,China	Landrace
GN0977	Gou Tou Shu	Fujian,China	Landrace
GN0978	Hong Wei Shu	Yongchun,Fujian,China	Landrace
GN0979	Jue Fen Fan Shu	Changting,Fujian,China	Landrace
GN0980	Tie Xian Shu	Fuding,Fujian,China	Landrace
GN0982	Huang Shu	Fujian,China	Landrace
GN0984	Jiao Tong Zhong	Zhangpu,Fujian,China	Landrace
GN0985	Dian Ping Shu	Jinjiang,Fujian,China	Landrace
GN0986	Shu Fan Gang Shu Yu	Jinjiang,Fujian,China	Landrace
GN0987	Mu dong Gua	Fuding,Fujian,China	Landrace
GN0990	Zhu Xi Ben	Xianyou,Fujian,China	Landrace
GN0991	De Hua Zao Shu	Fujian,China	Landrace
GN0992	Jie Hong	Jinjiang,Fujian,China	Landrace
GN0994	Tan Zuan	Changle,Fujian,China	Landrace
GN0995	Hai Quan Shu	Yuling,Guangxi,China	Landrace
GN1000	Jin Gua Nang	Fangchenggang,Guangxi,China	Landrace
GN1002	Liu Shi Shu	Luchuan,Guangxi,China	Landrace
GN1004	Nan Gua Shu	Guanyang,Guangxi,China	Landrace
GN1005	Pa Man Shu	Xicheng,Guangxi,China	Landrace
GN1006	Hong Xin Shu	Luchuan,Guangxi,China	Landrace
GN1007	Bai Shu	Hechi,Guangxi,China	Landrace
GN1010	Hua Xin Shu	Nanning,Guangxi,China	Landrace
GN1011	Si Bu Jin	Yuling,Guangxi,China	Landrace
GN1013	Wu Si Nan	Guanyang,Guangxi,China	Landrace
GN1014	Liu Shi Ri	Fangchenggang,Guangxi,China	Landrace
GN1016	Gui Shu 82-134	Nanning,Guangxi,China	Breeding line
GN1018	ZH85-18	Zhanjiang,Guangdong,China	Improved cultivar
GN1019	Guang Shu 85-29	Guangzhou,Guangdong,China	Improved cultivar
GN1020	Guang Shu 62	Guangzhou,Guangdong,China	Improved cultivar
GN1022	P85-3	Guangzhou,Guangdong,China	Improved cultivar
GN1026	Huang Lue Shu	Zhanjiang,Guangdong,China	Landrace

GN1027	Xi Tou Shu	Yangjiang,Guangdong,China	Landrace
GN1028	Guang Shu 86-8	Guangzhou,Guangdong,China	Improved cultivar
GN1029	Guang Shu 86-78	Guangzhou,Guangdong,China	Improved cultivar
GN1032	Guang Shu 86-82	Guangzhou,Guangdong,China	Improved cultivar
GN1035	Guang Shu 111	Guangzhou,Guangdong,China	Improved cultivar
GN1038	Yu 83-538	Zhengzhou,Henan,China	Improved cultivar
GN1039	P85-44	Guangzhou,Guangdong,China	Improved cultivar
GN1040	Xiang 6	Changsha,Hunani,China	Improved cultivar
GN1043	Pu 83-91	Puning,Guangdong,China	Improved cultivar
GN1044	Pei 26	Fujian,China	Breeding line
GN1045	TIS9101	Nigeria	Introduced Resources
GN1046	Tib11	Nigeria	Introduced Resources
GN1047	TIS3290	Nigeria	Introduced Resources
GN1048	Tib9	Nigeria	Introduced Resources
GN1049	TIS70357	Nigeria	Introduced Resources
GN1050	TIS5125	Nigeria	Introduced Resources
GN1053	TIS8401	Nigeria	Introduced Resources
GN1055	P85-49	Nigeria	Introduced Resources
GN1056	Zhan 85-38	Zhanjiang,Guangdong,China	Improved cultivar
GN1057	CN1232-9	Thailand	Introduced Resources
GN1058	CN1038-16	Thailand	Introduced Resources
GN1061	Pu 81-113	Puning,Guangdong,China	Improved cultivar
GN1063	Pan Yu Yuan Ye	Guangzhou,Guangdong,China	Landrace
GN1064	Pan Yu Da Bai	Guangzhou,Guangdong,China	Landrace
GN1065	Pan Yu Que Ye	Guangzhou,Guangdong,China	Landrace
GN1066	Guang Shu 87-17	Guangzhou,Guangdong,China	Improved cultivar
GN1068	Guang Shu 87-21	Guangzhou,Guangdong,China	Improved cultivar
GN1069	Guang Shu 87-47	Guangzhou,Guangdong,China	Improved cultivar
GN1070	Guang Shu 87-48	Guangzhou,Guangdong,China	Improved cultivar
GN1073	Guang Shu 87-76	Guangzhou,Guangdong,China	Improved cultivar
GN1074	Guang Shu 87-78	Guangzhou,Guangdong,China	Improved cultivar
GN1075	Guang Shu 87-8-1	Guangzhou,Guangdong,China	Improved cultivar
GN1078	Guang Shu 86-71	Guangzhou,Guangdong,China	Improved cultivar
GN1079	Xiang Guang Yin	Hongkong,China	Landrace
GN1080	Tai Wan Shu	Taiwan,China	Landrace
GN1086	Guang Shu 88-114	Guangzhou,Guangdong,China	Improved cultivar
GN1087	Yu Lan Zhong	Jiexi,Guangdong,China	Landrace
GN1089	Guang Shu 85-66	Guangzhou,Guangdong,China	Improved cultivar
GN1091	CN1656-37	Peru	Genetic stocks
GN1092	Guang Shu 88-70	Guangzhou,Guangdong,China	Improved cultivar
GN1094	NPSP-545	Philippines	Introduced Resources
GN1097	Sieteflores	Philippines	Introduced Resources
GN1098	NPSP-608	Philippines	Introduced Resources
GN1104	Su Shu 1 Hao	Nanjing,Jiangshu,China	Improved cultivar

GN1106	Nan Shu 88	Nanchong,Sichuan,China	Improved cultivar
GN1107	Yang Hong Shao	Chongqing,China	Landrace
GN1110	Fu Jian Hong Shu	Fujian,China	Landrace
GN1111	Ya Jiao Ba Fan	Fujian,China	Landrace
GN1116	Xiang Zhong	Shantou,Guangdong,China	Landrace
GN1119	Guang Shu 88-26	Guangzhou,Guangdong,China	Improved cultivar
GN1120	Guang Shu 88-43	Guangzhou,Guangdong,China	Improved cultivar
GN1130	Zhi Wu Yuan Yin	Guangzhou,Guangdong,China	Landrace
GN1131	Guang Shu 89-42	Guangzhou,Guangdong,China	Improved cultivar
GN1132	Guang Shu 87-66	Guangzhou,Guangdong,China	Improved cultivar
GN1134	Guang Shu 89-66	Guangzhou,Guangdong,China	Improved cultivar
GN1135	Guang Shu 90-99	Guangzhou,Guangdong,China	Improved cultivar
GN1136	Jin 3101	Shanxi,China	Improved cultivar
GN1138	Guang Shu 90-79	Guangzhou,Guangdong,China	Improved cultivar
GN1139	Guang Shu 90-85	Guangzhou,Guangdong,China	Improved cultivar
GN1140	VS-4ul	Peru	Genetic stocks
GN1141	Yan Fen 1 Hao	Fengshun,Guangdong,China	Improved cultivar
GN1142	440036ul	Peru	Introduced Resources
GN1143	Waef	Peru	Introduced Resources
GN1144	L-9	Peru	Introduced Resources
GN1145	CIP-Jewel	Peru	Introduced Resources
GN1146	BANS-19	Peru	Introduced Resources
GN1149	Huang Pi Zi Rou Shu	Foshan,Guangdong,China	Landrace
GN1150	Guang Shu 91-2	Guangzhou,Guangdong,China	Improved cultivar
GN1153	Guang Shu 91-17	Guangzhou,Guangdong,China	Improved cultivar
GN1157	Guang Shu 91-116	Guangzhou,Guangdong,China	Improved cultivar
GN1158	Zhan 89-10	Guangzhou,Guangdong,China	Improved cultivar
GN1162	Hai 3	Hainan,China	Landrace
GN1163	Jia Shan You	Hainan,China	Landrace
GN1164	Chuan Yin	Hainan,China	Landrace
GN1165	Huang Pi 9 Hao	Hainan,China	Landrace
GN1167	Mu Dong	Hainan,China	Landrace
GN1168	Nan 32	Haikou,Hainan,China	Improved cultivar
GN1169	Wu Zhao Bai	Hainan,China	Landrace
GN1171	Weng Cai Zhong	Hainan,China	Landrace
GN1174	Hong Gu/Biao Ri	Haikou,Hainan,China	Breeding line
GN1177	Ri Ben Shu	Hainan,China	Landrace
GN1178	Yue Jin Shu	Hainan,China	Landrace
GN1186	Guang Shu 91-46	Guangzhou,Guangdong,China	Improved cultivar
GN1187	Guang Shu 92-97	Guangzhou,Guangdong,China	Improved cultivar
GN1188	Guang Shu 92-12	Guangzhou,Guangdong,China	Improved cultivar
GN1189	Guang Shu 92-45	Guangzhou,Guangdong,China	Improved cultivar
GN1191	Guang Shu 92-26	Guangzhou,Guangdong,China	Improved cultivar
GN1195	Guang Shu 93-77	Guangzhou,Guangdong,China	Improved cultivar

GN1196	Guang Shu 93-85	Guangzhou,Guangdong,China	Improved cultivar
GN1198	Guang Shu 93-36	Guangzhou,Guangdong,China	Improved cultivar
GN1199	Tai Ru	Fujian,China	Improved cultivar
GN1200	Xia Yin 1 Hao	U.S.A	Introduced Resources
GN1201	Chao 93-30	Shantou,Guangdong,China	Improved cultivar
GN1202	Guang Shu 93-68	Guangzhou,Guangdong,China	Improved cultivar
GN1204	Guang Shu 92-93	Guangzhou,Guangdong,China	Improved cultivar
GN1205	Guang Shu 92-85	Guangzhou,Guangdong,China	Improved cultivar
GN1206	Guang Shu 93-7	Guangzhou,Guangdong,China	Improved cultivar
GN1208	Guang Shu 94-21	Guangzhou,Guangdong,China	Improved cultivar
GN1209	Guang Shu 95-29	Guangzhou,Guangdong,China	Improved cultivar
GN1210	Jie Yang Shi Pai Hong	Jieyang,Guangdong,China	Landrace
GN1211	Guang Shu 94-4	Guangzhou,Guangdong,China	Improved cultivar
GN1215	Beauregard	U.S.A	Introduced Resources
GN1217	Guang Shu 92-56	Guangzhou,Guangdong,China	Improved cultivar
GN1218	Guang Shu 92-66	Guangzhou,Guangdong,China	Improved cultivar
GN1219	Guang Shu 95-145	Guangzhou,Guangdong,China	Improved cultivar
GN1222	Hernadez	U.S.A	Introduced Resources
GN1223	HiDry	U.S.A	Introduced Resources
GN1225	Sumor	U.S.A	Introduced Resources
GN1227	Pu Shu 23	Puning,Guangdong,China	Improved cultivar
GN1228	Zhan 93-16	Zhanjiang,Guangdong,China	Improved cultivar
GN1229	Guang Shu 95-1	Guangzhou,Guangdong,China	Improved cultivar
GN1230	Chao Shu 18	Chaoan,Guangdong,China	Improved cultivar
GN1232	Hou Long Hong	Shantou,Guangdong,China	Landrace
GN1233	Chao Zhou	Shantou,Guangdong,China	Landrace
GN1234	Yan Shu 5 Hao	Longyan,Fujian,China	Improved cultivar
GN1237	Yan Shu 251	Yantai,Shandong,China	Improved cultivar
GN1239	Yan Shu 27	Yantai,Shandong,China	Improved cultivar
GN1245	AB940078-1	Peru	Introduced Resources
GN1246	AB94001-8	Peru	Introduced Resources
GN1249	CH1232-9	Xuzhou,Jiangshu,China	Improved cultivar
GN1250	Y-6	Xuzhou,Jiangshu,China	Wild
GN1251	Y-2	Xuzhou,Jiangshu,China	Wild
GN1252	Jin Shan 908	Fuzhou,Fujian,China	Improved cultivar
GN1253	Jin Shan 1885	Fuzhou,Fujian,China	Improved cultivar
GN1257	Ning 180	Nanjing,Jiangshu,China	Improved cultivar
GN1258	Lu Shu 2 Hao	Yantai,Shandong,China	Improved cultivar
GN1259	Lu Shu 3 Hao	Yantai,Shandong,China	Improved cultivar
GN1262	W10-1	Jiangshu,China	Wild
GN1263	Zi K-3	Jiangshu,China	Improved cultivar
GN1264	7K-2	Jiangshu,China	Improved cultivar
GN1266	W3-2	Jiangshu,China	Wild
GN1269	Su Shu 0 Hao	Jiangshu,China	Improved cultivar

GN1270	KW7-1	Jiangshu,China	Wild
GN1273	Ning 12-17	Nanjing,Jiangshu,China	Improved cultivar
GN1276	Guang Shu 96-13	Guangzhou,Guangdong,China	Improved cultivar
GN1277	Guang Shu 96-31	Guangzhou,Guangdong,China	Improved cultivar
GN1278	Shang 52-7	Shangqiu,Henan,China	Improved cultivar
GN1279	Bai Pi Hong Xin	Jiangshu,China	Landrace
GN1280	Su Shu 4 Hao	Nanjing,Jiangshu,China	Improved cultivar
GN1281	Xiang Fen Hong Pi	Jiangshu,China	Improved cultivar
GN1282	Nan Jing 88-10	Nanjing,Jiangshu,China	Improved cultivar
GN1283	"9503"	Jiangshu,China	Improved cultivar
GN1284	E Shu 3 Hao	Wuhan,Hubei,China	Improved cultivar
GN1285	Hong Sa Mo	Japan	Introduced Resources
GN1286	Hong Shou	Japan	Introduced Resources
GN1287	Jin Shan 25	Fuzhou,Fujian,China	Improved cultivar
GN1288	Gui Shu 93-35	Nanning,Guangxi,China	Improved cultivar
GN1289	Jin Shan 57	Fuzhou,Fujian,China	Improved cultivar
GN1290	Quan Shu 23	Quanzhou,Fujian,China	Improved cultivar
GN1292	Pu Shu 94-118	Puning,Guangdong,China	Improved cultivar
GN1293	Pu Shu 94-24	Puning,Guangdong,China	Improved cultivar
GN1295	Guang Shu 97-136	Guangzhou,Guangdong,China	Improved cultivar
GN1296	Guang Shu 97-96	Guangzhou,Guangdong,China	Improved cultivar
GN1297	Guang Shu 69	Guangzhou,Guangdong,China	Improved cultivar
GN1300	Zhan Jian Cai Ye	Zhanjiang,Guangdong,China	Landrace
GN1304	Jin Shan 1193	Fuzhou,Fujian,China	Improved cultivar
GN1306	Guang Shu 98-19	Guangzhou,Guangdong,China	Improved cultivar
GN1308	Nan Chong 9014-3	Nanchong,Sichuan,China	Improved cultivar
GN1311	Guang Shu 94-84	Guangzhou,Guangdong,China	Improved cultivar
GN1312	Guang Shu 97-40	Guangzhou,Guangdong,China	Improved cultivar
GN1315	Xu 98-22-23	Xuzhou,Jiangshu,China	Improved cultivar
GN1316	Kang bing Du Zhong	Xuzhou,Jiangshu,China	Genetic stocks
GN1317	Pu Ning Zhong	Puning,Guangdong,China	Landrace
GN1318	Rao Ping Zhong	Raoping,Guangdong,China	Landrace
GN1319	Guang Shu 99-28	Guangzhou,Guangdong,China	Improved cultivar
GN1321	Guang Shu 99-68	Guangzhou,Guangdong,China	Improved cultivar
GN1323	Pu Zi	Taiwan,China	Improved cultivar
GN1325	Long Shu 1 Hao	Longyan,Fujian,China	Improved cultivar
GN1327	Tai Yin 01	Taiwan,China	Genetic stocks
GN1329	Hai Yang Zhong	Guangzhou,Guangdong,China	Landrace
GN1331	Zhen Nong Ke	Zhejiang,China	Genetic stocks
GN1333	Guang Shu 97	Guangzhou,Guangdong,China	Improved cultivar
GN1334	Mei Ying 1 Hao	Zhengyang,Henan,China	Improved cultivar
GN1336	Guang Shu 2K-29	Guangzhou,Guangdong,China	Improved cultivar
GN1337	Guang Shu 2K-30	Guangzhou,Guangdong,China	Improved cultivar
GN1338	Ning Zi 1 Hao	Nanjing,Jiangshu,China	Improved cultivar

GN1339	Xu Zi Shu 1 Hao	Xuzhou,Jiangshu,China	Improved cultivar
GN1340	Fu Jian 60 Ri	Fujian,China	Landrace
GN1342	Nan Fu	Fujian,China	Landrace
GN1345	Chang Ting Dong Jie Zhong	Fujian,China	Landrace
GN1346	Chan Man Zhong	Fujian,China	Landrace
GN1347	Yu Bei Hong	Fujian,China	Landrace
GN1348	Da Tou Huang	Fujian,China	Landrace
GN1349	Bei Jiao	Fujian,China	Landrace
GN1350	Gou Tou Hong	Fujian,China	Landrace
GN1351	Luo Bo Shu	Fujian,China	Landrace
GN1352	AymuraSaki	Japan	Introduced Resources
GN1353	Tan Sang Ni Ya	Tanzania	Introduced Resources
GN1354	Guang Shu 98-83	Guangzhou,Guangdong,China	Improved cultivar
GN1355	Guang Zi Shu 1 Hao	Guangzhou,Guangdong,China	Improved cultivar
GN1356	Cong Hua Zhong	Guangzhou,Guangdong,China	Landrace
GN1358	Guang Shu 98-54	Guangzhou,Guangdong,China	Improved cultivar
GN1359	Pei Si	Fujian,China	Landrace
GN1360	Guang Yuan	Fujian,China	Landrace
GN1361	Te Bie Shu	Fujian,China	Landrace
GN1363	Wu Ping Nong Jia Zhong-4	Fujian,China	Landrace
GN1364	Wu Ping Nong Jia Zhong-5	Fujian,China	Landrace
GN1366	Man Shan Hong	Fujian,China	Landrace
GN1367	Huang Shu	Guangdong,China	Landrace
GN1368	Niao Yao Li	Guangdong,China	Landrace
GN1376	Shu Yuan	Guangdong,China	Landrace
GN1377	Liu Shi Ri	Hainan,China	Landrace
GN1381	San Jiao Ning	Hainan,China	Landrace
GN1384	Hong Pi Huang Du	Hainan,China	Landrace
GN1385	Xian Gou Shu	Hainan,China	Landrace
GN1394	Zhan Jiang Cai	Zhanjiang,Guangdong,China	Landrace
GN1396	Yan 7-3	Longyan,Fujian,China	Improved cultivar
GN1397	Fu Shu 7-6	Fuzhou,Fujian,China	Improved cultivar
GN1398	Wan Shu 8 Hao	Fuyang,Anhui,China	Improved cultivar
GN1403	Ri Ben Zi	Japan	Introduced Resources
GN1405	Guang 27	Guangzhou,Guangdong,China	Improved cultivar
GN1406	Guang Shu 95	Guangzhou,Guangdong,China	Improved cultivar
GN1407	Guang Shu 98	Guangzhou,Guangdong,China	Improved cultivar
GN1408	Guang Shu 99-78	Guangzhou,Guangdong,China	Improved cultivar
GN1413	Mu Gua Shu	Heng,Guangxi,China	Landrace
GN1414	Zhang Teng	Chongqing,China	Landrace
GN1415	Wu Jian Tiao	Chongqing,China	Landrace
GN1416	Tai Wan Peng	Taiwan,China	Landrace
GN1419	Hai shi	U.S.A	Introduced Resources
GN1421	Nong Lin 19	Japan	Introduced Resources

GN1422	PR-S16-6	Philippines	Introduced Resources
GN1423	PR-S252	Philippines	Introduced Resources
GN1424	Ning Zi Shu 1 HaoP-4	Nanjing,Jiangshu,China	Improved cultivar
GN1426	Tai Nong 71	Taiwan,China	Improved cultivar
GN1428	Chuan Cai Shu 17	Chengdu,Sichuan,China	Improved cultivar
GN1429	Quan Shu 830	Quanzhou,Fujian,China	Improved cultivar
GN1430	Yan Zi Shu 337	Yantai,Shandong,China	Improved cultivar
GN1431	Ji Shu 18	Jinan,Shandong,China	Improved cultivar
GN1433	Ji Shu 22-41	Shijiazhuang,Hebei,China	Improved cultivar
GN1434	Zi 311	Wuhan,Hubei,China	Improved cultivar
GN1437	Yang Chun Shu	Yangchun,Guangdong,China	Landrace
GN1438	PI318846-3	Peru	Introduced Resources
GN1440	Jewel	Peru	Introduced Resources
GN1442	Zhua Bai Zhong	Guangzhou,Guangdong,China	Landrace
GN1443	Huang Ding Cheng	Guangzhou,Guangdong,China	Landrace
GN1444	Sha Geng Tiao	Santai,Sichuan,China	Landrace
GN1445	Tai Wan Qiu	Taiwan,China	Landrace
GN1446	W-4	Jinan,Shandong,China	Wild
GN1448	Guang Shu 2K-66	Guangzhou,Guangdong,China	Improved cultivar
GN1450	Guang Shu 2K-25	Guangzhou,Guangdong,China	Improved cultivar
GN1453	Chao 98-38	Shantou,Guangdong,China	Improved cultivar
GN1454	Fu Shu 5 Hao	Fuzhou,Fujian,China	Improved cultivar
GN1455	Gui Shu 96-8	Nanning,Guangxi,China	Improved cultivar
GN1456	Jin Shan 291	Fuzhou,Fujian,China	Improved cultivar
GN1457	Pu Shu 25	Puning,Guangdong,China	Improved cultivar
GN1458	Pu 99-116	Puning,Guangdong,China	Improved cultivar
GN1460	Zhan 99-9	Zhanjiang,Guangdong,China	Improved cultivar
GN1461	Ri Ben Shu	Hainan,China	Landrace
GN1462	Da Ye Hong Xin	Hainan,China	Landrace
GN1467	Chi Ke Shu	Hainan,China	Landrace
GN1468	Hong Gu Bei Gan Er	Hainan,China	Landrace
GN1471	Hei Gu	Hainan,China	Landrace
GN1472	Hai Bei Gan Shu	Hainan,China	Landrace
GN1473	Shi Liu Shu	Hainan,China	Landrace
GN1474	Hei Xin Gan	Hainan,China	Landrace
GN1476	Da Tian Gen	Hainan,China	Landrace
GN1478	Ba Xiang Huang	Hainan,China	Landrace
GN1479	Jin Gua Huang	Hainan,China	Landrace
GN1480	Mu Dong	Hainan,China	Landrace
GN1481	Ku Liang Hong	Hainan,China	Landrace
GN1482	Chun Mian	Hainan,China	Landrace
GN1485	Dou Yun Hong Pi	Guizhou,China	Landrace
GN1491	Gui Ding He Hong Pi Hong Xin	Guizhou,China	Landrace

GN1492	Gui Zhou Bai Pi Shao	Guizhou,China	Landrace
GN1493	Kai Li Hong Xin Shao	Guizhou,China	Landrace
GN1495	Du Shan Zi Hua Xin	Guizhou,China	Landrace
GN1497	Yin Jiang Tu Huang Shao	Guizhou,China	Landrace
GN1501	An Long Bai Pi Hong Xin	Guizhou,China	Landrace
GN1503	Tong Ren Bai Pi Hong Xin	Guizhou,China	Landrace
GN1504	Shi Qian Zi Pi Hong Xin	Guizhou,China	Landrace
GN1505	Gui Zhou Zi Hong Pi Huang Xin	Guizhou,China	Landrace
GN1509	Meng Zi Yang Hong	Guizhou,China	Landrace
GN1512	Meng Zi Huang Xin	Yunnan,China	Landrace
GN1513	Jian Shui Huang Xin	Yunnan,China	Landrace
GN1515	Pu Er Da Bai	Yunnan,China	Landrace
GN1516	Pu Er Shui Guo Shan Yu	Yunnan,China	Landrace
GN1517	Jing Hong Hong Pi	Yunnan,China	Landrace
GN1518	Pu Er Huang Shan Yu	Yunnan,China	Landrace
GN1519	Hua Bei Za Jiao	Yunnan,China	Landrace
GN1520	Meng La Hong Pi	Yunnan,China	Landrace
GN1521	Meng La Zi Hong Pi	Yunnan,China	Landrace
GN1522	Ying Jiang Xiao Ye Bai Pi	Yunnan,China	Landrace
GN1523	Bao Shan Xiao Huang Shan Yu	Yunnan,China	Landrace
GN1524	Ying Jiang Hong Pi	Yunnan,China	Landrace
GN1525	Bai Shui Guo Shan Yao	Yunnan,China	Landrace
GN1526	Ying Jiang Huang Bai Xin	Yunnan,China	Landrace
GN1527	Mang Shi Hong Pi	Yunnan,China	Landrace
GN1530	Ying Jiang Zi Hong Pi Bai Xin	Yunnan,China	Landrace
GN1531	Ying Jiang Zi Pi	Yunnan,China	Landrace
GN1532	Rui Li Zi Hong Pi	Yunnan,China	Landrace
GN1533	Kun Ming Gan Xin Hong Shu	Yunnan,China	Landrace
GN1536	Lv 06	Hefei,Anhui,China	Improved cultivar
GN1538	Lv Shu 17	Hefei,Anhui,China	Improved cultivar
GN1540	Zhe 132	Hangzhou,Zhejiang,China	Improved cultivar
GN1541	Zhe 255	Hangzhou,Zhejiang,China	Improved cultivar
GN1542	Zhe 3481	Hangzhou,Zhejiang,China	Improved cultivar
GN1543	Zhe Shu 13	Hangzhou,Zhejiang,China	Improved cultivar
GN1545	Wen Lai Shu	Brunei	Introduced Resources
GN1546	Shan Tou Nong Jia Zhong	Shantou,Guangdong,China	Landrace
GN1548	V140	U.S.A	Introduced Resources
GN1550	Long Shu 10 Hao	Longyan,Fujian,China	Improved cultivar
GN1551	Yang Jiang Hong Kao Shu	Yangjiang,Guangdong,China	Landrace
GN1552	Cong Hua Tai Wan Cai Ye	Guangzhou,Guangdong,China	Landrace
GN1553	Yun Nan San Xin Zhong	Yunnan,China	Landrace
GN1554	Qi Cai Shu	Yangxi,Guangdong,China	Landrace
GN1555	Jin Guan Huang	Yangxi,Guangdong,China	Landrace

GN1557	Yang Xi Shu	Yangxi,Guangdong,China	Landrace
GN1562	Bai Zi Gao	Maoming,Guangdong,China	Landrace
GN1564	Xiao Zi Bai	Maoming,Guangdong,China	Landrace
GN1566	Zi Huang Dong	Yangjiang,Guangdong,China	Landrace
GN1571	Cai Yong	Hangzhou,Zhejiang,China	Improved cultivar
GN1572	Hong Ju Zi	Guangzhou,Guangdong,China	Landrace
GN1573	Zi Bai Jiao	Guangzhou,Guangdong,China	Landrace
GN1576	Guang Shu 01-84	Guangzhou,Guangdong,China	Improved cultivar
GN1577	Guang Cai Shu 14	Guangzhou,Guangdong,China	Improved cultivar
GN1578	Fei Lu Bin Hong Pi	Philippines	Introduced Resources
GN1579	LC-6	Philippines	Introduced Resources
GN1580	Ji Long Shu	Malaysia	Introduced Resources
GN1581	Lu Feng Ji Dan Huang 1	Lufeng,Guangdong,China	Landrace
GN1582	Guang Shu Cai 1 Hao	Guangzhou,Guangdong,China	Improved cultivar
GN1583	Guang Shu 87	Guangzhou,Guangdong,China	Improved cultivar
GN1584	Guang Shu 79	Guangzhou,Guangdong,China	Improved cultivar
GN1585	Guang Shu Cai 2 Hao	Guangzhou,Guangdong,China	Improved cultivar
GN1587	Guang Zi Shu 2 Hao	Guangzhou,Guangdong,China	Improved cultivar
GN1588	TA2	Japan	Introduced Resources
GN1589	A3	Japan	Introduced Resources
GN1590	Fu Shu 13	Fuzhou,Fujian,China	Improved cultivar
GN1591	Ba Tou Hai Bian Zhong	Shantou,Guangdong,China	Landrace
GN1592	Xin Long Cai Zhong	Shantou,Guangdong,China	Landrace
GN1593	Xin Hua	Shantou,Guangdong,China	Landrace
GN1594	Lian Shang Hai Bian Jiu Cai	Shantou,Guangdong,China	Landrace
GN1596	Lian Xia Nan Fen Zhong	Shantou,Guangdong,China	Landrace
GN1598	Fu Shu 18	Fuzhou,Fujian,China	Improved cultivar
GN1602	Xiang Yu Shu	Huidong,Guangdong,China	Landrace
GN1603	Gao Zhou Fen Xiang Shu	Gaozhou,Guangdong,China	Landrace
GN1604	Wei Duo Li	Shijiazhuang,Hebei,China	Improved cultivar
GN1606	Nan Cheng Zhong	Haikou,Hainan,China	Landrace
GN1607	Lian Shang Zhu Tou	Shantou,Guangdong,China	Landrace
GN1611	Huang Pi Shu	Zhanjiang,Guangdong,China	Landrace
GN1615	Nan Ao Zhong	Shantou,Guangdong,China	Landrace
GN1618	Yue Nan Zi	Vietnam	Introduced Resources
GN1619	Gan Bu Zhong	Shantou,Guangdong,China	Landrace
GN1622	Tian E Shu	Zhanjiang,Guangdong,China	Landrace
GN1623	Hong Gao Li	Longmen,Guangdong,China	Landrace
GN1624	Guang Shu 03-118	Guangzhou,Guangdong,China	Improved cultivar
GN1625	Yue Nan Zi Luo Lan	Vietnam	Introduced Resources
GN1626	Shang Ying Chi Pi Lian	Shanwei,Guangdong,China	Landrace
GN1627	Zi Xin Hong Shu	Qiannanzhou,Guizhou,China	Landrace
GN1629	Hai Nan Huang	Haikou,Hainan,China	Landrace
GN1630	Quan Shu 9 Hao	Quanzhou,Fujian,China	Breeding line

GN1632	Da Shu Zi Zhong	Vietnam	Introduced Resources
GN1633	Guang Zi Shu 3 Hao	Guangzhou,Guangdong,China	Breeding line
GN1634	Nan Ping Lv Jing	Zhanjiang,Guangdong,China	Landrace
GN1636	Dong Hai Shu	Zhanjiang,Guangdong,China	Landrace
GN1637	Da Zhai Hong	Guangzhou,Guangdong,China	Landrace
GN1639	Guang Shu 08-236	Guangzhou,Guangdong,China	Breeding line
GN1640	Guang Shu 11-83	Guangzhou,Guangdong,China	Breeding line
GN1661	Mian Zi Shu 9 Hao	Mianyang,Sichuan,China	Improved cultivar
A232	Yang Jiang Qiu Shu	Yangjiang,Guangdong,China	Landrace
A236	Long Jing Wei	Longmen,Guangdong,China	Landrace
A237	Jia Gong Pin Zhong	Guangzhou,Guangdong,China	Landrace
A240	Nan Shu Fan	Enping,Guangdong,China	Landrace
A242	Guang Shu 01-24	Guangzhou,Guangdong,China	Improved cultivar
A247	Chong Hua Chi Shu	Guangzhou,Guangdong,China	Landrace
A252	Wan Shu 31 Hao	Hefei,Anhui,China	Improved cultivar
A263	A5	Japan	Introduced Resources
A271	Xing Ning Fen Shu	Xingning,Guangdong,China	Landrace
A276	Xu 02S20-1	Xuzhou,Jiangshu,China	Improved cultivar
A278	Zheng Qun Zi 1 Hao	Zhengzhou,Henan,China	Improved cultivar
A280	B1364-4	Nanjing,Jiangshu,China	Genetic stocks
A284	Xu Suo Shu	Xuwen,Guangdong,China	Improved cultivar
A331	Xu Shu 22	Xuzhou,Jiangshu,China	Improved cultivar
A346	Ji Shu 22	Jinan,Shandong,China	Improved cultivar
A348	Shu 273	Xuzhou,Jiangshu,China	Improved cultivar
A354	Yu 0146-30	-	Improved cultivar
A369	Ning 17-6	Nanjing,Jiangshu,China	Improved cultivar
A377	Shu Shu 12	Nanjing,Jiangshu,China	Improved cultivar
A393	Da Li Shu	Dali,Yunnan,China	Landrace
A397	Huang Pi Zhong	Jinan,Shandong,China	Landrace
A400	Chang Zi Shu	Haikou,Hainan,China	Landrace
A402	Nong Yuan Zhong	Guangzhou,Guangdong,China	Landrace
A403	Xiang 75-55	Hunani,China	Improved cultivar
A421	Jia Xi Zhong 2	Shanwei,Guangdong,China	Landrace
A422	Jia Xi Zhong	Shanwei,Guangdong,China	Landrace
A423	Xiao Mao Zhong	Shanwei,Guangdong,China	Landrace
A429	Chi Pi Lian	Shanwei,Guangdong,China	Landrace
A430	Shan Shu 710	Shanwei,Guangdong,China	Improved cultivar
A442	Guang Zi Shu 5 Hao	Guangzhou,Guangdong,China	Breeding line
A445	Guang Shu 06-89	Guangzhou,Guangdong,China	Breeding line
A447	Guang Shu 146	Guangzhou,Guangdong,China	Improved cultivar
A455	Ning 16-2	Nanjing,Jiangshu,China	Breeding line
A457	Ning 43-8	Nanjing,Jiangshu,China	Breeding line
A468	Hong Gu Zai	Zhanjiang,Guangdong,China	Landrace
A476	Fu Cheng Zi Jing Shu	Zhanjiang,Guangdong,China	Landrace

A478	Ji Dan Huang	Zhanjiang,Guangdong,China	Landrace
A493	Ning Zhong 408	Jiangshu,China	Improved cultivar
A494	Si Ji Hong	-	Landrace
A508	Jiu Li Xiang	Yangjiang,Guangdong,China	Landrace
A517	Xin Yu Mian Shu	Yangjiang,Guangdong,China	Landrace
A521	Jin Li Shu	Yangjiang,Guangdong,China	Landrace
A523	Shai Bu Wei Shu	Yangjiang,Guangdong,China	Landrace
A534	Sha Xi Shu	Jiangmen,Guangdong,China	Landrace
A540	Lie Ye Zi Rou Shu		Landrace
A542	Xiao Ma Zi Shu	Jiangmen,Guangdong,China	Landrace
A552	Ri Ben Zi Luo Lan	Japan	Introduced Resources
A555	Hong Rou Shu	Huizhou,Guangdong,China	Landrace
A557	Ao Tou Shu	Huizhou,Guangdong,China	Landrace
A562	Feng Zu Shu	Huizhou,Guangdong,China	Landrace
A567	3 Wen	-	Breeding line
A570	Huang Jin Niu		Landrace
A571	Bai Yu Jiu Li Xiang		Landrace
A579	Pan Yu Ji Gu Xiang	Panyu,Guangdong,China	Landrace
A587	Jin Shan 679	Fuzhou,Fujian,China	Breeding line
A588	Shen Ji	-	Breeding line
A589	Quan 76	Quanzhou,Fujian,China	Breeding line
A591	03-165	Guangzhou,Guangdong,China	Breeding line
A597	Ze Zi Shu 1 Hao	Zhejiang,China	Improved cultivar
A604	Wan Xi 56	Chongqing,China	Breeding line
A605	Long Yan 8-6	Longyan,Fujian,China	Breeding line
A610	Long Yan 7-3	Longyan,Fujian,China	Breeding line
A620	Gui 96-8	Nanning,Guangxi,China	Improved cultivar
A621	Gui Fen 1 Hao	Nanning,Guangxi,China	Improved cultivar
A625	Ning Cai	-	Breeding line
A628	EC04	-	Breeding line
A631	Guang Shu 155	Guangzhou,Guangdong,China	Improved cultivar
A634	Zhan Jiang Zhen Zhu Hong	Zhanjiang,Guangdong,China	Landrace
A638	Long Shu 14	Longyan,Fujian,China	Improved cultivar
A639	Pu Shu 16 Hao	Putian,Fujian,China	Improved cultivar
A640	Guang Shu 42	Guangzhou,Guangdong,China	Improved cultivar
A641	Quan Shu 10 Hao	Quanzhou,Fujian,China	Improved cultivar
A646	Guang Zi Shu 7 Hao	Guangzhou,Guangdong,China	Improved cultivar
A647	Pu Shu 32	Puning,Guangdong,China	Improved cultivar
A652	Zheng Guo 1		Breeding line
A653	Zheng Guo 2		Breeding line
A654	Zheng Guo 3		Breeding line
A659	Wan Shu 5 Hao	Chongqing,China	Improved cultivar
A660	Wan Shu 6 Hao	Chongqing,China	Improved cultivar
A662	Huang Yang	-	Landrace

A664	Qing Shui 2 Hao		Breeding line
A665	Quang Shu 12 Hao	Quanzhou,Fujian,China	Breeding line
A666	Quang Shu 17 Hao	Quanzhou,Fujian,China	Breeding line
A668	Fang Cheng Gang 1 Hao	Guangxi,China	Breeding line
A669	Fang Cheng Gang 2 Hao	Guangxi,China	Breeding line
A672	Gui Fen 2 Hao	Guangxi,China	Landrace
A674	Ping Nan Yin	Guangxi,China	Landrace
A681	Long Shu 7-3	Longyan,Fujian,China	Improved cultivar
A683	Long Shu 9 Hao	Longyan,Fujian,China	Improved cultivar
A689	Long Shu 21 Hao	Longyan,Fujian,China	Improved cultivar
A690	Long Shu 24 Hao	Longyan,Fujian,China	Improved cultivar
A691	Long Shu 28 Hao	Longyan,Fujian,China	Improved cultivar
A694	Q201	Guangzhou,Guangdong,China	Genetic stocks
A698	Ning Cai F18-1	Nanjing,Jiangshu,China	Breeding line
A699	Guang Cai 5 Hao	Guangzhou,Guangdong,China	Improved cultivar
A700	Ji Dan Huang		Landrace
A701	Jiu Li Xiang		Landrace
A702	Yi Dian Hong		Landrace
A703	Hei Gui		Landrace
A704	Shuang Sheng Huang		Landrace
A706	Hai Nan Huang		Landrace
A709	onlean	Baton Rouge,U.S.A	Introduced Resources
A712	Shuang Pi Hong	Gaozhou,Guangdong,China	Landrace
A713	Pu Zi Shu 3 Hao	Putian,Fujian,China	Improved cultivar
A715	Fu 24	Fuzhou,Fujian,China	Improved cultivar
A716	Guang Zi Shu 8 Hao	Guangzhou,Guangdong,China	Improved cultivar
A717	Guang Shu 08-6	Guangzhou,Guangdong,China	Improved cultivar
A718	Guang Shu 25	Guangzhou,Guangdong,China	Improved cultivar
A719	Guang Shu 82	Guangzhou,Guangdong,China	Improved cultivar
A722	Xiang Yu Shu		Improved cultivar
A724	CN591-51	Peru	Introduced Resources
A726	105086.1 INIA308,ARNE	Peru	Introduced Resources
A729	106212.1	Peru	Introduced Resources
A731	106445.2 5773	Peru	Introduced Resources
A737	106539.1 6887	Peru	Introduced Resources
A742	106734.1 8824	Peru	Introduced Resources
A744	106861.3	Peru	Introduced Resources
A746	187004.2 LM87.045	Peru	Introduced Resources
A748	187017.1 SALYBORO	Peru	Introduced Resources
A749	188002.1 SR88.029	Peru	Introduced Resources
A751	190023.43 SR90301	CIP	Introduced Resources
A752	194281.2 LM94.167	Peru	Introduced Resources
A753	195037 SR95002/40060 coloroda	CIP	Introduced Resources

A757	199062.1/420001 paramongui.NaraniaOsouro	CIP	Introduced Resources
A759	400039 10-C-1 unknown	Peru	Introduced Resources
A760	420607 Trujiuano	CIP	Introduced Resources
A764	421781 Rompe Costel	Peru	Introduced Resources
A765	CIP 422546 Huambacho	Peru	Introduced Resources
A768	40011 w-216	CIP	Introduced Resources
A769	440185 L0-323	Peru	Introduced Resources
A770	440088	CIP	Introduced Resources
A772	440396 BNAS White	Peru	Introduced Resources
A779	Yan Shu 24	Yantai,Shandong,China	Improved cultivar
A781	Yan Shu 25	Yantai,Shandong,China	Improved cultivar
A782	Shang Shu 19	Shangqiu,Henan,China	Improved cultivar
A783	Luo Shu 11 Hao	Luoyang,Henan,China	Improved cultivar
A784	Ji Shu 23	Jinan,Shandong,China	Improved cultivar
A785	Ji Shu 21-1	Jinan,Shandong,China	Improved cultivar
A787	Zheng Hong 25	Zhengzhou,Henan,China	Breeding line
A790	Ji Hei 1 Hao	Jinan,Shandong,China	Improved cultivar
A792	Su Shu 9 Hao	Nanjing,Jiangshu,China	Improved cultivar
A793	Shang Shu 12	Shangqiu,Henan,China	Improved cultivar
A794	Luo Shu 12	Luoyang,Henan,China	Improved cultivar
A795	Xu Shu 22	Xuzhou,Jiangshu,China	Improved cultivar
A796	Shang Shu 11	Shangqiu,Henan,China	Improved cultivar
A798	Mian Zi Shu 9 Hao	Mianyang,Sichuan,China	Improved cultivar
A799	Shang Shu 9 Hao	Shangqiu,Henan,China	Improved cultivar
A800	Su Shu 16	Nanjing,Jiangshu,China	Improved cultivar
A801	Xu Zi 12	Xuzhou,Jiangshu,China	Improved cultivar
A803	Ji Shu 25-1	Jinan,Shandong,China	Breeding line
A803	Ji Shu 25	Jinan,Shandong,China	Breeding line
A806	Xu Zi Shu 3 Hao	Xuzhou,Jiangshu,China	Improved cultivar
A808	Wan Su 58	Hefei,Anhui,China	Breeding line
A813	BC-1-5	Guangzhou,Guangdong,China	Genetic stocks
A814	BC-1-8	Guangzhou,Guangdong,China	Genetic stocks
A815	BC-1-10	Guangzhou,Guangdong,China	Genetic stocks
A816	TBN-1	Guangzhou,Guangdong,China	Genetic stocks
A817	TBH-2	Guangzhou,Guangdong,China	Genetic stocks
A819	TBB-1	Guangzhou,Guangdong,China	Genetic stocks
A821	Huang Jin Qian Guan	Kagoshima,Japan	Introduced Resources
A824	E2017		Breeding line
A827	Cai Shu 9-15-13		Breeding line
A828	Zhan Shu 407	Zhanjiang,Guangdong,China	Breeding line
A829	Ji Shu 17	Jinan,Shandong,China	Improved cultivar
A830	Shang Shu 29Z1-1	Shangqiu,Henan,China	Breeding line
A832	Shang Xu Z30-1	Shangqiu,Henan,China	Breeding line

A833	Su Shu 22	Nanjing,Jiangshu,China	Improved cultivar
A834	E3588		Breeding line
A835	Xu Yu 35	Xuzhou,Jiangshu,China	Improved cultivar
A836	Pu Shu 28	Puning,Guangdong,China	Improved cultivar
A839	Luo Xu 8 Hao	Luohe,Henan,China	Improved cultivar
A840	Zhan Shu 93-16	Zhanjiang,Guangdong,China	Improved cultivar
A842	Ji Shu 982	Shijiazhuang,Hebei,China	Improved cultivar
A843	Wan Zi Shu 56	Chongqing,China	Improved cultivar
A845	Zhan Shu 01-2	Zhanjiang,Guangdong,China	Improved cultivar
A847	Yan Zi Shu 3 Hao	Yantai,Shandong,China	Improved cultivar
A849	evangeline	U.S.A	Introduced Resources
A854	Hai Nan Gong Si Zi 1 Hao	Hainan,China	Breeding line
A855	Hai Nan Gong Si 2 Hao	Hainan,China	Breeding line
A856	Fu Cai Shu 23	Fuzhou,Fujian,China	Improved cultivar
A859	Guang Shu 08-237	Guangzhou,Guangdong,China	Improved cultivar
A863	Zou Tu Bian Ti		Others
A869	San Se Gan Shu		Others
A888	Nong Da Bai	Beijing,China	Improved cultivar
A889	Huang Mei Gui		Landrace
A890	Huang Xiang Jiao		Landrace
A905	08-236	Guangzhou,Guangdong,China	Breeding line
A906	You 11-139	Guangzhou,Guangdong,China	Breeding line
A907	Guang Cai Shu 6 Hao	Guangzhou,Guangdong,China	Improved cultivar
A908	Guang Cai Shu 7 Hao	Guangzhou,Guangdong,China	Improved cultivar
A909	Cai 11-52	Guangzhou,Guangdong,China	Breeding line
A910	Cai 17-10	Guangzhou,Guangdong,China	Breeding line
A912	Zi 12-007	Guangzhou,Guangdong,China	Breeding line
A913	Hu 12-044	Guangzhou,Guangdong,China	Breeding line
A914	Guang Zi Shu 9 Hao	Guangzhou,Guangdong,China	Improved cultivar
A915	Guang Zi Shu 10 Hao	Guangzhou,Guangdong,China	Improved cultivar
A917	Q197	Guangzhou,Guangdong,China	Breeding line
A918	Guang Shu 72-50	Guangzhou,Guangdong,China	Improved cultivar
A968	Ba Xin 1 Hao	Papua New Guinea	Introduced Resources
A969	Ba Xin 2 Hao	Papua New Guinea	Introduced Resources
A970	Ba Xin 3 Hao	Papua New Guinea	Introduced Resources
A971	Ba Xin 4 Hao	Papua New Guinea	Introduced Resources
A997	Guang Shu 14-76	Guangzhou,Guangdong,China	Breeding line

Table S3. Genotypics of 1021 sweet potato germplasm resources.

Accession number	Genotypic								
	A	B	C	D	E	F	G	H	I
GN0001	80	25	41	57	6	55	21	58	101
GN0002	102	13	79	56	7	266	70	58	101
GN0005	106	25	75	11	28	241	125	56	99

GN0007	88	13	85	60	17	243	144	56	112
GN0008	88	17	81	27	28	146	31	48	120
GN0010	85	27	36	27	17	304	120	36	56
GN0012	103	17	90	14	17	274	3	33	120
GN0013	91	13	91	57	17	282	133	56	99
GN0015	89	13	95	40	17	270	123	56	48
GN0018	93	17	91	61	17	134	18	46	99
GN0019	99	17	78	57	28	34	120	62	117
GN0023	92	17	81	11	28	271	176	46	120
GN0025	86	17	81	32	17	155	26	50	117
GN0027	91	13	95	23	17	237	28	53	41
GN0029	100	24	78	61	28	291	159	40	57
GN0030	89	13	95	40	17	264	123	56	56
GN0031	85	16	76	57	17	226	125	58	-
GN0032	99	13	79	45	28	18	120	40	99
GN0033	78	17	36	23	17	147	62	35	118
GN0037	106	17	68	18	17	291	23	51	99
GN0038	86	17	81	32	17	155	144	58	120
GN0039	106	27	80	21	17	286	57	52	117
GN0040	92	11	31	60	22	247	116	56	26
GN0041	109	17	51	32	28	303	44	56	54
GN0043	96	13	36	61	17	297	106	50	99
GN0047	109	13	76	46	28	19	20	29	117
GN0049	90	13	79	64	28	270	25	33	101
GN0050	99	24	78	61	28	291	159	40	80
GN0051	109	17	19	61	17	296	19	11	99
GN0052	86	27	81	27	17	288	29	50	117
GN0056	66	13	87	56	28	218	129	36	120
GN0059	99	17	87	40	17	206	46	33	67
GN0062	93	13	95	40	17	270	123	56	48
GN0064	101	17	81	41	17	265	125	40	101
GN0069	91	13	91	57	17	282	133	56	117
GN0070	106	13	76	41	28	151	103	58	9
GN0071	93	13	95	40	17	264	123	44	48
GN0075	91	17	19	40	17	263	176	56	37
GN0080	106	17	79	57	28	245	54	58	42
GN0094	100	17	92	57	17	29	110	36	99
GN0099	100	17	95	11	22	286	140	56	101
GN0103	100	17	61	59	17	175	118	56	101
GN0106	93	17	91	61	17	134	116	58	101
GN0107	46	17	7	57	22	100	138	36	99
GN0111	88	25	80	27	17	296	24	50	101
GN0113	93	17	12	16	28	288	19	56	56
GN0115	100	17	96	56	17	236	120	58	56

GN0120	100	17	87	42	17	214	119	28	45
GN0121	91	17	81	57	17	237	131	50	56
GN0123	101	13	95	42	28	288	159	50	111
GN0124	111	11	98	23	28	285	129	56	99
GN0125	66	13	95	41	17	304	31	39	56
GN0126	100	17	95	42	22	288	178	33	101
GN0128	86	17	27	23	25	111	137	48	50
GN0130	90	13	62	30	17	223	129	57	54
GN0132	106	25	41	23	17	243	126	60	99
GN0133	86	17	95	42	17	288	178	50	117
GN0135	99	17	92	41	17	221	149	58	120
GN0137	86	17	95	59	25	13	109	56	56
GN0138	91	13	91	57	25	282	133	56	117
GN0139	99	17	87	40	17	206	46	33	40
GN0144	108	17	57	47	17	291	70	50	117
GN0150	106	17	69	16	28	290	124	51	45
GN0151	106	17	69	16	28	290	124	51	40
GN0153	90	17	69	61	17	86	22	36	101
GN0155	85	25	52	60	17	267	137	58	91
GN0156	88	13	81	27	17	287	129	48	117
GN0157	106	25	50	56	17	223	32	48	99
GN0158	100	17	87	18	22	180	112	33	104
GN0162	100	17	87	18	22	180	112	26	104
GN0168	96	25	69	61	17	279	125	52	117
GN0169	96	27	92	56	28	50	137	34	92
GN0170	99	16	95	49	17	286	178	34	120
GN0172	99	16	95	49	17	286	178	34	101
GN0173	86	13	62	61	17	262	29	50	117
GN0179	107	17	86	57	25	202	155	58	120
GN0180	109	17	68	23	17	252	146	61	99
GN0181	109	17	68	23	17	252	146	51	99
GN0183	106	13	56	42	28	217	120	50	54
GN0184	109	13	81	60	17	282	137	50	101
GN0186	106	13	76	14	28	288	149	50	120
GN0187	106	13	76	14	28	288	149	58	120
GN0191	66	16	80	46	28	303	45	58	111
GN0192	100	17	87	42	17	214	119	38	40
GN0196	92	13	84	60	17	137	84	50	117
GN0197	106	27	98	29	25	42	126	36	99
GN0198	87	13	79	64	28	270	30	33	101
GN0199	99	17	56	61	22	186	126	58	99
GN0200	7	13	30	42	17	301	19	36	99
GN0201	68	13	30	42	17	301	19	31	99
GN0203	96	16	32	57	17	288	64	32	99

GN0205	87	13	20	65	25	173	94	34	44
GN0206	101	13	30	57	17	85	100	36	99
GN0207	103	17	36	56	17	164	89	36	120
GN0208	99	13	87	56	17	39	140	48	101
GN0209	103	17	98	64	17	247	89	36	120
GN0210	100	17	81	61	6	287	139	33	101
GN0211	70	17	87	56	17	77	140	58	15
GN0212	107	17	82	20	25	302	73	58	120
GN0213	101	17	36	56	17	164	89	36	120
GN0214	96	13	75	63	17	231	127	52	117
GN0215	107	17	50	56	17	73	131	36	117
GN0216	107	17	50	56	17	73	131	36	111
GN0217	91	13	92	46	6	284	93	40	56
GN0218	100	17	87	18	22	180	112	33	101
GN0219	102	13	59	65	17	284	25	42	117
GN0224	102	13	59	65	17	284	137	42	117
GN0257	88	25	80	27	17	296	127	58	101
GN0259	92	13	84	60	17	137	17	58	117
GN0262	96	17	95	57	17	282	129	58	40
GN0271	106	13	98	57	17	212	149	62	38
GN0272	107	17	59	40	17	287	75	49	104
GN0284	92	17	76	61	17	296	139	22	99
GN0292	88	17	50	57	17	243	137	55	117
GN0297	15	13	76	41	17	153	103	58	91
GN0298	109	13	81	42	25	247	109	56	42
GN0299	100	17	62	40	15	97	139	34	101
GN0300	106	25	85	56	17	15	120	48	120
GN0304	91	17	11	57	28	288	24	38	117
GN0305	90	17	17	15	17	155	70	36	117
GN0307	105	13	81	42	28	65	34	36	120
GN0312	99	13	31	40	17	296	137	58	54
GN0320	101	17	81	56	17	296	71	58	101
GN0324	100	17	78	57	28	36	170	33	99
GN0326	100	17	62	40	23	97	139	34	101
GN0351	101	17	81	57	25	75	89	58	111
GN0355	86	17	81	27	28	287	25	56	101
GN0356	100	13	14	65	17	265	137	58	54
GN0362	99	17	79	57	17	284	82	56	92
GN0363	102	13	80	56	28	255	137	58	117
GN0365	99	17	81	27	25	166	84	36	99
GN0374	100	17	-	57	28	36	170	11	99
GN0375	106	13	80	23	17	226	128	36	111
GN0376	90	27	90	17	17	264	169	58	120
GN0380	66	25	80	60	17	67	132	56	75

GN0381	66	25	80	60	17	67	132	48	50
GN0382	66	25	80	60	17	67	132	48	38
GN0383	66	25	87	56	17	256	175	61	12
GN0388	106	27	92	23	17	79	81	50	117
GN0389	92	17	87	10	17	279	70	58	120
GN0391	91	13	92	46	6	286	93	40	40
GN0392	8	17	92	57	17	135	161	57	108
GN0396	11	25	80	60	2	67	162	56	72
GN0397	11	25	80	60	3	67	132	56	64
GN0398	12	25	80	60	2	67	126	56	67
GN0399	66	25	80	26	3	67	165	56	35
GN0400	101	17	81	57	25	75	8	58	61
GN0401	106	27	92	23	3	3	7	58	65
GN0402	66	25	80	60	2	67	165	56	64
GN0404	86	13	18	61	17	296	117	36	99
GN0410	106	17	92	15	16	184	84	36	101
GN0411	51	25	95	45	7	68	128	36	78
GN0414	66	25	79	27	17	146	144	58	99
GN0415	66	25	80	60	17	67	132	56	78
GN0417	51	25	80	60	3	67	126	56	78
GN0418	53	13	80	22	7	167	97	60	5
GN0419	51	25	80	60	2	67	126	56	78
GN0420	37	13	98	11	17	203	70	62	101
GN0421	42	17	61	35	2	175	122	56	67
GN0422	51	13	92	34	7	219	149	55	78
GN0423	88	11	80	27	17	296	129	58	66
GN0425	56	25	41	3	7	243	106	60	100
GN0426	42	17	62	40	4	97	118	34	101
GN0427	60	13	80	22	25	198	149	57	76
GN0429	42	17	61	59	7	175	122	56	101
GN0430	51	13	95	41	17	304	137	39	82
GN0433	28	25	42	42	3	140	120	56	99
GN0434	56	13	61	14	17	113	106	36	82
GN0437	106	25	76	61	25	296	84	36	101
GN0438	74	25	87	45	10	283	144	60	6
GN0440	21	17	60	44	5	193	157	56	101
GN0442	93	25	87	45	10	283	137	60	26
GN0446	81	-	79	14	-	204	140	40	120
GN0449	99	13	87	10	30	254	140	58	99
GN0451	87	17	98	45	17	219	128	58	117
GN0453	93	17	12	16	28	288	125	56	82
GN0454	23	13	87	26	3	109	76	56	80
GN0460	61	17	55	40	17	282	113	58	93
GN0462	33	17	60	44	15	193	164	56	101

GN0463	92	13	79	46	17	213	137	36	117
GN0467	85	25	52	60	17	267	110	58	91
GN0468	96	12	98	56	26	9	140	36	117
GN0469	96	25	76	45	17	279	129	62	120
GN0470	49	17	91	59	16	295	109	39	80
GN0471	40	13	30	42	17	301	125	36	40
GN0473	100	17	87	42	17	214	119	38	66
GN0479	42	13	76	46	7	22	120	38	80
GN0480	56	17	95	57	17	291	129	58	50
GN0489	42	16	13	42	17	224	61	58	117
GN0503	52	13	56	60	22	264	144	58	99
GN0505	100	13	76	46	28	22	120	38	117
GN0512	85	13	92	60	28	303	140	36	101
GN0520	86	17	7	57	22	100	138	36	99
GN0521	42	17	61	59	2	175	122	56	101
GN0523	99	17	29	61	17	284	111	56	99
GN0527	106	17	81	61	25	262	69	33	99
GN0528	41	17	81	56	3	296	65	58	101
GN0529	100	13	76	46	7	22	120	38	117
GN0530	56	13	50	56	17	161	94	36	82
GN0538	93	17	91	61	17	134	116	58	99
GN0542	106	17	92	60	17	298	91	56	93
GN0544	52	13	85	60	17	243	144	56	75
GN0545	59	13	76	20	3	149	149	56	15
GN0546	96	17	92	57	28	135	129	57	99
GN0547	55	13	73	41	17	80	140	33	117
GN0551	106	25	58	23	17	286	137	40	82
GN0553	42	13	76	46	13	22	120	38	80
GN0558	42	17	81	41	3	86	140	36	118
GN0560	103	13	79	57	28	20	74	36	101
GN0562	42	17	87	42	17	214	119	38	40
GN0563	42	13	51	41	17	287	128	58	120
GN0564	25	17	79	19	17	255	108	33	42
GN0565	56	13	87	17	16	69	119	36	104
GN0569	42	24	78	27	7	291	159	40	54
GN0572	44	17	31	15	28	287	178	58	82
GN0573	56	25	58	23	3	286	137	40	42
GN0578	66	13	95	41	17	304	144	39	82
GN0579	101	13	79	41	28	238	131	58	99
GN0580	86	27	92	11	17	172	108	58	42
GN0581	25	17	79	19	25	255	108	33	42
GN0582	23	25	80	61	17	265	67	56	80
GN0585	102	17	95	23	17	191	72	56	117
GN0586	55	13	85	27	17	182	134	58	60

GN0587	107	17	95	23	25	107	125	56	99
GN0589	52	17	81	27	17	288	137	33	99
GN0590	88	17	81	27	17	288	137	33	99
GN0591	42	17	62	54	17	235	108	58	112
GN0592	107	17	95	57	17	68	137	55	101
GN0593	43	17	81	41	3	86	140	36	117
GN0595	85	13	98	60	17	150	133	33	101
GN0597	87	13	79	64	28	270	140	38	101
GN0602	24	17	43	40	17	260	161	56	37
GN0605	91	13	60	30	17	268	144	58	101
GN0606	96	17	80	61	17	265	129	56	66
GN0607	50	13	62	30	17	223	129	57	54
GN0608	24	13	95	57	17	264	15	58	80
GN0611	104	18	57	47	17	291	70	58	80
GN0612	88	13	85	60	17	243	144	56	115
GN0613	51	27	92	11	3	172	108	58	42
GN0617	59	17	59	40	3	287	75	57	80
GN0618	42	16	68	57	10	151	44	58	117
GN0619	42	13	76	46	13	22	120	38	117
GN0620	50	17	95	42	28	291	13	56	117
GN0622	50	17	80	18	3	264	172	56	99
GN0624	59	15	26	57	3	273	57	58	92
GN0630	55	13	73	41	17	80	119	33	117
GN0633	106	13	95	57	17	55	129	57	117
GN0636	56	25	58	23	3	286	110	40	56
GN0637	86	17	55	40	32	288	163	58	117
GN0638	107	27	95	14	32	104	140	58	120
GN0639	106	13	95	45	16	235	128	60	116
GN0641	101	13	95	42	28	288	159	58	117
GN0643	94	17	92	57	17	294	114	57	99
GN0644	100	17	87	42	17	214	119	38	70
GN0646	105	13	80	42	28	64	149	40	120
GN0648	102	17	95	23	25	191	72	56	117
GN0649	56	13	98	57	17	63	125	34	111
GN0650	99	17	87	40	25	206	46	33	67
GN0651	43	17	34	30	17	297	128	40	37
GN0652	100	13	14	65	17	265	137	58	80
GN0653	91	13	95	23	17	237	121	58	66
GN0656	91	11	80	27	17	296	129	58	40
GN0658	42	27	79	44	15	70	183	36	99
GN0659	91	13	80	61	25	265	129	56	101
GN0661	101	17	34	30	17	297	128	40	62
GN0662	55	17	79	22	17	143	144	58	120
GN0663	42	17	96	56	17	236	120	58	82

GN0664	106	13	87	42	17	253	119	33	93
GN0665	100	17	87	42	17	214	119	56	70
GN0667	100	17	87	42	17	214	119	38	69
GN0672	42	17	34	30	17	297	128	40	37
GN0673	42	17	96	56	17	236	120	58	56
GN0677	42	13	68	21	17	252	120	57	99
GN0678	86	17	81	27	17	288	137	33	99
GN0679	42	17	78	23	28	36	170	33	99
GN0680	42	17	87	5	3	214	119	38	40
GN0681	26	17	81	2	17	255	133	58	40
GN0682	23	13	58	18	17	126	129	62	120
GN0683	24	13	62	61	17	262	139	58	117
GN0684	91	7	81	41	28	135	149	58	113
GN0686	63	13	90	44	28	125	146	36	117
GN0687	27	13	77	61	17	273	70	58	99
GN0688	10	17	81	22	2	271	171	58	117
GN0689	29	17	81	27	17	255	133	58	40
GN0690	91	13	95	23	3	237	121	58	40
GN0691	46	13	90	57	22	273	137	58	117
GN0693	56	13	60	61	13	231	146	58	80
GN0695	30	17	76	32	28	279	54	58	40
GN0696	24	13	62	61	17	262	139	58	99
GN0701	86	13	62	27	17	262	139	58	100
GN0706	88	27	81	27	7	207	137	60	101
GN0707	100	17	87	42	17	214	119	38	67
GN0709	107	13	95	7	25	90	82	58	118
GN0711	99	17	97	63	17	264	159	58	82
GN0713	99	17	97	63	17	264	159	58	55
GN0714	99	-	97	63	17	264	159	58	81
GN0717	88	17	57	61	28	211	133	58	81
GN0718	91	16	14	49	22	284	126	33	117
GN0724	91	13	81	23	17	146	133	58	120
GN0731	106	17	71	9	17	287	137	36	66
GN0736	63	13	90	44	17	125	146	36	117
GN0740	91	13	20	20	28	149	140	60	120
GN0741	91	13	62	27	17	263	127	58	88
GN0742	91	16	14	53	22	284	126	33	117
GN0743	103	17	92	56	25	106	100	36	110
GN0744	67	13	92	57	17	237	114	58	101
GN0745	100	17	87	42	17	214	119	38	67
GN0747	34	13	79	57	16	20	74	36	101
GN0749	95	17	10	11	17	129	129	56	117
GN0750	109	17	79	27	17	284	128	56	99
GN0754	96	17	81	60	17	68	139	33	117

GN0756	106	13	76	56	17	288	129	61	120
GN0757	106	13	40	54	17	262	140	57	117
GN0758	85	17	95	57	17	303	133	58	120
GN0760	92	17	76	61	17	296	139	33	100
GN0761	109	13	81	42	17	247	109	56	66
GN0762	13	13	92	26	17	146	129	40	120
GN0763	106	13	80	41	17	304	125	38	120
GN0764	109	13	80	56	17	219	54	58	42
GN0766	66	17	87	25	17	298	137	36	13
GN0767	106	13	80	21	17	165	82	58	26
GN0770	66	15	80	60	28	275	129	58	120
GN0773	100	17	87	42	17	214	119	38	46
GN0774	99	24	78	61	28	291	159	40	87
GN0775	108	25	83	41	17	246	129	51	31
GN0777	108	25	83	41	17	246	129	61	31
GN0778	66	26	98	41	28	62	86	38	120
GN0783	31	13	87	45	28	60	170	33	24
GN0786	104	13	79	10	17	288	144	57	20
GN0789	91	17	81	27	17	255	133	58	66
GN0793	108	17	57	47	17	291	70	58	99
GN0794	86	17	55	40	28	288	163	58	117
GN0796	106	13	66	45	17	223	137	40	101
GN0797	35	16	57	57	28	285	74	58	117
GN0798	67	13	93	57	17	249	120	40	99
GN0800	84	17	49	46	17	30	146	36	117
GN0802	95	13	92	45	17	260	137	40	26
GN0804	34	13	87	42	17	255	147	40	56
GN0806	100	17	58	59	17	192	118	56	101
GN0807	66	25	80	60	17	67	132	56	42
GN0811	79	13	61	44	17	273	70	58	99
GN0812	92	16	87	23	28	227	137	36	15
GN0813	90	17	80	18	17	264	172	56	104
GN0814	66	17	66	46	25	290	160	62	120
GN0815	100	17	96	56	17	236	120	58	82
GN0816	83	13	81	26	28	217	124	58	67
GN0817	88	13	92	45	17	41	137	36	100
GN0819	100	17	87	42	17	-	119	38	46
GN0820	88	13	85	60	17	243	144	56	120
GN0822	88	13	85	60	17	243	176	56	115
GN0823	100	16	68	57	10	151	44	58	117
GN0824	96	17	95	57	17	282	129	58	66
GN0825	107	17	95	57	32	58	134	58	42
GN0827	99	13	87	56	17	39	140	56	101
GN0828	86	17	7	57	22	4	138	36	99

GN0830	80	15	95	45	17	304	137	36	26
GN0834	100	27	79	44	23	70	183	36	101
GN0835	83	13	81	60	28	217	124	58	66
GN0837	100	13	30	57	28	296	84	36	99
GN0839	100	27	79	44	15	70	183	36	100
GN0840	66	25	95	45	17	68	134	36	115
GN0844	99	13	66	57	16	78	137	56	101
GN0845	91	13	95	23	17	26	121	58	66
GN0847	91	17	19	45	7	298	110	58	112
GN0849	109	17	80	61	17	286	120	58	120
GN0852	106	17	80	23	7	286	120	58	120
GN0853	66	27	92	11	17	172	108	58	42
GN0854	100	17	87	46	17	214	119	38	46
GN0856	107	17	95	57	28	58	134	58	50
GN0858	79	13	79	45	19	147	140	33	101
GN0860	109	13	80	56	17	219	54	58	67
GN0864	86	18	80	−	17	269	118	33	101
GN0865	108	25	83	41	17	246	129	61	3
GN0865	106	13	58	57	25	291	92	36	67
GN0866	99	13	76	16	6	187	137	36	36
GN0867	107	13	80	48	17	197	16	58	120
GN0868	111	17	95	61	32	58	134	58	67
GN0870	103	17	92	56	25	106	100	36	117
GN0872	107	17	94	42	28	287	184	58	120
GN0874	66	13	95	41	15	304	144	39	56
GN0875	100	24	78	61	28	291	159	40	54
GN0877	100	13	8	57	17	288	129	57	26
GN0882	108	17	57	47	17	291	70	58	117
GN0884	91	25	92	46	16	284	93	40	66
GN0885	95	13	92	45	17	260	137	40	15
GN0886	109	17	87	46	17	−	−	38	46
GN0888	106	25	92	61	17	124	144	60	101
GN0889	79	15	95	45	17	304	144	39	56
GN0891	109	17	92	57	17	250	65	58	82
GN0895	96	17	92	57	28	135	129	57	101
GN0900	100	13	76	46	28	22	120	38	101
GN0902	66	15	80	26	28	275	129	58	120
GN0905	106	17	95	57	17	291	129	58	50
GN0908	106	17	92	61	16	214	140	58	46
GN0909	99	17	80	46	17	200	158	60	31
GN0910	99	15	79	40	17	245	140	34	24
GN0913	99	17	80	46	17	−	158	60	31
GN0914	99	17	80	42	17	200	158	60	33
GN0916	91	13	62	27	17	263	127	58	80

GN0917	109	17	95	42	22	53	120	34	101
GN0919	100	17	79	44	15	70	183	36	99
GN0923	108	11	19	60	25	247	66	56	26
GN0925	66	17	84	8	17	296	140	58	117
GN0926	99	17	80	46	17	200	158	60	6
GN0928	86	13	98	60	17	150	–	33	–
GN0931	103	17	92	56	25	23	100	36	117
GN0932	101	17	92	62	28	222	128	58	42
GN0933	109	17	79	61	17	277	129	60	46
GN0934	109	17	79	61	17	232	129	60	42
GN0935	92	13	85	61	17	182	134	58	94
GN0936	92	17	62	55	28	293	70	56	117
GN0944	84	15	95	56	25	51	128	34	46
GN0945	88	9	56	36	17	264	149	33	117
GN0946	80	13	37	9	28	202	91	57	129
GN0947	109	17	81	57	17	287	144	57	80
GN0948	110	11	81	11	17	303	47	34	48
GN0949	106	13	87	60	28	261	53	36	101
GN0950	106	18	42	10	17	288	84	58	91
GN0952	106	17	79	57	28	245	54	58	67
GN0954	86	13	62	61	17	262	139	58	117
GN0956	66	9	56	56	17	5	140	58	99
GN0957	106	13	81	57	28	92	55	36	101
GN0959	100	24	78	61	28	–	–	40	56
GN0963	92	17	76	61	17	296	139	33	101
GN0965	109	13	66	1	17	198	144	36	117
GN0972	89	13	95	40	17	264	123	56	77
GN0973	89	13	95	40	17	264	123	56	51
GN0974	80	13	49	42	7	296	169	58	33
GN0975	100	25	78	61	28	291	159	40	54
GN0976	106	13	66	61	17	240	55	60	101
GN0977	99	18	42	41	17	284	103	56	101
GN0978	62	13	80	55	17	64	140	36	120
GN0979	62	13	80	55	17	64	140	36	–
GN0980	92	17	98	45	17	296	101	40	42
GN0982	91	13	56	60	22	1	144	58	–
GN0984	109	13	42	57	28	219	48	58	101
GN0985	107	17	92	27	17	268	65	58	81
GN0986	92	17	81	11	28	271	176	50	120
GN0987	90	17	95	42	28	291	13	56	117
GN0990	62	13	80	55	17	64	140	36	117
GN0991	62	13	80	55	17	64	140	36	118
GN0992	91	17	92	15	7	296	127	33	99
GN0994	92	17	76	61	17	296	139	33	99

GN0995	87	13	79	61	22	246	144	56	30
GN1000	100	17	87	42	17	214	119	38	42
GN1002	100	27	87	44	23	70	182	36	101
GN1004	71	17	79	19	17	255	108	33	42
GN1005	89	13	95	40	17	264	123	56	48
GN1006	102	13	14	45	28	244	96	56	120
GN1007	85	25	95	58	17	299	109	56	101
GN1010	91	13	92	27	16	281	125	36	43
GN1011	91	13	92	27	16	281	125	36	63
GN1013	91	3	23	57	17	304	144	52	100
GN1014	91	17	23	57	17	304	144	60	120
GN1016	91	2	95	60	28	51	144	53	115
GN1018	96	1	81	59	17	273	137	9	127
GN1019	62	13	80	55	17	64	140	18	118
GN1020	109	13	98	57	25	212	149	52	64
GN1022	99	2	79	57	17	284	82	44	95
GN1026	99	2	81	27	17	166	84	15	100
GN1027	100	24	81	14	17	130	83	36	47
GN1028	86	19	69	45	17	130	127	16	98
GN1029	86	25	69	45	17	130	127	38	96
GN1032	66	13	39	54	17	298	144	46	28
GN1035	101	17	81	45	17	88	140	56	101
GN1038	85	23	52	60	17	267	137	46	105
GN1039	91	17	81	27	16	176	137	58	40
GN1040	100	17	87	42	17	214	119	25	73
GN1043	99	17	79	57	17	284	82	44	106
GN1044	109	13	66	61	25	271	137	44	108
GN1045	107	13	84	45	17	2	124	4	27
GN1046	99	15	80	58	17	53	106	56	101
GN1047	108	2	57	47	17	291	70	46	127
GN1048	88	1	56	60	22	264	144	46	100
GN1049	106	1	84	11	27	117	128	2	18
GN1050	88	13	51	22	17	245	107	36	37
GN1053	106	17	49	60	28	245	137	36	92
GN1055	100	2	81	27	17	166	84	3	108
GN1056	99	2	81	27	17	166	84	3	121
GN1057	107	17	92	27	17	268	65	58	54
GN1058	90	17	95	23	28	299	83	46	101
GN1061	91	-	50	57	17	243	137	47	127
GN1063	88	10	95	27	17	288	125	44	127
GN1064	99	13	31	40	17	296	137	46	85
GN1065	108	25	83	41	17	246	129	51	6
GN1066	106	17	49	60	28	245	137	3	106
GN1068	106	17	57	54	17	212	64	58	92

GN1069	85	13	98	60	17	150	133	1	109
GN1070	111	17	79	42	17	228	68	46	73
GN1073	66	25	81	60	17	296	106	36	15
GN1074	106	17	92	65	28	255	129	45	104
GN1075	106	17	80	60	17	226	144	58	38
GN1078	62	25	81	56	17	103	131	36	117
GN1079	88	13	41	61	28	225	139	58	120
GN1080	68	25	81	56	17	103	131	3	127
GN1086	106	13	40	14	27	219	140	41	120
GN1087	109	13	35	10	17	228	74	57	91
GN1089	66	13	95	41	17	304	144	39	56
GN1091	91	17	50	57	17	243	137	43	127
GN1092	99	13	92	61	17	223	95	56	31
GN1094	38	13	79	42	17	259	124	3	127
GN1097	36	17	78	42	28	254	149	36	99
GN1098	100	27	79	44	15	70	183	36	99
GN1104	99	13	83	10	17	234	90	37	101
GN1106	106	13	87	60	17	258	66	58	31
GN1107	109	13	87	60	17	258	66	46	6
GN1110	38	13	79	42	17	259	124	36	117
GN1111	62	13	80	55	17	64	140	27	127
GN1116	91	16	14	49	22	284	126	22	127
GN1119	106	17	95	57	17	265	139	56	54
GN1120	85	17	86	42	24	178	116	38	120
GN1130	91	13	92	46	16	284	93	8	89
GN1131	96	13	80	42	17	142	126	46	108
GN1132	91	13	90	57	17	168	131	58	117
GN1134	109	25	79	57	17	297	45	50	101
GN1135	109	17	79	64	28	265	73	58	101
GN1136	99	17	92	57	17	186	50	50	105
GN1138	109	13	80	56	28	256	129	50	84
GN1139	109	17	79	61	17	228	129	50	74
GN1140	109	13	37	27	17	208	134	44	120
GN1141	100	13	30	42	17	278	181	10	14
GN1142	100	25	27	56	17	284	74	6	126
GN1143	106	13	70	46	17	195	47	56	31
GN1144	110	11	81	11	25	303	47	23	83
GN1145	66	13	75	60	17	281	78	36	26
GN1146	99	25	80	60	28	284	137	58	120
GN1149	106	13	58	44	17	157	144	36	99
GN1150	109	17	57	54	17	212	64	50	122
GN1153	100	18	30	64	28	247	84	46	109
GN1157	92	16	87	23	28	227	137	36	26
GN1158	109	13	57	53	28	298	-	51	101

GN1162	91	11	57	14	17	298	149	46	67
GN1163	102	13	83	57	17	133	146	61	42
GN1164	86	17	95	42	17	288	178	58	117
GN1165	38	13	80	27	17	296	129	3	118
GN1167	46	17	79	42	17	259	124	46	127
GN1168	47	17	87	18	17	180	112	46	127
GN1169	46	17	91	23	17	89	127	46	127
GN1171	106	17	71	9	17	287	137	36	40
GN1174	106	17	95	42	17	288	178	9	28
GN1177	86	17	95	59	25	11	109	56	50
GN1178	99	17	81	27	17	166	84	36	99
GN1186	111	17	98	61	17	162	84	54	123
GN1187	100	13	92	57	17	188	100	27	72
GN1188	99	17	66	27	28	296	125	39	117
GN1189	83	26	87	45	22	283	144	60	26
GN1191	109	17	79	41	28	299	66	33	42
GN1195	91	13	30	65	28	233	94	9	127
GN1196	96	13	70	41	17	234	140	8	118
GN1198	99	17	92	56	17	14	128	46	127
GN1199	96	13	92	56	17	14	128	40	117
GN1200	109	13	57	14	28	298	149	61	120
GN1201	103	17	68	42	17	74	128	44	128
GN1202	106	27	81	45	17	88	140	50	127
GN1204	100	13	95	59	25	13	109	3	109
GN1205	100	13	77	45	28	263	140	9	106
GN1206	78	11	30	45	17	275	83	33	99
GN1208	103	16	53	41	17	265	103	3	125
GN1209	109	17	89	54	17	40	113	58	101
GN1210	100	17	41	57	17	148	113	46	125
GN1211	111	13	79	57	17	187	83	10	125
GN1215	106	13	61	46	6	235	140	62	118
GN1217	106	17	43	56	28	228	140	36	92
GN1218	100	13	95	48	17	296	58	58	42
GN1219	85	27	20	55	17	224	125	56	99
GN1222	106	25	85	41	17	281	84	36	42
GN1223	96	21	1	39	17	153	103	27	91
GN1225	106	25	79	46	17	123	88	58	95
GN1227	106	13	76	64	17	287	128	33	101
GN1228	102	13	98	27	17	183	94	40	92
GN1229	107	13	76	20	17	149	149	56	15
GN1230	99	17	79	57	17	187	83	58	112
GN1232	19	13	79	42	17	259	124	3	127
GN1233	107	17	6	23	17	304	109	61	120
GN1234	109	13	92	61	17	244	129	44	7

GN1237	79	13	80	56	17	258	135	27	125
GN1239	79	13	80	56	17	258	135	36	125
GN1245	83	13	87	13	17	185	46	46	108
GN1246	91	27	49	64	17	271	175	46	105
GN1249	101	27	87	42	17	187	63	56	46
GN1250	102	25	92	26	17	228	82	62	26
GN1251	93	13	95	40	17	264	123	44	83
GN1252	91	7	81	41	28	135	149	58	91
GN1253	100	13	25	58	22	299	138	46	127
GN1257	106	25	48	60	17	258	134	58	112
GN1258	95	13	76	26	17	159	144	25	115
GN1259	91	15	95	60	17	264	144	56	117
GN1262	107	13	80	51	17	291	73	36	15
GN1263	106	17	98	11	17	236	84	40	101
GN1264	106	25	79	57	17	297	45	58	104
GN1266	99	13	79	55	28	225	137	58	101
GN1269	80	25	95	45	17	304	137	44	29
GN1270	109	13	87	42	15	246	140	44	108
GN1273	79	13	95	41	17	304	144	17	86
GN1276	79	25	87	54	17	187	104	22	29
GN1277	66	25	87	54	17	187	104	33	26
GN1278	106	13	81	41	28	265	74	58	117
GN1279	106	17	81	10	28	296	74	56	117
GN1280	106	13	37	27	17	208	134	56	101
GN1281	109	13	37	27	17	208	134	44	128
GN1282	102	25	86	54	17	59	113	58	42
GN1283	48	13	31	57	17	264	123	44	127
GN1284	63	13	81	60	17	296	106	24	19
GN1285	106	17	57	54	17	212	64	58	101
GN1286	99	13	79	38	28	225	137	58	99
GN1287	99	13	79	-	28	225	137	46	108
GN1288	99	25	80	60	28	284	137	46	118
GN1289	45	25	80	60	28	284	137	46	118
GN1290	54	13	80	66	28	225	138	44	79
GN1292	95	17	83	57	17	54	98	58	101
GN1293	95	17	83	57	17	56	98	58	101
GN1295	107	13	92	64	17	93	141	36	120
GN1296	106	25	63	46	17	243	128	58	92
GN1297	104	13	79	60	17	196	94	33	120
GN1300	92	17	77	48	17	246	139	56	13
GN1304	46	25	95	58	17	299	109	44	109
GN1306	93	13	95	40	17	264	123	44	77
GN1308	45	13	92	61	17	223	95	44	6
GN1311	106	17	98	11	17	236	84	18	109

GN1312	79	25	81	59	28	120	149	56	112
GN1315	66	25	80	60	17	67	132	44	39
GN1316	95	13	92	45	17	260	137	13	26
GN1317	66	25	80	60	17	67	132	48	52
GN1318	99	18	95	57	17	172	131	46	94
GN1319	99	13	92	61	17	223	95	48	31
GN1321	99	17	95	41	28	257	91	12	115
GN1323	90	17	60	55	19	192	111	9	109
GN1325	83	13	81	60	28	217	124	58	40
GN1327	99	17	95	41	28	257	91	36	120
GN1329	80	13	95	56	17	51	128	5	50
GN1331	106	25	64	45	22	212	149	58	120
GN1333	106	17	93	60	17	199	128	33	91
GN1334	99	15	95	28	27	187	136	58	56
GN1336	99	15	95	-	27	187	136	46	56
GN1337	81	17	79	10	17	204	140	29	120
GN1338	88	13	80	27	22	258	138	46	117
GN1339	88	13	80	27	22	258	138	58	120
GN1340	62	13	80	55	17	64	140	27	118
GN1342	63	17	76	27	17	124	146	58	17
GN1345	91	13	95	23	17	237	121	50	41
GN1346	91	13	95	23	17	237	121	58	40
GN1347	88	17	81	27	28	146	144	56	120
GN1348	87	13	79	64	28	270	137	26	101
GN1349	107	17	58	57	7	235	131	50	99
GN1350	106	13	87	60	17	258	66	58	33
GN1351	88	-	83	41	17	296	139	56	13
GN1352	91	7	81	41	28	135	149	58	111
GN1353	91	13	92	53	16	284	93	40	56
GN1354	88	17	83	41	17	296	139	54	13
GN1355	107	17	77	20	25	246	70	58	120
GN1356	91	13	92	53	6	284	93	8	54
GN1358	81	17	79	10	17	204	140	8	120
GN1359	88	13	56	60	22	264	144	50	117
GN1360	99	25	93	56	32	110	140	58	101
GN1361	13	13	92	26	17	146	129	29	120
GN1363	107	15	80	23	17	256	129	48	118
GN1364	107	15	80	23	17	256	129	44	117
GN1366	86	17	95	42	17	288	178	58	120
GN1367	86	17	80	41	17	269	118	33	101
GN1368	86	17	80	-	17	269	118	9	101
GN1376	87	13	81	60	25	294	131	40	120
GN1377	89	13	95	40	17	264	123	56	54
GN1381	89	13	95	40	17	264	123	56	59

GN1384	75	25	86	39	17	296	131	50	120
GN1385	89	13	95	40	17	264	123	44	53
GN1394	107	27	76	56	17	141	144	46	99
GN1396	91	13	92	46	17	284	93	8	40
GN1397	106	13	98	57	25	51	140	49	42
GN1398	72	13	73	60	22	247	124	62	120
GN1403	89	13	95	40	17	264	123	44	48
GN1405	99	17	67	41	17	275	131	10	120
GN1406	107	25	5	22	17	94	95	58	120
GN1407	107	17	58	57	17	235	131	58	117
GN1408	107	17	58	57	17	235	131	50	99
GN1413	100	17	51	64	28	284	64	50	40
GN1414	109	13	42	57	28	219	48	46	92
GN1415	109	13	42	57	28	219	48	58	104
GN1416	99	17	81	57	17	247	82	58	99
GN1419	107	17	95	57	28	58	134	58	56
GN1421	91	17	9	60	28	245	140	58	101
GN1422	100	11	98	46	17	274	140	46	15
GN1423	106	17	69	16	28	290	124	51	41
GN1424	67	12	98	14	28	216	144	46	117
GN1426	106	13	80	60	16	291	144	36	99
GN1428	106	15	80	56	17	228	140	46	117
GN1429	106	13	80	60	16	291	144	36	100
GN1430	97	13	61	60	17	286	139	58	117
GN1431	106	25	50	56	28	161	94	24	112
GN1433	88	13	80	60	17	258	66	44	50
GN1434	107	27	44	56	32	82	140	36	91
GN1437	99	24	78	61	28	291	159	40	54
GN1438	101	13	80	60	28	237	94	58	26
GN1440	87	17	61	27	22	299	126	34	117
GN1442	92	13	80	57	17	249	125	56	54
GN1443	91	13	92	46	17	284	93	8	54
GN1444	9	17	66	27	28	296	125	39	117
GN1445	106	17	92	15	6	184	84	27	101
GN1446	109	13	77	15	17	121	149	50	95
GN1448	90	17	79	45	17	271	139	56	15
GN1450	101	13	19	57	22	289	138	58	101
GN1453	99	13	88	15	17	71	84	56	101
GN1454	75	-	86	39	17	296	131	50	120
GN1455	83	11	87	27	17	265	129	48	99
GN1456	106	13	87	12	17	296	137	62	120
GN1457	103	17	75	46	17	297	173	33	101
GN1458	106	13	87	-	17	296	137	62	117
GN1460	106	17	61	55	17	170	84	27	37

GN1461	91	13	81	23	17	146	133	50	120
GN1462	100	27	79	44	17	70	183	31	99
GN1467	103	17	91	59	17	295	109	39	120
GN1468	80	13	95	56	17	51	128	34	50
GN1471	62	25	69	61	17	278	63	33	26
GN1472	92	13	81	59	17	273	137	30	118
GN1473	92	13	98	45	17	99	128	7	54
GN1474	92	13	98	45	17	99	128	38	56
GN1476	95	17	83	57	17	56	98	50	101
GN1478	86	13	90	57	17	273	137	58	117
GN1479	99	13	92	61	17	223	95	44	31
GN1480	86	17	95	42	17	288	178	46	117
GN1481	109	13	42	57	17	219	48	58	92
GN1482	94	17	95	42	17	293	166	50	117
GN1485	92	17	80	56	17	38	138	36	111
GN1491	106	13	80	41	17	304	125	28	120
GN1492	79	13	95	41	17	258	140	39	112
GN1493	106	25	92	60	17	76	140	33	91
GN1495	89	17	91	23	17	89	127	58	99
GN1497	88	13	73	41	17	80	140	33	117
GN1501	99	13	66	57	17	78	137	48	101
GN1503	101	17	81	56	17	296	71	46	101
GN1504	111	25	95	68	17	304	174	38	38
GN1505	95	17	70	23	17	284	144	51	40
GN1509	95	17	70	23	17	284	144	61	40
GN1512	100	13	95	53	17	98	89	26	56
GN1513	70	15	92	57	17	297	128	33	32
GN1515	100	13	68	55	17	252	120	49	99
GN1516	88	13	56	60	17	264	144	46	117
GN1517	88	17	81	27	17	146	144	48	120
GN1518	101	17	81	56	17	296	71	58	104
GN1519	101	13	80	60	17	237	94	58	15
GN1520	66	15	83	57	17	301	69	58	101
GN1521	96	13	70	61	17	286	6	58	40
GN1522	91	7	81	41	17	135	149	58	117
GN1523	99	13	79	45	17	27	120	40	99
GN1524	106	13	98	57	17	212	149	52	39
GN1525	99	24	78	61	17	291	159	29	54
GN1526	106	24	80	60	17	197	92	56	117
GN1527	62	25	75	61	17	278	63	30	26
GN1530	106	13	98	57	17	212	149	52	38
GN1531	101	17	81	10	17	287	98	56	15
GN1532	101	17	81	10	17	287	98	44	15
GN1533	102	13	80	60	17	245	91	62	15

GN1536	106	17	87	49	17	108	131	56	120
GN1538	89	13	95	40	17	264	123	44	49
GN1540	101	13	80	60	17	237	94	50	12
GN1541	57	13	92	26	17	233	125	46	14
GN1542	107	13	95	60	17	250	4	52	23
GN1543	105	-	80	60	17	247	185	46	22
GN1545	90	17	60	55	17	192	111	33	101
GN1546	88	17	70	15	17	186	127	50	56
GN1548	103	25	79	14	17	139	60	58	99
GN1550	89	13	81	46	17	286	122	46	41
GN1551	106	13	80	44	17	31	123	44	99
GN1552	106	13	80	44	17	31	123	56	40
GN1553	66	25	80	48	17	66	126	14	26
GN1554	99	13	87	14	17	83	85	33	101
GN1555	101	18	81	45	17	88	140	56	100
GN1557	106	17	61	55	17	170	84	36	71
GN1562	101	13	92	27	17	81	94	36	101
GN1564	101	17	92	27	17	102	103	36	101
GN1566	61	17	55	40	17	282	113	58	94
GN1571	89	13	76	25	17	242	99	40	101
GN1572	88	17	70	15	17	186	127	58	82
GN1573	107	17	76	23	17	100	122	58	101
GN1576	100	13	92	61	17	222	80	56	82
GN1577	92	17	80	56	17	38	138	36	114
GN1578	70	11	83	40	17	28	170	36	99
GN1579	70	11	83	40	17	28	170	36	100
GN1580	88	16	86	57	17	33	144	40	67
GN1581	106	17	61	55	17	170	84	36	37
GN1582	88	17	70	15	17	186	127	58	59
GN1583	99	13	92	61	17	223	95	56	3
GN1584	102	13	79	56	17	296	128	36	101
GN1585	92	17	85	61	17	191	137	36	99
GN1587	103	13	85	14	17	255	70	58	120
GN1588	106	13	92	61	17	124	144	58	101
GN1589	69	13	83	45	17	282	137	36	117
GN1590	88	20	77	41	17	147	144	58	94
GN1591	103	13	85	14	17	255	70	58	120
GN1592	92	13	19	57	17	119	125	34	100
GN1593	101	17	81	61	17	186	137	36	100
GN1594	99	25	33	52	17	280	131	58	100
GN1596	99	25	33	52	17	280	-	58	99
GN1598	107	25	81	17	17	239	45	58	101
GN1602	99	17	81	27	17	166	84	36	100
GN1603	106	25	50	56	17	161	94	36	115

GN1604	106	17	49	60	17	245	137	36	95
GN1606	101	17	81	57	17	75	89	58	103
GN1607	85	17	68	42	17	99	125	58	120
GN1611	100	13	95	48	17	296	58	58	67
GN1615	99	17	79	57	17	284	82	56	95
GN1618	96	13	76	41	17	153	103	58	94
GN1619	18	17	70	40	17	292	93	58	101
GN1622	96	17	95	57	17	282	129	58	81
GN1623	83	25	98	44	17	44	85	33	26
GN1624	66	13	80	56	17	258	135	36	115
GN1625	96	17	76	41	17	153	103	58	94
GN1626	63	25	76	43	17	114	74	58	95
GN1627	63	25	76	43	17	114	74	58	101
GN1629	109	24	21	46	17	284	87	33	67
GN1630	100	13	79	52	17	227	146	56	120
GN1632	96	13	76	41	17	153	103	58	91
GN1633	103	7	81	41	17	135	134	58	99
GN1634	100	17	57	61	17	270	139	56	101
GN1636	109	17	92	57	17	250	65	58	56
GN1637	111	13	79	41	17	93	94	33	101
GN1639	89	25	58	42	17	145	94	56	120
GN1640	17	17	67	63	17	300	93	33	95
GN1661	106	13	76	41	17	151	103	58	11
A232	72	25	86	39	17	296	131	58	120
A236	109	18	69	9	17	284	95	58	99
A237	95	17	81	57	17	303	131	58	67
A240	66	13	81	60	17	296	106	36	15
A242	79	8	87	42	17	264	172	58	118
A247	96	16	22	57	17	288	64	32	100
A252	89	15	81	60	17	251	149	56	115
A263	15	15	87	45	17	291	130	58	101
A271	88	11	80	27	17	296	129	58	40
A276	88	17	83	60	17	296	139	56	13
A278	101	17	81	45	17	17	140	56	101
A280	86	13	81	41	17	152	82	58	120
A284	88	26	75	60	17	186	137	56	120
A331	91	13	92	46	17	284	93	40	82
A346	88	13	85	60	17	251	144	56	120
A348	61	17	55	40	17	282	113	58	111
A354	91	17	90	60	17	246	70	56	112
A369	89	13	85	58	17	228	118	58	92
A377	99	13	80	57	17	226	128	58	92
A393	95	13	96	56	17	131	134	58	99
A397	66	13	97	27	17	275	129	56	118

A400	87	25	36	57	17	154	144	55	14
A402	79	13	78	39	17	25	91	58	92
A403	109	17	76	48	17	299	156	32	101
A421	109	17	98	65	17	265	144	58	82
A422	109	17	92	65	17	265	144	58	82
A423	91	16	69	49	17	284	126	33	118
A429	99	13	83	45	17	297	128	58	101
A430	62	13	27	24	17	287	144	58	99
A442	100	13	95	41	17	169	137	36	114
A445	73	17	92	61	17	162	131	58	67
A447	99	13	64	46	17	223	140	36	101
A455	79	13	61	27	17	121	144	62	120
A457	106	24	85	61	17	247	66	58	15
A468	106	17	61	55	17	170	84	36	63
A476	101	17	81	57	17	75	89	58	114
A478	109	17	92	57	17	250	65	58	46
A493	88	17	87	42	17	215	70	58	101
A494	106	4	58	57	17	291	92	36	67
A508	91	13	92	46	17	284	93	40	81
A517	109	17	81	57	17	287	144	57	81
A521	99	17	50	11	17	178	78	36	100
A523	106	25	56	56	17	161	94	36	120
A534	75	17	79	56	17	296	129	40	120
A540	96	13	76	41	17	153	103	58	103
A542	88	13	85	60	17	251	144	56	115
A552	58	17	–	–	17	16	186	58	107
A555	100	15	81	57	17	304	107	58	67
A557	100	17	81	57	17	243	141	36	67
A562	100	15	81	57	17	304	107	58	81
A567	66	13	48	56	17	51	124	39	50
A570	108	22	85	56	17	228	47	62	12
A571	86	13	95	57	17	227	89	58	99
A579	87	25	96	61	17	230	139	58	102
A587	22	12	81	10	17	247	128	58	99
A588	91	13	70	56	17	105	140	36	117
A589	102	13	79	68	17	96	137	58	117
A591	102	21	78	56	17	228	51	62	15
A597	62	13	80	14	17	216	185	58	93
A604	106	13	80	56	17	299	84	58	90
A605	91	13	92	46	17	284	93	40	54
A610	91	13	92	46	17	284	93	40	80
A620	83	11	87	27	17	265	129	56	99
A621	106	13	85	56	17	284	140	56	76
A625	63	13	81	44	17	127	144	56	99

A628	91	13	92	46	17	284	93	40	42
A631	101	17	81	10	17	296	65	58	101
A634	99	25	87	57	17	285	77	38	101
A638	99	13	61	61	17	228	146	56	82
A639	88	17	56	60	17	276	125	33	117
A640	87	17	77	20	17	284	137	33	82
A641	99	17	79	61	17	296	140	58	67
A646	100	13	95	41	17	169	137	36	113
A647	106	15	79	57	17	299	140	55	12
A652	96	17	76	41	17	153	103	58	93
A653	101	25	79	41	17	101	137	58	99
A654	106	13	92	61	17	244	129	56	3
A659	106	13	66	45	17	303	171	58	26
A660	99	13	95	52	17	242	185	58	95
A662	37	17	92	40	17	299	179	40	101
A664	66	25	47	57	17	288	120	59	78
A665	81	27	76	64	17	112	137	56	117
A666	61	13	95	61	17	244	127	56	62
A668	107	17	80	45	17	91	69	58	101
A669	94	13	81	45	17	258	102	56	80
A672	100	17	35	45	17	274	93	56	118
A674	106	13	76	42	17	228	109	57	117
A681	91	13	92	46	17	6	93	40	80
A683	99	13	76	60	17	245	144	56	82
A689	106	13	78	46	17	293	92	36	67
A690	106	13	82	48	17	268	70	58	-
A691	99	13	42	56	17	265	141	33	120
A694	82	12	43	61	17	231	176	62	120
A698	76	18	95	60	17	264	144	58	99
A699	109	13	95	56	17	132	144	34	100
A700	100	17	61	59	17	175	122	56	101
A701	91	13	92	46	17	284	93	40	82
A702	101	13	56	55	17	72	82	58	95
A703	102	13	83	56	17	258	91	56	6
A704	95	13	77	45	17	237	93	62	101
A706	109	13	61	41	17	115	140	58	92
A709	106	13	61	46	17	235	140	62	120
A712	106	25	50	56	17	161	94	36	120
A713	107	17	77	20	17	246	70	58	101
A715	107	13	80	51	17	291	73	36	3
A716	88	9	95	41	17	303	95	60	56
A717	101	17	51	64	17	284	64	58	40
A718	99	13	81	60	17	235	83	56	6
A719	106	13	70	46	17	195	47	48	31

A722	67	14	31	46	17	275	43	56	99
A724	66	17	40	42	17	296	179	61	15
A726	101	13	66	60	17	190	149	36	112
A729	79	13	92	46	17	287	171	39	13
A731	102	13	98	41	17	228	100	55	120
A737	79	18	3	61	17	263	119	36	116
A742	99	13	98	41	17	233	119	60	117
A744	64	-	2	57	17	216	137	33	101
A746	87	13	88	64	17	32	92	58	99
A748	78	17	88	53	17	182	42	58	99
A749	98	13	66	42	17	128	59	56	99
A751	101	13	87	45	17	36	82	58	101
A752	100	13	87	57	17	189	102	60	117
A753	101	13	76	41	17	297	119	58	103
A757	106	13	88	60	17	163	172	36	99
A759	111	25	27	46	17	297	137	55	120
A760	86	13	64	42	17	12	11	58	104
A764	4	25	80	46	17	10	140	58	103
A765	5	17	-	6	17	136	9	54	40
A768	92	15	98	61	17	291	91	38	120
A769	106	13	92	60	17	45	85	33	99
A770	88	13	87	60	17	253	140	36	24
A772	32	13	80	45	17	35	178	58	117
A779	92	11	80	59	17	46	140	62	114
A781	108	13	83	21	17	65	149	58	95
A782	79	13	61	44	17	273	70	58	100
A783	92	11	80	59	17	46	140	62	124
A784	65	13	16	58	17	159	144	58	73
A785	99	13	51	41	17	287	128	58	120
A787	63	25	59	44	17	144	149	58	108
A790	67	12	98	14	17	216	144	58	100
A792	88	13	90	56	17	116	144	56	109
A793	99	13	46	60	17	212	103	33	7
A794	99	13	87	56	17	243	128	58	92
A795	63	25	59	44	17	144	149	58	99
A796	99	13	87	56	17	243	128	58	95
A798	106	13	88	60	17	163	172	36	117
A799	83	13	88	43	17	264	70	60	28
A800	106	24	85	61	17	247	66	58	19
A801	87	17	72	61	17	231	137	56	118
A803	99	13	51	41	17	287	128	58	82
A803	106	13	28	58	17	61	86	38	120
A806	66	13	69	41	17	298	178	58	92
A808	63	25	59	44	17	144	149	58	100

A813	37	17	98	44	17	209	173	40	120
A814	90	17	87	40	17	205	173	40	101
A815	37	17	79	42	17	206	173	40	101
A816	102	25	86	54	17	59	113	58	67
A817	102	13	86	54	17	59	113	58	67
A819	102	25	86	54	17	59	113	58	67
A821	88	13	56	60	17	264	144	58	108
A824	101	17	24	41	17	179	90	36	108
A827	88	25	98	9	17	61	139	62	85
A828	87	13	80	32	17	299	84	36	108
A829	108	11	19	60	17	247	66	56	26
A830	88	25	98	9	17	61	139	62	67
A832	67	14	31	46	17	275	43	56	101
A833	101	17	54	41	17	237	84	58	101
A834	88	25	66	60	17	223	137	58	25
A835	100	13	76	14	17	246	74	58	-
A836	106	13	76	64	17	287	128	33	109
A839	101	16	54	41	17	237	84	58	101
A840	102	13	98	27	17	183	94	62	104
A842	88	13	80	60	17	304	56	58	82
A843	106	13	80	56	17	299	95	58	112
A845	85	13	47	55	17	285	12	58	114
A847	88	25	85	56	17	271	149	58	118
A849	99	13	87	15	17	229	140	58	101
A854	106	13	80	56	17	299	95	58	114
A855	106	13	95	58	17	270	137	58	117
A856	99	25	59	44	17	144	149	58	100
A859	99	13	60	11	17	296	149	61	95
A863	109	13	95	56	17	132	144	34	99
A869	90	13	38	23	17	139	120	57	65
A888	88	13	85	36	17	243	144	56	78
A889	101	13	75	37	17	302	118	58	118
A890	101	13	65	23	17	254	170	56	101
A905	99	17	69	14	17	303	128	58	101
A906	101	13	84	60	17	294	140	36	101
A907	88	13	86	61	17	295	144	58	99
A908	88	13	95	68	17	48	140	36	94
A909	88	13	80	57	17	171	145	36	94
A910	100	13	76	54	17	198	1	36	67
A912	111	13	80	45	17	279	144	58	101
A913	87	17	79	41	17	296	129	36	101
A914	90	25	83	45	17	279	52	58	99
A915	96	13	83	45	17	122	60	58	94
A917	90	17	92	44	17	180	158	36	101

A918	105	25	92	60	17	247	137	36	3
A968	101	11	27	56	17	37	178	58	101
A969	111	11	27	46	17	299	168	58	15
A970	92	13	87	60	17	294	178	60	101
A971	101	11	27	56	17	37	178	58	120
A997	101	13	87	26	17	246	144	58	6

Table S4. Phenotypics of 1021 sweet potato germplasm resources.

Accession	Phenotypic															
number	a	b	c	d	e	f	g	h	i	j	k	l	m	n	o	p
GN0001	6	6	1	2	2	4	2	2	2	1	3	2	3	6	2	5
GN0002	7	3	1	2	2	4	0	2	2	3	3	3	3	5	3	3
GN0005	6	6	1	2	2	4	1	2	3	1	3	2	3	3	8	1
GN0007	6	6	1	2	2	3	1	5	2	3	3	2	3	3	2	2
GN0008	2	6	2	2	3	4	1	2	2	3	3	2	3	5	8	1
GN0010	7	3	1	2	2	2	3	2	2	3	3	0	3	6	9	2
GN0012	5	4	1	2	2	2	0	2	2	3	2	0	3	6	3	4
GN0013	2	6	1	2	2	4	1	2	3	3	3	2	3	6	4	1
GN0015	6	6	1	2	2	4	0	5	2	2	3	2	4	6	4	1
GN0018	6	4	1	2	2	4	2	2	2	2	3	2	3	3	8	1
GN0019	6	4	2	2	2	4	0	2	3	2	2	2	3	5	8	4
GN0023	2	6	2	2	3	4	0	2	2	3	3	0	2	6	1	1
GN0025	2	6	1	2	2	4	2	2	2	3	3	0	3	5	8	1
GN0027	2	4	2	2	2	4	3	5	2	2	4	2	3	5	5	3
GN0029	2	3	1	2	3	3	2	2	3	2	3	0	2	5	6	1
GN0030	2	6	1	2	2	4	1	2	2	2	3	2	4	5	6	1
GN0031	2	6	1	2	2	3	1	2	2	1	3	2	4	5	8	2
GN0032	2	3	1	2	2	3	0	2	2	2	2	2	2	3	3	1
GN0033	6	3	2	2	2	4	0	5	2	3	2	2	2	5	5	3
GN0037	2	6	1	2	2	2	0	2	2	2	3	2	3	5	1	1
GN0038	7	6	1	2	3	4	2	2	2	3	3	2	3	3	7	1
GN0039	2	6	2	2	2	4	1	5	2	1	3	2	4	6	6	1
GN0040	2	3	2	2	2	3	0	2	2	3	3	0	2	6	1	2
GN0041	2	6	1	2	2	4	1	2	2	2	3	2	3	3	1	1
GN0043	2	3	1	2	2	4	3	5	2	4	2	2	2	5	8	1
GN0047	6	3	1	2	2	4	3	2	2	2	3	0	4	3	1	9
GN0049	2	3	2	2	2	3	0	2	2	2	3	2	3	7	1	1
GN0050	2	6	2	2	2	4	1	2	2	2	3	2	4	3	2	1
GN0051	6	3	1	1	3	4	0	2	3	2	2	2	2	1	5	1
GN0052	2	6	1	2	2	3	0	2	1	2	3	0	4	5	1	1
GN0056	2	3	2	1	2	3	1	2	2	1	3	2	3	5	8	1
GN0059	2	6	1	1	2	3	3	2	1	2	3	2	4	3	8	1
GN0062	2	3	1	2	2	3	0	2	2	1	3	0	3	6	8	3
GN0064	6	3	1	2	2	2	1	2	2	2	3	2	2	3	4	3

GN0069	5	5	1	2	3	4	2	2	2	2	3	3	3	6	1	1
GN0070	2	6	2	2	3	4	3	2	2	2	3	2	3	3	9	3
GN0071	2	3	2	2	2	3	3	2	2	2	3	2	4	3	8	2
GN0075	2	3	2	2	2	4	0	2	3	1	3	2	3	5	8	2
GN0080	7	3	2	2	2	3	1	2	2	3	3	2	3	5	3	4
GN0094	2	6	1	2	2	4	2	2	2	2	3	2	4	5	9	1
GN0099	2	6	2	2	2	4	2	2	1	2	3	2	4	3	8	1
GN0103	7	3	1	2	2	4	1	5	2	2	3	2	4	3	6	2
GN0106	2	6	2	2	1	4	0	5	1	2	3	2	3	6	8	1
GN0107	2	6	1	2	1	4	0	5	2	2	3	2	2	3	9	2
GN0111	7	5	1	2	3	4	1	2	2	3	3	2	3	3	2	2
GN0113	2	6	2	2	2	4	1	2	2	2	3	2	3	5	1	1
GN0115	7	6	2	2	1	2	1	2	2	2	3	-	4	3	8	1
GN0120	2	3	2	2	2	4	0	2	2	2	3	0	2	5	1	1
GN0121	2	6	1	2	2	4	1	2	2	2	3	0	2	3	4	3
GN0123	5	6	1	2	2	2	1	2	2	3	3	2	3	3	5	1
GN0124	2	6	2	2	2	4	0	2	3	3	2	2	2	3	2	2
GN0125	2	3	2	1	2	2	3	2	1	3	3	0	3	5	2	2
GN0126	2	6	2	2	3	4	0	2	2	2	2	0	1	0	6	1
GN0128	2	3	2	2	2	4	1	2	2	3	3	0	2	3	8	1
GN0130	2	6	2	2	2	4	1	2	2	3	3	2	2	3	8	2
GN0132	2	6	1	2	2	4	1	5	2	2	2	2	2	5	8	9
GN0133	2	6	2	2	2	4	0	2	2	3	3	3	3	5	9	1
GN0135	2	3	1	2	2	3	1	2	3	1	3	3	3	6	8	1
GN0137	2	6	2	2	2	4	1	2	2	1	3	2	3	5	3	1
GN0138	2	6	2	2	2	4	0	5	2	2	3	3	3	6	1	1
GN0139	7	5	1	2	2	4	0	2	3	3	2	3	3	3	8	1
GN0144	1	6	1	2	2	3	0	2	2	2	3	2	2	3	6	2
GN0150	7	3	1	2	2	2	3	2	2	2	3	0	3	3	8	3
GN0151	7	6	1	2	2	2	2	2	2	3	3	2	3	3	8	3
GN0153	6	6	1	2	3	4	0	2	2	3	3	4	3	3	2	1
GN0155	2	3	2	2	2	4	0	2	2	3	3	2	2	5	1	1
GN0156	2	3	1	2	2	4	0	5	2	3	3	0	2	3	1	2
GN0157	7	3	1	2	2	4	0	2	2	2	2	2	3	6	9	1
GN0158	2	6	1	2	3	4	0	2	2	3	3	2	3	6	1	1
GN0162	2	6	1	2	3	4	1	2	2	3	3	0	3	5	1	1
GN0168	7	6	1	2	2	4	1	2	2	2	3	4	3	3	4	5
GN0169	2	6	2	1	3	2	1	2	2	3	2	0	2	7	1	1
GN0170	2	6	1	2	3	3	2	2	2	2	3	2	3	5	4	1
GN0172	1	2	1	2	2	2	3	2	2	1	3	0	4	5	7	5
GN0173	6	6	2	2	2	3	0	2	1	3	3	2	2	3	7	2
GN0179	2	3	1	2	1	4	2	5	1	2	3	0	3	3	1	1
GN0180	7	3	1	2	1	2	3	2	1	1	3	2	2	3	8	2
GN0181	6	6	2	2	3	2	0	2	2	3	3	2	2	7	1	3

GN0183	2	6	2	2	2	4	0	2	2	2	3	2	3	3	1	1
GN0184	2	6	1	2	2	2	2	2	2	3	3	2	2	5	6	3
GN0186	2	3	1	2	1	4	2	2	2	2	3	–	2	6	1	1
GN0187	2	6	1	2	2	2	1	2	1	2	3	2	4	6	8	7
GN0191	2	3	1	1	2	4	1	2	2	2	3	0	3	3	8	1
GN0192	7	3	2	2	2	4	0	5	2	3	2	2	3	3	8	1
GN0196	2	4	1	2	2	4	1	2	2	3	2	0	1	6	8	1
GN0197	2	6	2	2	3	4	1	2	2	2	2	0	1	3	8	1
GN0198	1	3	2	1	2	2	0	2	2	3	2	0	2	6	1	1
GN0199	6	5	2	2	2	3	0	2	2	1	3	3	1	6	7	1
GN0200	2	3	1	2	3	3	3	2	2	2	3	0	1	3	8	5
GN0201	2	3	2	2	3	4	2	2	2	1	3	2	1	6	8	1
GN0203	6	3	2	2	2	4	1	2	2	3	2	0	2	3	1	3
GN0205	2	6	1	2	2	3	1	2	2	3	2	2	1	1	6	1
GN0206	2	3	2	2	2	4	2	2	2	1	2	4	1	3	6	1
GN0207	2	3	1	2	2	4	2	2	2	1	2	2	1	3	2	2
GN0208	2	6	2	2	3	4	2	5	2	1	3	3	1	6	6	1
GN0209	2	6	1	2	2	4	1	2	2	3	2	4	2	6	2	2
GN0210	2	3	2	2	2	4	1	2	2	1	3	2	1	3	6	1
GN0211	2	6	2	2	3	4	2	2	2	1	3	3	1	6	4	2
GN0212	2	3	1	2	2	4	0	5	2	1	2	4	2	6	9	9
GN0213	2	3	2	2	3	4	1	2	2	2	2	2	1	3	3	2
GN0214	2	6	1	2	3	4	1	2	2	3	3	3	1	3	8	2
GN0215	2	3	2	2	2	4	2	2	2	2	3	2	1	3	2	2
GN0216	2	3	1	2	2	4	2	2	2	2	3	2	2	3	8	2
GN0217	2	6	1	2	3	4	2	2	2	3	3	0	2	3	1	2
GN0218	2	6	1	2	2	4	1	2	2	3	3	0	3	3	1	1
GN0219	2	6	2	1	1	4	2	2	1	4	3	0	1	6	8	2
GN0224	2	6	2	2	2	3	0	2	3	3	2	2	1	6	8	1
GN0257	2	6	1	1	2	2	1	2	2	3	2	0	1	6	5	1
GN0259	2	6	1	2	2	4	1	4	2	2	3	3	2	6	1	2
GN0262	2	4	1	1	2	2	0	2	2	3	2	3	1	6	1	2
GN0271	2	6	1	1	2	4	1	2	2	3	2	3	1	6	9	1
GN0272	6	6	1	2	2	4	1	2	2	2	3	0	1	6	5	3
GN0284	6	3	1	2	3	3	0	2	3	3	3	0	2	3	7	1
GN0292	5	6	1	2	2	4	0	2	2	3	3	0	3	5	6	2
GN0297	2	6	2	2	2	4	0	2	2	3	2	0	2	3	8	1
GN0298	2	6	1	2	3	4	1	2	3	2	3	0	2	3	7	3
GN0299	2	6	1	2	2	4	0	2	2	3	3	0	3	6	8	3
GN0300	2	3	2	2	2	4	2	2	2	3	3	0	3	5	9	2
GN0304	6	6	1	2	2	3	2	2	2	3	3	0	2	5	9	1
GN0305	6	3	2	2	3	4	2	5	2	3	3	0	4	3	1	1
GN0307	5	3	1	2	3	4	3	2	2	2	3	0	1	6	6	4
GN0312	6	3	1	2	1	2	1	5	2	3	3	0	2	5	5	2

GN0320	2	4	2	2	2	4	2	5	2	3	3	0	2	3	9	3
GN0324	2	3	2	2	3	4	0	2	2	2	2	0	2	3	1	2
GN0326	6	3	1	2	2	3	0	2	2	3	3	0	2	3	5	1
GN0351	2	3	2	2	3	4	1	5	2	4	2	0	1	3	8	4
GN0355	2	6	1	2	3	4	3	2	2	2	3	0	3	6	4	2
GN0356	2	6	2	2	2	4	3	2	2	2	3	0	2	6	7	3
GN0362	6	3	2	2	2	4	3	5	2	3	3	0	2	3	5	2
GN0363	6	3	2	2	2	2	0	2	2	1	2	0	1	3	3	1
GN0365	5	3	1	2	2	2	1	2	2	2	3	4	3	3	1	2
GN0374	2	3	-	2	2	4	0	4	2	2	2	0	1	3	7	1
GN0375	2	6	1	2	2	3	1	2	2	2	2	0	1	3	1	1
GN0376	2	6	2	2	2	3	2	2	2	2	3	3	2	3	6	1
GN0380	2	3	1	2	2	3	0	2	2	3	2	0	1	2	1	2
GN0381	2	3	2	2	2	4	1	5	2	1	3	2	2	2	8	4
GN0382	2	3	2	2	3	4	2	5	2	1	3	0	2	3	7	4
GN0383	2	6	2	2	2	2	3	2	2	1	3	3	2	3	8	4
GN0388	2	6	1	2	3	3	3	2	2	1	3	3	2	3	8	1
GN0389	2	3	1	2	2	4	1	5	2	2	3	0	2	2	5	2
GN0391	2	6	1	2	3	4	0	2	2	3	2	2	1	3	4	4
GN0392	2	3	2	2	2	4	0	5	2	1	2	2	1	2	8	4
GN0396	2	3	2	2	2	4	2	5	2	1	2	0	2	6	8	4
GN0397	2	3	2	2	2	4	1	5	2	1	2	4	1	6	1	2
GN0398	2	3	2	2	3	4	1	5	2	1	2	0	2	6	7	3
GN0399	2	3	1	2	3	4	1	2	2	1	2	2	1	3	5	2
GN0400	2	3	1	2	3	4	2	5	2	1	3	2	2	6	6	1
GN0401	2	6	1	2	3	4	0	5	2	3	2	3	1	6	1	1
GN0402	2	3	2	2	3	4	3	5	2	2	2	2	3	3	8	3
GN0404	2	3	2	2	3	3	3	5	2	1	2	0	1	6	5	4
GN0410	2	3	2	2	2	4	1	5	2	1	2	2	1	3	3	4
GN0411	2	5	2	2	2	4	0	2	2	3	2	2	1	3	2	4
GN0414	2	3	1	2	2	4	2	5	2	2	3	0	1	1	9	2
GN0415	2	3	2	2	3	4	2	2	2	1	2	4	1	2	8	3
GN0417	2	3	1	2	2	4	2	5	2	1	3	2	2	6	8	3
GN0418	2	3	2	2	3	3	1	2	2	1	2	2	1	2	8	3
GN0419	2	3	2	2	2	4	2	5	2	1	3	0	2	6	9	3
GN0420	6	3	2	2	3	4	3	2	2	1	2	0	1	3	8	4
GN0421	2	6	1	2	3	2	3	2	3	1	3	0	2	3	1	3
GN0422	2	6	1	2	2	4	2	2	2	2	2	0	1	2	8	1
GN0423	2	3	2	1	3	3	2	2	2	2	2	0	1	2	2	2
GN0425	2	6	1	2	2	3	2	2	2	1	3	4	1	2	1	2
GN0426	2	6	1	2	3	4	0	5	2	1	2	0	1	3	8	3
GN0427	2	3	2	5	2	4	1	5	2	3	3	2	2	2	1	2
GN0429	2	3	2	2	3	4	0	2	2	2	2	3	1	3	8	2
GN0430	2	3	2	2	3	3	1	2	3	1	2	2	1	2	7	3

GN0433	6	3	2	2	2	4	1	2	2	2	3	4	1	3	4	3
GN0434	2	3	2	2	3	4	2	2	2	2	2	2	1	3	7	4
GN0437	2	3	1	2	2	3	2	2	2	2	3	2	1	2	1	1
GN0438	2	3	2	2	3	4	0	2	3	1	2	2	1	3	5	4
GN0440	7	3	2	2	2	3	0	2	2	1	2	4	1	2	8	2
GN0442	2	3	1	2	3	4	2	2	3	1	2	0	1	2	3	3
GN0446	2	3	1	2	3	4	2	5	2	1	3	0	1	3	8	2
GN0449	2	6	2	2	2	4	2	5	2	2	3	2	2	3	8	5
GN0451	2	3	1	2	2	3	2	2	2	1	2	4	1	6	3	5
GN0453	2	3	1	2	2	3	3	2	2	2	3	0	1	3	5	2
GN0454	2	3	2	2	2	2	2	2	2	1	2	4	1	4	5	4
GN0460	2	3	2	2	3	3	1	2	2	2	3	4	1	3	8	2
GN0462	2	3	1	2	2	4	1	2	2	3	3	4	1	6	6	2
GN0463	2	3	2	2	3	4	0	2	2	2	3	0	1	2	3	4
GN0467	2	6	2	2	2	4	1	2	1	3	3	–	2	6	4	1
GN0468	2	6	1	2	3	3	0	2	2	3	3	2	2	2	1	1
GN0469	2	6	1	2	3	4	1	2	2	2	3	2	3	2	8	2
GN0470	2	3	1	2	2	2	2	2	2	3	3	0	2	6	8	1
GN0471	2	6	1	2	2	4	2	2	2	2	3	0	1	4	8	2
GN0473	2	3	2	2	2	3	3	5	2	3	3	2	2	7	1	1
GN0479	5	3	2	2	2	2	0	2	2	2	3	2	3	6	7	1
GN0480	5	3	1	2	3	2	2	2	2	1	3	2	2	3	8	3
GN0489	2	6	1	2	2	4	0	2	1	3	3	2	1	3	5	2
GN0503	2	5	2	2	3	4	2	2	2	2	2	0	1	6	1	1
GN0505	2	3	0	1	3	3	2	2	2	1	2	0	2	3	9	3
GN0512	2	3	2	2	3	4	0	5	2	2	3	0	1	2	6	3
GN0520	2	3	2	2	2	3	2	5	2	1	3	0	1	6	6	2
GN0521	2	3	1	2	2	4	2	5	2	2	3	2	2	3	1	2
GN0523	5	3	1	2	2	4	0	2	2	2	2	2	2	3	9	1
GN0527	2	3	1	2	2	4	2	2	2	3	2	3	1	3	9	1
GN0528	2	3	1	2	3	4	2	2	2	3	2	4	2	2	9	3
GN0529	6	6	1	2	2	4	0	2	2	3	3	4	2	3	7	1
GN0530	2	3	1	2	2	4	2	2	2	3	2	3	1	6	8	1
GN0538	2	3	2	2	2	4	0	5	2	1	3	3	2	6	4	2
GN0542	2	6	2	2	2	4	1	2	2	3	3	0	3	3	1	1
GN0544	2	3	1	2	3	4	2	2	2	2	3	0	2	3	8	1
GN0545	2	4	1	2	2	4	2	5	2	2	3	0	1	1	9	1
GN0546	2	3	2	2	3	4	1	2	2	2	2	0	1	2	7	3
GN0547	6	3	2	2	3	4	1	2	3	3	3	4	1	6	4	3
GN0551	7	3	1	2	3	4	3	2	2	1	3	0	2	6	7	5
GN0553	2	6	2	2	3	4	2	2	2	2	3	0	1	3	9	3
GN0558	6	6	1	3	3	4	0	2	2	2	3	3	1	0	4	4
GN0560	2	3	2	2	2	4	0	2	2	1	3	4	1	0	7	1
GN0562	2	6	2	2	3	3	0	2	2	4	3	0	2	5	1	1

GN0563	2	6	2	2	3	4	0	2	3	2	3	0	2	6	1	1
GN0564	2	3	2	2	3	4	0	2	2	3	3	3	1	6	4	4
GN0565	2	3	2	2	3	4	0	2	2	3	3	3	1	6	4	4
GN0569	6	3	1	2	2	2	3	2	2	2	3	0	2	3	7	2
GN0572	7	6	2	2	3	4	0	2	2	3	3	0	2	8	4	1
GN0573	2	3	2	1	2	2	2	2	2	3	2	2	1	4	1	3
GN0578	2	3	2	2	3	2	3	2	2	2	2	3	1	2	7	2
GN0579	2	3	2	2	2	4	2	2	1	3	3	3	2	0	7	9
GN0580	2	6	1	2	3	4	0	2	2	4	2	0	2	7	8	5
GN0581	2	6	1	2	3	4	0	2	2	3	2	0	2	3	6	1
GN0582	2	6	2	2	3	4	1	4	2	3	3	0	2	3	1	1
GN0585	2	5	1	2	2	4	3	2	2	2	2	4	1	4	5	3
GN0586	2	6	1	2	2	4	2	2	2	3	2	0	1	3	7	1
GN0587	2	3	1	2	3	4	2	5	3	2	3	0	1	2	7	3
GN0589	6	5	1	2	2	4	2	5	2	1	3	0	1	3	7	9
GN0590	2	6	2	2	3	4	1	2	2	2	3	0	1	3	9	1
GN0591	2	6	1	2	3	4	2	2	2	1	3	2	1	3	9	5
GN0592	2	2	1	2	3	2	2	2	2	3	4	0	1	3	4	3
GN0593	6	5	2	2	3	4	2	5	3	1	2	0	1	6	3	5
GN0595	6	3	2	2	2	4	1	5	2	3	3	0	2	6	8	9
GN0597	5	3	2	2	2	2	1	2	2	3	2	0	1	7	9	2
GN0602	6	6	1	2	3	4	1	2	3	3	2	0	1	6	4	2
GN0605	2	3	1	2	3	4	0	2	3	3	3	2	3	6	8	1
GN0606	2	6	2	2	3	4	0	2	2	3	3	0	2	3	1	1
GN0607	2	6	2	2	2	4	1	2	1	3	3	2	1	3	2	2
GN0608	6	6	1	2	3	4	1	2	2	2	2	0	1	3	5	1
GN0611	2	6	2	2	2	4	1	2	1	2	3	0	2	6	4	1
GN0612	7	3	2	2	2	3	2	2	2	2	3	3	2	3	4	5
GN0613	2	6	1	2	3	4	1	2	2	2	3	0	1	4	9	5
GN0617	6	6	1	2	3	4	2	5	2	3	2	0	1	6	7	4
GN0618	7	6	2	1	2	4	1	5	1	2	3	0	2	3	4	3
GN0619	2	6	2	2	3	3	1	2	2	1	2	0	3	6	7	1
GN0620	2	6	2	2	3	4	1	2	2	3	3	0	2	3	9	1
GN0622	2	6	1	2	3	4	1	2	2	3	3	0	1	2	1	1
GN0624	6	6	1	2	2	4	1	5	1	3	3	0	4	3	4	3
GN0630	6	3	1	2	3	2	0	2	2	2	3	0	3	2	4	3
GN0633	6	6	1	2	3	3	1	5	3	3	2	0	1	4	6	3
GN0636	2	3	2	2	2	2	1	2	2	2	2	0	1	3	7	4
GN0637	6	6	1	2	3	4	3	2	2	3	3	0	2	4	6	1
GN0638	2	3	2	2	3	4	3	2	2	3	2	0	1	4	9	3
GN0639	2	6	2	2	2	4	3	2	2	2	4	0	3	3	9	3
GN0641	6	3	1	2	3	2	1	2	2	2	3	0	3	1	2	1
GN0643	7	6	1	2	2	4	2	2	2	3	3	-	2	3	7	1
GN0644	6	6	1	2	2	4	2	5	2	2	3	0	2	3	7	3

GN0646	2	6	1	2	3	4	1	7	2	3	3	0	3	3	9	3
GN0648	2	6	1	2	3	4	2	7	2	3	2	0	1	3	4	5
GN0649	2	6	2	2	3	4	0	7	2	2	2	3	2	3	1	1
GN0650	6	6	1	2	2	2	3	2	2	2	3	0	2	3	9	2
GN0651	2	6	2	2	2	4	1	2	2	3	2	0	2	6	1	1
GN0652	2	3	2	2	3	2	3	2	2	3	3	0	2	1	7	3
GN0653	2	6	2	2	2	4	1	2	2	3	2	0	2	3	9	4
GN0656	2	6	1	1	2	4	3	2	2	4	3	0	2	3	4	3
GN0658	2	6	1	2	2	4	2	2	2	3	3	0	2	6	8	3
GN0659	2	6	1	2	3	4	2	2	2	3	3	0	2	3	4	3
GN0661	7	6	2	2	3	4	1	2	2	3	3	0	2	3	4	3
GN0662	2	3	1	2	2	2	3	2	2	4	3	0	2	4	9	1
GN0663	2	3	1	1	2	4	1	2	2	3	3	0	2	2	4	3
GN0664	6	6	1	2	3	4	1	7	2	3	3	0	2	6	1	1
GN0665	2	6	1	2	3	4	1	2	2	4	3	3	1	3	5	1
GN0667	7	6	1	2	2	4	1	2	1	3	3	3	3	3	4	2
GN0672	1	6	2	2	3	4	2	2	2	3	3	0	2	1	2	1
GN0673	2	2	2	2	3	2	0	2	3	3	2	2	2	3	5	1
GN0677	6	6	2	2	3	4	1	2	2	4	3	0	2	6	6	3
GN0678	6	5	1	2	2	4	1	2	2	2	2	3	1	6	6	2
GN0679	2	3	1	2	2	2	0	2	2	2	4	0	2	1	4	1
GN0680	2	6	2	1	2	4	2	2	2	3	3	0	1	2	7	5
GN0681	6	6	2	2	3	2	0	2	2	2	2	2	1	3	4	3
GN0682	2	6	2	2	3	4	0	2	2	2	2	3	1	3	4	3
GN0683	6	6	2	2	3	4	0	2	1	3	3	3	2	3	6	3
GN0684	2	3	-	2	2	4	1	2	2	2	3	0	2	3	1	1
GN0686	2	6	1	2	2	4	0	2	1	3	3	3	3	4	8	1
GN0687	6	6	2	2	3	4	0	2	2	2	3	3	2	3	6	1
GN0688	2	6	1	2	3	3	1	2	2	2	3	0	2	6	4	1
GN0689	6	6	2	2	2	2	0	2	2	3	2	3	1	3	4	9
GN0690	2	5	2	2	3	4	2	2	2	3	3	0	1	3	8	4
GN0691	6	6	1	2	3	4	2	2	2	3	2	0	2	4	4	2
GN0693	6	6	1	2	3	2	0	2	2	3	3	0	2	4	4	3
GN0695	2	6	2	2	2	4	1	2	1	4	3	0	2	3	1	1
GN0696	2	6	2	2	2	4	0	5	1	3	3	0	2	3	4	3
GN0701	6	6	2	2	3	4	1	2	2	1	3	3	3	4	7	3
GN0706	2	6	1	2	3	4	0	2	2	3	2	0	1	6	9	1
GN0707	7	6	1	2	2	4	0	2	2	3	3	3	2	3	4	4
GN0709	7	6	1	2	3	4	1	2	2	2	3	3	2	3	8	2
GN0711	6	6	2	2	3	4	3	5	2	1	3	2	1	3	4	3
GN0713	2	6	2	2	3	2	1	2	2	3	3	0	2	3	2	2
GN0714	2	6	2	2	3	4	2	2	2	2	3	3	1	6	6	1
GN0717	6	6	1	2	3	4	3	2	2	2	3	0	2	6	4	3
GN0718	6	6	1	2	3	4	0	2	2	3	2	0	2	6	4	9

GN0724	2	6	1	2	2	4	1	5	2	2	3	-	2	3	9	1
GN0731	6	6	1	2	3	4	0	2	2	3	3	0	3	3	7	2
GN0736	2	6	2	2	3	4	0	2	2	2	2	0	1	3	8	1
GN0740	1	3	1	2	2	4	1	5	2	2	3	0	3	3	4	3
GN0741	2	6	2	2	3	4	0	2	2	2	4	2	2	6	1	1
GN0742	7	3	1	2	2	4	0	5	1	1	4	3	3	2	8	1
GN0743	2	6	1	2	3	4	0	2	2	2	2	2	1	3	4	1
GN0744	6	6	1	2	3	4	0	5	2	2	3	0	1	6	2	3
GN0745	2	6	1	2	3	4	0	2	2	1	3	3	2	3	2	1
GN0747	2	6	2	2	2	4	1	2	1	3	4	0	3	4	7	1
GN0749	2	6	1	2	1	4	0	5	1	3	3	-	2	6	1	2
GN0750	6	6	1	2	3	4	0	2	2	3	2	2	2	6	9	1
GN0754	1	6	1	1	3	2	1	2	2	3	2	0	2	7	1	1
GN0756	2	3	1	2	2	2	0	2	2	2	3	0	2	4	7	1
GN0757	2	3	1	2	3	4	3	2	2	2	3	0	3	4	9	1
GN0758	6	6	2	5	2	4	0	2	2	3	3	-	2	2	6	1
GN0760	6	3	1	5	3	4	0	2	2	2	2	0	2	7	7	1
GN0761	1	5	1	1	3	4	1	5	2	3	3	0	2	3	7	2
GN0762	2	3	2	2	3	2	1	2	3	3	2	2	1	3	1	3
GN0763	2	3	2	2	2	3	2	5	2	2	2	0	1	3	3	3
GN0764	1	3	2	1	2	2	3	2	2	3	2	0	2	3	4	3
GN0766	2	3	1	2	2	2	1	2	2	3	2	2	1	6	9	4
GN0767	2	3	1	2	2	4	2	5	2	1	3	0	1	3	9	3
GN0770	2	6	2	2	2	4	1	7	1	3	3	2	3	3	7	5
GN0773	2	3	0	2	2	4	1	2	2	2	2	2	1	6	1	2
GN0774	2	3	2	2	3	4	1	5	2	2	3	0	2	3	4	5
GN0775	2	6	0	1	2	2	1	2	2	2	3	0	3	3	7	5
GN0777	2	6	2	2	2	3	3	2	2	3	3	0	1	3	5	5
GN0778	2	6	2	1	3	4	1	3	2	3	3	0	2	3	5	5
GN0783	2	3	2	2	3	4	0	2	3	3	3	0	2	3	2	2
GN0786	1	6	1	2	2	2	2	2	2	2	3	0	3	3	3	5
GN0789	2	6	1	4	2	4	1	5	2	2	3	3	3	3	2	3
GN0793	2	3	2	2	3	4	1	2	2	2	3	2	3	3	7	3
GN0794	6	6	2	2	3	2	3	7	2	1	4	0	4	3	9	1
GN0796	6	6	1	2	2	2	3	2	3	3	2	0	2	4	7	1
GN0797	1	6	2	1	2	3	2	2	2	2	3	0	3	7	2	1
GN0798	2	6	1	1	2	4	1	2	2	3	3	2	2	5	9	1
GN0800	2	3	1	2	3	4	1	2	3	3	2	0	2	6	4	9
GN0802	2	6	1	2	2	2	2	2	2	3	3	0	2	7	8	3
GN0804	1	6	0	2	2	3	2	2	2	3	3	2	2	3	8	2
GN0806	1	6	2	2	3	5	1	2	2	2	3	0	3	3	1	2
GN0807	4	6	2	2	3	4	3	5	2	3	3	0	3	3	6	3
GN0811	1	3	1	2	3	4	3	5	2	2	3	0	2	3	2	2
GN0812	7	6	2	2	2	3	0	2	3	2	3	0	4	3	1	3

GN0813	9	6	2	2	3	4	1	2	2	2	3	0	2	3	2	1
GN0814	6	6	2	2	2	3	0	2	3	3	3	0	2	3	9	1
GN0815	2	3	1	2	2	4	2	5	2	3	2	0	2	3	2	1
GN0816	2	6	1	2	2	2	0	2	1	3	3	0	1	3	1	2
GN0817	2	6	2	2	2	4	2	2	3	2	2	0	2	2	4	1
GN0819	2	6	2	2	2	4	0	2	2	2	3	0	2	3	4	2
GN0820	2	6	1	2	2	2	3	2	2	2	2	0	2	3	7	5
GN0822	2	6	1	2	2	5	1	5	2	2	3	0	2	3	4	2
GN0823	7	6	2	2	3	2	3	2	3	2	1	4	1	4	7	5
GN0824	1	3	1	1	2	4	1	2	3	3	2	2	2	3	2	2
GN0825	2	6	2	1	2	2	1	2	2	2	3	4	2	3	6	5
GN0827	2	6	1	2	2	4	2	5	3	2	2	0	2	2	7	2
GN0828	1	6	1	2	2	4	0	5	2	2	2	0	2	5	7	1
GN0830	1	6	2	2	2	5	0	2	2	2	2	0	1	6	6	5
GN0834	2	6	1	2	3	4	0	2	2	2	2	0	3	4	1	1
GN0835	2	3	1	4	3	4	0	1	2	3	3	2	2	2	7	2
GN0837	2	3	1	2	3	4	0	2	3	3	2	2	2	2	4	3
GN0839	2	6	2	2	2	3	0	2	2	2	3	0	4	0	9	1
GN0840	2	3	2	2	2	4	0	5	3	3	3	0	1	6	7	3
GN0844	2	3	1	2	3	3	0	2	2	3	3	0	2	3	1	1
GN0845	6	5	2	2	3	4	0	2	3	3	3	2	2	6	1	1
GN0847	2	3	1	2	2	2	0	2	2	3	3	0	2	5	9	1
GN0849	2	3	1	2	2	3	0	2	2	3	3	3	1	0	7	3
GN0852	2	6	1	2	2	3	0	2	2	3	3	0	3	6	7	4
GN0853	2	6	1	2	3	3	1	2	2	3	2	0	1	5	9	4
GN0854	2	3	2	2	3	4	1	2	3	3	2	0	2	6	8	4
GN0856	6	3	1	2	3	4	3	2	3	3	2	0	2	4	4	3
GN0858	2	6	2	2	3	4	1	2	2	3	2	0	1	3	4	3
GN0860	2	3	2	2	3	4	1	2	2	3	2	0	2	4	6	3
GN0864	2	3	2	2	3	4	3	2	3	2	2	0	2	6	7	1
GN0865	7	3	2	2	3	4	0	2	2	3	2	0	1	3	6	4
GN0865	1	6	2	2	2	4	1	2	2	1	2	3	1	6	8	9
GN0866	6	6	2	6	3	5	0	5	2	3	2	4	1	3	6	3
GN0867	1	3	1	2	3	4	1	2	3	3	2	0	2	6	9	2
GN0868	2	6	1	2	3	4	0	2	2	3	3	2	1	3	4	3
GN0870	7	3	1	2	2	4	0	2	2	2	2	4	2	3	2	1
GN0872	5	6	1	2	3	4	2	5	2	3	2	0	3	6	6	2
GN0874	2	3	1	2	3	2	1	2	2	3	2	3	2	3	6	4
GN0875	6	3	1	3	3	4	0	2	2	2	3	0	1	3	9	3
GN0877	2	3	2	2	3	3	1	2	3	2	3	0	2	3	1	1
GN0882	2	6	1	2	3	3	0	2	2	3	3	2	2	4	4	1
GN0884	2	6	1	2	2	4	2	2	2	2	2	0	2	7	3	4
GN0885	2	6	1	2	3	2	0	2	2	3	2	0	1	6	7	3
GN0886	7	6	1	2	3	2	0	2	3	3	3	0	1	4	7	2

GN0888	2	6	1	2	2	4	2	2	2	2	3	0	3	3	6	3
GN0889	2	4	1	2	3	3	0	2	2	3	2	3	2	6	6	2
GN0891	6	4	1	2	3	4	0	5	3	3	2	2	1	3	9	3
GN0895	6	3	1	2	3	4	1	5	3	3	2	2	1	3	9	2
GN0900	2	3	2	2	3	4	2	2	3	3	2	0	2	6	9	3
GN0902	6	6	1	2	3	4	0	5	3	3	2	3	1	5	9	3
GN0905	2	3	1	3	3	4	0	2	3	3	3	0	1	5	9	3
GN0908	2	6	2	2	3	4	2	2	2	1	3	4	1	0	4	9
GN0909	2	3	2	2	3	4	0	2	2	3	3	0	2	3	9	1
GN0910	2	3	2	2	3	4	2	2	3	3	3	0	2	8	8	1
GN0913	6	3	1	3	3	2	1	5	2	2	3	0	2	3	8	4
GN0914	6	4	1	3	2	3	1	5	2	3	3	0	2	3	2	3
GN0916	5	6	1	2	3	4	1	2	2	3	3	0	2	3	3	4
GN0917	6	6	1	2	2	4	3	5	2	2	2	2	2	7	2	2
GN0919	2	6	1	2	1	4	1	2	2	2	3	0	2	3	6	3
GN0923	1	6	1	2	2	3	1	2	2	3	3	0	3	2	8	1
GN0925	6	6	1	2	2	4	1	5	2	3	3	0	2	4	5	1
GN0926	6	6	2	2	3	4	0	2	3	3	2	2	2	7	1	1
GN0928	2	6	1	2	3	2	1	2	3	3	2	2	2	3	1	1
GN0931	2	3	1	2	3	2	2	2	2	3	3	2	2	4	4	2
GN0932	2	6	2	2	3	4	2	2	2	4	3	0	2	4	7	2
GN0933	2	3	2	2	2	4	3	2	2	3	2	0	2	3	4	4
GN0934	6	6	2	2	3	4	1	5	3	3	3	0	3	3	6	1
GN0935	2	4	2	2	3	4	1	2	2	4	2	0	2	4	7	1
GN0936	6	6	2	2	3	4	0	2	2	3	2	2	2	3	6	1
GN0944	2	3	2	2	3	3	0	5	3	1	2	0	1	6	8	1
GN0945	2	6	2	2	3	4	2	2	2	2	3	2	1	6	1	1
GN0946	2	3	1	3	3	4	1	5	2	2	3	0	2	8	7	1
GN0947	6	3	1	3	3	2	2	5	3	2	2	0	2	3	9	3
GN0948	2	3	2	2	3	4	2	2	3	2	3	3	2	6	1	2
GN0949	6	6	1	2	3	4	0	2	2	2	3	0	2	6	4	1
GN0950	2	3	2	2	3	3	0	2	3	2	2	3	1	6	6	2
GN0952	2	4	1	4	3	4	0	2	2	2	3	3	3	6	7	2
GN0954	2	6	2	2	3	4	1	2	2	2	3	0	2	-	8	1
GN0956	2	3	1	4	2	2	0	2	2	2	3	2	2	-	7	3
GN0957	2	3	2	2	2	4	0	2	2	2	2	0	1	6	2	2
GN0959	2	3	2	3	3	5	1	3	3	1	3	0	2	3	9	3
GN0963	6	3	1	2	2	4	0	5	2	3	3	0	2	6	8	5
GN0965	2	6	1	2	3	3	0	7	3	2	3	0	2	2	8	3
GN0972	1	3	1	2	2	4	0	1	2	3	3	0	3	4	6	3
GN0973	2	3	1	2	2	4	1	2	2	3	3	0	3	4	8	1
GN0974	6	6	2	2	1	4	0	2	1	2	3	0	3	7	6	2
GN0975	2	3	1	2	1	4	1	2	1	3	3	2	3	7	7	2
GN0976	2	6	1	2	2	4	1	2	2	1	3	3	2	4	4	3

GN0977	2	3	2	2	3	3	1	2	2	3	3	2	1	3	6	3
GN0978	6	6	1	2	2	4	0	2	2	2	3	0	4	3	7	1
GN0979	6	3	2	2	1	2	0	2	1	2	3	0	4	6	2	1
GN0980	2	3	2	1	1	4	1	2	2	2	3	0	4	7	7	1
GN0982	2	6	1	2	3	2	0	2	2	2	3	4	2	6	4	3
GN0984	2	6	1	2	1	2	1	2	2	2	3	3	2	7	7	1
GN0985	6	6	2	2	1	4	1	2	2	2	2	0	3	7	1	2
GN0986	2	6	1	1	3	4	0	2	2	3	2	0	2	3	2	2
GN0987	2	6	1	2	1	4	0	2	2	2	2	0	2	7	8	2
GN0990	6	6	2	1	3	4	1	5	2	3	2	0	2	3	7	2
GN0991	2	6	2	2	2	4	0	2	2	2	2	0	1	4	4	2
GN0992	6	6	1	2	1	4	1	5	2	2	3	0	3	7	4	3
GN0994	2	3	2	2	2	4	0	2	2	3	3	2	3	2	6	1
GN0995	2	6	2	2	3	4	1	2	3	3	2	0	2	4	1	3
GN1000	1	6	1	1	2	3	2	2	2	3	3	0	2	4	2	3
GN1002	2	6	1	2	3	4	1	2	2	3	3	0	3	4	8	1
GN1004	5	6	1	1	2	4	1	2	3	3	3	0	2	3	8	1
GN1005	2	6	1	2	2	3	1	2	3	2	3	0	4	4	8	3
GN1006	6	6	1	2	2	3	2	2	2	1	3	2	2	3	8	1
GN1007	6	6	2	2	2	4	1	2	1	2	3	2	4	3	8	2
GN1010	6	6	2	2	2	4	1	5	2	2	3	2	2	3	5	1
GN1011	2	3	1	2	3	4	1	2	3	3	3	0	2	4	8	1
GN1013	5	6	1	2	3	4	2	2	3	3	3	0	2	4	1	1
GN1014	5	6	1	2	3	4	2	5	2	2	3	0	2	4	1	1
GN1016	5	4	2	2	3	3	2	2	3	2	2	0	1	6	2	2
GN1018	2	3	1	2	2	2	1	2	2	2	2	0	3	6	7	2
GN1019	2	6	2	2	3	4	1	2	2	3	3	2	2	4	7	1
GN1020	2	3	1	2	3	4	1	2	2	2	2	0	2	6	7	4
GN1022	4	3	-	2	3	5	2	2	2	4	3	2	1	6	8	2
GN1026	6	3	2	2	2	2	1	2	3	2	2	0	2	3	4	5
GN1027	2	6	2	2	1	4	2	2	2	3	3	4	1	3	9	9
GN1028	6	3	1	2	3	4	3	2	2	1	3	4	4	3	4	2
GN1029	2	4	1	2	3	4	3	2	3	2	2	2	2	6	8	1
GN1032	2	3	2	2	3	2	1	2	2	3	2	2	2	4	7	2
GN1035	2	6	2	2	3	2	2	2	2	4	3	0	3	7	3	4
GN1038	6	3	1	3	3	4	3	2	4	2	3	2	2	6	4	3
GN1039	1	6	1	2	3	4	2	2	3	2	3	4	2	3	4	2
GN1040	2	6	2	2	3	4	1	2	3	1	3	0	1	6	5	2
GN1043	2	3	1	2	3	4	2	2	3	2	3	0	3	3	4	3
GN1044	2	3	1	2	2	2	1	2	3	3	2	0	1	3	4	3
GN1045	2	6	2	2	2	4	2	5	2	2	3	0	1	2	7	4
GN1046	5	6	2	2	3	2	2	2	2	3	3	0	1	3	7	5
GN1047	2	6	1	2	2	3	0	2	2	1	3	2	1	6	1	2
GN1048	2	6	2	2	3	4	1	2	3	2	3	0	1	3	1	2

GN1049	2	3	2	2	3	4	1	5	2	1	3	2	1	6	6	2
GN1050	2	6	2	2	2	3	1	2	3	2	4	3	1	6	9	3
GN1053	5	3	2	1	3	4	2	2	3	2	2	0	2	0	8	1
GN1055	6	3	1	4	3	4	2	2	3	3	3	0	2	3	7	3
GN1056	6	3	1	2	3	3	0	2	2	2	2	0	4	3	6	2
GN1057	2	6	1	2	3	4	3	5	3	3	2	2	2	6	2	2
GN1058	6	3	1	2	3	4	1	5	3	2	3	0	2	6	6	3
GN1061	5	6	2	2	2	3	0	2	2	3	3	2	2	3	4	2
GN1063	1	3	2	2	2	2	2	2	2	-	2	2	2	3	4	4
GN1064	5	6	1	2	3	4	0	5	3	2	2	0	2	3	1	1
GN1065	2	3	1	2	3	2	0	2	2	2	3	2	2	4	6	2
GN1066	2	3	2	2	2	4	1	5	2	2	2	2	1	6	7	5
GN1068	2	6	1	2	3	5	1	3	2	2	3	0	2	3	4	5
GN1069	6	3	2	2	3	2	1	2	3	2	3	0	2	5	7	3
GN1070	2	6	1	2	2	4	3	2	2	2	2	4	1	3	4	3
GN1073	2	3	2	2	3	4	1	2	2	2	2	4	2	3	1	1
GN1074	2	6	2	2	3	4	0	2	2	3	2	0	1	6	4	1
GN1075	2	3	2	2	3	2	1	5	2	3	3	2	2	4	7	2
GN1078	2	3	1	2	3	2	1	2	2	2	3	3	2	1	4	3
GN1079	7	6	1	2	3	2	0	2	3	3	2	0	1	3	6	2
GN1080	7	3	2	2	3	4	0	2	2	2	3	2	2	5	2	9
GN1086	2	4	1	2	3	4	1	2	1	3	3	0	2	6	5	7
GN1087	2	6	1	2	2	4	1	2	2	2	3	0	2	3	7	3
GN1089	6	3	1	2	2	2	0	2	1	2	2	0	1	0	4	3
GN1091	6	3	2	2	3	2	1	2	1	2	3	0	2	5	9	3
GN1092	6	4	1	2	3	4	3	2	2	2	3	2	1	3	3	4
GN1094	2	6	2	4	3	2	1	2	2	2	3	0	2	3	9	1
GN1097	2	6	2	2	3	3	1	2	1	2	3	0	2	3	9	3
GN1098	1	3	2	2	2	2	0	2	1	1	3	0	2	6	1	3
GN1104	2	3	1	2	2	4	1	5	1	4	2	0	2	3	2	4
GN1106	2	3	2	2	3	4	1	2	1	3	3	0	2	6	7	3
GN1107	2	3	1	2	1	5	0	2	2	2	3	0	4	7	2	2
GN1110	7	3	1	2	3	2	2	2	1	3	3	0	2	6	8	3
GN1111	2	6	2	2	3	4	0	2	1	2	3	2	1	3	1	1
GN1116	2	6	2	2	3	4	0	2	2	3	2	0	2	6	2	9
GN1119	2	3	1	2	2	4	0	2	2	2	2	3	2	3	7	4
GN1120	6	6	1	6	3	4	0	5	3	2	3	0	2	3	1	9
GN1130	2	6	1	2	2	5	1	2	2	2	2	2	2	3	4	4
GN1131	6	5	1	2	2	2	1	2	2	2	2	3	1	6	4	1
GN1132	2	6	1	2	2	2	2	2	2	3	2	0	2	3	1	1
GN1134	2	6	2	2	2	4	0	2	2	2	3	0	1	3	4	3
GN1135	5	6	2	2	3	4	1	2	2	3	3	0	2	6	4	3
GN1136	6	3	1	2	2	5	1	6	2	2	3	0	1	3	7	3
GN1138	6	3	2	2	3	4	3	2	2	2	3	0	2	6	4	3

GN1139	2	6	2	2	2	4	1	2	2	2	3	0	2	3	6	2
GN1140	2	3	2	2	2	4	2	2	2	3	2	0	2	6	7	5
GN1141	2	3	2	2	2	4	1	2	2	2	3	2	2	3	9	3
GN1142	2	6	2	2	3	4	0	2	2	2	2	2	3	0	7	1
GN1143	2	6	2	2	3	4	3	2	2	2	2	0	4	0	7	1
GN1144	2	3	1	2	3	2	2	2	2	2	3	0	3	6	1	2
GN1145	6	3	1	2	2	2	1	2	2	2	3	0	2	3	7	5
GN1146	7	3	2	2	3	2	3	2	2	2	3	0	3	3	1	2
GN1149	2	3	1	2	2	4	0	4	2	2	2	0	1	4	2	9
GN1150	2	6	1	2	3	2	0	2	3	2	3	3	2	3	8	2
GN1153	2	6	2	2	2	5	0	2	3	2	2	4	2	6	1	1
GN1157	6	3	2	2	3	5	3	6	3	2	3	0	2	4	7	1
GN1158	6	3	1	2	2	2	0	2	2	2	2	0	1	6	4	3
GN1162	2	3	2	2	3	3	2	2	2	2	2	0	3	6	1	9
GN1163	2	3	1	2	3	4	3	5	2	3	3	0	4	6	4	9
GN1164	1	6	2	2	3	4	0	2	2	3	2	0	3	4	1	1
GN1165	2	6	2	2	3	4	3	2	3	2	3	0	3	3	8	1
GN1167	2	6	2	2	3	4	2	2	1	3	3	0	4	6	8	1
GN1168	6	6	1	2	3	5	1	6	2	3	2	3	2	3	9	9
GN1169	2	6	1	2	3	4	1	2	2	3	3	3	3	6	8	1
GN1171	6	6	2	2	3	4	1	2	2	3	3	0	3	6	1	1
GN1174	6	5	1	2	3	4	1	2	2	3	3	0	3	3	7	2
GN1177	2	6	1	2	3	4	2	2	2	3	3	0	3	3	8	1
GN1178	2	3	1	2	2	5	2	4	2	2	3	0	2	3	9	1
GN1186	2	6	2	2	3	2	1	5	2	2	3	0	1	3	8	9
GN1187	2	3	1	2	3	2	0	2	3	2	2	0	2	6	4	2
GN1188	2	3	2	2	3	4	1	2	2	1	3	0	1	3	1	1
GN1189	2	5	1	2	2	4	0	5	2	2	3	0	1	3	2	2
GN1191	2	6	1	2	3	5	1	6	3	2	2	2	2	3	3	5
GN1195	2	3	1	2	3	2	1	2	2	2	3	3	2	3	1	2
GN1196	2	6	1	2	3	4	1	2	2	2	2	4	1	4	2	5
GN1198	2	6	2	2	3	5	1	2	3	3	3	2	1	3	1	1
GN1199	2	3	2	2	2	2	0	2	1	3	3	2	1	7	7	5
GN1200	2	6	1	2	3	4	0	2	1	3	3	2	1	5	8	9
GN1201	2	6	2	2	3	2	1	2	2	2	3	0	1	3	3	5
GN1202	6	6	2	2	3	4	0	4	2	2	3	4	2	7	8	4
GN1204	2	3	1	2	3	2	0	2	2	2	3	2	1	3	3	5
GN1205	7	3	1	2	2	4	1	2	2	2	3	3	2	3	4	2
GN1206	6	3	1	2	2	4	0	5	1	2	3	3	2	2	4	9
GN1208	2	3	1	2	3	3	0	2	3	1	3	2	1	3	7	1
GN1209	2	3	1	5	3	4	0	5	2	2	3	2	1	4	4	4
GN1210	2	6	2	2	2	4	0	2	2	3	2	2	1	7	4	5
GN1211	7	3	1	2	3	3	2	2	2	1	3	3	1	6	7	9
GN1215	7	3	2	2	2	4	0	2	1	1	3	3	1	3	7	5

GN1217	6	6	1	2	3	4	0	4	2	2	2	3	1	2	6	5
GN1218	2	6	1	2	3	4	1	2	2	3	2	3	1	7	4	1
GN1219	5	3	2	5	2	4	0	2	2	2	2	0	1	6	6	4
GN1222	2	6	2	2	2	4	0	2	1	1	3	4	2	3	5	5
GN1223	2	3	2	2	2	4	0	2	1	1	3	4	2	3	1	2
GN1225	2	3	1	2	2	2	0	2	1	1	3	4	1	2	2	3
GN1227	2	6	2	5	2	2	0	2	1	2	2	3	2	6	4	3
GN1228	6	3	1	5	3	2	0	2	2	2	2	2	1	6	7	4
GN1229	2	4	2	2	2	4	1	2	2	1	3	4	1	6	9	9
GN1230	2	1	2	4	3	4	2	2	2	2	2	4	1	3	4	5
GN1232	2	6	1	2	3	2	0	2	2	2	3	2	2	3	8	1
GN1233	9	3	1	5	2	3	0	2	1	1	3	2	3	0	5	4
GN1234	6	6	1	5	3	2	0	2	1	2	2	2	1	3	8	5
GN1237	2	3	2	2	2	4	0	2	2	1	2	0	1	6	7	5
GN1239	2	3	2	2	2	4	0	2	2	2	3	0	2	6	8	5
GN1245	2	6	-	2	3	2	0	2	2	3	2	0	1	3	8	4
GN1246	2	3	2	2	3	4	0	5	2	1	3	0	1	3	2	1
GN1249	2	6	1	2	3	4	2	4	2	2	2	2	1	6	1	2
GN1250	2	6	2	2	2	3	3	7	2	3	2	3	2	3	7	3
GN1251	2	3	1	2	2	4	1	2	1	1	3	3	1	6	7	5
GN1252	2	3	2	2	3	4	0	2	2	3	2	2	1	3	7	2
GN1253	2	3	2	2	3	4	0	2	2	3	2	2	1	8	8	3
GN1257	6	3	-	2	2	4	1	5	2	1	3	0	1	3	8	2
GN1258	2	4	1	2	3	4	1	2	2	2	3	0	2	3	7	2
GN1259	2	3	-	2	3	4	2	2	2	2	3	0	2	6	7	1
GN1262	2	6	1	2	1	4	0	2	1	1	3	2	1	0	5	1
GN1263	6	3	1	2	3	4	0	2	1	1	3	2	2	3	8	2
GN1264	5	3	2	3	3	4	0	2	2	2	4	0	2	6	4	3
GN1266	2	6	2	2	1	2	0	2	1	2	4	0	4	0	8	4
GN1269	5	4	1	3	3	4	0	2	2	3	2	2	1	6	7	4
GN1270	2	6	2	3	1	5	0	2	1	1	3	3	1	0	8	5
GN1273	2	3	2	2	3	4	0	2	2	2	3	0	2	6	8	3
GN1276	2	6	1	2	2	4	1	5	2	2	3	2	1	2	7	2
GN1277	6	3	1	2	2	4	1	2	2	1	3	3	1	7	4	3
GN1278	2	6	1	2	3	2	0	2	2	2	2	2	1	3	7	4
GN1279	2	6	1	2	3	4	0	2	1	3	3	0	1	3	2	2
GN1280	2	6	1	2	3	2	1	2	2	2	2	2	1	3	8	4
GN1281	2	3	1	2	2	4	0	2	1	2	3	0	2	3	7	4
GN1282	2	6	-	2	3	4	1	2	2	2	2	3	1	2	9	5
GN1283	2	3	1	2	2	4	1	2	1	3	2	2	2	4	7	1
GN1284	2	6	1	2	3	4	2	2	2	1	3	0	1	7	8	1
GN1285	2	3	1	2	2	4	2	2	2	1	3	0	1	5	8	3
GN1286	2	3	1	2	2	3	2	2	2	1	3	0	2	3	8	3
GN1287	2	6	2	2	2	4	2	2	1	2	3	0	1	6	6	3

GN1288	2	3	1	2	3	2	0	2	2	2	2	0	1	7	8	2
GN1289	2	3	1	2	2	4	1	2	1	2	3	2	1	3	7	3
GN1290	2	4	1	2	2	4	2	2	1	3	2	0	1	3	7	2
GN1292	6	6	1	2	1	2	3	2	1	3	3	3	2	7	4	5
GN1293	6	6	2	3	2	2	3	2	1	2	2	2	1	3	8	2
GN1295	4	3	2	2	2	2	2	2	1	2	3	2	1	2	4	5
GN1296	2	3	2	2	2	4	3	2	2	2	2	4	1	6	5	2
GN1297	2	3	2	2	2	4	3	2	1	1	3	3	1	6	7	4
GN1300	2	6	-	2	3	2	0	2	1	2	3	2	2	5	2	2
GN1304	2	6	-	2	3	4	1	2	2	2	3	2	1	4	8	3
GN1306	2	3	-	2	2	4	2	2	2	2	3	2	3	3	8	9
GN1308	2	6	-	2	3	4	2	2	2	1	3	2	1	6	7	2
GN1311	7	6	-	2	3	2	2	2	3	3	2	2	2	7	7	4
GN1312	6	3	-	2	3	3	1	2	2	2	3	2	1	2	4	5
GN1315	2	3	-	2	3	4	2	5	2	2	3	2	4	2	3	5
GN1316	2	6	-	2	3	2	0	2	2	4	2	2	1	6	7	2
GN1317	2	6	-	2	3	4	0	5	3	3	3	2	2	3	6	4
GN1318	2	6	-	2	3	4	0	5	2	3	2	2	2	3	7	4
GN1319	2	6	-	2	3	3	2	2	3	2	3	0	1	3	8	3
GN1321	6	6	-	3	3	4	2	2	2	2	3	0	2	5	4	3
GN1323	6	3	-	9	3	5	1	3	3	2	3	0	3	3	7	1
GN1325	2	3	-	4	3	4	1	2	2	4	3	0	2	7	7	4
GN1327	2	6	-	2	2	2	2	2	2	3	2	0	2	2	1	1
GN1329	2	6	-	2	3	4	1	5	2	3	3	0	2	6	7	2
GN1331	2	6	-	2	3	4	0	2	3	2	2	0	1	3	7	5
GN1333	6	3	-	3	3	4	2	2	2	3	2	2	2	5	7	2
GN1334	7	3	-	2	3	5	1	2	3	2	3	2	2	5	8	3
GN1336	5	6	-	2	3	4	1	2	2	2	3	3	1	3	9	3
GN1337	6	3	-	3	2	4	2	2	2	2	3	3	2	2	2	3
GN1338	2	3	-	2	2	2	1	2	2	2	3	0	2	3	9	9
GN1339	2	3	-	2	3	4	-	2	2	3	3	2	2	6	8	9
GN1340	2	6	-	4	2	5	1	2	2	4	2	0	3	4	7	2
GN1342	2	3	-	2	2	5	2	2	3	2	3	0	2	4	4	4
GN1345	2	6	-	2	2	5	2	2	2	3	3	0	3	3	2	2
GN1346	2	3	-	2	2	2	2	2	2	3	2	0	2	4	7	3
GN1347	2	6	-	2	2	4	2	2	2	3	2	3	2	4	2	5
GN1348	2	6	-	2	3	4	2	2	2	3	3	0	2	3	4	4
GN1349	7	6	-	2	3	4	2	5	3	3	3	2	3	4	7	3
GN1350	2	6	-	2	2	4	1	2	2	3	3	4	2	4	8	3
GN1351	5	6	-	2	2	4	3	2	2	4	3	3	2	3	8	1
GN1352	6	6	-	3	2	2	1	2	2	2	3	0	2	6	9	9
GN1353	5	6	-	2	3	2	0	2	2	3	2	0	3	3	2	2
GN1354	2	3	-	2	3	5	0	2	2	3	3	3	2	3	5	2
GN1355	5	6	-	2	3	4	1	2	2	3	3	0	2	6	8	9

GN1356	2	6	－	2	3	4	0	2	2	3	2	2	2	3	4	4
GN1358	6	3	－	3	2	5	0	2	2	3	2	4	2	3	7	3
GN1359	2	6	－	2	2	5	2	2	2	1	3	0	2	3	2	2
GN1360	6	6	－	2	2	2	3	2	2	2	3	0	2	3	8	2
GN1361	2	6	－	2	3	2	1	2	2	2	3	3	2	3	2	4
GN1363	2	3	－	2	2	5	1	2	2	3	3	2	3	3	6	4
GN1364	2	3	－	2	1	5	1	2	2	2	3	2	2	3	6	4
GN1366	2	6	－	2	2	4	1	2	2	2	3	2	2	3	2	1
GN1367	2	3	－	2	2	5	1	2	2	3	3	2	2	4	6	2
GN1368	2	6	－	2	2	5	1	2	3	3	3	3	2	7	7	1
GN1376	1	4	－	2	2	5	1	2	2	3	3	0	2	4	7	2
GN1377	2	6	－	2	2	3	0	2	2	3	3	2	1	4	8	1
GN1381	2	6	－	2	2	5	3	2	2	4	3	0	3	4	7	2
GN1384	1	6	－	2	2	4	3	2	2	4	3	0	2	3	2	3
GN1385	1	3	－	2	1	4	3	2	1	3	3	0	3	3	7	3
GN1394	2	3	－	2	3	3	0	2	2	3	2	0	2	3	1	1
GN1396	2	6	－	2	2	5	1	2	2	4	3	0	2	3	4	4
GN1397	8	4	－	2	3	4	0	2	2	3	3	0	1	3	6	4
GN1398	5	3	－	2	3	5	0	2	2	2	3	0	3	6	4	4
GN1403	2	6	－	2	3	5	0	2	3	2	3	0	1	4	8	9
GN1405	2	6	－	2	3	5	3	2	2	3	2	0	2	3	3	7
GN1406	5	3	－	2	3	4	0	5	2	3	3	0	2	3	5	7
GN1407	5	6	－	2	3	5	1	2	3	3	3	0	2	6	3	7
GN1408	2	6	－	2	3	4	2	5	2	2	3	0	2	3	8	9
GN1413	2	6	－	2	2	4	1	2	2	3	2	0	2	3	7	4
GN1414	2	6	－	2	2	4	1	2	2	3	3	0	2	3	7	4
GN1415	5	6	－	2	2	3	0	3	2	3	3	0	2	2	7	3
GN1416	6	6	－	2	2	4	0	2	2	2	3	2	3	6	7	4
GN1419	5	6	－	2	3	2	2	2	2	3	3	0	2	5	5	5
GN1421	5	6	－	2	2	4	1	5	2	3	3	0	2	5	7	2
GN1422	6	3	－	2	2	3	1	2	2	3	3	2	3	3	7	3
GN1423	5	2	－	2	2	2	2	2	2	2	2	0	2	3	7	3
GN1424	2	3	－	2	2	3	1	2	2	3	3	2	2	6	8	9
GN1426	2	6	－	2	2	2	0	2	2	3	2	0	2	4	1	1
GN1428	2	5	－	2	3	5	0	2	3	2	3	0	3	3	6	4
GN1429	2	3	－	2	2	2	2	2	2	3	2	0	2	3	4	4
GN1430	2	3	－	2	3	5	1	2	3	2	3	0	3	3	8	9
GN1431	2	3	－	2	3	5	2	2	3	2	3	0	3	3	8	9
GN1433	2	3	－	2	3	4	3	2	3	3	3	0	2	6	6	4
GN1434	2	3	－	2	3	5	1	2	3	3	3	0	3	3	8	10
GN1437	2	3	－	2	2	4	2	2	2	2	3	3	1	2	7	2
GN1438	2	6	－	2	2	2	1	2	1	3	3	4	2	3	3	4
GN1440	2	3	－	2	3	4	2	2	2	3	2	0	2	6	7	1
GN1442	2	6	－	2	3	5	0	2	2	3	2	0	2	6	7	1

GN1443	2	6	-	2	3	4	1	2	2	3	3	0	2	3	4	4
GN1444	6	6	-	2	2	4	1	2	2	3	3	0	2	3	1	1
GN1445	1	6	-	2	3	2	0	2	1	4	3	0	2	6	9	2
GN1446	2	5	-	2	2	2	2	2	1	3	3	4	2	6	6	5
GN1448	5	6	-	2	2	4	1	2	1	4	3	0	1	6	8	9
GN1450	2	4	-	2	3	5	1	5	2	2	3	0	2	6	9	7
GN1453	2	6	-	2	2	4	3	2	2	3	3	0	2	3	4	2
GN1454	2	6	-	2	3	3	0	2	2	3	2	0	1	4	8	2
GN1455	2	4	-	2	3	5	1	2	2	2	3	0	2	6	6	2
GN1456	2	3	-	2	3	4	2	2	2	3	3	0	2	6	6	2
GN1457	6	3	-	2	2	2	1	2	2	3	2	0	1	2	3	7
GN1458	2	6	-	2	2	2	2	2	2	3	3	0	2	3	4	5
GN1460	6	6	-	2	3	4	2	5	2	3	3	0	2	3	6	4
GN1461	2	6	-	2	2	4	2	2	2	3	2	0	2	4	4	3
GN1462	2	6	-	2	2	4	2	2	2	3	2	0	2	4	7	2
GN1467	2	3	-	2	2	5	3	2	2	3	3	0	2	7	7	1
GN1468	2	6	-	2	1	5	0	2	2	3	2	0	2	7	8	1
GN1471	2	3	-	2	2	2	3	2	3	3	2	0	2	4	4	3
GN1472	2	3	-	2	2	4	1	2	2	3	2	0	2	3	7	1
GN1473	2	6	-	2	2	4	3	2	2	3	2	0	3	4	6	3
GN1474	6	3	-	2	2	5	2	2	2	3	2	0	2	4	2	3
GN1476	2	3	-	2	2	4	3	5	3	3	3	2	2	4	8	1
GN1478	2	3	-	2	2	5	2	2	3	2	2	3	1	4	1	5
GN1479	4	6	-	2	2	4	1	2	2	3	3	2	3	3	8	1
GN1480	1	6	-	2	1	4	3	2	1	3	3	2	4	3	8	1
GN1481	6	6	-	2	3	4	1	5	3	2	2	3	1	7	4	5
GN1482	6	6	-	2	2	4	3	2	2	2	2	0	3	3	1	1
GN1485	2	4	-	2	2	4	2	5	3	3	3	2	2	3	6	2
GN1491	2	3	-	2	3	4	2	2	3	2	3	0	2	3	3	4
GN1492	1	4	-	2	3	4	2	5	3	2	3	0	2	3	4	3
GN1493	5	6	-	2	2	4	0	5	2	2	3	0	1	6	3	4
GN1495	6	4	-	6	2	4	0	5	2	3	3	0	2	4	5	1
GN1497	6	3	-	2	3	4	3	2	2	2	3	0	3	3	2	2
GN1501	2	3	-	2	2	2	3	2	3	3	3	0	2	3	1	3
GN1503	2	4	-	2	2	4	2	5	2	2	3	0	3	3	8	3
GN1504	2	3	-	2	2	2	3	2	3	2	3	0	3	3	8	3
GN1505	2	3	-	2	2	2	3	2	2	3	2	0	3	3	9	3
GN1509	2	3	-	2	2	2	3	2	3	2	3	0	2	3	3	3
GN1512	1	6	-	2	2	5	1	2	2	3	3	2	2	3	6	3
GN1513	1	3	-	2	2	2	1	2	2	3	3	0	2	3	7	4
GN1515	5	6	-	2	3	4	1	2	2	3	3	0	2	4	2	1
GN1516	7	6	-	2	3	4	1	2	2	3	2	0	3	3	2	2
GN1517	2	6	-	2	3	5	1	2	2	3	2	0	2	4	7	2
GN1518	7	6	-	2	3	5	1	2	3	3	3	0	2	3	4	3

GN1519	7	3	-	5	2	2	0	7	2	3	3	0	4	3	2	2
GN1520	7	6	-	2	2	5	0	2	2	3	2	0	2	3	8	1
GN1521	2	6	-	2	2	5	2	2	2	3	3	3	2	3	8	2
GN1522	6	6	-	2	2	5	2	2	3	2	2	4	3	4	1	1
GN1523	2	4	-	2	1	2	1	2	2	2	2	4	3	3	2	2
GN1524	2	3	-	2	2	4	1	2	3	3	2	0	3	3	6	2
GN1525	7	4	-	2	3	4	0	2	3	3	2	0	3	3	1	1
GN1526	2	6	-	2	3	4	1	2	3	2	3	0	3	4	3	2
GN1527	1	3	-	2	3	4	2	2	3	2	3	2	3	3	6	2
GN1530	2	3	-	2	2	3	1	2	2	2	3	0	2	3	8	3
GN1531	2	3	-	2	2	3	0	2	2	4	3	0	3	3	8	2
GN1532	2	6	-	2	3	2	0	2	3	3	3	0	1	3	6	1
GN1533	2	3	-	2	3	2	1	2	3	2	3	0	3	3	8	4
GN1536	2	3	-	2	2	5	0	2	2	2	3	2	2	6	8	7
GN1538	2	3	-	1	3	3	3	2	2	3	3	0	2	3	2	3
GN1540	2	3	-	2	3	4	2	2	2	3	3	0	2	6	8	5
GN1541	2	3	-	2	3	3	0	2	2	3	3	0	2	4	8	7
GN1542	2	3	-	2	3	3	1	2	2	3	3	0	2	4	8	7
GN1543	2	3	-	2	2	4	2	2	2	3	3	2	2	3	9	4
GN1545	5	3	-	2	3	5	2	2	2	2	3	0	2	3	9	2
GN1546	6	6	-	2	3	4	2	2	2	3	2	2	2	3	8	4
GN1548	1	6	-	2	3	5	0	2	2	3	2	2	2	3	9	9
GN1550	2	3	-	2	3	4	0	2	2	4	3	0	2	3	4	3
GN1551	2	3	-	2	3	5	2	2	2	3	3	0	3	3	9	9
GN1552	2	3	-	2	2	2	0	2	2	3	2	0	2	3	1	1
GN1553	1	3	-	2	-	5	2	3	-	-	3	0	-	6	2	4
GN1554	2	6	-	2	1	4	3	5	1	3	3	0	1	3	9	9
GN1555	2	6	-	2	3	2	2	2	2	3	3	0	2	3	5	4
GN1557	2	6	-	2	2	4	0	6	2	3	2	0	2	3	9	3
GN1562	2	6	-	2	3	5	1	6	3	3	2	0	2	3	9	1
GN1564	2	6	-	2	3	4	1	2	2	3	2	0	2	4	6	1
GN1566	5	3	-	2	2	2	1	2	2	3	3	0	2	6	8	3
GN1571	2	5	-	2	3	4	0	2	2	3	3	0	2	3	9	2
GN1572	6	6	-	2	3	4	2	2	2	2	3	0	1	5	8	4
GN1573	2	6	-	2	3	5	0	6	2	3	2	0	2	3	9	9
GN1576	2	3	-	2	2	4	3	2	2	2	3	0	2	3	8	7
GN1577	2	3	-	2	2	2	0	2	3	3	2	0	2	3	1	2
GN1578	2	3	-	2	2	4	2	2	3	2	3	0	2	3	8	2
GN1579	2	3	-	2	3	2	0	2	3	2	3	0	2	6	6	2
GN1580	2	3	-	2	3	5	1	3	2	3	3	0	2	4	9	1
GN1581	6	6	-	2	3	4	1	2	2	3	2	0	2	6	9	5
GN1582	2	6	1	2	3	2	0	2	2	2	1	0	2	3	2	2
GN1583	2	6	1	2	3	4	1	2	2	3	2	0	1	2	8	5
GN1584	2	3	2	2	2	2	2	2	1	3	3	2	2	6	3	7

GN1585	1	6	1	2	2	2	0	1	2	3	2	0	2	3	2	1
GN1587	3	5	2	2	2	2	2	2	3	2	1	2	2	3	9	10
GN1588	1	3	2	2	–	2	1	2	–	–	3	0	–	3	9	10
GN1589	2	6	1	2	2	4	1	2	2	3	2	0	2	3	1	9
GN1590	1	2	2	2	3	5	0	2	2	2	3	0	2	3	9	10
GN1591	6	6	1	2	2	5	2	5	2	3	3	3	2	3	8	4
GN1592	1	6	1	1	3	3	0	1	2	4	2	0	3	3	1	1
GN1593	5	6	1	2	1	4	2	2	1	3	3	3	1	7	4	4
GN1594	3	2	1	2	1	2	0	2	2	1	3	0	1	4	8	3
GN1596	5	5	2	2	1	4	1	2	2	2	3	2	1	2	7	3
GN1598	1	4	1	2	2	2	1	2	3	4	2	0	2	3	2	2
GN1602	6	3	1	2	2	2	0	2	2	3	2	0	2	6	3	4
GN1603	2	3	1	2	2	5	1	5	2	3	2	3	1	6	6	2
GN1604	3	2	2	2	2	4	1	2	2	3	3	0	2	6	3	5
GN1606	3	6	2	2	2	2	1	2	2	2	3	1	2	3	9	10
GN1607	5	6	2	2	3	4	0	2	1	4	2	1	2	3	8	2
GN1611	7	6	2	2	2	5	2	2	2	4	2	2	1	3	4	3
GN1615	6	5	1	2	1	3	2	2	1	3	3	0	1	3	4	4
GN1618	3	6	2	2	2	2	0	7	2	3	3	2	3	3	9	9
GN1619	6	6	2	2	3	4	2	2	2	3	3	3	2	3	8	4
GN1622	1	6	1	1	2	3	1	2	2	3	2	0	1	4	1	2
GN1623	1	6	2	2	2	4	1	2	2	2	3	0	1	3	3	5
GN1624	6	2	2	2	1	3	1	7	2	2	3	3	1	3	7	7
GN1625	2	6	1	2	1	2	1	7	1	3	3	3	2	6	9	10
GN1626	7	6	2	1	2	3	0	2	2	2	3	3	2	5	7	7
GN1627	2	6	2	2	2	5	0	5	3	3	2	0	2	2	7	9
GN1629	5	6	1	2	2	5	1	5	2	3	2	1	1	3	4	3
GN1630	2	3	1	2	2	4	2	2	2	2	3	2	1	3	7	3
GN1632	7	6	1	2	1	5	1	2	1	3	3	0	2	6	9	9
GN1633	3	3	2	1	1	1	1	1	2	4	3	0	1	4	9	9
GN1634	2	6	1	2	1	4	2	2	1	3	3	2	1	6	1	4
GN1636	1	3	2	2	1	5	0	2	2	3	3	0	1	2	7	2
GN1637	3	2	1	2	1	5	1	2	1	3	2	0	1	6	8	4
GN1639	5	6	1	2	–	3	1	2	–	–	3	4	–	3	4	3
GN1640	6	3	1	3	–	5	3	6	–	–	3	0	–	3	9	9
GN1661	3	6	2	2	1	2	1	2	2	2	3	2	2	6	9	9
A232	2	3	2	2	–	4	1	2	–	–	3	3	–	3	8	2
A236	3	6	1	2	–	5	0	5	–	–	3	0	–	6	8	1
A237	7	6	2	2	–	4	0	2	–	–	2	0	–	6	4	3
A240	2	6	1	2	–	4	2	2	–	–	3	0	–	5	8	1
A242	2	3	2	2	–	4	3	2	–	–	3	2	–	2	3	7
A247	5	5	1	2	–	5	2	5	–	–	3	0	–	3	8	1
A252	2	6	1	2	–	4	2	2	–	–	2	0	–	6	7	2
A263	2	3	2	2	–	2	1	2	–	–	3	0	–	3	9	10

A271	3	6	1	2	−	5	1	5	−	−	2	2	−	6	4	3
A276	6	4	1	2	−	2	2	2	−	−	2	2	−	2	9	9
A278	5	6	1	2	−	5	1	6	−	−	2	0	−	2	9	9
A280	3	5	1	2	−	5	3	5	−	−	3	0	−	6	7	2
A284	7	2	1	2	−	2	1	2	−	−	2	3	−	4	7	3
A331	−	−	−	−	−	−	−	−	−	−	−	−	−	−	−	−
A346	−	−	−	−	−	−	−	−	−	−	−	−	−	−	−	−
A348	−	−	−	−	−	−	−	−	−	−	−	−	−	−	−	−
A354	−	−	−	−	−	−	−	−	−	−	−	−	−	−	−	−
A369	−	−	−	−	−	−	−	−	−	−	−	−	−	−	−	−
A377	−	−	−	−	−	−	−	−	−	−	−	−	−	−	−	−
A393	6	6	1	2	−	4	1	2	−	−	3	1	−	3	2	1
A397	3	6	1	2	−	4	0	2	−	−	3	0	−	2	4	4
A400	3	6	2	2	−	2	1	2	−	−	3	1	−	3	9	9
A402	2	6	2	2	−	2	1	2	−	−	3	3	−	2	9	9
A403	2	3	1	2	−	4	0	2	−	−	2	0	−	4	4	1
A421	1	6	1	1	−	4	1	1	−	−	2	0	−	6	4	2
A422	6	6	1	2	−	5	0	2	−	−	2	2	−	6	7	9
A423	1	6	2	1	−	4	2	5	−	−	2	0	−	1	8	1
A429	3	6	2	1	−	3	3	5	−	−	3	0	−	4	4	4
A430	3	6	1	1	−	1	2	2	−	−	2	0	−	6	6	1
A442	1	4	1	1	−	2	2	2	−	−	2	0	−	6	9	9
A445	−	−	−	−	−	−	−	−	−	−	−	−	−	−	−	−
A447	2	6	1	2	−	3	1	−	−	−	3	0	−	2	3	7
A455	3	5	2	1	−	4	0	2	−	−	2	0	−	3	8	5
A457	2	3	1	2	−	2	1	2	−	−	3	0	−	6	8	8
A468	3	6	2	2	−	5	0	6	−	−	2	2	−	5	8	4
A476	2	6	1	2	−	5	1	3	−	−	2	2	−	3	7	4
A478	7	6	2	2	−	2	3	5	−	−	2	0	−	6	4	2
A493	7	6	2	2	−	5	2	2	−	−	2	0	−	6	7	2
A494	1	6	1	1	−	1	0	2	−	−	2	1	−	3	8	5
A508	2	6	1	2	−	5	1	2	−	−	2	1	−	2	4	4
A517	3	6	1	2	−	5	0	6	−	−	3	3	−	6	7	9
A521	5	3	1	2	−	2	2	2	−	−	3	2	−	3	7	3
A523	2	3	2	2	−	4	1	2	−	−	2	3	−	3	8	2
A534	2	6	1	1	−	4	1	2	−	−	3	2	−	2	2	5
A540	2	6	1	2	−	3	1	2	−	−	3	3	−	3	8	9
A542	1	5	1	1	−	5	0	2	−	−	2	1	−	6	8	2
A552	1	6	2	1	−	2	1	2	−	−	3	3	−	3	9	9
A555	1	6	1	2	−	4	1	3	−	−	3	0	−	3	7	7
A557	6	6	1	2	−	2	2	2	−	−	2	0	−	4	4	4
A562	4	6	1	2	−	5	2	2	−	−	3	3	−	6	3	7
A567	6	6	2	2	−	3	2	6	−	−	3	0	−	6	7	3
A570	1	6	1	2	−	4	0	2	−	−	2	3	−	3	9	9

A571	2	6	2	2	–	4	0	2	–	–	2	3	–	6	6	1
A579	5	6	1	1	–	2	0	2	–	–	3	0	–	5	2	3
A587	7	3	1	1	–	2	1	2	–	–	3	4	–	6	8	9
A588	6	6	1	2	–	4	1	5	–	–	3	3	–	3	4	3
A589	2	6	2	2	–	3	1	2	–	–	2	2	–	3	4	3
A591	2	3	2	2	–	5	2	2	–	–	2	3	–	3	7	2
A597	1	6	1	2	–	4	1	2	–	–	3	2	–	3	9	9
A604	2	6	2	2	–	4	2	2	–	–	2	2	–	5	9	9
A605	2	6	1	2	–	5	1	2	–	–	2	1	–	3	4	4
A610	1	6	1	2	–	5	0	2	–	–	2	2	–	3	4	4
A620	2	6	1	1	–	5	1	2	–	–	3	0	–	2	7	3
A621	2	5	2	1	–	3	2	2	–	–	3	1	–	3	8	3
A625	1	6	1	2	–	3	0	2	–	–	2	0	–	6	8	1
A628	2	3	1	2	–	3	0	1	–	–	3	0	–	4	6	2
A631	–	–	–	–	–	–	–	–	–	–	–	–	–	–	–	–
A634	6	3	1	6	–	5	1	6	–	–	3	0	–	3	8	2
A638	2	3	2	2	–	2	0	2	–	–	3	4	–	4	8	2
A639	–	–	–	–	–	–	–	–	–	–	–	–	–	–	–	–
A640	3	3	1	1	–	4	1	2	–	–	3	0	–	3	2	3
A641	2	6	2	2	–	5	2	2	–	–	2	0	–	3	6	4
A646	1	4	1	1	–	2	1	2	–	–	3	2	–	2	9	9
A647	5	5	1	2	–	3	1	2	–	–	3	1	–	6	7	7
A652	2	6	2	2	–	3	3	2	–	–	2	3	–	3	9	9
A653	2	6	2	2	–	3	1	2	–	–	3	2	–	6	9	9
A654	6	6	2	2	–	1	1	2	–	–	2	1	–	4	8	5
A659	5	6	2	2	–	5	1	2	–	–	2	2	–	6	8	2
A660	5	3	1	2	–	3	0	2	–	–	3	2	–	3	8	3
A662	8	6	2	8	–	3	2	1	–	–	3	2	–	5	8	3
A664	6	4	1	2	–	5	1	3	–	–	2	3	–	6	8	4
A665	2	4	1	2	–	4	0	2	–	–	2	2	–	6	4	3
A666	1	6	2	2	–	3	1	2	–	–	2	3	–	6	8	3
A668	1	3	2	2	–	4	1	5	–	–	2	2	–	3	8	9
A669	1	6	1	2	–	3	1	2	–	–	2	0	–	1	8	3
A672	6	5	1	2	–	2	0	2	–	–	2	2	–	4	3	3
A674	6	4	1	6	–	5	2	6	–	–	3	0	–	8	7	3
A681	2	6	1	2	–	5	1	2	–	–	2	3	–	4	4	3
A683	1	3	1	1	–	3	0	1	–	–	3	2	–	6	7	5
A689	2	6	1	2	–	4	1	2	–	–	2	1	–	6	8	2
A690	5	6	2	2	–	5	0	2	–	–	2	2	–	3	4	3
A691	2	3	1	2	–	5	1	2	–	–	2	2	–	4	8	1
A694	1	4	2	2	–	3	0	2	–	–	2	3	–	3	2	3
A698	2	6	2	2	–	1	1	2	–	–	3	2	–	6	4	3
A699	4	6	1	2	–	2	0	2	–	–	2	0	–	6	4	2
A700	3	3	1	2	–	4	3	5	–	–	2	2	–	6	2	2

A701	1	6	1	1	−	4	1	2	−	−	2	0	−	6	4	2
A702	6	6	1	2	−	5	0	6	−	−	3	1	−	3	8	2
A703	3	6	1	2	−	3	2	2	−	−	3	2	−	6	9	9
A704	1	6	1	2	−	4	1	2	−	−	2	0	−	3	9	4
A706	5	3	2	1	−	3	1	2	−	−	3	2	−	6	8	3
A709	5	3	2	2	−	3	0	2	−	−	3	2	−	3	7	5
A712	1	3	2	2	−	4	1	5	−	−	2	3	−	3	7	2
A713	3	6	1	2	−	5	0	2	−	−	2	2	−	3	9	9
A715	2	6	1	2	−	5	1	2	−	−	2	3	−	3	8	9
A716	3	6	2	2	−	5	1	2	−	−	2	3	−	6	9	9
A717	1	4	2	2	−	5	2	2	−	−	3	1	−	4	4	2
A718	3	6	1	2	−	5	2	2	−	−	2	3	−	3	8	3
A719	3	6	1	2	−	5	2	2	−	−	2	3	−	3	8	3
A722	6	4	2	2	−	5	0	5	−	−	2	2	−	4	7	1
A724	6	6	1	2	−	3	1	2	−	−	3	2	−	3	3	4
A726	5	6	1	2	−	2	1	2	−	−	2	4	−	3	3	7
A729	1	5	2	2	−	4	1	2	−	−	2	2	−	3	8	5
A731	2	3	2	2	−	4	1	5	−	−	3	4	−	6	3	5
A737	1	3	2	1	−	3	1	1	−	−	3	1	−	6	8	7
A742	1	3	2	2	−	4	3	2	−	−	3	2	−	6	7	7
A744	1	3	2	2	−	3	1	2	−	−	3	3	−	3	7	7
A746	1	6	1	2	−	3	1	7	−	−	3	1	−	2	4	3
A748	1	6	2	2	−	4	0	2	−	−	3	0	−	6	4	4
A749	1	6	2	2	−	4	0	4	−	−	2	0	−	6	4	4
A751	1	2	2	2	−	2	3	2	−	−	2	2	−	6	3	4
A752	7	3	2	2	−	3	1	2	−	−	3	3	−	6	7	3
A753	1	6	2	2	−	2	2	2	−	−	3	0	−	6	8	4
A757	3	6	2	2	−	5	1	2	−	−	2	0	−	−	−	−
A759	1	3	2	1	−	4	0	2	−	−	3	0	−	3	7	5
A760	1	4	1	2	−	4	3	2	−	−	2	0	−	−	−	−
A764	2	3	1	2	−	5	0	6	−	−	3	2	−	3	8	4
A765	7	6	1	2	−	5	0	3	−	−	2	0	−	−	−	−
A768	2	3	2	2	−	1	2	2	−	−	4	4	−	6	3	5
A769	1	3	2	2	−	4	0	3	−	−	3	0	−	6	7	7
A770	−	−	−	−	−	−	−	−	−	−	−	−	−	−	−	−
A772	1	3	2	2	−	3	0	2	−	−	3	0	−	3	4	4
A779	2	4	2	2	−	3	0	2	−	−	2	2	−	6	8	3
A781	−	−	−	−	−	−	−	−	−	−	−	−	−	−	−	−
A782	7	6	1	2	−	2	1	2	−	−	2	0	−	3	8	2
A783	2	6	2	1	−	4	2	2	−	−	3	0	−	2	8	3
A784	2	3	1	2	−	4	2	2	−	−	2	0	−	6	8	2
A785	2	3	1	2	−	5	0	3	−	−	2	0	−	3	7	3
A787	1	6	1	2	−	4	1	2	−	−	2	0	−	6	8	2
A790	1	4	1	2	−	3	0	2	−	−	3	1	−	6	8	9

A792	1	6	2	2	–	3	0	2	–	–	3	0	–	3	8	2
A793	1	6	1	1	–	3	2	1	–	–	3	0	–	6	8	2
A794	2	5	1	1	–	2	0	2	–	–	3	1	–	6	8	1
A795	2	6	1	2	–	4	2	2	–	–	2	1	–	3	8	2
A796	1	6	2	2	–	2	2	2	–	–	2	1	–	2	4	3
A798	3	6	2	2	–	2	1	2	–	–	3	2	–	6	9	9
A799	1	4	1	2	–	4	2	2	–	–	3	0	–	6	8	2
A800	2	3	1	2	–	1	0	2	–	–	3	0	–	6	8	3
A801	3	6	1	2	–	5	1	6	–	–	2	1	–	6	9	9
A803	2	3	1	2	–	4	2	2	–	–	3	0	–	4	8	3
A803	2	3	1	2	–	4	2	2	–	–	3	0	–	4	8	3
A806	6	6	1	2	–	4	0	2	–	–	3	0	–	6	9	9
A808	2	6	1	1	–	4	2	1	–	–	2	0	–	6	8	3
A813	6	4	1	6	–	4	0	3	–	–	3	1	–	3	8	3
A814	8	6	2	8	–	2	0	1	–	–	3	0	–	4	7	2
A815	1	6	2	5	–	4	0	3	–	–	3	0	–	8	8	3
A816	3	6	2	2	–	4	1	2	–	–	2	2	–	6	8	4
A817	2	6	2	2	–	4	1	2	–	–	2	2	–	5	8	4
A819	2	6	1	2	–	4	1	2	–	–	2	2	–	6	4	4
A821	2	6	1	2	–	5	1	2	–	–	3	0	–	3	4	3
A824	3	3	2	2	–	5	2	2	–	–	2	0	–	6	7	4
A827	1	5	1	2	–	1	0	1	–	–	3	2	–	3	8	9
A828	3	5	1	2	–	4	0	2	–	–	2	2	–	6	4	2
A829	2	6	2	2	–	4	1	2	–	–	3	2	–	6	8	2
A830	1	3	1	2	–	2	0	1	–	–	3	2	–	3	8	9
A832	1	6	2	2	–	4	0	2	–	–	2	2	–	6	9	9
A833	7	3	2	2	–	3	0	2	–	–	2	0	–	6	7	5
A834	2	6	2	2	–	4	1	2	–	–	3	0	–	6	9	9
A835	3	6	2	2	–	3	0	2	–	–	2	3	–	6	9	9
A836	6	6	1	2	–	2	0	2	–	–	2	2	–	6	4	3
A839	3	3	1	2	–	3	1	2	–	–	3	0	–	6	7	5
A840	7	3	1	2	–	2	0	2	–	–	2	2	–	4	7	5
A842	1	5	2	1	–	4	2	2	–	–	3	0	–	3	3	2
A843	1	6	2	2	–	4	2	2	–	–	3	2	–	3	9	9
A845	2	6	2	2	–	3	0	2	–	–	2	2	–	3	8	2
A847	1	3	2	2	–	5	1	2	–	–	2	2	–	3	8	9
A849	6	6	2	2	–	4	0	2	–	–	4	0	–	6	7	7
A854	6	6	2	2	–	3	1	4	–	–	3	0	–	4	9	9
A855	2	3	1	2	–	4	1	2	–	–	2	0	–	6	8	3
A856	1	6	1	2	–	4	0	2	–	–	3	2	–	6	8	2
A859	–	–	–	–	–	–	–	–	–	–	–	–	–	–	–	–
A863	5	6	1	2	–	2	0	2	–	–	2	0	–	6	8	4
A869	9	6	1	2	–	2	0	2	–	–	3	2	–	3	4	1
A888	2	3	1	2	–	5	2	2	–	–	2	0	–	6	8	2

A889	6	3	2	2	–	3	0	2	–	–	2	3	–	3	3	5
A890	6	3	1	2	–	4	0	2	–	–	3	3	–	3	3	7
A905	5	6	2	2	–	2	0	2	–	–	3	3	–	3	2	3
A906	2	6	1	2	–	2	1	2	–	–	2	2	–	3	4	3
A907	1	6	1	2	–	2	1	2	–	–	2	2	–	4	2	2
A908	2	6	1	2	–	2	0	2	–	–	2	2	–	4	4	2
A909	2	6	2	2	–	2	0	2	–	–	2	3	–	6	1	1
A910	5	6	1	5	–	5	0	6	–	–	2	4	–	9	4	3
A912	3	3	1	2	–	3	2	2	–	–	2	2	–	4	9	10
A913	1	5	1	2	–	2	1	2	–	–	2	2	–	2	4	4
A914	2	3	2	2	–	2	1	2	–	–	2	2	–	4	9	10
A915	2	6	2	2	–	2	2	2	–	–	2	3	–	3	9	9
A917	6	6	2	5	–	5	0	6	–	–	2	1	–	9	8	3
A918	2	6	2	2	–	2	2	2	–	–	2	1	–	3	3	5
A968	–	–	–	–	–	–	–	–	–	–	–	–	–	–	–	–
A969	–	–	–	–	–	–	–	–	–	–	–	–	–	–	–	–
A970	–	–	–	–	–	–	–	–	–	–	–	–	–	–	–	–
A971	–	–	–	–	–	–	–	–	–	–	–	–	–	–	–	–
A997	–	–	–	–	–	–	–	–	–	–	–	–	–	–	–	–
